# Development of a Continuous Flow Baldwin Rearrangement Process and Its Comparison to Traditional Batch Mode

**DOI:** 10.1021/acs.oprd.3c00213

**Published:** 2023-09-11

**Authors:** Arlene Bonner, Marcus Baumann

**Affiliations:** † School of Chemistry, University College Dublin, Science Centre South, Belfield, Dublin 4, Ireland D04 N2E2

**Keywords:** aziridine, Baldwin rearrangement, flow chemistry, high throughput, scale-up

## Abstract

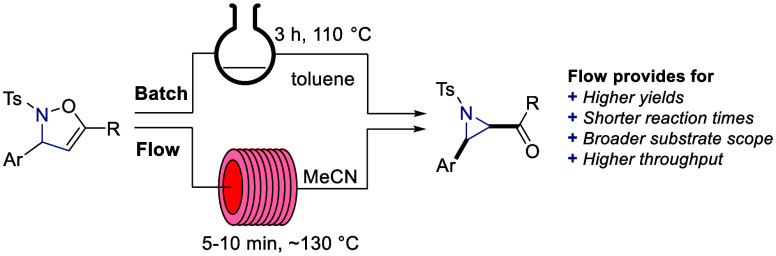

A new and highly efficient continuous flow process is presented for the synthesis of aziridines via the thermal Baldwin rearrangement. The process was initially explored using traditional batch synthesis techniques but suffered from moderate yields, long reaction times, and moderate diastereoselectivities. Here we demonstrate that the process can be greatly improved upon its transfer to continuous flow, which afforded the aziridine targets in high yields, short reaction times, and consistently high diastereoselectivities, with the high-throughput process rendering multigram quantities of product in short periods of time. In addition, flow processing extended the substrate scope including several examples that had failed in batch mode, thus demonstrating the value of this overlooked entry into valuable aziridine species.

## Introduction

**Figure 1:**
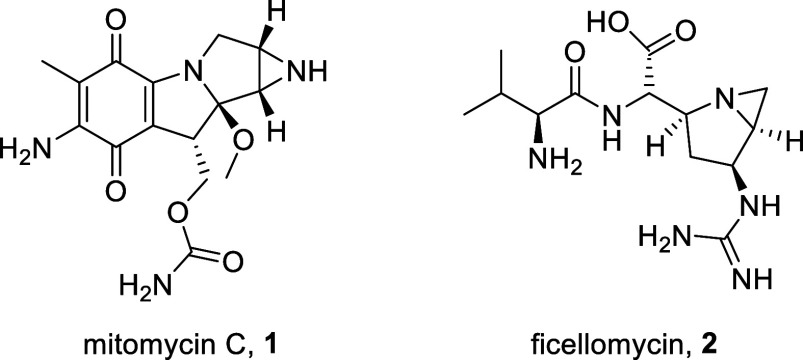
Structures of mitomycin C and ficellomycin.

Heterocyclic chemistry is said to constitute around 65 percent of the organic chemistry literature.^1^ Aziridines are a class of versatile building blocks and are one of the most underexplored heterocycles, especially in comparison to epoxides and their five- and six-membered ring analogues, despite their various biological and industrial applications.^2,3^ Perhaps their most well-known applications are their use in bioactive molecules (Figure 1), which display anticancer and antibacterial properties, including mitomycin C (**1**) and ficellomycin (**2**).^4^

**Scheme 1:**
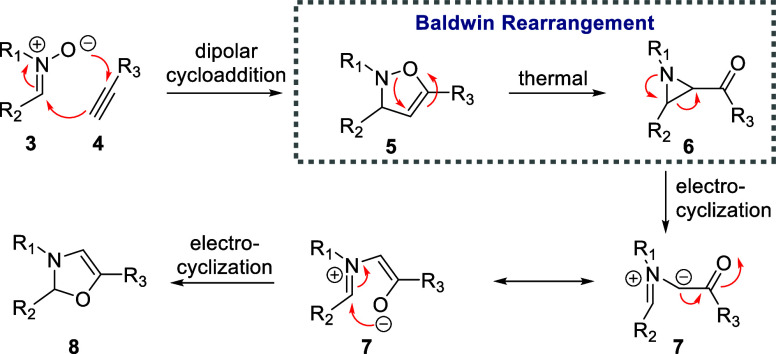
General Features of the Baldwin Rearrangement and Potential Competing Pathways

While numerous aziridine syntheses exist,^5^ such as the classical method of alkene aziridination via addition of nitrenes, one overlooked method is the Baldwin rearrangement.^6^ The Baldwin rearrangement describes the thermally induced ring contraction of 2,3-dihydroisoxazoles into acyl aziridines, and although discovered already in 1968, the rearrangement has largely been forgotten about since then. Baldwin’s seminal work was followed by studies that revealed that the nature of the substituents greatly affects the progress of the reaction, with electron withdrawing groups and strongly donating nitrogen substituents favoring this 1,3-sigmatropic rearrangement.^6,7^ As the Baldwin rearrangement is typically thermally driven, side reactions arising from the electrocyclic ring opening of the aziridine product are often observed leading to oxazolines (**8**) via azomethine ylide intermediates (**7**, Scheme 1).^6,8^ Poor control over heat transfer is the main culprit in this case that presents a limitation of existing Baldwin rearrangement protocols in batch reactors. It has been found that the opening of aziridines to azomethine ylides can be affected by factors such as steric hindrance, bridgehead ring systems adjacent to the nitrogen atom, and the electronic properties of their *N*-substituents.^9^

To date, the sparse literature on the Baldwin rearrangement highlights studies that have been performed using batch or microwave irradiation methods. Although useful, these syntheses suffer from long reaction times (up to 72 h), variable yields, limited substrate scope, or limited scalability.^10,11^ We set out to overcome these drawbacks using continuous flow technology. Numerous studies have demonstrated that performing chemical reactions in continuous mode, using tubing as vessels, leads to a number of advantages because of improved heat and mass transfer, scalability, safety, and reproducibility.^12^ These are exploited to improve chemical processes and, often, to perform chemical reactions that have been forgotten or are forbidden in batch.^13^ In addition, continuous flow reactors have been exploited with great success for the effective generation of many other heterocyclic building blocks with potential industrial uses.^14^

We therefore envisaged developing a continuous flow synthesis of aziridines via the thermal Baldwin rearrangement. The report presented herein describes the optimization and synthesis of a library of aziridines in both batch and flow mode, with the flow results contrasting to the batch results in terms of improved yields, reaction time, diastereoselectivity, substrate scope, and scalability.

## Results and Discussion

**Scheme 2:**
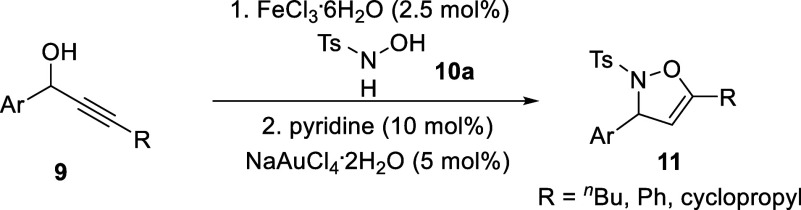
Synthesis of 2,3-Dihydroisoxazoles **11**

The initial step toward the Baldwin rearrangement involved the synthesis of 2,3-dihydroisoxazoles from the corresponding propargylic alcohols and *N*-hydroxylsulfonamide, prepared via literature-known procedures.^8c,15,16^ Using these, a variety of isoxazolines were synthesized (Scheme 2), which also identified limitations of this procedure, such as the use of alkyl or heteroaromatic substituents instead of aromatics (see Supporting Information for full details).

**Scheme 3:**
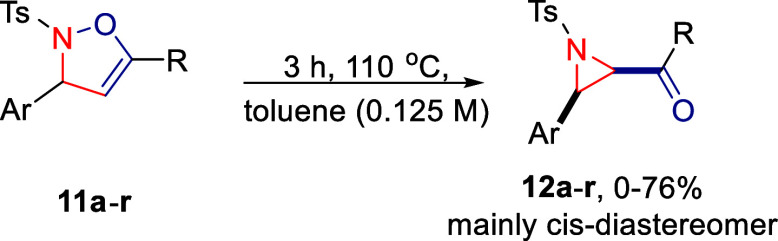
Batch Synthesis of Aziridines (**12**) via the Thermally Induced Baldwin Rearrangement

With a library of isoxazolines at hand bearing both electron-rich and electron-poor aryl groups and aromatic and aliphatic R groups, the thermal Baldwin rearrangement was studied under batch and continuous flow conditions using isoxazoline **11a** (Ar = 2,4-difluorophenyl, R = ^*n*^Bu) as a model substrate. As previously discussed, Baldwin demonstrated that electron-rich substituents favor the rearrangement, and consequently, high temperatures are typically required if the isoxazoline possesses electron withdrawing nitrogen substituents.^6,8c^ Therefore, a solution of isoxazoline **11a** was heated at reflux in toluene, and the reaction was monitored by ^1^H NMR hourly. Full conversion of **11a** (60% ^1^H NMR yield) was observed after 3 h, and these conditions were employed for the Baldwin rearrangement in batch (Scheme 3), resulting in the corresponding *cis*-aziridines as the major diastereomer based on previously reported ^3^*J*_*H–H*_ values and DFT studies of *N*-sulfonylaziridines.^7a,17^

**Scheme 4:**
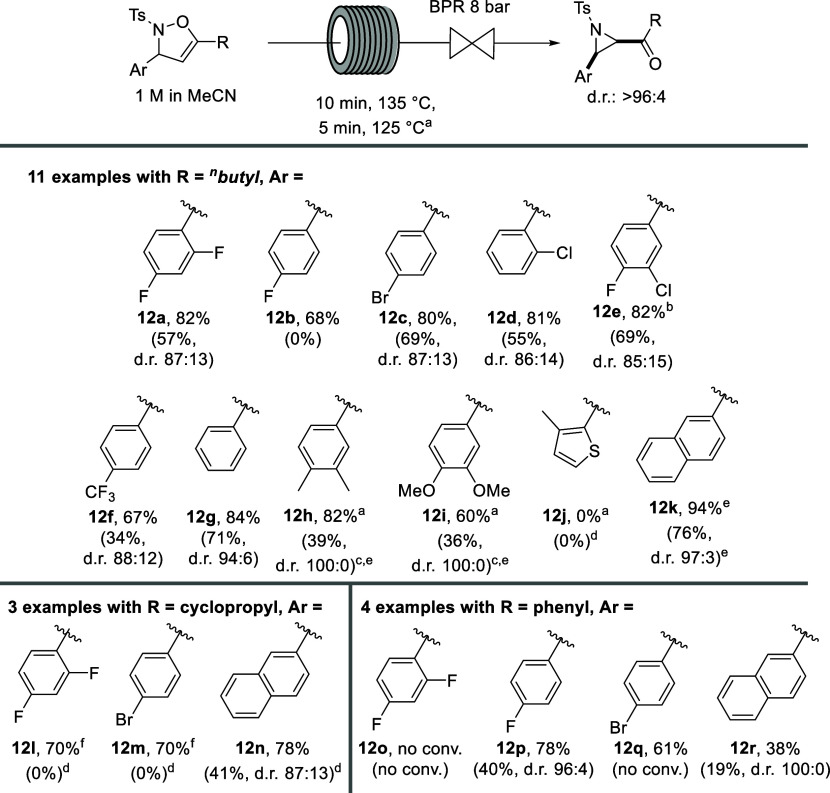
Substrate Scope and Isolated Yields for the Continuous Flow Synthesis of Aziridines Batch yields and diastereoselectivities are indicated in brackets for comparison. Batch reaction conditions: 3 h reflux in toluene, 0.125 M. Flow reaction conditions: 5 min residence time, 125 °C, 8 bar, 1 M in MeCN. 0.125 M. 6 h reflux in MeCN, 0.125 M. 1 h reflux in toluene, 0.125 M. ^1^H NMR yield. 0.15 M.

The study of the thermal Baldwin rearrangement under batch conditions revealed variability in both product yield and diastereoselectivity (for individual values, see Scheme 4). As this observation was often accompanied by substantial amounts of unidentified side products, which likely are related to alternative reaction pathways (see Scheme 1), the process was translated to a continuous flow setup, with model substrate **11a** employed in the optimization study. It was hypothesized that the superior reaction control in flow mode along with the ability to superheat the reaction mixture and thus effectively use low-boiling solvents may have a beneficial impact on the reaction outcome.

Initially, optimization experiments were performed at a concentration of 0.125 M in toluene and focused on maintaining similar temperatures to the batch rearrangement while significantly reducing the residence time, facilitated by the superior heat transfer provided by a continuous flow system. Two experiments were carried out 10 °C below the batch temperature at 100 °C, with residence times of 30 and 90 min, respectively (Table 1, entries 1 and 2). While these conditions resulted in some aziridine formation, large amounts of starting material remained. Consequently, the residence time of 90 min was maintained, and the temperature was increased incrementally by 10 °C from 110 to 140 °C (Table 1, entries 3–6), enabled by the 100 PSI back-pressure regulator (BPR). The temperature of 110 °C gave the highest yield of 78% for aziridine **12a**, with little isoxazoline **11a** remaining (8%). Further increasing the temperature beyond 110 °C with a 90 min residence time resulted in a drop in yield of aziridine due to decomposition, with complete consumption of the starting material (Table 1, entries 4–6). As it was desirable to have a shorter residence time in view of throughput, the residence time was reduced to 30, 45, and 60 min, and the temperature was increased to 120 °C to ensure almost full consumption of starting material (Table 1, entries 7–10). The incremental increase in residence time from 30 to 90 min resulted in increasing ^1^H NMR yields (61–79%) with residence time, with little to no starting material remaining. Finally, the residence time was significantly reduced to 6–15 min, and temperature was increased to 130 °C (Table 1, entries 11–15). The longer residence times of 12–15 min resulted in the highest ^1^H NMR yields of aziridine **12a** with some isoxazoline remaining, while the shorter residence times of 6 and 9 min resulted in lower yields of 51 and 58%, respectively, with more starting material remaining (entries 14–15). The highest yield of 83% aziridine was achieved with a residence time of 12 min at 130 °C. These data indicate that the thermal Baldwin rearrangement benefits from shorter residence times at elevated temperatures, which provide for high substrate conversion and minimal competitive degradation of the aziridine products. When the experiment was repeated with the conditions described above (12 min at 130 °C), however, the ^1^H NMR yield fluctuated between 48 and 83%. After the method of ^1^H NMR sample preparation was verified, the only remaining variable in the experiment was the pressure applied by the 100 PSI BPR, which fluctuated between 6 and 8 bar. To mitigate this fluctuation, one of the peristaltic pumps of the Vapourtec easy-Scholar flow system was used as the BPR, and the pressure was set to 8 bar, resulting in a reproducible yield of 83%. Although 5 bar of pressure was also confirmed to be sufficient to achieve an 83% yield of aziridine **12a** in toluene at 130 °C, the higher pressure of 8 bar was selected in case higher temperatures were required to achieve high yields for other substrates.

**Table 1:**
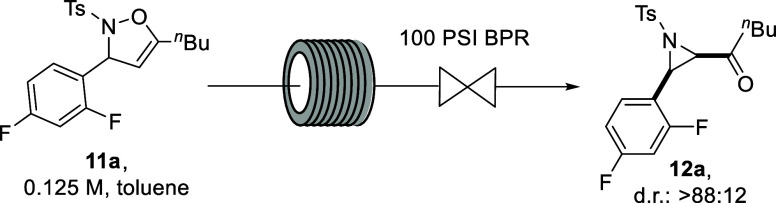
Continuous Flow Optimization of the Thermal Baldwin Rearrangement of Substrate **11a**

Entry	Residence time	Temperature	^1^H NMR yield of **12a**^a^	Remaining **11a**^a^
1	30 min	100 °C	28%	47%
2	90 min	100 °C	40%	50%
3	90 min	110 °C	78%	8%
4	90 min	120 °C	47%	0%
5	90 min	130 °C	42%	0%
6	90 min	140 °C	0%	0%
7	30 min	120 °C	61%	10%
8	45 min	120 °C	78%	0%
9	60 min	120 °C	79%	0%
10	40 min	120 °C	63%	5%
11	15 min	130 °C	63%	0%
12	12 min	130 °C	48–83%	14–46%
13	13 min	130 °C	74%	12%
14	9 min	130 °C	58%	33%
15	6 min	130 °C	51%	38%

a Determined by ^1^H NMR of the crude reaction mixture using 1,2-bis(bromomethyl)benzene as an internal standard.

**Figure 2:**
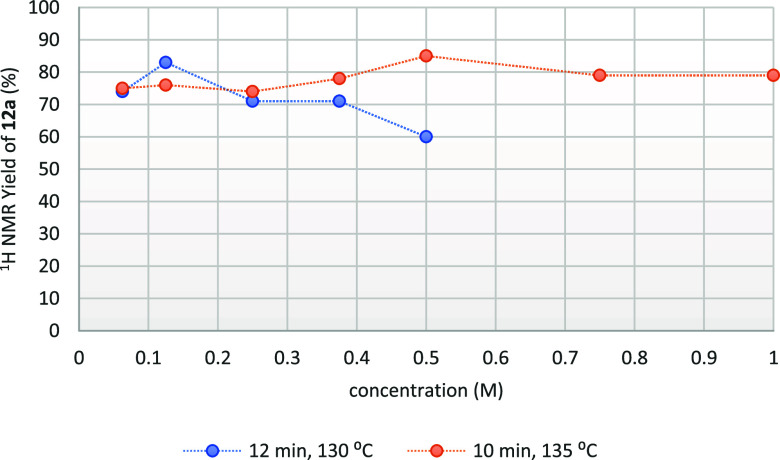
Effect of the concentration on the yield of aziridine **12a**.

Subsequently, a concentration study was performed in toluene varying the concentration from 0.0625 to 0.5 M using the optimized flow conditions of a residence time of 12 min and a temperature of 130 °C (Figure 2). Under these conditions, it was found that 0.125 M was the optimum concentration as the yield decreased above 0.125 M, with 0.5 M yielding only 60% of aziridine **12a**. With the aim of having a reaction with high throughput, it was desirable to reoptimize the conditions of residence time and temperature to achieve higher yields at higher concentrations. Further experimentation showed that a residence time of 10 min and temperature of 135 °C resulted in high yields in more concentrated solutions. The highest ^1^H NMR yield under these conditions was 85% at 0.5 M, but doubling the concentration to 1 M resulted in a small drop in yield to 79%. The remaining optimization experiments were therefore performed at 135 °C, with a residence time of 10 min in a 1 M solution, resulting in high-throughput reaction conditions, as shown in Figure 2.

Finally, a solvent study was performed with the aim of increasing the diastereomeric ratio of the product. The study revealed that solvents such as CPME and 1,1,1-trifluoromethylbenzene gave similar diastereoselectivities to toluene, whereas chlorobenzene gave a slightly higher diastereoselectivity. In view of overall performance and greenness, MeCN was identified as the best solvent choice of the study (Table 2, entry 2). The positive effect of MeCN on both the product yield and diastereoselectivity is noteworthy, as it shows that an aprotic and modestly polar solvent improves the stereoselectivity, while the superheating facilitated with a backpressure regulator in flow mode provides for faster reaction rates with minimal decomposition.

**Table 2:** Solvent Study of the Thermal Continuous Flow Synthesis of Aziridine **12a**

Entry^a^	Solvent	^1^H NMR Yield 12a (%)^b^	*Cis:trans*^b^
1	toluene	79	88:12
2	MeCN	87	96:4
3	chlorobenzene	80	96:4
4	CPME	74	88:12
5	1,1,1-trifluoromethylbenzene	80	89:11
6	toluene:MeCN (75:25)	69	87:13
7	toluene:MeCN (50:50)	79	89:11
8	toluene:MeCN (25:75)	81	89:11

a Reaction conditions: 10 min residence time, 135 °C, 8 bar pressure, 1 M.

b Determined by ^1^H NMR of the crude reaction mixture using 1,2-bis(bromomethyl)benzene as an internal standard.

Using the optimized conditions for residence time, temperature, pressure, and solvent (Table 2, entry 2), the remaining isoxazolines underwent the thermal continuous flow rearrangement giving the corresponding aziridine products in high yields as shown in Scheme 4.

It was found that carrying out the Baldwin rearrangement under the optimal flow conditions resulted not only in higher yields, diastereoselectivities, and throughputs than the corresponding batch procedure but also a larger functional group tolerance. As with the batch yields, those bearing electron-poor aryl groups resulted in higher yields of the corresponding aziridines under flow conditions—in agreement with previous studies which have found that electron-rich aryl groups on the carbon adjacent to the nitrogen of the isoxazoline ring favor the Baldwin rearrangement due to the rate of reaction being increased but also result in faster decomposition of the resulting aziridine.^8c^ Unsurprisingly, higher yields were achieved with electron-poor aryl groups and the ^*n*^butyl group as the R substituent (**12a**–**f**), producing high yields of 67–82% in flow compared to lower and more variable yields in batch (0–69%). Aziridines **12g** and **12k** were the highest yielding aziridines of the study in both batch and flow, producing yields of 84% and 94%, respectively, in flow and 71 and 76% in batch. Despite the near quantitative yield of **12k** (94% ^1^H NMR yield), isolation was difficult, and column chromatography resulted in almost complete decomposition of the product. For compounds bearing an electron-rich aryl ring, it was required to reoptimize the residence time and reaction temperature to increase the yield and prevent extensive decomposition of the aziridine. After additional optimization experiments in flow mode, it was found that milder reaction conditions (i.e., reduced residence time and reactor temperature) of 5 min and 125 °C gave good to high isolated yields of aziridines **12h**–**i**. As expected, the more electron-rich 3,4-dimethoxyphenyl aziridine **12i** resulted in a yield (60%) lower than that of the 3,4-dimethylphenyl aziridine **12h** (82%). Importantly, the synthesis of these two aziridines was not successful using toluene in batch, with complete decomposition occurring after just 1 h of reflux. As such, milder reaction conditions were employed by refluxing the corresponding isoxazolines in MeCN for 6 h, which achieved yields of 39 and 36% of **12h** and **12i** respectively. Additionally, it was attempted to synthesize an aziridine bearing a heteroaromatic moiety. Thiophene-containing isoxazoline **11j** underwent the Baldwin rearrangement in batch and flow but resulted in complete decomposition of the isoxazoline substrate, even under the milder flow conditions (5 min, 125 °C) vs a 1 h reaction time in batch, suggesting that highly electron-rich aromatic systems on the carbon adjacent to the nitrogen atom may be a limitation of the Baldwin rearrangement, as they require more extensive optimization, as further evidenced with substrates **11h** and **11i**. Aziridines **12l**–**n** provided little success in batch, likely due to the reactivity of the cyclopropyl ring. After 1 h in batch, complete decomposition of the starting isoxazolines **11l**–**m** had occurred with no aziridine present. In addition, in the presence of the naphthalene ring, the highest yield of aziridine **12n** (41%) was achieved after 1 h, with the yield decreasing thereafter. In flow, however, the cyclopropyl ring was well-tolerated, and aziridines **12l**–**n** were all synthesized in high yields of 70–78%. Subsequently, the R group was varied to include an aromatic ring in substrates **12o**–**r**, where electronic factors seemed to affect the yield. Isoxazoline **11o** bearing a 2,4-difluorophenyl ring resulted in no conversion of the starting material in either batch nor flow. In contrast, when a 4-fluorophenyl ring was present, the corresponding aziridine **12p** was successfully synthesized in both flow and batch, albeit in significantly lower yields in batch (78 vs 40%, respectively), indicating that the fluorine atom in the *ortho* position is electronically disfavored when R is a phenyl ring. Finally, the superiority of flow synthesis over batch synthesis was further demonstrated with aziridines **12q**–**r**, in which 4-bromophenyl and naphthalene rings were employed, respectively. Product **12q** resulted in a yield of 61% in flow, with no conversion of the corresponding isoxazoline observed in batch, while **12r** was prepared in a 38% yield in flow compared to a lower yield of 19% in batch.

**Scheme 5:**
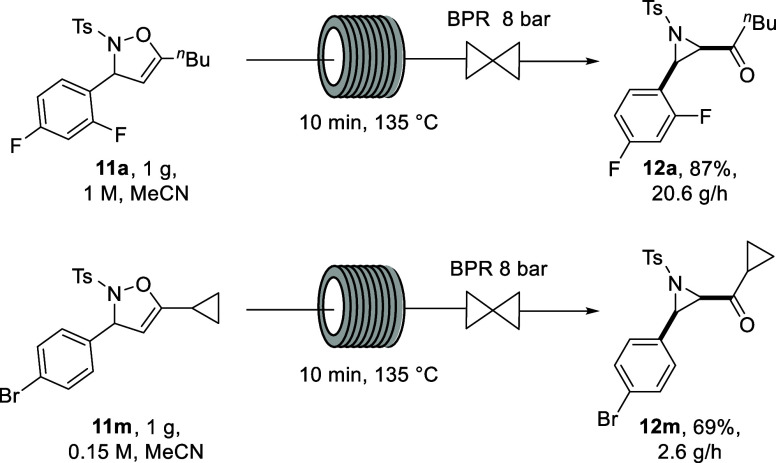
Scaled Continuous Flow Experiments Generating Aziridines **12a** and **12m**

After the various advantages of the continuous flow synthesis of aziridines via the Baldwin rearrangement over the batch procedure were demonstrated, it was desirable to demonstrate the robustness and scalability of the flow procedure. As such, two 1 g scale experiments were performed, synthesizing aziridines **12a** and **12m** (Scheme 5).

Both gram-scale syntheses compared well with the smaller 300 mg scale syntheses performed initially and demonstrated the high throughput of the continuous flow reaction. The yield of aziridine **12a** was slightly higher in the scale-up experiment, compared to the smaller scale experiment (87 vs 82%). The scale-up of isoxazoline **11a** resulted in a high throughput of 20.6 g/h (52 mmol/h) if the system was run continuously for 1 h. Substrate **11m** was chosen for the second scale-up experiment, and the resulting yield of 69% compared well with the original yield of **12m** of 70%. This resulted in a throughput of 2.6 g/h (6 mmol/h), with a lower throughput compared to **12a** due to the limited solubility of isoxazoline **11m** in MeCN. Both scaled reactions demonstrate the robustness of the method and the facile scalability associated with continuous flow technology.

## Conclusions

In conclusion, we have exploited the advantages of continuous flow technology and developed a continuous flow synthesis of aziridines via the underutilized Baldwin rearrangement, yielding a library of aziridines in higher yields, diastereoselectivities, and throughputs than the corresponding batch procedure, with a larger functional group tolerance, in a 5–10 min residence time. The chosen solvent (i.e., MeCN) hereby played a crucial role, as it allowed for consistently high diastereoselectivities, and the ability to superheat the reaction mixture (ca. 50 °C above the atmospheric boiling point) accounts for faster reaction rates, higher yields, and minimized product decomposition that characterize this flow process. Additionally, the robustness and scalability of the continuous flow method have been demonstrated by carrying out two gram-scale syntheses. These results not only showcase a robust entry to diverse aziridines but moreover demonstrate the power of continuous flow technology in streamlining the accessibility of valuable heterocyclic entities and overcoming issues related to initial batch processes.

## Experimental Section

### Procedure for the Synthesis of *N*-Hydroxylsulfonamides, **10a**–**10b**

Prepared according to modified literature procedure.^18^ Hydroxylamine hydrochloride (557 mg, 8 mmol, 2 equiv) was dissolved in water (8 mL, 1 M) at 0 °C. A solution of Na_2_CO_3_ (848 mg, 8 mmol, 2 equiv) in water (4 mL, 2 M) was added dropwise to the hydroxylamine solution at an internal reaction temperature of 5–15 °C and stirred for 15 min. THF (4 mL) and methanol (1 mL) were added, followed by the addition of *p*-toluenesulfonyl chloride (763 mg, 4 mmol, 1 equiv) in portions at an internal reaction temperature of 5–15 °C. After complete addition, the reaction was stirred at rt for 4 h. The mixture was extracted with EtO_2_ (2 × 20 mL), and the combined organic layers were washed with brine and dried over Na_2_SO_4_ and filtered. Solvent was evaporated **in vacuo**, and the resulting residue was used without further purification.

### Procedure for the Synthesis of *N*-Hydroxylsulfonamide, **10c**

Prepared according to a modified literature procedure.^16^ EtOAc (10 mL) was mixed with a solution of NaHCO_3_ (2.0 g, 24 mmol, 2.4 equiv) in water (5 mL, 4.8 M). Hydroxylamine hydrochloride (834 mg, 12 mmol, 1.2 equiv) was added, and the reaction mixture was stirred until dissolved at r.t.. A solution of 4-trifluoromethyl benzoyl chloride (2.1 g, 10 mmol, 1 equiv) in ethyl acetate (5 mL, 2 M) was added dropwise. The mixture was stirred at r.t. for 15 min. EtOAc was removed *in vacuo*, and the resulting residue was filtered under vacuum and used without further purification.

### General Procedure **1** for the Synthesis of Propargyl Alcohols, **9a**–**9v**

Prepared according to a modified literature procedure.^19^ LiHMDS (5.5 mL, 1 M in THF, 5.5 mmol, 1.38 equiv) was added to a clean, dry flask, followed by toluene (5 mL, 1.1 M) at −78 °C under N_2_ atmosphere. The alkyne solution (5 mmol, 1.25 equiv) was added dropwise to this solution. The reaction was stirred at −78 °C for 20 min and then at r.t. for 1 h. After cooling to −78 °C, the aldehyde solution (4 mmol, 1 equiv) was added dropwise, and the reaction was stirred at −78 °C for 20 min and then at rt for 1 h, before being quenched with aqueous NH_4_Cl (20 mL). The mixture was extracted with EtOAc (2 × 20 mL), and the combined organic layers were washed with brine, dried over Na_2_SO_4_, and filtered. Solvent was evaporated *in vacuo*, and the resulting residue was purified by flash chromatography (EtOAc/cyclohexane).

### General Procedure **2** for the Synthesis of Isoxazolines, **11a**–**11r**

Prepared according to a modified literature procedure.^8c^ To a stirred solution of propargyl alcohol (1.2 mmol) in DCM (5 mL, 0.24 M) were added FeCl_3_·6H_2_O (2.5 mol %) and hydroxylamine (187 mg, 1 mmol), and the solution was refluxed for 30 min. Pyridine (10 mol %) was then added to the reaction mixture, followed by NaAuCl_4_·2H_2_O (5 mol %), and the reflux was maintained for 2 h. The reaction mixture was filtered, solvent was evaporated *in vacuo*, and the resulting residue was purified by flash chromatography (EtOAc/cyclohexane).

### General Procedure **3** for the Synthesis of Aziridines **12a**–**12r** (Batch)

A solution of isoxazoline in toluene (0.125 M) was refluxed for 1–3 h. Solvent was evaporated *in vacuo*, and the resulting residue was purified by flash chromatography (EtOAc/cyclohexane).

### General Procedure **4** for the Synthesis of Aziridines **12a**–**12r** (Flow)

The flow system was flushed with MeCN prior to adding reagents. A solution of isoxazoline in MeCN (0.125, 0.15, or 1 M) was injected at the appropriate flow rate (1 mL/min, 10 min residence time or 2 mL/min, 5 min residence time) under 8 bar pressure. The resulting reaction stream was collected, the solvent was evaporated *in vacuo*, and the resulting residue was purified by flash chromatography (EtOAc/cyclohexane).

### Spectroscopic Data of Aziridine Products **12a**–**12r**

#### 1-((2*R*,3*R*)-3-(2,4-Difluorophenyl)-1-tosylaziridin-2-yl)pentan-1-one, **12a**

Yield: 82% (123 mg, 0.31 mmol). Appearance: Pale yellow oil. HR-MS (QTOF) *m*/*z*: [M + H]^+^ Calcd for C_20_H_21_F_2_NO_3_SH^+^ 394.1283; found 394.1285. ^1^H NMR (400 MHz, CDCl_3_) δ 7.91 (d, *J* = 8.2 Hz, 2H), 7.38 (d, *J* = 7.9 Hz, 2H), 7.27–7.21 (m, 1H), 6.81–6.73 (m, 2H), 4.17 (d, *J* = 7.9 Hz, 1H), 3.74 (d, *J* = 7.9, 1H), 2.46 (s, 3H), 2.30–2.11 (m, 2H), 1.35–1.24 (m, 2H), 1.03 (sext, *J* = 7.4 Hz, 2H), 0.73 (t, *J* = 7.4 Hz, 3H). ^13^C{^1^H} NMR (101 MHz, CDCl_3_) δ 200.8 (C), 163.0 (dd, *J* = 251, 12 Hz, CF), 161.2 (dd, *J* = 251, 12 Hz, CF), 145.5 (C), 133.7 (C), 130.5 (dd, *J* = 10, 5 Hz, CH), 130.0 (2CH), 128.2 (2CH), 115.0 (dd, *J* = 14, 4 Hz, C), 111.4 (dd, *J* = 10, 5 Hz, CH), 103.8 (dd, *J* = 26, 24 Hz, CH), 47.8 (CH), 41.4 (CH_2_), 40.9 (d, *J* = 4 Hz, CH), 24.8 (CH_2_), 21.9 (CH_2_), 21.7 (CH_3_), 13.5 (CH_3_). ^19^F NMR (376 MHz, CDCl_3_) δ −113.4 (m), −108.5 (m). IR (neat) v/cm^–1^: 2958 (w), 2929 (w), 2872 (w), 1718 (m), 1598 (m), 1507 (s), 1334 (s), 1162 (s), 1091 (s), 816 (m).

#### 1-((2*R*,3*R*)-3-(4-Fluorophenyl)-1-tosylaziridin-2-yl)pentan-1-one, **12b**

Yield: 68% (204 mg, 0.54 mmol). Appearance: Pale yellow solid. Melting range: 77–78 °C. HR-MS (QTOF) *m*/*z*: [M + H]^+^ Calcd for C_20_H_22_FNO_3_SH^+^ 376.1377; found 376.1378. ^1^H NMR (500 MHz, CDCl_3_) δ 7.91 (d, *J* = 8.3 Hz, 2H), 7.38 (d, *J* = 7.8 Hz, 2H), 7.22–7.19 (m, 2H), 6.98–6.94 (m, 2H), 4.11 (d, *J* = 7.8 Hz, 1H), 3.62 (d, *J* = 7.8 Hz, 1H), 2.46 (s, 4H), 2.17–2.10 (m, 1H), 1.99–1.93 (m, 1H), 1.26–1.15 (m, 2H), 1.00–0.93 (m, 2H), 0.68 (t, *J* = 7.3 Hz, 3H). ^13^C{^1^H} NMR (126 MHz, CDCl_3_) δ 202.4 (C), 162.9 (d, *J* = 248 Hz, CF), 145.6 (C), 134.0 (C), 130.2 (2CH), 129.4 (d, *J* = 9 Hz, 2CH), 128.3 (2CH), 127.4 (d, *J* = 4 Hz, C), 115.8 (d, *J* = 22 Hz, 2CH), 48.9 (CH), 45.2 (CH), 41.0 (CH_2_), 24.8 (CH_2_), 22.0 (CH_2_), 21.9 (CH_3_), 13.7 (CH_3_). ^19^F NMR (376 MHz, CDCl_3_) δ −112.7 (m). IR (neat): v/cm^–1^: 2960 (m), 2931 (m), 2874 (m), 1725 (m), 1596 (m), 1511 (s), 1337 (s), 1156 (s), 1086 (m), 820 (s).

#### 1-((2*R*,3*R*)-3-(4-Bromophenyl)-1-tosylaziridin-2-yl)pentan-1-one, **12c**

Yield: 80% (240 mg, 0.55 mmol). Appearance: Pale yellow oil. HR-MS (QTOF) *m*/*z*: [M + H]^+^ Calcd for C_20_H_22_BrNO_3_SH+ 436.0577; found 436.0578. ^1^H NMR (400 MHz, CDCl_3_) δ 7.90 (d, *J* = 8.3 Hz, 2H) 7.40–7.36 (m, 4H), 7.10 (d, *J* = 8.3, 2H), 4.07 (d, *J* = 7.9 Hz, 1H), 3.65 (d, *J* = 7.9 Hz, 1H), 2.45 (s, 3H), 2.14 (ddd, *J* = 17.4, 8.2, 6.3 Hz, 1H), 1.98 (ddd, *J* = 17.4, 8.2, 6.3 Hz, 1H), 1.30–1.15 (m, 2H), 1.03–0.92 (m, 2H), 0.68 (t, *J* = 7.4 Hz, 3H). ^13^C{^1^H} NMR (101 MHz, CDCl_3_) δ 201.9 (C), 145.5 (C), 133.8 (C), 131.7 (2CH), 130.4 (C), 130.0 (2CH), 129.1 (2CH), 128.2 (2CH), 122.8 (C), 48.7 (CH), 45.1 (CH), 40.9 (CH_2_), 24.7 (CH_2_), 21.8 (CH_2_), 21.7 (CH_3_), 13.5 (CH_3_). IR (neat): v/cm^–1^: 2957 (w), 2930 (w), 2871 (w), 1713 (m), 1596 (m), 1490 (m), 1455 (m), 1331 (s), 1091 (m), 678 (m).

#### 1-((2*R*,3*R*)-3-(2-Chlorophenyl)-1-tosylaziridin-2-yl)pentan-1-one, **12d**

Yield: 81% (243 mg, 0.62 mmol). Appearance: Pale yellow oil. HR-MS (QTOF) *m*/*z*: [M + H]^+^ Calcd for C_20_H_22_ClNO_3_SH^+^ 392.1082; found 392.1086. ^1^H NMR (400 MHz, CDCl_3_) δ 7.91 (d, *J* = 8.3 Hz, 2H), 7.37 (d, *J* = 8.0 Hz, 2H), 7.30 (ddd, *J* = 9.5, 7.6, 1.6 Hz, 2H), 7.21–7.16 (m, 2H), 4.23 (d, *J* = 7.9 Hz, 1H), 3.78 (d, *J* = 7.9 Hz, 1H), 2.45 (s, 3H), 2.28–2.20 (m, 1H), 2.09–2.01 (m, 1H), 1.29–1.19 (m, 2H), 0.99–0.87 (m, 2H), 0.68 (t, *J* = 7.3 Hz, 3H). ^13^C{^1^H} NMR (101 MHz, CDCl_3_) δ 201.0 (C), 145.5 (C), 134.0 (C), 133.6 (C), 130.1 (2CH), 129.9 (CH), 129.7 (CH), 129.6 (CH), 129.3 (CH), 128.3 (2CH), 126.9 (C), 48.2 (CH), 44.8 (CH), 40.9 (CH_2_), 24.9 (CH_2_), 21.9 (CH_2_), 21.8 (CH_3_), 13.7 (CH_3_). IR (neat): v/cm^–1^: 2957 (w), 2930 (w), 2871 (w), 1714 (m), 1596 (m), 1494 (m), 1333 (s), 1162 (s), 1091 (m), 759 (m).

#### 1-((2*R*,3*R*)-3-(3-Chloro-4-fluorophenyl)-1-tosylaziridin-2-yl)pentan-1-one, **12e**

Yield: 82% (246 mg, 0.60 mmol). Appearance: White solid. HR-MS (QTOF) *m*/*z*: [M + Na]^+^ Calcd for C_20_H_21_ClFNO_3_SNa^+^ 432.0807; found 432.0807. ^1^H NMR (500 MHz, CDCl_3_) δ 7.91 (d, *J* = 8.3 Hz, 2H), 7.39 (d, *J* = 8.3 Hz, 2H), 7.28 (dd, *J* = 6.8, 2.0 Hz, 1H), 7.13–7.10 (m, 1H), 7.04 (t, *J* = 8.6 Hz, 1H), 4.06 (d, *J* = 7.8 Hz, 1H), 3.63 (d, *J* = 7.8 Hz, 1H), 2.46 (s, 3H), 2.19–2.13 (m, 1H), 2.06–2.00 (m, 1H), 1.30–1.20 (m, 2H), 0.99 (h, *J* = 7.1 Hz, 2H), 0.70 (t, *J* = 7.3 Hz, 3H). ^13^C{^1^H} NMR (126 MHz, CDCl_3_) δ 201.7 (C), 158.2 (d, *J* = 251 Hz, CF), 145.7 (C), 133.8 (C), 130.2 (2CH), 130.0 (CH), 128.7 (d, *J* = 5 Hz, C), 128.3 (2CH), 127.5 (d, *J* = 8 Hz, CH), 121.5 (d, *J* = 18 Hz, C), 116.9 (d, *J* = 22 Hz, CH), 48.9 (CH), 44.5 (CH), 41.2 (CH_2_), 24.8 (CH_2_), 22.0 (CH_2_), 21.9 (CH_3_), 13.6 (CH_3_). ^19^F NMR (376 MHz, CDCl_3_) δ/ppm −114.7 (m). IR (neat) v/cm^–1^: 2958 (m), 2930 (m), 2872 (w), 1719 (m), 1596 (m), 1502 (m), 1331 (m), 1162 (s), 1087 (m), 673 (s).

#### 1-((2*R*,3*R*)-1-Tosyl-3-(4-(trifluoromethyl)phenyl)aziridin-2-yl)pentan-1-one, **12f**

Yield: 67% (201 mg, 0.47 mmol). Appearance: Pale yellow oil. HR-MS (QTOF) *m*/*z*: [M + Na]^+^ Calcd for C_21_H_22_F_3_NO_3_SNa^+^ 448.1165; found 448.1163. ^1^H NMR (400 MHz, CDCl_3_) δ 7.92 (d, *J* = 8.3 Hz, 2H), 7.54 (d, *J* = 8.2 Hz, 2H), 7.39 (d, *J* = 8.0 Hz, 2H), 7.36 (d, *J* = 8.7 Hz, 2H), 4.16 (d, *J* = 7.9 Hz, 1H), 3.70 (d, *J* = 7.9 Hz, 1H), 2.47 (s, 3H), 2.15 (ddd, *J* = 17.5, 8.0, 6.4 Hz, 1H), 2.00 (ddd, *J* = 17.4, 7.9, 6.6 Hz, 1H), 1.29–1.15 (m, 2H), 0.95 (s, *J* = 7.2 Hz, 2H), 0.66 (t, *J* = 7.3 Hz, 3H). ^13^C{^1^H} NMR (101 MHz, CDCl_3_) δ 201.8 (C), 145.8 (C), 135.6 (C), 133.9 (C), 131.0 (q, *J* = 33 Hz, C), 130.2 (2CH), 128.4 (2CH), 128.1 (2CH), 126.6 (q, *J* = 272 Hz, CF_3_), 125.7 (q, *J* = 4 Hz, 2CH), 48.8 (CH), 45.2 (CH), 41.1 (CH_2_), 24.8 (CH_2_), 21.9 (CH_2_), 21.9 (CH_3_), 13.6 (CH_3_). ^19^F NMR (376 MHz, CDCl_3_) δ −62.8 (s). IR (neat) v/cm^–1^: 2962 (w), 2935 (w), 2877 (w), 1726 (m), 1595 (w), 1397 (m), 1322 (s), 1154 (s), 1067 (s), 808 (m).

#### 1-((2*R*,3*R*)-3-Phenyl-1-tosylaziridin-2-yl)pentan-1-one, **12g**

Yield: 84% (252 mg, 0.71 mmol). Appearance: White solid. Melting range: 88–89 °C. HR-MS (QTOF) *m*/*z*: [M + H]^+^ Calcd for C_20_H_23_NO_3_SH^+^ 358.1471; found 358.1471. ^1^H NMR (500 MHz, CDCl_3_) δ 7.92 (d, *J* = 8.3 Hz, 2H), 7.38 (d, *J* = 7.8 Hz, 2H), 7.27–7.25 (m, 3H), 7.22–7.20 (m, 2H), 4.14 (d, *J* = 7.8 Hz, 1H), 3.63 (d, *J* = 7.8 Hz, 1H), 2.46 (s, 3H), 2.13 (ddd, *J* = 17.6, 8.1, 6.4 Hz, 1H), 1.92 (ddd, *J* = 17.6, 8.1, 6.6 Hz, 1H), 1.27–1.11 (m, 2H), 0.98–0.87 (m, 2H), 0.65 (t, *J* = 7.3 Hz, 3H). ^13^C{^1^H} NMR (126 MHz, CDCl_3_) δ 202.8 (C), 145.5 (C), 134.1 (C), 131.5 (C), 130.1 (2CH), 128.7 (CH), 128.7 (2CH), 128.4 (2CH), 127.6 (2CH), 48.9 (CH), 45.9 (CH), 40.8 (CH_2_), 24.8 (CH_2_), 21.9 (CH_2_), 21.9 (CH_3_), 13.7 (CH_3_). IR (neat): v/cm^–1^: 2957 (m), 2928 (m), 2872 (m), 1724 (m), 1595 (m), 1497 (m), 1339 (s), 1154 (s), 1086 (m), 822 (m).

#### 1-((2*R*,3*R*)-3-(3,4-Dimethylphenyl)-1-tosylaziridin-2-yl)pentan-1-one, **12h**

Yield: 82% (246 mg, 0.64 mmol). Appearance: Pale yellow oil. HR-MS (QTOF) *m*/*z*: [M + Na]^+^ Calcd for C_22_H_27_NO_3_SNa^+^ 408.1604; Found 408.1606. ^1^H NMR (600 MHz, CDCl_3_) δ 7.92 (d, *J* = 8.2 Hz, 2H), 7.38 (d, *J* = 8.2 Hz, 2H), 7.01 (d, *J* = 7.6 Hz, 1H), 6.95 (s, 1H), 6.91 (d, *J* = 7.6 Hz, 1H), 4.07 (d, *J* = 8.2 Hz, 1H), 3.58 (d, *J* = 8.2 Hz, 1H), 2.46 (s, 3H), 2.19 (s, 3H), 2.17 (s, 3H), 2.15–2.11 (m, 1H), 1.95 (ddd, *J* = 17.6, 8.2, 6.5 Hz, 1H), 1.27–1.15 (m, 2H), 1.00–0.93 (m, 2H), 0.66 (t, *J* = 7.3 Hz, 3H). ^13^C{^1^H} NMR (151 MHz, CDCl_3_) δ 203.1 (C), 145.4 (C), 137.2 (C), 137.0 (C), 134.2 (C), 130.1 (2CH), 129.8 (CH), 128.8 (C), 128.7 (CH), 128.4 (2CH), 124.9 (CH), 49.0 (CH), 45.9 (CH), 40.8 (CH_2_), 24.8 (CH_2_), 22.0 (CH_2_), 21.9 (CH_3_), 19.8 (CH_3_), 19.6 (CH_3_), 13.7 (CH_3_). IR (neat): v/cm^–1^: 2957 (w), 2928 (w), 2870 (w), 1720 (m), 1598 (m), 1497 (w), 1334 (m), 1157 (s), 1090 (m), 813 (m).

#### 1-((2*R*,3*R*)-3-(3,4-Dimethoxyphenyl)-1-tosylaziridin-2-yl)pentan-1-one, **12i**

Yield: 60% (251 mg, 0.61 mmol). Appearance: Yellow oil. HR-MS (QTOF) *m*/*z*: [M + H]^+^ Calcd for C_22_H_27_NO_5_SH^+^ 418.1683; found 418.1683. ^1^H NMR (500 MHz, CDCl_3_) δ 7.93 (d, *J* = 8.3 Hz, 2H), 7.38 (d, *J* = 8.3 Hz, 2H), 6.78–6.73 (m, 2H), 6.65 (d, *J* = 2.0 Hz, 1H), 4.07 (d, *J* = 7.8 Hz, 1H), 3.83 (s, 3H), 3.77 (s, 3H), 3.61 (d, *J* = 7.8 Hz, 1H), 2.46 (s, 3H), 2.17 (dd, *J* = 17.6 8.1, 6.1 Hz, 1H), 2.00–1.93 (m, 1H), 1.27–1.20 (m, 2H), 1.03–0.96 (m, 2H), 0.68 (t, *J* = 7.3 Hz, 3H). ^13^C{^1^H} NMR (126 MHz, CDCl_3_) δ 203.0 (C), 149.3 (C), 149.0 (C), 145.4 (C), 134.2 (C), 130.1 (2CH), 128.4 (2CH), 123.9 (C), 120.1 (CH), 111.1 (CH), 110.4 (CH), 56.0 (CH_3_), 56.0 (CH_3_), 49.0 (CH), 45.8 (CH), 40.9 (CH_2_), 24.8 (CH_2_), 22.0 (CH_2_), 21.9 (CH_3_), 13.7 (CH_3_). IR (neat) v/cm^–1^: 2956 (m), 2932 (m), 2871 (m), 1715 (m), 1595 (m), 1513 (s), 1337 (m), 1161 (s), 1089 (m), 813 (m).

#### 1-((2*R*,3*R*)-3-(Naphthalen-2-yl)-1-tosylaziridin-2-yl)pentan-1-one, **12k**

Yield: 94% (282 mg, 0.74 mmol). Appearance: Pale yellow oil. HR-MS (QTOF) *m*/*z*: [M + H]^+^ Calcd for C_24_H_25_NO_3_SH^+^ 408.1628; found 408.1628. ^1^H NMR (500 MHz, CDCl_3_) δ 7.96 (d, *J* = 8.3 Hz, 2H), 7.79–7.74 (m, 3H), 7.70 (s, 1H), 7.48–7.46 (m, 2H), 7.40 (d, *J* = 8.3 Hz, 2H), 7.31 (dd, *J* = 8.6, 1.7 Hz, 1H), 4.29 (d, *J* = 8.3 Hz, 1H), 3.70 (d, *J* = 7.9 Hz, 1H), 2.47 (s, 3H), 2.16–2.11 (m, 1H), 1.97–1.91 (m, 1H), 1.22–1.10 (m, 2H), 0.90–0.85 (m, 2H), 0.56 (t, *J* = 7.3 Hz, 3H). IR (neat): v/cm^–1^: 3054 (w), 3009 (w), 2863 (w), 1617 (m), 1508 (m), 1358 (m), 1284 (m), 1152 (m), 1121 (m), 818 (s). Compound was found to be unstable toward silica gel chromatography.

#### ((2*R*,3*R*)-3-(2,4-Difluorophenyl)-1-tosylaziridin-2-yl)(cyclopropyl)methanone, **12l**

Yield: 70% (210 mg, 0.56 mmol). Appearance: White solid. Melting range: 97–99 °C. HR-MS (QTOF) *m*/*z*: [M + H]^+^ Calcd for C_19_H_17_F_2_NO_3_SH^+^ 378.0970; found 378.0971. ^1^H NMR (500 MHz, CDCl_3_) δ 7.92 (d, *J* = 8.3 Hz, 2H), 7.38 (d, *J* = 7.8 Hz, 2H), 7.23 (td, *J* = 8.6, 6.1 Hz, 1H), 6.78–6.74 (m, 2H), 4.17 (d, *J* = 7.3 Hz, 1H), 3.83 (d, *J* = 7.8 Hz, 1H), 2.46 (s, 3H), 1.97–1.92 (m, 1H), 0.84–0.74 (m, 2H), 0.70–0.65 (m, 1H), 0.63–0.58 (m, 1H). ^13^C{^1^H} NMR (126 MHz, CDCl_3_) δ 201.0 (C), 163.2 (dd, *J* = 252, 12 Hz, CF), 161.2 (dd, *J* = 252, 13 Hz, CF), 145.6 (C), 133.8 (C), 130.5 (dd, *J* = 10, 5 Hz, CH), 130.2 (2CH), 128.4 (2CH), 115.5 (dd, *J* = 14, 4 Hz, C), 111.4 (dd, *J* = 21, 4 Hz, CH), 103.9 (dd, *J* = 25, 24 Hz, CH), 48.9 (CH), 40.9 (d, *J* = 4 Hz, CH), 21.9 (CH_3_), 19.3 (CH), 12.2 (CH_2_), 12.2 (CH_2_). ^19^F NMR (376 MHz, CDCl_3_) δ/ppm −108.7 (m), −113.3 (m). IR (neat): v/cm^–1^: 3080 (w), 3071 (w), 3011 (w), 1694 (m), 1598 (m), 1507 (m), 1335 (s), 1163 (s), 1092 (s), 816 (m).

#### ((2*R*,3*R*)-3-(4-Bromophenyl)-1-tosylaziridin-2-yl)(cyclopropyl)methanone, **12m**

Yield: 70% (210 mg, 0.50 mmol). Appearance: White solid. Melting range: 134–136 °C. HR-MS (QTOF) *m*/*z*: [M + Na]^+^ Calcd for C_19_H_18_BrNO_3_SNa^+^ 444.0064; found 444.0063. ^1^H NMR (500 MHz, CDCl_3_) δ 7.92 (d, *J* = 8.3 Hz, 2H), 7.40–7.37 (m, 4H), 7.10 (d, *J* = 8.3 Hz, 2H), 4.07 (d, *J* = 7.8 Hz, 1H), 3.75 (d, *J* = 7.8 Hz, 1H), 2.46 (s, 3H), 1.93 (tt, *J* = 7.8, 4.6 Hz, 1H), 0.88–0.83 (m, 1H), 0.77–0.72 (m, 1H), 0.60–0.51 (m, 2H). ^13^C{^1^H} NMR (126 MHz, CDCl_3_) δ 201.9 (C), 145.6 (C), 133.9 (C), 131.7 (2CH), 130.9 (C), 130.2 (2CH), 129.3 (2CH), 128.3 (2CH), 122.7 (C), 49.7 (CH), 45.4 (CH), 21.9 (CH_3_), 19.3 (CH), 12.6 (CH_2_), 12.4 (CH_2_). IR (neat): v/cm^–1^: 2993 (w), 2922 (w), 2852 (w), 1703 (m), 1595 (m), 1489 (m), 1337 (s), 1154 (s), 1081 (s), 660 (m).

#### ((2*R*,3*R*)-3-(Naphthalen-2-yl)-1-tosylaziridin-2-yl)(cyclopropyl)methanone, **12n**

Yield: 78% (234 mg, 0.60 mmol). Appearance: White solid. HR-MS (QTOF) *m*/*z*: [M + Na]^+^ Calcd for C_23_H_21_NO_3_SNa^+^ 414.1134; found 414.1140. ^1^H NMR (500 MHz, CDCl_3_) δ 7.97 (d, *J* = 8.3 Hz, 2H), 7.80–0.76 (m, 1H), 7.74–7.73 (m, 2H), 7.71 (s, 1H), 7.48–7.44 (m, 2H), 7.39 (d, *J* = 8.3 Hz, 2H), 7.31 (dd, *J* = 8.6, 1.7 Hz, 1H), 4.29 (d, *J* = 7.8 Hz, 1H), 3.81 (d, *J* = 8.3 Hz, 1H), 2.46 (s, 3H), 2.01–1.96 (m, 1H), 0.83–0.78 (m, 1H), 0.67 (qd, *J* = 7.3, 3.4 Hz, 1H), 0.46–0.41 (m, 1H), 0.34 (qd, *J* = 8.3, 3.9 Hz, 1H). ^13^C{^1^H} NMR (126 MHz, CDCl_3_) δ 202.5 (C), 145.5 (C), 134.1 (C), 133.2 (C), 133.0 (C), 130.2 (2CH), 129.2 (C), 128.4 (2CH), 128.4 (CH), 128.0 (CH), 127.9 (CH), 127.1 (CH), 126.6 (CH), 126.6 (CH), 124.8 (CH), 49.9 (CH), 46.2 (CH), 21.9 (CH_3_), 19.3 (CH), 12.6 (CH_2_), 12.3 (CH_2_). IR (neat) v/cm^–1^: 2954 (w), 2924 (w), 2855 (w), 1703 (m), 1595 (m), 1509 (m), 1341 (s), 1154 (s), 1081 (s), 820 (s).

#### ((2*R*,3*R*)-3-(4-Fluorophenyl)-1-tosylaziridin-2-yl)(phenyl)methanone, **12p**

Yield: 78% (234 mg, 0.59 mmol). Appearance: Pale yellow solid. Melting range: 138–140 °C. HR-MS (QTOF) *m*/*z*: [M + Na]^+^ Calcd for C_22_H_18_FNO_3_SNa^+^ 418.0884; Found 418.0882. ^1^H NMR (400 MHz, CDCl_3_) δ 7.97 (d, *J* = 8.2 Hz, 2H), 7.86–7.84 (m, 2H), 7.55 (t, *J* = 7.4 Hz, 1H), 7.42–7.37 (m, 3H), 7.35 (s, 1H), 7.24–7.19 (m, 2H), 6.89–6.83 (m, 2H), 4.40 (d, *J* = 7.4 Hz, 1H), 4.33 (d, *J* = 7.8 Hz, 1H), 2.44 (s, 3H). ^13^C{^1^H} NMR (101 MHz, CDCl_3_) δ 188.9 (C), 162.9 (d, *J* = 248 Hz, CF), 145.4 (C), 135.7 (C), 134.4 (C), 134.1 (CH), 130.1 (CH), 129.3 (d, *J* = 8 Hz, 2CH), 128.9 (3CH), 128.5 (2CH), 128.2 (2CH), 127.1 (d, *J* = 3 Hz, C), 115.6 (d, *J* = 22 Hz, 2CH), 48.3 (CH), 45.9 (CH), 21.9 (CH_3_). ^19^F NMR (376 MHz, CDCl_3_) δ −112.9 (m). IR (neat): v/cm^–1^: 2955 (w), 2924 (m), 2854 (w), 1691 (m), 1597 (m), 1511 (m), 1327 (m), 1160 (s), 1091 (m), 814 (m).

This data is consistent with published work.^20^

#### ((2*R*,3*R*)-3-(4-Bromophenyl)-1-tosylaziridin-2-yl)(phenyl)methanone, **12q**

Yield: 61% (183 mg, 0.40 mmol). Appearance: Pale yellow oil. HR-MS (QTOF) *m*/*z*: [M + Na]^+^ Calcd for C_22_H_18_BrNO_3_SNa^+^ 480.0064; found 480.0059. ^1^H NMR (500 MHz, CDCl_3_) δ 7.96 (d, *J* = 8.3 Hz, 2H), 7.85 (dd, *J* = 8.1, 1.2 Hz, 2H), 7.57–7.53 (m, 1H), 7.43–7.39 (m, 2H), 7.36 (d, *J* = 7.8 Hz, 2H), 7.31–7.29 (m, 2H), 7.11 (d, *J* = 8.3 Hz, 2H), 4.42 (d, *J* = 7.8 Hz, 1H), 4.29 (d, *J* = 7.8 Hz, 1H), 2.44 (s, 3H). ^13^C{^1^H} NMR (126 MHz, CDCl_3_) δ 188.8 (C), 145.5 (C), 135.6 (C), 134.3 (C), 134.2 (CH), 131.7 (2CH), 130.4 (C), 130.1 (2CH), 129.2 (2CH), 128.9 (2CH), 128.5 (2CH), 128.2 (2CH), 122.9 (C), 48.2 (CH), 45.9 (CH), 21.9 (CH_3_). IR (neat): v/cm^–1^: 2988 (w), 2923 (w), 1690 (m), 1596 (m), 1489 (m), 1327 (m), 1157 (s), 1089 (m), 813 (m), 676 (s).

This data is consistent with published work.^21^

#### ((2*R*,3*R*)-3-(Naphthalen-2-yl)-1-tosylaziridin-2-yl)(phenyl)methanone, **12r**

Yield: 38% (114 mg, 0.27 mmol). Appearance: White solid. HR-MS (QTOF) *m*/*z*: [M + Na]^+^ Calcd for C_26_H_21_NO_3_SNa^+^ 450.1134; Found 450.1138. ^1^H NMR (500 MHz, CDCl_3_) δ 8.01 (d, *J* = 8.3 Hz, 2H), 7.86 (dd, *J* = 8.6, 1.2 Hz, 2H), 7.73 (s, 1H), 7.72–7.68 (m, 2H), 7.64 (d, *J* = 8.8 Hz, 1H), 7.52–7.49 (m, 1H), 7.42–7.35 (m, 6H), 7.31 (dd, *J* = 8.6, 1.7 Hz, 1H), 4.51 (d, *J* = 7.8 Hz, 1H), 4.48 (d, *J* = 7.8 Hz, 1H), 2.43 (s, 3H). ^13^C{^1^H} NMR (126 MHz, CDCl_3_) δ: 189.1 (C), 145.3 (C), 135.8 (C), 134.5 (C), 134.0 (CH), 133.3 (C), 132.9 (C), 130.1 (2CH), 128.8 (2CH), 128.5 (2CH), 128.3 (CH), 128.3 (2CH), 128.2 (C), 127.7 (2CH), 127.2 (CH), 126.5 (CH), 126.4 (CH), 124.6 (CH), 48.6 (CH), 46.9 (CH), 21.9 (CH_3_). IR (neat) v/cm^–1^: 2954 (m), 2923 (m), 2854 (m), 1689 (m), 1596 (m), 1509 (m), 1328 (s), 1158 (s), 1089 (s), 814 (m).

## Supporting Information Available

Materials and methods, synthetic procedures, unsuccessful substrates, and ^1^H NMR, ^13^C NMR, HRMS, IR, and melting point (where applicable) data of all synthesized compounds (PDF)
