# Supplementary material for: Development of a Continuous Flow Baldwin Rearrangement Process and Its Comparison to Traditional Batch Mode
Source: Org Process Res Dev. 2023 Sep 11;28(5):1567–75. doi: 10.1021/acs.oprd.3c00213 (PMC11110046; doi:10.1021/acs.oprd.3c00213)
Supplement: Supplementary file 1 — op3c00213_si_001.pdf [file op3c00213_si_001.pdf]

SUPPORTING INFORMATION

Development of a Continuous Flow Baldwin Rearrangement Process and its Comparison to Traditional Batch Mode

Arlene Bonner[a], Marcus Baumann[a]*

[a]School of Chemistry, University College Dublin, Science Centre South, Belfield, Dublin 4, Ireland, D04 N2E2.

*Email: marcus.baumann@ucd.ie

Table of Contents

1. Materials and Methods **SI-2**

2. Synthetic Procedures **SI-3**

3. Unsuccessful substrates **SI-5**

4. Spectroscopic Data

4.1 Propargyl Alcohols (**9a-9v**) **SI-6**

4.2 Hydroxylamines (**10a-10c**) **SI-15**

4.3 Isoxazolines (**11a-11r**) **SI-16**

4.4 Aziridines (**12a-12r**) **SI-25**

5. Copies of ^{1}H and ^{13}C NMR Spectra **SI-34**

6. References **SI-104**

1. Materials and Methods

Unless otherwise stated, all solvents were purchased from Fisher Scientific, Sigma, and Honeywell and used without further purification. Substrates and reagents were purchased from Fluorochem, Alpha Aesar or Sigma and used as received.

^{1}H-NMR spectra were recorded on 400 MHz, 500 MHz and 600 MHz instruments and are reported relative to residual solvent: $CDCl_3$ (δ 7.26 ppm) and DMSO-d_6 (δ 39.52 ppm). ^{13}C-NMR spectra were recorded on the same instruments (100, 125 and 150 MHz) and are reported relative to $CDCl_3$ (δ 77.16 ppm). ^{19}F-NMR spectra were recorded on 400 MHz (376 MHz) spectrometer.

Data for ^{1}H-NMR are reported as follows: chemical shift (δ/ ppm) (multiplicity, coupling constant (Hz), integration). Multiplicities are reported as follows: s = singlet, d = doublet, t = triplet, q = quartet, sext = sextet, sept = septet, m = multiplet. Data for ^{13}C-NMR are reported in terms of chemical shift (δ/ ppm) and multiplicity (C, CH, CH_2 or CH_3). COSY, HSQC and HMBC experiments were used in the structural assignment.

IR spectra were obtained by use of a Bruker Platinum spectrometer (neat, ATR sampling) with the intensities of the characteristic signals being reported as weak (w, <20% of tallest signal), medium (m, 21-70% of tallest signal) or strong (s, >71% of tallest signal) .

High-resolution mass spectrometry was performed using the indicated techniques on amicromass LCT orthogonal time-of-flight mass spectrometer with leucine-enkephalin (Tyr-Gly-Phe-Leu) as an internal lock mass.

Thermal continuous flow experiments were performed using a Vapourtec easy-Scholar system.

2. Synthetic Procedures and Spectroscopic Data

Procedure for the synthesis of *N*-hydroxylsulfonamides, **10a – 10b**

Prepared according to modified literature procedure.[SI1] Hydroxylamine hydrochloride (557 mg, 8 mmol, 2 equiv.) was dissolved in water (8 mL, 1 M) at 0 ^{0}C. A solution of Na_2CO_3 (848 mg, 8 mmol, 2 equiv.) in water (4 mL, 2 M) was added dropwise to the hydroxylamine solution at an internal reaction temperature of 5-15 ^{o}C and stirred for 15 min. THF (4 mL) and methanol (1 mL) were added, followed by the addition of *p*-toluenesulfonyl chloride (763 mg, 4 mmol, 1 equiv) in portions at an internal reaction temperature of 5-15 ^{0}C. After complete addition, the reaction was stirred at r.t. for 4 h. The mixture was extracted with EtO_2 (2 x 20 mL) and the combined organic layers were washed with brine and dried over Na_2SO_4 and filtered. Solvent was evaporated *in vacuo* and the resulting residue was used without further purification.

Procedure for the synthesis of *N*-hydroxylsulfonamide, **10c**

Prepared according to a modified literature procedure.[SI2] EtOAc (10 mL) was mixed with a solution of $NaHCO_3$ (2.0 g, 24 mmol, 2.4 equiv.) in water (5 mL, 4.8 M). Hydroxylamine hydrochloride (834 mg, 12 mmol, 1.2 equiv.) was added and the reaction mixture was stirred until dissolved at r.t.. A solution of 4-trifluoromethyl benzoyl chloride (2.1 g, 10 mmol, 1 equiv.) in ethyl acetate (5 mL, 2 M) was added dropwise. The mixture was stirred at r.t. for 15 mins. EtOAc was removed *in vacuo*, the resulting residue was filtered under vacuum and used without further purification.

General procedure 1 for the synthesis of propargyl alcohols, **9a-9v**

Prepared according to a modified literature procedure.[SI3] LiHMDS (5.5 mL, 5.5 mmol, 1.375 equiv.) was added to a clean, dry flask, followed by toluene (5 mL, 1.1 M) at -78 ^{o}C under N_2 atmosphere. The alkyne solution (5 mmol, 1.25 equiv.) was added dropwise to the solution. The reaction was stirred at -78 ^{0}C for 20 min and then at r.t. for 1 hour. After cooling to -78 ^{o}C, the aldehyde solution (4 mmol, 1 equiv.) was added dropwise and the reaction was stirred at -78 ^{o}C for 20 min and then at r.t. for 1 h, before being quenched with aqueous NH_4Cl (20 mL). The mixture was extracted with EtOAc (2 x 20 mL) and the combined organic layers were washed with brine and dried over Na_2SO_4 and filtered. Solvent was evaporated *in vacuo* and the resulting residue was purified by flash chromatography (EtOAc:cyclohexane, 3:7).

General procedure 2 for the synthesis of isoxazolines, **11a-11r**

Prepared according to a modified literature procedure.[SI4] To a stirred solution of propargyl alcohol (1.2 mmol) in DCM (5 mL, 0.24 M), $FeCl_3{\cdot}6H_2O$ (2.5 mol%) and hydroxylamine (187

mg, 1 mmol) were added and the solution was refluxed for 30 min. Pyridine (10 mol%) was then added to the reaction mixture, followed by $NaAuCl_4 \cdot 2H_2O$ (5 mol%) and the refluxed was maintained for 2 h. The reaction mixture was filtered, solvent was evaporated *in vacuo* and the resulting residue was purified by flash chromatography (EtOAc:cyclohexane, 3:7).

General procedure 3 for the synthesis of aziridines **12a-12r** (batch)

A solution of isoxazoline in toluene (0.125 M) was refluxed for 1-3 h. Solvent was evaporated *in vacuo* and the resulting residue was purified by flash chromatography (EtOAc:cyclohexane, 3:7).

General procedure 4 for the synthesis of aziridines **12a-12r** (flow)

The flow system was flushed with MeCN prior to adding reagents. A solution of isoxazoline in MeCN (0.125 M, 0.15 M, or 1 M) was injected at the appropriate flow rate (1 mL/min, 10 min residence time or 2 mL/min, 5 min residence time) under 8 bar of pressure. The resulting reaction stream was collected, the solvent was evaporated *in vacuo* and the resulting residue was purified by flash chromatography (EtOAc:cyclohexane, 3:7).

3. Unsuccessful Substrates

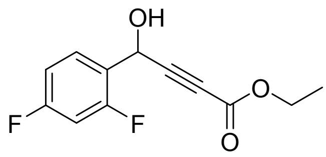

Decomposition

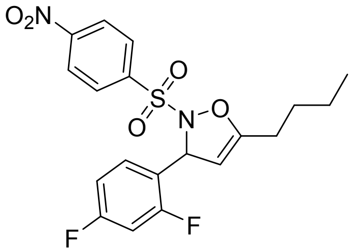

No reaction

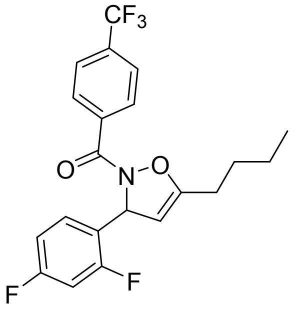

No reaction

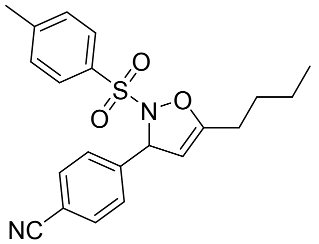

0%

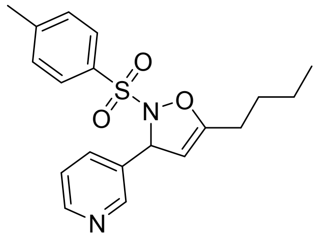

Decomposition

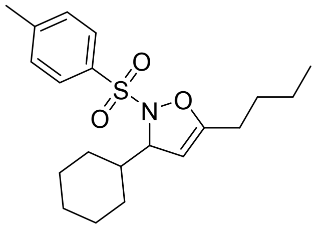

No reaction

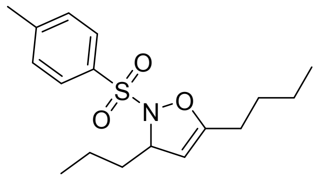

No reaction

4.1 Propargyl Alcohols

1-(2,4-Difluorophenyl)hept-2-yn-1-ol, 9a

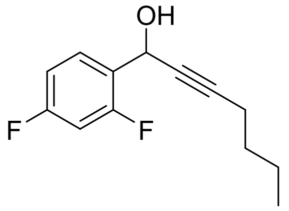

Chemical Formula: $C_{13}H_{14}F_2O$
Exact Mass: 224.1013

Yield: 85% (762 mg, 3.40 mmol)

Appearance: Pale yellow liquid

HR-MS (QTOF) m/z: $[M+H]^+$ Calcd for $C_{13}H_{14}F_2OH^+$ 225.1085; Found 225.1087

Prepared according to General Procedure 1 and purified by flash chromatography (EtOAc:cyclohexane, 3:7, R_f = 0.53). **^{1}H-NMR (500 MHz, CDCl$_3$)** δ/ppm 7.63 (td, *J* = 8.8, 6.8 Hz, 1H), 6.90 – 6.86 (m, 1H), 6.80 (ddd, *J* = 11.2, 8.8, 2.4 Hz, 1H), 5.67 (s, 1H), 2.47 (br s, 1H), 2.26 (td, *J* = 7.3, 2.2 Hz, 2H), 1.54 – 1.48 (m, 2H), 1.44 – 1.37 (m, 2H), 0.91 (t, *J* = 7.3 Hz, 3H). **^{13}C-NMR (126 MHz, CDCl$_3$)** δ/ppm 162.9 (dd, *J* = 250, 12 Hz, CF), 160.3 (dd, *J* = 250 Hz, 12 Hz, CF), 129.4 (dd, *J* = 10, 5 Hz, CH), 124.6 (dd, *J* = 13, 4 Hz, C), 111.3 (dd, *J* = 22, 4 Hz, CH), 103.9 (dd, *J* = 26, 25 Hz, CH), 87.9 (C), 78.6 (C), 58.6 (d, *J* = 5 Hz, CH), 30.5 (CH_2), 21.9 (CH_2), 18.4 (CH_2), 13.5 (CH_3). **^{19}F-NMR (376 MHz, CDCl$_3$)** δ/ppm -115.2 (m), -110.0 (m). **IR (neat)** ν/cm^{-1}: 3326 (br w), 2958 (m), 2933 (m), 2863 (m), 2226 (w), 1608 (s), 1501 (s), 1268 (m), 965 (s), 849 (s).

1-(4-Fluorophenyl)hept-2-yn-1-ol, 9b

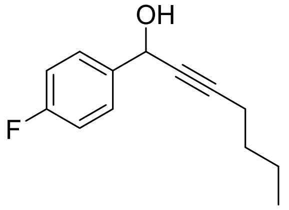

Chemical Formula: $C_{13}H_{15}FO$
Exact Mass: 206.1107

Yield: 81% (668 mg, 3.24 mmol)

Appearance: Pale yellow liquid

HR-MS (QTOF) m/z: $[M+H]^+$ Calcd for $C_{13}H_{15}FOH^+$ 207.1180; Found 207.1180

Prepared according to General Procedure 1 and purified by flash chromatography (EtOAc:cyclohexane, 3:7, R_f = 0.55). **^{1}H-NMR (500 MHz, CDCl$_3$)** δ/ppm 7.54 – 7.50 (m, 2H), 7.07 – 7.03 (m, 2H), 5.43 (d, *J* = 5.9 Hz, 1H), 2.28 (td, *J* = 7.1, 2.0 Hz, 2H), 2.10 (d, *J* = Hz, 1H) 1.55 – 1.50 (m, 2H), 1.46 – 1.38 (m, 2H), 0.92 (t, *J* = 7.3 Hz, 3H). **^{13}C-NMR (126 MHz, CDCl$_3$)** δ/ppm 162.6 (d, *J* = 247 Hz, CF), 137.1 (C), 128.5 (d, *J* = 8 Hz, 2CH), 115.3 (d, *J* = 21 Hz, 2CH), 88.0 (C), 79.7 (C), 64.2 (CH), 30.6 (CH_2), 22.0 (CH_2), 18.5 (CH_2), 13.6 (CH_3). **^{19}F NMR (470 MHz, CDCl$_3$)** δ/ppm -114.2 (m). **IR (neat)** ν/cm^{-1}: 3349 (br w), 2958 (m), 2933 (m), 2864 (m), 2223 (w), 1893 (w), 1506 (s), 1222 (s), 993 (s), 838 (s).

This data is consistent with published work. [SI5]

1-(4-Bromophenyl)hept-2-yn-1-ol, 9c

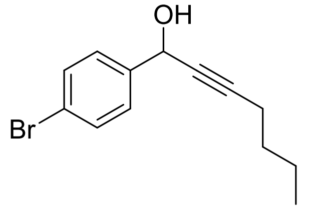

Chemical Formula: $C_{13}H_{15}BrO$
Exact Mass: 266.0306

Yield: 78% (833 mg, 3.12 mmol)

Appearance: Pale yellow liquid

HR-MS (QTOF) m/z: $[M+H]^+$ Calcd for $C_{13}H_{15}BrOH^+$ 267.0379; Found 267.0379

Prepared according to General Procedure 1 and purified by flash chromatography (EtOAc:cyclohexane, 3:7, R_f = 0.48). **^{1}H NMR (500 MHz, CDCl$_3$)** δ/ppm 7.50 – 7.47 (m, 2H), 7.42 – 7.39 (m, 2H), 5.40 (m, 1H), 2.26 (td, *J* = 7.3, 2.2 Hz, 2H), 1.55 – 1.48 (m, 2H), 1.45 – 1.37 (m, 2H), 0.91 (t, *J* = 7.1 Hz, 3H). **^{13}C NMR (126 MHz, CDCl$_3$)** δ/ppm 140.3 (C), 131.6 (2CH), 128.3 (2CH), 122.1 (C), 88.1 (C), 79.5 (C), 64.1 (CH), 30.6 (CH_2), 22.0 (CH_2), 18.5 (CH_2), 13.6 (CH_3). **IR (neat)** v/cm^{-1}: 3326 (br w), 2957 (m), 2931 (m), 2871 (m), 2223 (w), 1485 (m), 1379 (m), 1009 (s), 944 (w), 772 (m).

This data is consistent with published work. [SI5]

1-(2-Chlorophenyl)hept-2-yn-1-ol, 9d

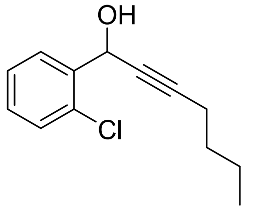

Chemical Formula: $C_{13}H_{15}ClO$
Exact Mass: 222.0811

Yield: 59% (526 mg, 2.36 mmol)

Appearance: Pale yellow liquid

HR-MS (QTOF) m/z: $[M+H]^+$ Calcd for $C_{13}H_{15}ClOH^+$ 223.0884; Found 223.0883

Prepared according to General Procedure 1 and purified by flash chromatography (EtOAc:cyclohexane, 3:7, R_f = 0.58). **^{1}H-NMR (400 MHz, CDCl$_3$)** δ/ppm 7.76 (dd, *J* = 7.7, 1.9 Hz, 1H), 7.38 – 7.24 (m, 4H), 5.82 – 5.80 (m 1H), 2.35 (d, *J* = 5.5 Hz, 1H), 2.27 (td, *J* = 7.1, 2.1 Hz, 2H), 1.55 – 1.48 (m, 2H), 1.47 – 1.37 (m, 2H), 0.91 (t, *J* = 7.2 Hz, 3H). **^{13}C-NMR (101 MHz, CDCl$_3$)** δ/ppm 138.5 (C), 132.8 (C), 129.7 (CH), 129.5 (CH), 128.3 (CH), 127.1 (CH), 87.9 (C), 78.7 (C), 62.2 (CH), 30.6 (CH_2), 22.0 (CH_2), 18.5 (CH_2), 13.6 (CH_3). **IR (neat)** v/cm^{-1}: 3358 (br w), 2957 (m), 2932 (m), 2872 (m), 2226 (w), 1467 (m), 1251 (w), 1135 (m), 995 (m), 750 (s).

This data is consistent with published work. [SI6]

1-(3-Chloro-4-fluorophenyl)hept-2-yn-1-ol, 9e

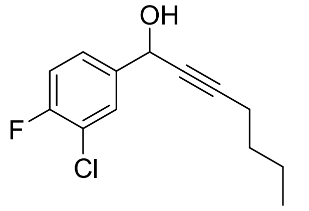

Chemical Formula: $C_{13}H_{14}ClFO$
Exact Mass: 240.0717

Yield: 62% (597 mg, 2.48 mmol)

Appearance: Pale yellow liquid

HR-MS (QTOF) m/z: $[M+H]^+$ Calcd for $C_{13}H_{14}ClFOH^+$ 241.0790; Found 241.0787

Prepared according to General Procedure 1 and purified by flash chromatography (EtOAc:cyclohexane, 3:7, R_f = 0.49). **^{1}H NMR (400 MHz, CDCl$_3$)** δ/ppm 7.58 (dd, *J* = 7.0, 2.2 Hz, 1H), 7.40 – 7.36 (m, 1H), 7.11 (t, *J* = 8.7 Hz, 1H), 5.39 – 5.38 (m, 1H), 2.28 – 2.24 (m, 2H), 1.56 – 1.48 (m, 2H), 1.46 – 1.36 (m, 2H), 0.91 (t, *J* = 7.3 Hz, 3H). **^{13}C NMR (101 MHz, CDCl$_3$)** δ/ppm 157.9 (d, *J* = 249 Hz, CF), 138.4 (d, *J* = 4 Hz, C), 129.1 (CH), 126.5 (d, *J* = 8 Hz, CH), 121.0 (d, *J* = 18 Hz, C), 116.6 (d, *J* = 21 Hz, CH), 88.6 (C), 79.3 (C), 63.7 (CH), 30.6 (CH_2), 22.1 (CH_2), 18.5 (CH_2), 13.6 (CH_3). **^{19}F-NMR (376 MHz, CDCl$_3$)** δ/ppm -116.4 (m). **IR (neat)** v/cm^{-1}: 3329 (w), 2959 (m), 2933 (m), 2873 (w), 2227 (w), 1496 (s), 1249 (s), 1123 (m), 738 (m), 686 (m).

1-(4-(Trifluoromethyl)phenyl)hept-2-yn-1-ol, 9f

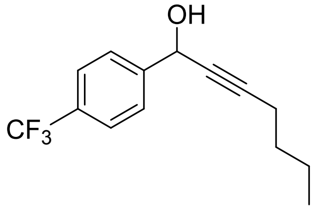

Chemical Formula: $C_{14}H_{15}F_3O$
Exact Mass: 256.1075

Yield: 70% (718 mg, 2.80 mmol)

Appearance: Pale yellow liquid

HR-MS (QTOF) m/z: $[M+Na]^+$ Calcd for $C_{14}H_{15}F_3ONa^+$ 279.0967; Found 279.0935

Prepared according to General Procedure 1 and purified by flash chromatography (EtOAc:cyclohexane, 3:7, R_f = 0.52). **^{1}H NMR (400 MHz, CDCl$_3$)** δ/ppm 7.65 – 7.60 (m, 4H), 5.48 (s, 1H), 2.26 (td, *J* = 6.8, 2.0 Hz, 4H), 1.55 – 1.48 (m, 2H), 1.45 – 1.36 (m, 2H), 0.91 (t, *J* = 7.2 Hz, 3H). **^{13}C NMR (101 MHz, CDCl$_3$)** δ/ppm 145.0 (d, *J* = 2 Hz, C), 130.2 (q, *J* = 33 Hz, C), 126.8 (2CH), 125.4 (q, *J* = 4 Hz, 2CH), 124.0 (q, *J* = 272 Hz, CF_3), 88.4 (C), 79.3 (C), 64.1 (CH), 30.5 (CH_2), 21.9 (CH_2), 18.4 (CH_2), 13.5 (CH_3). **^{19}F NMR (376 MHz, CDCl$_3$)** δ/ppm -62.6 (s). **IR (neat)** v/cm^{-1}: 3309 (br w), 2934 (m), 2865 (m), 2225 (w), 1620 (m), 1466 (m), 1325 (s), 1127 (s), 1017 (s), 861 (m).

This data is consistent with published work. [SI7]

1-Phenylhept-2-yn-1-ol, 9g

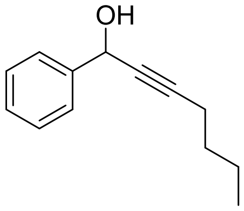

Chemical Formula: $C_{13}H_{16}O$
Exact Mass: 188.1201

Yield: 71% (535 mg, 2.84 mmol)

Appearance: Pale yellow liquid

HR-MS (QTOF) m/z: $[M+H]^+$ Calcd for $C_{13}H_{16}OH^+$ 189.1274; Found 189.1274

Prepared according to General Procedure 1 and purified by flash chromatography (EtOAc:cyclohexane, 3:7, R_f = 0.54). **^{1}H NMR (500 MHz, $CDCl_3$)** δ/ppm 7.55 (d, *J* = 7.3 Hz, 2H), 7.39 – 7.37 (m, 2H), 7.34 – 7.31 (m, 1H), 5.45 (s, 1H), 2.39 (d, *J* = 4.9 Hz, 1H), 2.29 (td, *J* = 7.1, 2.0 Hz, 2H), 1.57 – 1.51 (m, 2H), 1.48 – 1.40 (m, 2H), 0.94 (t, *J* = 7.3 Hz, 3H). **^{13}C NMR (126 MHz, $CDCl_3$)** δ/ppm 141.3 (C), 128.5 (2CH), 128.2 (CH), 126.6 (2CH), 87.6 (C), 80.0 (C), 64.8 (CH), 30.7 (CH_2), 22.0 (CH_2), 18.5 (CH_2), 13.6 (CH_3). **IR (neat)** ν/cm^{-1}: 3351(br w), 2957 (m), 2932 (m), 2871 (m), 2226 (w), 1951 (w), 1453 (m), 1134 (m), 999 (m), 696 (s).

This data is consistent with published work. [SI6]

1-(3,4-Dimethylphenyl)hept-2-yn-1-ol, 9h

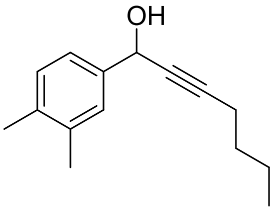

Chemical Formula: $C_{15}H_{20}O$
Exact Mass: 216.1514

Yield: 77% (666 mg, 3.08 mmol)

Appearance: Pale yellow liquid

HR-MS (QTOF) m/z: $[M+H]^+$ Calcd for $C_{15}H_{20}OH^+$ 217.1587; Found 217.1588

Prepared according to General Procedure 1 and purified by flash chromatography (EtOAc:cyclohexane, 3:7, R_f = 0.55). **^{1}H-NMR (500 MHz, $CDCl_3$)** δ/ppm 7.30 – 7.25 (m, 2H), 7.14 (d, J = 7.8 Hz, 1H), 5.39 (m, 1H), 2.29 – 2.26 (m, 8H), 1.54 – 1.50 (m, 2H), 1.47 – 1.39 (m, 2H), 0.92 (t, J = 7.1 Hz, 2H). **^{13}C-NMR (126 MHz, $CDCl_3$)** δ/ppm 138.9 (C), 136.8 (C), 136.7 (C), 129.8 (CH), 127.9 (CH), 124.0 (CH), 87.4 (C), 80.1 (C), 64.8 (CH), 30.7 (CH_2), 22.0 (CH_2), 19.8 (CH_3), 19.5 (CH_3), 18.5 (CH_2), 13.6 (CH_3). **IR (neat)** ν/cm^{-1}: 3335 (br w), 2956 (s), 2930 (s), 2860 (m), 2224 (w), 1454 (s), 1382 (m), 1118 (m), 989 (s), 763 (m).

1-(3,4-Dimethoxyphenyl)hept-2-yn-1-ol, 9i

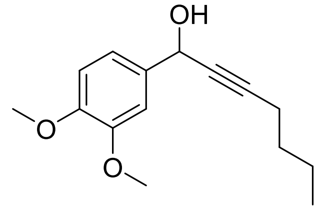

Chemical Formula: $C_{15}H_{20}O_3$
Exact Mass: 248.1412

Yield: 78% (775 mg, 3.12 mmol)

Appearance: Yellow liquid

HR-MS (QTOF) m/z: $[M+Na]^+$ Calcd for $C_{15}H_{20}O_3Na^+$ 271.1305; Found 271.1303

Prepared according to General Procedure 1 and purified by flash chromatography (EtOAc:cyclohexane, 3:7, R_f = 0.26). **^{1}H NMR (500 MHz, $CDCl_3$)** δ/ppm 7.10 – 7.06 (m, 2H), 6.84 (d, *J* = 7.8 Hz, 1H), 5.39 (s, 1H), 3.89 (s, 3H), 3.87 (s, 3H), 2.27 (td, *J* = 7.1, 2.0 Hz, 2H), 1.55 – 1.49 (m, 2H), 1.46 – 1.39 (m, 2H), 0.91 (t, *J* = 7.3 Hz, 3H). **^{13}C NMR (126 MHz, $CDCl_3$)** δ/ppm 149.0 (C), 149.0 (C), 134.0 (C), 119.0 (CH), 110.9 (CH), 109.9 (CH), 87.5 (C), 80.0 (C), 64.7 (CH), 55.9 (CH_3), 55.8 (CH_3), 30.6 (CH_2), 22.0 (CH_2), 18.5 (CH_2), 13.6 (CH_3). **IR (neat)** v/cm^{-1}: 3490 (br w), 2956 (m), 2933 (m), 2871 (w), 2220 (w), 1513 (s), 1260 (s), 1130 (m), 1026 (s), 762 (m).

1-(3-Methylthiophen-2-yl)hept-2-yn-1-ol, 9j

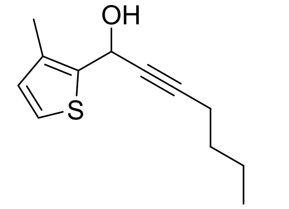

Chemical Formula: $C_{12}H_{16}OS$
Exact Mass: 208.0922

Yield: 67% (558 mg, 2.68 mmol)

Appearance: Brown oil

HR-MS (QTOF) m/z: $[M+H]^+$ Calcd for $C_{12}H_{16}OSH^+$ 209.0995; Found 209.0998

Prepared according to General Procedure 1 and purified by flash chromatography (EtOAc:cyclohexane, 3:7, R_f = 0.52). **^{1}H NMR (500 MHz, $CDCl_3$)** δ/ppm 7.16 (d, *J* = 4.9 Hz, 1H), 6.82 (d, *J* = 4.9 Hz, 1H), 5.66 – 5.65 (m, 1H), 2.29 (s, 3H), 2.27 (dd, *J* = 7.1, 2.2 Hz, 2H), 1.57 – 1.51 (m, 2H), 1.48 – 1.41 (m, 2H), 0.93 (t, *J* = 7.1 Hz, 3H). **^{13}C NMR (126 MHz, $CDCl_3$)** δ/ppm 138.3 (C), 134.9 (C), 130.5 (CH), 123.8 (CH), 86.9 (C), 79.7 (C), 58.6 (CH), 30.5 (CH_2), 21.9 (CH_2), 18.4 (CH_2), 13.7 (CH_3), 13.6 (CH_3). **IR (neat)** v/cm^{-1}: 3272 (br w), 2957 (m), 2929 (m), 2870 (m), 2217 (w), 1682 (m), 1454 (m), 1377 (m), 1124 (s), 965 (m), 712 (s).

1-(Naphthalen-2-yl)-3-phenylprop-2-yn-1-ol, 9k

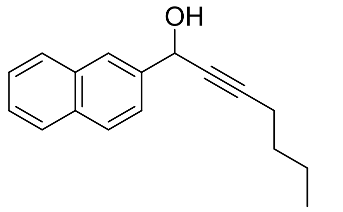

Chemical Formula: $C_{17}H_{18}O$
Exact Mass: 238.1358

Yield: 71% (676 mg, 2.84 mmol)

Appearance: Pale yellow liquid

HR-MS (QTOF) m/z: $[M+H]^+$ Calcd for $C_{17}H_{18}OH^+$ 239.1430; Found 239.1434

Prepared according to General Procedure 1 and purified by flash chromatography (EtOAc:cyclohexane, 3:7, R_f = 0.50). **^{1}H NMR (500 MHz, $CDCl_3$)** δ/ppm 8.00 (s, 1H), 7.88 – 7.85 (m, 3H), 7.68 (dd, *J* = 8.6, 1.7 Hz, 1H), 7.53 – 7.49 (m, 2H), 5.64 (d, *J* = 6.5 Hz, 1H), 2.37 (d, *J* = 5.9 Hz, 1H), 2.33 (td, *J* = 7.1, 2.0 Hz, 2H), 1.61 – 1.55 (m, 2H), 1.51 – 1.44 (m, 2H), 0.96 (t, *J* = 7.3 Hz, 3H). **^{13}C NMR (126 MHz, $CDCl_3$)** δ/ppm 138.7 (C), 133.2 (C), 133.2 (C), 128.4 (CH), 128.2 (CH), 127.7 (CH), 126.2 (CH), 126.2 (CH), 125.3 (CH), 124.7 (CH), 88.0 (C), 80.0 (C), 65.0 (CH), 30.7 (CH_2), 22.0 (CH_2), 18.6 (CH_2), 13.6 (CH_3). **IR (neat)** v/cm^{-1}: 3376 (br w), 2956 (m), 2930 (m), 2861 (m), 2214 (w), 1916 (w), 1690 (m), 1118 (m), 999 (m), 754 (s).

This data is consistent with published work. [SI7]

3-Cyclopropyl-1-(2,4-difluorophenyl)prop-2-yn-1-ol, 9l

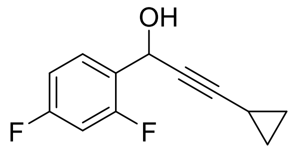

Chemical Formula: $C_{12}H_{10}F_2O$
Exact Mass: 208.0700

Yield: 81% (675 mg, 3.24 mmol)

Appearance: Pale yellow liquid

HR-MS (QTOF) m/z: $[M+H]^+$ Calcd for $C_{12}H_{10}F_2OH^+$ 209.0772; Found 209.0775

Prepared according to General Procedure 1 and purified by flash chromatography (EtOAc:cyclohexane, 3:7, R_f = 0.49). **^{1}H NMR (500 MHz, CDCl$_3$)** δ/ppm 7.60 (td, *J* = 8.3, 6.4 Hz, 1H), 6.90 – 6.85 (m, 1H), 6.79 (ddd, *J* = 10.3, 8.8, 2.4 Hz, 1H), 5.64 (dd, *J* = 5.4, 1.5 Hz, 1H), 2.45 (d, *J* = 5.9 Hz, 1H), 1.30 – 1.27 (m, 1H), 0.81 – 0.78 (m, 2H), 0.73 – 0.70 (m, 2H). **^{13}C NMR (126 MHz, CDCl$_3$) δ** 163.4 (dd, *J* = 249, 12 Hz, CF), 160.8 (dd, *J* = 249, 12 Hz, CF) 130.0 (dd, *J* = 9, 5 Hz, CH), 125.1 (dd, *J* = 13, 4 Hz, C), 111.9 (dd, *J* = 21, 4 Hz CH), 104.5 (dd, *J* = 26, 26 Hz, CH), 91.4 (C), 74.3 (C), 59.2 (d, *J* = 4 Hz, CH), 8.9 (2CH$_2$), 0.0 (CH). **^{19}F NMR (376 MHz, CDCl$_3$)** δ -115.2 (m), -110.0 (m). **IR (neat)** ν/cm^{-1}: 3334 (br w), 3083 (w), 3012 (w), 2237 (m), 1610 (s), 1501 (s), 1270 (w), 1139 (s), 965 (s), 850 (s).

1-(4-Bromophenyl)-3-cyclopropylprop-2-yn-1-ol, 9m

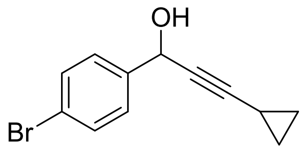

Chemical Formula: $C_{12}H_{11}BrO$
Exact Mass: 249.9993

Yield: 78% (784 mg, 3.12 mmol)

Appearance: Pale yellow liquid

HR-MS (QTOF) m/z: $[M+Na]^+$ Calcd for $C_{12}H_{11}BrONa^+$ 272.9985; Found 272.9982

Prepared according to General Procedure 1 and purified by flash chromatography (EtOAc:cyclohexane, 3:7, R_f = 0.46). **^{1}H NMR (500 MHz, CDCl$_3$)** δ/ppm 7.50 – 7.48 (m, 2H), 7.40 – 7.38 (m, 2H), 5.37 (m, 1H), 2.12 (d, *J* = 5.9 Hz, 1H), 1.30 (m, 1H), 0.83 – 0.79 (m, 2H), 0.72 (dt, *J* = 7.3, 4.4 Hz, 2H). **^{13}C NMR (126 MHz, CDCl$_3$)** δ/ppm 140.2 (C), 131.6 (2CH), 128.3 (2CH), 122.1 (C), 91.1 (C), 74.7 (C), 64.1 (CH), 8.3 (2CH$_2$). **IR (neat)** ν/cm^{-1}: 3331 (br m), 3092 (w), 3010 (w), 2864 (w), 2236 (m), 1630 (m), 1485 (s), 1154 (m), 983 (s), 738 (s).

This data is consistent with published work. [SI8]

3-Cyclopropyl-1-(naphthalen-2-yl)prop-2-yn-1-ol, 9n

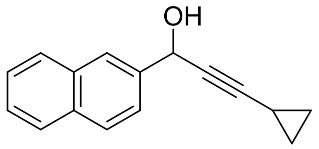

Chemical Formula: $C_{16}H_{14}O$
Exact Mass: 222.1045

Yield: 77% (685 mg, 3.10 mmol)

Appearance: Pale yellow liquid

HR-MS (QTOF) m/z: $[M+H]^+$ Calcd for $C_{16}H_{14}O$ 223.1025; Found 223.1031

Prepared according to General Procedure 1 and purified by flash chromatography (EtOAc:cyclohexane, 3:7, R_f = 0.38). **^{1}H NMR (500 MHz, CDCl$_3$)** δ/ppm 7.96 (s, 1H), 7.88 – 7.83 (m, 3H), 7.65 (dd, *J* = 8.6, 1.7 Hz, 1H), 7.53 – 7.49 (m, 2H), 5.59 (dd, *J* = 6.0, 1.6 Hz, 1H), 2.58 (d, *J* = 5.9 Hz, 1H), 1.36 (ddtd, *J* = 8.3, 6.8, 5.1, 1.7 Hz, 1H), 0.85 – 0.76 (m, 4H). **^{13}C NMR (126 MHz, CDCl$_3$) δ/ppm** 138.6 (C), 133.2 (C), 133.2 (C), 128.5 (CH), 128.3 (CH),

127.7 (CH), 126.2 (CH), 126.2 (CH), 125.3 (CH), 124.7 (CH), 91.1 (C), 75.3 (C), 64.9 (CH), 8.4 ($2CH_2$), -0.3 (CH). **IR (neat)** ν/cm^{-1}: 3358 (br w), 3091 (w), 3054 (w), 3009 (w), 2234 (w), 1508 (m), 1358 (m), 1121 (m), 991 (m), 754 (s).

This data is consistent with published work. [SI9]

1-(2,4-Difluorophenyl)-3-phenylprop-2-yn-1-ol, 9o

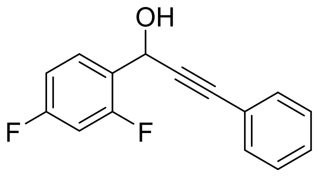

Chemical Formula: $C_{15}H_{10}F_2O$
Exact Mass: 244.0700

Yield: 72% (703 mg, 2.88 mmol)

Appearance: Pale yellow liquid

HR-MS (QTOF) m/z: $[M+H]^+$ Calcd for $C_{15}H_{10}F_2OH^+$ 245.0772; Found 245.0773

Prepared according to General Procedure 1 and purified by flash chromatography (EtOAc:cyclohexane, 3:7, R_f = 0.34). **^{1}H NMR (400 MHz, CDCl$_3$)** δ/ppm 7.71 (td, *J* = 8.6, 6.7 Hz, 1H), 7.47 – 7.44 (m, 2H), 7.34 – 7.28 (m, 3H), 6.93 – 6.88 (m, 1H), 6.86 – 6.80 (m, 1H), 5.92 (d, *J* = 5.9 Hz, 1H), 2.59 (d, *J* = 5.5 lHz, 1H). **^{13}C NMR (101 MHz, CDCl$_3$)** δ/ppm 163.0 (dd, *J* = 250, 12 Hz, CF), 160.3 (dd, *J* = 250 Hz, 12 Hz, CF), 131.8 (2CH), 129.6 (dd, *J* = 10 Hz, 5 Hz, CH), 128.8 (CH), 128.3 (2CH), 124.0 (dd, *J* = 13, 4 Hz, C), 122.0 (C), 111.5 (dd, *J* = 21 Hz, 4 Hz, CH), 104.1 (dd, *J* = 26, 25 Hz, CH), 87.3 (C), 86.8 (C), 59.0 (d, *J* = 5 Hz, CH). **^{19}F NMR (376 MHz, CDCl$_3$)** δ/ppm -114.8 (m), -109.5 (m). **IR (neat)** ν/cm^{-1}: 3322 (br w), 3080 (w), 2232 (w), 1894 (w), 1611 (s), 1502 (s), 1273 (m), 972 (s), 851 (m), 757 (s).

This data is consistent with published work. [SI10]

1-(4-Fluorophenyl)-3-phenylprop-2-yn-1-ol, 9p

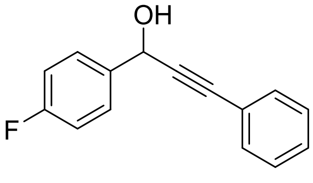

Chemical Formula: $C_{15}H_{11}FO$
Exact Mass: 226.0794

Yield: 78% (705 mg, 3.12 mmol)

Appearance: Pale yellow liquid

HR-MS (QTOF) m/z: $[M+Na]^+$ Calcd for $C_{15}H_{11}FONa^+$ 249.0686; Found 249.0685

Prepared according to General Procedure 1 and purified by flash chromatography (EtOAc:cyclohexane, 3:7, R_f = 0.33). **^{1}H NMR (500 MHz, CDCl$_3$)** δ/ppm 7.61 – 7.57 (m, 2H), 7.49 – 7.47 (m, 2H), 7.35 – 7.31 (m, 3H), 7.11 – 7.06 (m, 2H), 5.68 (d, *J* = 6.4 Hz, 1H), 2.49 (d, *J* = 5.9 Hz, 1H). **^{13}C NMR (126 MHz, CDCl$_3$)** δ/ppm 162.7 (d, *J* = 247 Hz, CF), 136.5 (d, *J* = 3 Hz, C), 131.7 (2CH), 128.7 (CH), 128.6 (d, *J* = 9 Hz, 2CH), 128.4 (2CH), 122.2 (C), 115.5 (d, *J* = 22 Hz, 2CH), 88.5 (C), 86.9 (C), 64.4 (CH). **^{19}F NMR (376 MHz, CDCl$_3$)** δ/ppm -113.8 (m). **IR (neat)** ν/cm^{-1}: 3332 (br w), 3058 (w), 2899 (w), 2228 (w), 1893 (w), 1604 (m), 1507 (s), 1221 (s), 835 (s), 754 (s).

This data is consistent with published work. [SI11]

1-(4-Bromophenyl)-3-phenylprop-2-yn-1-ol, 9q

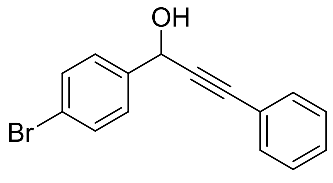

Chemical Formula: $C_{15}H_{11}BrO$
Exact Mass: 285.9993

Yield: 70% (804 mg, 2.80 mmol)

Appearance: Pale yellow liquid

HR-MS (QTOF) m/z: $[M+Na]^+$ Calcd for $C_{15}H_{11}BrONa^+$ 308.9985; Found 308.9982

Melting point: 60 – 62 ^{0}C

Prepared according to General Procedure 1 and purified by flash chromatography (EtOAc:cyclohexane, 3:7, R_f = 0.42). **^{1}H-NMR (500 MHz, $CDCl_3$)** δ/ppm 7.54 – 7.52 (m, 2H), 7.51 – 7.46 (m, 4H), 7.35 – 7.31 (m, 3H), 5.66 (d, *J* = 5.9 Hz, 1H), 2.45 (d, *J* = 5.9 Hz, 1H). **^{13}C-NMR (126 MHz, $CDCl_3$)** δ/ppm 139.6 (C), 131.8 (4CH), 128.80 (3CH), 128.4 (CH), 128.4 (CH), 122.4 (C), 122.1 (C), 88.2 (C), 87.0 (C), 64.4 (CH). **IR (neat)** ν/cm^{-1}: 3292 (br w), 3056 (w), 2873 (w), 2228 (w), 1634 (w), 1486 (s), 1103 (w), 998 (m), 756 (s), 690 (s).

This data is consistent with published work. [SI8]

1-(Naphthalen-2-yl)-3-phenylprop-2-yn-1-ol, 9r

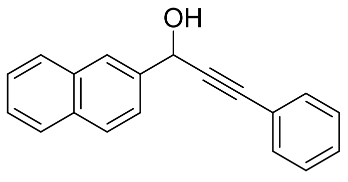

Chemical Formula: $C_{19}H_{14}O$
Exact Mass: 258.1045

Yield: 62% (644 mg, 2.48 mmol)

Appearance: White solid

HR-MS (QTOF) m/z: $[M+Na]^+$ Calcd for $C_{19}H_{14}ONa^+$ 281.0937; Found 281.0939

Melting point: 90 – 91 ^{0}C

Prepared according to General Procedure 1 and purified by flash chromatography (EtOAc:cyclohexane, 3:7, R_f = 0.43). **^{1}H NMR (400 MHz, $CDCl_3$)** δ/ppm 8.04 (s, 1H), 7.88 – 7.83 (m, 3H), 7.73 (dd, *J* = 8.6, 1.6 Hz, 1H), 7.52 – 7.48 (m, 4H), 7.35 – 7.30 (m, 3H), 5.85 (s, 1H), 2.67 (s, 1H). **^{13}C NMR (101 MHz, $CDCl_3$)** δ/ppm 138.0 (C), 133.3 (C), 133.2 (C), 131.8 (2CH), 128.7 (CH), 128.6 (CH), 128.4 (2CH), 128.3 (CH), 127.7 (CH), 126.3 (CH), 126.3 (CH), 125.5 (CH), 124.7 (CH), 122.4 (C), 88.8 (C), 86.9 (C), 65.2 (CH). **IR (neat)** ν/cm^{-1}: 3233 (br w), 2964 (w), 2919 (w), 2851 (w), 2220 (w), 2098 (w), 1488 (m), 1122 (m), 994 (m), 749 (s).

This data is consistent with published work. [SI12]

4-(1-Hydroxyhept-2-yn-1-yl)benzonitrile, 9s

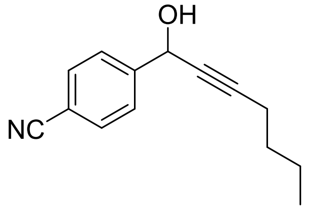

Chemical Formula: $C_{14}H_{15}NO$
Exact Mass: 213.1154

Yield: 68% (580 mg, 2.72 mmol)

Appearance: Pale yellow liquid

HR-MS (QTOF) m/z: $[M+H]^+$ Calcd for $C_{14}H_{15}NOH^+$ 214.1226; Found 214.1226

Prepared according to General Procedure 1 and purified by flash chromatography (EtOAc:cyclohexane, 3:7, R_f = 0.63). **^{1}H NMR (500 MHz, $CDCl_3$)** δ/ppm 7.64 (s, 4H), 5.48 (s,

1H), 2.57 (br s, 1H), 2.26 (td, *J* = 7.1, 2.0 Hz, 2H), 1.53 – 1.48 (m, 2H), 1.43 – 1.36 (m, 2H), 0.90 (t, *J* = 7.3 Hz, 3H). **^{13}C NMR (126 MHz, $CDCl_3$)** δ/ppm 146.4 (C), 132.3 (2CH), 127.2 (2CH), 118.7 (C), 111.7 (C), 88.6 (C), 79.0 (C), 63.9 (CH), 30.5 (CH_2), 21.9 (CH_2), 18.4 (CH_2), 13.5 (CH_3). **IR (neat)** v/cm^{-1}: 3416 (br w), 2957 (s), 2932 (s), 2871 (s), 2229 (s), 1607 (m), 1459 (m), 1262 (s), 1016 (s), 859 (s).

This data is consistent with published work. [SI6]

1-(Pyridin-3-yl)hept-2-yn-1-ol, 9t

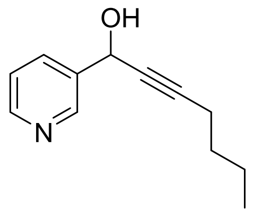

Chemical Formula: $C_{12}H_{15}NO$
Exact Mass: 189.1154

Yield: 75% (567 mg, 3.00 mmol)

Appearance: Pale yellow liquid

HR-MS (QTOF) m/z: $[M+H]^+$ Calcd for $C_{12}H_{15}NOH^+$ 190.1226; Found 190.1230

Prepared according to General Procedure 1 and purified by flash chromatography (EtOAc:cyclohexane, 3:7, R_f = 0.05). **^{1}H NMR (500 MHz, $CDCl_3$)** δ/ppm 8.75 (s, 1H), 8.54 – 8.53 (m, 1H), 7.89 (d, *J* = 7.8 Hz, 1H), 7.31 (dd, *J* = 7.8, 4.9 Hz, 1H), 5.51 (s, 1H), 2.28 (td, *J* = 7.1, 2.0 Hz, 2H), 1.56 – 1.50 (m, 2H), 1.45 – 1.38 (m, 2H), 0.91 (t, *J* = 7.3 Hz, 3H). **^{13}C NMR (126 MHz, $CDCl_3$)** δ/ppm 149.2 (CH), 148.3 (CH), 137.0 (C), 134.5 (CH), 123.4 (CH), 88.5 (C), 79.1 (C), 62.5 (CH), 30.5 (CH_2), 22.0 (CH_2), 18.4 (CH_2), 13.6 (CH_3). **IR (neat)** v/cm^{-1}: 3152 (br w), 2956 (m), 2930 (m), 2860 (m), 2222 (w), 2092 (w), 1593 (m), 1426 (s), 1019 (s), 712 (s).

This data is consistent with published work. [SI13]

1-Cyclohexylhept-2-yn-1-ol, 9u

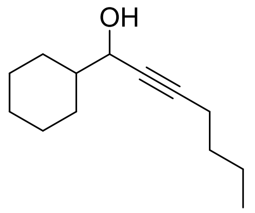

Chemical Formula: $C_{13}H_{22}O$
Exact Mass: 194.1671

Yield: 61% (476 mg, 2.44 mmol)

Appearance: Pale yellow liquid

HR-MS (QTOF) m/z: $[M+H]^+$ Calcd for $C_{13}H_{22}OH^+$ 195.1743; Found 195.1748

Prepared according to General Procedure 1 and purified by flash chromatography (EtOAc:cyclohexane, 3:7, R_f = 0.57). **^{1}H NMR (500 MHz, $CDCl_3$)** δ/ppm 4.13 – 4.12 (m, 1H), 2.22 (td, *J* = 7.1, 2.0 Hz, 2H), 1.85 – 1.82 (m, 2H), 1.78 – 1.75 (m, 2H), 1.69 – 1.65 (m, 1H), 1.53 – 1.51 (m, 1H), 1.51 – 1.46 (m, 2H), 1.44 – 1.37 (m, 2H), 1.27 – 1.19 (m, 2H), 1.19 – 1.10 (m, 2H), 1.05 (qd, *J* = 12.5, 3.7 Hz, 1H), 0.91 (t, *J* = 7.3 Hz, 3H). **^{13}C NMR (126 MHz, $CDCl_3$)** δ/ppm 86.2 (C), 80.1 (C), 67.4 (CH), 44.4 (CH), 30.8 (CH_2), 28.6 (CH_2), 28.1 (CH_2), 26.4 (CH_2), 25.9 (CH_2), 25.9 (CH_2), 21.9 (CH_2), 18.4 (CH_2), 13.6 (CH_3). **IR (neat)** v/cm^{-1}: 3341 (br w), 2955 (m), 2923 (s), 2852 (s), 2225 (w), 1624 (w), 1449 (m), 1141 (m), 1009 (s), 733 (w).

This data is consistent with published work. [SI6]

Dec-5-yn-4-ol, 9v

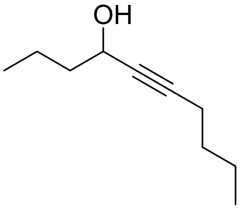

Chemical Formula: $C_{10}H_{18}O$
Exact Mass: 154.1358

Yield: 40% (247 mg, 1.60 mmol)

Appearance: Pale yellow liquid

HR-MS (QTOF) m/z: $[M+Na]^+$ Calcd for $C_{10}H_{18}ONa^+$ 177.1250; Found 177.1249

Prepared according to General Procedure 1 and purified by flash chromatography (EtOAc:cyclohexane, 3:7, R_f = 0.49). **^{1}H NMR (500 MHz, $CDCl_3$)** δ/ppm 4.37 – 4.35 (m, 1H), 2.21 (td, *J* = 7.1, 2.0 Hz, 2H), 1.69 – 1.62 (m, 2H), 1.50 – 1.38 (m, 6H), 0.95 (t, *J* = 7.3 Hz, 3H), 0.91 (t, *J* = 7.3 Hz, 3H). **^{13}C NMR (126 MHz, $CDCl_3$)** δ/ppm 85.5 (C), 81.3 (C), 62.5 (CH), 40.3 (CH_2), 30.7 (CH_2), 21.9 (CH_2), 18.5 (CH_2), 18.3 (CH_2), 13.8 (CH_3), 13.6 (CH_3). **IR (neat)** v/cm^{-1}: 3341 (br w), 2957 (s), 2931 (s), 2872 (m), 1713 (w), 1458 (m), 1149 (m), 1102 (m), 1024 (s), 744 (w).

This data is consistent with published work. [SI14]

4.2 Hydroxylamines

***N*-Hydroxy-4-methylbenzenesulfonamide, 10a**

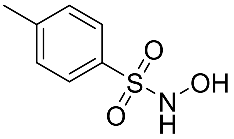

Chemical Formula: $C_7H_9NO_3S$
Exact Mass: 187.0303

Yield: 62% (464 mg, 2.5 mmol)

Appearance: White solid

HR-MS (QTOF) m/z: $[M+H]^+$ Calcd for $C_7H_9NO_3SH^+$ 188.0376; Found 188.0384

Melting point: 141 – 143 ^{0}C

^{1}H NMR (500 MHz, DMSO-d_6) δ/ppm 9.53 (d, *J* = 2.9 Hz, 1H), 9.47 (d, *J* = 3.4 Hz, 1H), 7.71 – 7.69 (m, 2H), 7.40 (d, *J* = 7.8 Hz, 2H), 2.38 (s, 3H). **^{13}C NMR (126 MHz, DMSO-d_6)** δ/ppm 144.0 (C), 134.9 (C), 129.8 (2CH), 128.6 (2CH), 21.5 (CH_3). **IR (neat)** v/cm^{-1}: 3372 (m), 3252 (m), 3221 (w), 1916 (w), 1651 (m), 1494 (w), 1345 (m), 1291 (m), 1159 (s), 718 (s).

This data is consistent with published work. [SI2]

N-Hydroxy-4-nitrobenzenesulfonamide, 10b

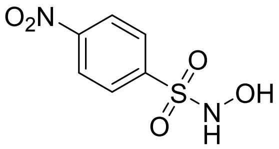

Chemical Formula: $C_6H_6N_2O_5S$
Exact Mass: 217.9997

Yield: 67% (585 mg, 2.7 mmol)

Appearance: Yellow solid

HR-MS (QTOF) m/z: $[M+H]^+$ Calcd for $C_6H_6N_2O_5SH^+$ 219.0070; Found 219.0071

Melting point: 162 – 163 ^{0}C

^{1}H NMR (500 MHz, DMSO-d$_6$) δ/ppm 9.94 (d, *J* = 2.9 Hz, 1H), 9.86 (d, *J* = 2.9 Hz, 1H), 8.43 (d, *J* = 8.8 Hz, 2H), 8.08 (d, *J* = 8.8 Hz, 2H). **^{13}C NMR (126 MHz, DMSO-d$_6$)** δ/ppm 150.6 (C), 143.3 (C), 130.2 (2CH), 124.7 (2CH). **IR (neat) v/cm^{-1}:** 3446 (m), 3245 (m), 3102 (w), 1606 (w), 1524 (m), 1346 (s), 1305 (m), 1163 (s), 913 (m), 742 (s).

This data is consistent with published work. [SI15]

N-hydroxy-4-(trifluoromethyl)benzamide, 10c

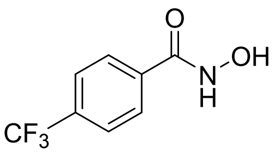

Chemical Formula: $C_8H_6F_3NO_2$
Exact Mass: 205.0351

Yield: 86% (1.755 g, 8.6 mmol)

Appearance: White solid

HR-MS (QTOF) m/z: $[M+H]^+$ Calcd for $C_8H_6F_3NO_2H^+$ 206.0423; Found 206.0425

Melting point: 155 – 156 ^{0}C

^{1}H NMR (400 MHz, DMSO-d$_6$) δ/ppm 7.91 (d, *J* = 7.8 Hz, 2H), 7.79 (d, *J* = 8.2 Hz, 2H). **^{13}C NMR (101 MHz, DMSO-d$_6$)** δ/ppm 163.2 (C), 137.1 (C), 131.6 (q, *J* = 32 Hz, C), 128.2 (2CH), 125.8 (q, *J* = 4 Hz, 2CH), 124.4 (q, *J* = 272 Hz, CF_3). **^{19}F NMR (376 MHz, DMSO-d$_6$)** δ/ppm -61.4 (s). **IR (neat) v/cm^{-1}:** 3300 (m), 3039 (br m), 2754 (br m), 1650 (m), 1560 (m), 1323 (m), 1138 (s), 1071 (s), 864 (s), 763 (m).

This data is consistent with published work. [SI2]

4.3 Isoxazolines

5-Butyl-3-(2,4-difluorophenyl)-2-tosyl-2,3-dihydroisoxazole, 11a

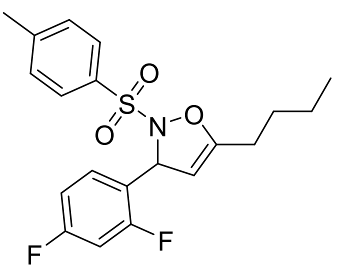

Chemical Formula: $C_{20}H_{21}F_2NO_3S$
Exact Mass: 393.1210

Yield: 93% (364 mg, 0.93 mmol)

Appearance: Pale yellow oil

HR-MS (QTOF) m/z: $[M+H]^+$ Calcd for $C_{20}H_{21}F_2NO_3SH^+$ 394.1283; Found 394.1307

Prepared according to General Procedure 2 and purified by flash chromatography (EtOAc:cyclohexane, 3:7, R_f = 0.52). **^{1}H NMR (400 MHz, CDCl$_3$)** δ/ppm 7.88 (d, *J* = 7.9 Hz, 2H), 7.48 – 7.43 (td, J = 8.6, 6.7 Hz, 1H), 7.36 (d, *J* = 7.9 Hz, 2H), 6.90 – 6.86 (m, 1H), 6.82 – 6.76 (m, 1H), 6.00 (s, 1H), 4.49 (d, *J* = 1.7, 1.2 Hz, 1H), 2.47 (s, 3H), 2.04 – 1.97 (m, 2H), 1.34 – 1.27 (m, 2H), 1.25 – 1.18 (m, 2H), 0.85 (t, *J* = 7.4 Hz, 3H). **^{13}C NMR (101MHz, CDCl$_3$)** δ/ppm 162.6 (dd, *J* = 250, 12 Hz, CF), 159.5 (dd, *J* = 250, 12 Hz, CF), 156.4 (C), 145.5 (C), 130.8 (C), 129.7 (2CH), 129.5 (2CH), 129.4 (dd, *J* = 10, 5 Hz, CH), 123.7 (dd, *J* = 14, 4 Hz, C), 111.8 (dd, *J* = 21, 4 Hz, CH), 103.8 (dd, *J* = 26, 25 Hz, CH), 95.8 (CH), 63.0 (d, *J* = 3 Hz, CH), 28.3 (CH$_2$), 25.1 (CH$_2$), 22.1 (CH$_2$), 21.7 (CH$_3$), 13.6 (CH$_3$). **^{19}F NMR (376 MHz, CDCl$_3$)** δ/ppm -115.5 (m), -110.5 (m). **IR (neat) v/cm^{-1}:** 2957 (m), 2930 (m), 2871 (m), 1687 (m), 1501 (s), 1402 (w), 1365 (s), 1169 (s), 1089 (s), 681 (s).

5-Butyl-3-(4-fluorophenyl)-2-tosyl-2,3-dihydroisoxazole, 11b

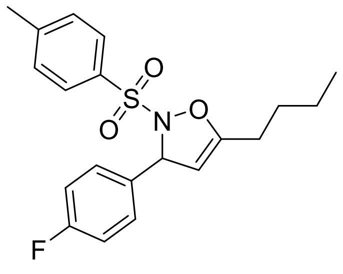

Chemical Formula: $C_{20}H_{22}FNO_3S$
Exact Mass: 375.1304

Yield: 66% (248 mg, 0.66 mmol)

Appearance: Pale yellow oil

HR-MS (QTOF) m/z: [M+H]$^+$ Calcd for $C_{20}H_{22}FNO_3SH^+$ 376.1377; Found 376.1379

Prepared according to General Procedure 2 and purified by flash chromatography (EtOAc:cyclohexane, 3:7, R_f = 0.47). **^{1}H NMR (500 MHz, CDCl$_3$)** δ/ppm 7.87 (d, *J* = 8.3 Hz, 2H), 7.37 – 7.31 (m, 4H), 7.06 – 7.00 (m, 2H), 5.77 – 5.75 (m, 1H), 4.52 – 4.50 (m, 1H), 2.45 (s, 3H), 2.11 – 1.98 (m, 2H), 1.40 – 1.31 (m, 2H), 1.28 – 1.22 (m, 2H), 0.87 (t, *J* = 7.3 Hz, 3H). **^{13}C NMR (126 MHz, CDCl$_3$)** δ/ppm 162.7 (d, *J* = 246 Hz, CF), 156.7 (C), 145.4 (C), 136.0 (d, *J* = 3.8 Hz, C) , 130.9 (C), 129.7 (2CH), 129.5 (2CH), 129.0 (d, *J* = 7.6 Hz, 2CH), 115.6 d, *J* = 21.9 Hz, 2CH), 95.7 (d, *J* = 1.9 Hz, CH), 68.6 (CH), 28.4 (CH$_2$), 25.2 (CH$_2$), 22.1 (CH$_2$), 21.7 (CH$_3$), 13.7 (CH$_3$). **^{19}F NMR (376 MHz, CDCl$_3$)** δ/ppm -114.0 (m). **IR (neat)** v/cm^{-1}: 2961 (w), 2929 (w), 2873 (w), 1687 (m), 1508 (m), 1355 (m), 1217 (m), 1160 (s), 1087 (m), 678 (s).

3-(4-Bromophenyl)-5-butyl-2-tosyl-2,3-dihydroisoxazole, 11c

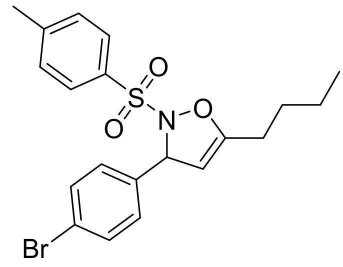

Chemical Formula: $C_{20}H_{22}BrNO_3S$
Exact Mass: 435.0504

Yield: 72% (314 mg, 0.72 mmol)

Appearance: Pale yellow solid

HR-MS (QTOF) m/z: [M+H]$^+$ Calcd for $C_{20}H_{22}BrNO_3SH^+$ 436.0577; Found 436.0577

Prepared according to General Procedure 2 and purified by flash chromatography (EtOAc:cyclohexane, 3:7, R_f = 0.67). **^{1}H NMR (400 MHz, CDCl$_3$)** δ/ppm 7.87 (d, *J* = 8.3 Hz, 2H), 7.47 – 7.49 (m, 2H), 7.35 (d, *J* = 8.3 Hz, 2H), 7.22 – 7.24 (m, 2H), 5.73 (s, 1H), 4.51 –

4.52 (m, 1H), 2.46 (s, 3H), 2.01 – 2.08 (m, 2H), 1.32 – 1.38 (m, 2H), 1.21 – 1.29 (m, 2H), 0.87 (t, *J* = 7.1 Hz, 3H). **^{13}C NMR (101MHz, $CDCl_3$)** δ/ppm 156.9 (C), 145.4 (C), 139.1 (C), 131.8 (2 CH), 131.0 (C), 129.6 (2 CH), 129.5 (2 CH), 128.8 (2 CH), 122.3 (C), 95.4 (CH), 68.4 (CH), 28.4 (CH_2), 25.2 (CH_2), 22.1 (CH_2), 21.7 (CH_3), 13.7 (CH_3). **IR (neat) ν/cm^{-1}:** 2961 (w), 2929 (w), 2860 (w), 1686 (m), 1485 (m), 1413 (w), 1354 (m), 1166 (s), 1088 (w), 681 (s).

5-Butyl-3-(2-chlorophenyl)-2-tosyl-2,3-dihydroisoxazole, 11d

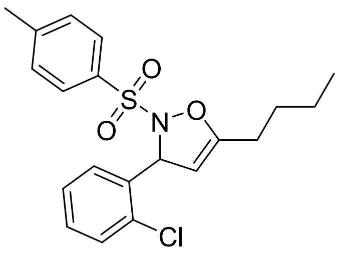

Chemical Formula: $C_{20}H_{22}ClNO_3S$
Exact Mass: 391.1009

Yield: 58% (227 mg, 0.58 mmol)

Appearance: Pale yellow oil

HR-MS (QTOF) m/z: $[M+H]^+$ Calcd for $C_{20}H_{22}ClNO_3SH^+$ 392.1082; Found 392.1084

Prepared according to General Procedure 2 and purified by flash chromatography (EtOAc:cyclohexane, 3:7, R_f = 0.58). **^{1}H NMR (400 MHz, $CDCl_3$)** δ/ppm 7.90 (d, *J* = 8.2 Hz, 2H), 7.61 (dd, *J* = 7.8, 1.6 Hz, 1H), 7.37 (d, *J* = 8.2 Hz, 2H, 7.34 – 7.19 (m, 4H), 6.14 (d, *J* = 2.0 Hz, 1H), 4.59 (m, 1H), 2.47 (s, 3H), 2.00 – 1.94 (m, 2H), 1.31 – 1.25 (m, 2H), 1.20 – 1.15 (m, 2H), 0.83 (t, *J* = 7.1 Hz, 3H). **^{13}C NMR (101 MHz, $CDCl_3$)** 155.7 (C), 145.4 (C), 138.1 (C), 131.0 (C), 130.8 (C), 129.7 (2CH), 129.5 (2CH), 129.3 (CH), 129.0 (CH), 128.1 (CH), 127.6 (CH), 96.4 (CH), 66.6 (CH), 28.3 (CH_2), 25.1 (CH_2), 22.1 (CH_2), 21.7 (CH_3), 13.7 (CH_3). **IR (neat) ν/cm^{-1}:** 2955 (w), 2932 (w), 2873 (w), 1691 (m), 1441 (m), 1358 (m), 1270 (w), 1162 (s), 1088 (m), 673 (s).

5-Butyl-3-(3-chloro-4-fluorophenyl)-2-tosyl-2,3-dihydroisoxazole, 11e

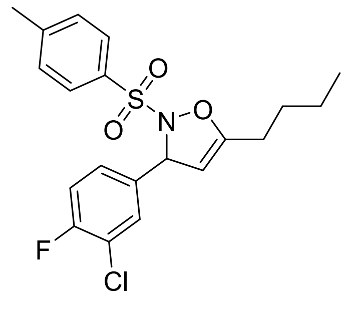

Chemical Formula: $C_{20}H_{21}ClFNO_3S$
Exact Mass: 409.0915

Yield: 65% (266 mg, 0.65 mmol)

Appearance: White solid

HR-MS (QTOF) m/z: $[M+H]^+$ Calcd for $C_{20}H_{21}ClFNO_3SH^+$ 410.0987; Found 410.0988

Prepared according to General Procedure 2 and purified by flash chromatography (EtOAc:cyclohexane, 3:7, R_f = 0.56). **^{1}H NMR (400 MHz, $CDCl_3$)** δ/ppm 7.87 (d, *J* = 8.2 Hz, 2H), 7.39 (dd, *J* = 6.8, 2.2 Hz, 1H), 7.35 (d, *J* = 8.2 Hz, 2H), 7.27 – 7.23 (m, 1H), 7.12 (t, *J* = 8.6 Hz, 1H), 5.73 (s, 1H), 4.52 – 4.51 (m, 1H), 2.46 (s, 3H), 2.14 – 1.98 (m, 2H), 1.43 – 1.32 (m, 2H), 1.29 – 1.20 (m, 2H), 0.88 (t, *J* = 7.2 Hz, 3H). **^{13}C NMR (126 MHz, $CDCl_3$)** δ/ppm 158.0 (d, *J* = 250 Hz, CF), 157.2 (C), 145.5 (C), 137.2 (d, *J* = 8 Hz, C), 130.8 (C), 129.7 (2CH), 129.5 (2CH), 129.4 (CH), 127.0 (d, *J* = 8 Hz, CH), 121.3 (d, *J* = 18 Hz, C), 116.8 (d, *J* = 22 Hz, CH), 95.2 (d, *J* = 2 Hz, CH), 68.0 (CH), 28.4 (CH_2), 25.2 (CH_2), 22.1 (CH_2), 21.7 (CH_3), 13.7 (CH_3).

IR (neat) v/cm^{-1}: 2965 (w), 2938 (w), 2878 (w), 1679 (m), 1500 (m), 1413 (w), 1248 (m), 1166 (s), 1087 (m), 682 (s).

5-Butyl-2-tosyl-3-(4-(trifluoromethyl)phenyl)-2,3-dihydroisoxazole, 11f

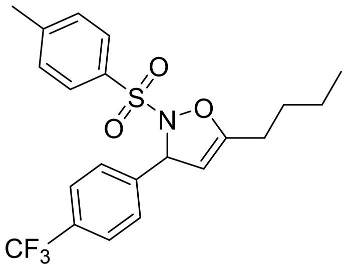

Chemical Formula: $C_{21}H_{22}F_3NO_3S$
Exact Mass: 425.1272

Yield: 60% (255 mg, 0.60 mmol)

Appearance: Pale yellow oil

HR-MS (QTOF) m/z: $[M+H]^+$ Calcd for $C_{21}H_{22}F_3NO_3SH^+$ 426.1345; Found 426.1344

Prepared according to General Procedure 2 and purified by flash chromatography (EtOAc:cyclohexane, 3:7, R_f = 0.53). **^{1}H NMR (500 MHz, CDCl$_3$)** δ/ppm 7.88 (d, *J* = 8.3 Hz, 2H), 7.62 (d, *J* = 8.3 Hz, 2H), 7.49 (d, *J* = 7.8 Hz, 2H), 7.36 (d, *J* = 7.8 Hz, 2H), 5.83 (s, 1H), 4.56 – 4.55 (m, 1H), 2.47 (s, 3H), 2.13 – 2.00 (m, 2H), 1.40 – 1.33 (m, 2H), 1.28 – 1.21 (m, 2H), 0.87 (t, *J* = 7.3 Hz, 3H). **^{13}C NMR (126 MHz, CDCl$_3$)** δ/ppm 157.2 (C), 145.7 (C), 144.2 (C), 131.0 (C), 130.6 (q, *J* = 32 Hz, C), 129.9 (2CH), 129.6 (2CH), 127.5 (2CH), 126.7 (q, *J* = 272 Hz, CF_3), 125.9 (q, *J* = 3.8 Hz, 2CH), 95.5 (CH), 68.6 (CH), 28.5 (CH_2), 25.3 (CH_2), 22.3 (CH_2), 21.9 (CH_3), 13.8 (CH_3). **^{19}F NMR (376 MHz, CDCl$_3$)** δ/ppm -62.6 (s). **IR (neat)** v/cm^{-1}: 2964 (w), 2933 (w), 2877 (w), 1686 (m), 1501 (w), 1345 (m), 1321 (s), 1154 (s), 1089 (s), 682 (s).

5-Butyl-3-phenyl-2-tosyl-2,3-dihydroisoxazole, 11g

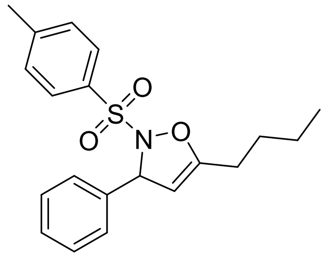

Chemical Formula: $C_{20}H_{23}NO_3S$
Exact Mass: 357.1399

Yield: 68% (243 mg, 0.68 mmol)

Appearance: Pale yellow oil

HR-MS (QTOF) m/z: $[M+Na]^+$ Calcd for $C_{20}H_{23}NO_3SNa^+$ 380.1291; Found 380.1290

Prepared according to General Procedure 2 and purified by flash chromatography (EtOAc:cyclohexane, 3:7, R_f = 0.51). **^{1}H NMR (500 MHz, CDCl$_3$)** δ/ppm 7.90 (d, *J* = 8.3 Hz, 2H), 7.39 – 7.30 (m, 7H), 5.80 – 5.79 (m, 1H), 4.56 – 4.54 (m, 1H), 2.47 (s, 3H), 2.13 – 2.00 (m, 2H), 1.43 – 1.33 (m, 2H), 1.30 – 1.23 (m, 2H), 0.89 (t, *J* = 7.3 Hz, 3H). **^{13}C NMR (126 MHz, CDCl$_3$)** δ/ppm 156.4 (C), 145.3 (C), 140.1 (C), 131.2 (C), 129.6 (2CH), 129.5 (2CH), 128.7 (2CH), 128.3 (CH), 127.1 (2CH), 96.0 (CH), 69.1 (CH), 28.4 (CH_2), 25.2 (CH_2), 22.2 (CH_2), 21.7 (CH_3), 13.7 (CH_3). **IR (neat)** v/cm^{-1}: 2958 (m), 2930 (m), 2872 (m), 1687 (m), 1494 (m), 1403 (w), 1356 (m), 1162 (s), 1088 (m), 679 (s).

5-Butyl-3-(3,4-dimethylphenyl)-2-tosyl-2,3-dihydroisoxazole, 11h

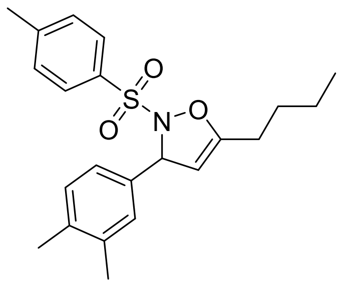

Chemical Formula: $C_{22}H_{27}NO_3S$
Exact Mass: 385.1712

Yield: 77% (297 mg, 0.77 mmol)

Appearance: Pale yellow oil

HR-MS (QTOF) m/z: $[M+H]^+$ Calcd for $C_{22}H_{27}NO_3SH^+$ 386.1784; Found 386.1785

Prepared according to General Procedure 2 and purified by flash chromatography (EtOAc:cyclohexane, 3:7, R_f = 0.58). **^{1}H NMR (500 MHz, $CDCl_3$)** δ/ppm 7.88 (d, *J* = 8.3 Hz, 2H), 7.34 (d, *J* = 7.8 Hz, 2H), 7.13 – 7.07 (m, 3H), 5.72 – 5.72 (m, 1H), 4.50 – 4.50 (m, 1H), 2.46 (s, 3H), 2.26 (s, 3H), 2.25 (s, 3H), 2.12 – 1.98 (m, 2H), 1.40 – 1.32 (m, 2H), 1.30 – 1.22 (m, 2H), 0.87 (t, *J* = 7.1 Hz, 3H). **^{13}C NMR (126 MHz, $CDCl_3$)** δ/ppm 156.1 (C), 145.1 (C), 137.5 (C), 137.0 (C), 136.8 (C), 131.2 (C), 129.9 (CH), 129.6 (2CH), 129.5 (2CH), 128.3 (CH), 124.4 (CH), 96.2 (CH), 69.1 (CH), 28.4 (CH_2), 25.2 (CH_2), 22.2 (CH_2), 21.7 (CH_3), 19.8 (CH_3), 19.5 (CH_3), 13.7 (CH_3). **IR (neat) v/cm^{-1}:** 2957 (w), 2931 (w), 2871 (w), 1687 (m), 1503 (m), 1402 (m), 1265 (w), 1166 (s), 1089 (m), 682 (s).

5-Butyl-3-(3,4-dimethoxyphenyl)-2-tosyl-2,3-dihydroisoxazole, 11i

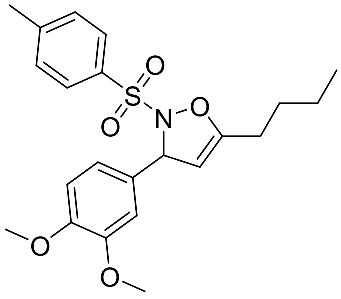

Chemical Formula: $C_{22}H_{27}NO_5S$
Exact Mass: 417.1610

Yield: 75% (313 mg, 0.75 mmol)

Appearance: Pale yellow oil

HR-MS (QTOF) m/z: $[M+H]^+$ Calcd for $C_{22}H_{27}NO_5SH^+$ 418.1682; Found 418.1683

Prepared according to General Procedure 2 and purified by flash chromatography (EtOAc:cyclohexane, 3:7, R_f = 0.28). **^{1}H NMR (400 MHz, $CDCl_3$)** δ/ppm 7.88 (d, *J* = 8.7 Hz, 2H), 7.34 (d, *J* = 8.7 Hz, 2H), 6.91 – 6.82 (m, 3H), 5.73 (d, *J* = 2.3 Hz, 1H), 4.51 (d, *J* = 2.7 Hz, 1H), 3.89 (s,3H), 3.87 (s, 3H), 2.46, (s, 3H), 2.10 – 1.98 (m, 2H), 1.40 – 1.34 (m, 2H), 1.31 – 1.24 (m, 3H), 0.87 (t, *J* = 7.3 Hz, 2H). **^{13}C NMR (101MHz, $CDCl_3$)** δ/ppm 156.3 (C), 149.2 (C), 149.1 (C), 145.2 (C), 132.6 (C), 131.1 (C), 129.6 (2CH), 129.5 (2CH), 119.3 (CH), 110.0 (CH), 110.3 (CH), 96.0 (CH), 69.1 (CH), 56.0 (CH_3), 55.9 (CH_3), 28.4 (CH_2), 25.2 (CH_2), 22.1 (CH_2), 21.7 (CH_3), 13.7 (CH_3). **IR (neat) v/cm^{-1}:** 2956 (w), 2930 (w), 2871 (w), 1687 (m), 1513 (m), 1420 (m), 1357 (s), 1164 (s), 1088 (m), 684 (s).

5-Butyl-3-(3-methylthiophen-2-yl)-2-tosyl-2,3-dihydroisoxazole, 11j

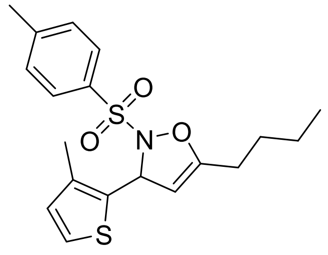

Chemical Formula: $C_{19}H_{23}NO_3S_2$
Exact Mass: 377.1119

Yield: 5% (19 mg, 0.05 mmol)

Appearance: Brown oil

HR-MS (QTOF) m/z: $[M+H]^+$ Calcd for $C_{19}H_{23}NO_3S_2H^+$ 378.1192; Found 378.1192

Prepared according to General Procedure 2 and purified by flash chromatography (EtOAc:cyclohexane, 3:7, R_f = 0.51). **^{1}H NMR (500 MHz, CDCl$_3$)** δ/ppm 7.88 (d, *J* = 8.3 Hz, 2H), 7.35 (d, *J* = 7.8 Hz, 2H), 7.18 (d, *J* = 5.4 Hz, 1H), 6.85 (d, *J* = 5.4 Hz, 1H), 6.13 (s, 1H), 4.59 – 4.58 (m, 1H), 2.46 (s, 3H), 2.39 (s, 3H), 2.13 – 2.03 (m, 2H), 1.43 – 1.37 (m, 2H), 1.34 – 1.29 (m, 2H), 0.90 (t, *J* = 7.3 Hz, 3H). **^{13}C NMR (126 MHz, CDCl$_3$)** δ/ppm 157.6 (C), 145.3 (C), 137.3 (C), 135.5 (C), 131.4 (C), 130.8 (CH), 129.6 (2CH), 129.5 (2CH), 124.7 (CH), 96.3 (CH), 62.8 (CH), 28.4 (CH_2), 25.3 (CH_2), 22.1 (CH_2), 21.7 (CH_3), 13.8 (CH_3), 13.6 (CH_3). **IR (neat)** v/cm^{-1}: 2957 (m), 2928 (m), 2871 (m), 1664 (m), 1493 (m), 1401 (m), 1337 (m), 1162 (s), 1089 (m), 673 (s).

5-Butyl-3-(naphthalen-2-yl)-2-tosyl-2,3-dihydroisoxazole, 11k

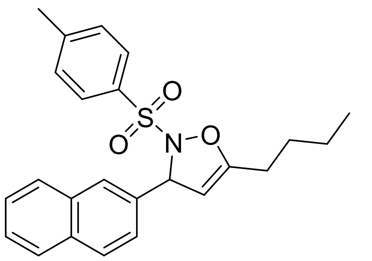

Chemical Formula: $C_{24}H_{25}NO_3S$
Exact Mass: 407.1555

Yield: 73% (298 mg, 0.73 mmol)

Appearance: Pale yellow oil

HR-MS (QTOF) m/z: $[M+H]^+$ Calcd for $C_{24}H_{25}NO_3SH^+$ 408.1628; Found 408.1628

Prepared according to General Procedure 2 and purified by flash chromatography (EtOAc:cyclohexane, 3:7, R_f = 0.47). **^{1}H NMR (500 MHz, CDCl$_3$)** δ/ppm 7.93 (d, *J* = 8.3 Hz, 2H), 7.87 – 7.82 (m, 3H), 7.80 (s, 1H), 7.52 – 7.48 (m, 3H), 7.37 (d, *J* = 8.3 Hz, 2H), 5.97 – 5.96 (m, 1H), 4.63 – 4.62 (m, 1H), 2.48 (s, 3H), 2.17 – 2.03 (m, 2H), 1.44 – 1.37 (m, 2H), 1.32 – 1.25 (m, 2H), 0.89 (t, *J* = 7.3 Hz, 3H). **^{13}C NMR (126 MHz, CDCl$_3$)** δ/ppm 156.6 (C), 145.3 (C), 137.4 (C), 133.2 (C), 133.2 (C), 131.2 (C), 129.6 (2CH), 129.5 (2CH), 128.7 (CH), 128.1 (CH), 127.7 (CH), 126.3 (CH), 126.2 (CH), 125.9 (CH), 125.0 (CH), 96.0 (CH), 69.3 (CH), 28.4 (CH_2), 25.3 (CH_2), 22.2 (CH_2), 21.7 (CH_3), 13.7 (CH_3). **IR (neat)** v/cm^{-1}: 2958 (m), 2927 (m), 2871 (m), 1714 (m), 1509 (w), 1402 (m), 1344 (m), 1156 (s), 1089 (m), 661 (s).

5-Cyclopropyl-3-(2,4-difluorophenyl)-2-tosyl-2,3-dihydroisoxazole, 11l

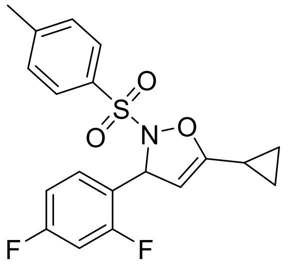

Chemical Formula: $C_{19}H_{17}F_2NO_3S$
Exact Mass: 377.0897

Yield: 81% (306 mg, 0.81 mmol)

Appearance: White solid

HR-MS (QTOF) m/z: $[M+H]^+$ Calcd for $C_{19}H_{17}F_2NO_3SH^+$ 378.0970; Found 378.0971

Prepared according to General Procedure 2 and purified by flash chromatography (EtOAc:cyclohexane, 3:7, R_f = 0.50). **^{1}H NMR (400 MHz, $CDCl_3$)** δ/ppm 7.88 (d, *J* = 8.2 Hz, 2H), 7.44 (td, *J* = 8.6, 6.3 Hz, 1H), 7.37 (d, *J* = 8.2 Hz, 2H), 6.89 (td, *J* = 8.8, 3.3 Hz, 1H), 6.79 (ddd, *J* = 11.0, 9.0, 2.7 Hz, 1H), 6.00 (d, *J* = 2.3 Hz, 1H), 4.47 (d, *J* = 2.7 Hz, 1H), 2.48 (s, 3H), 1.35 – 1.28 (m, 1H), 0.76 – 0.69 (m, 2H), 0.62 – 0.56 (m, 1H), 0.51 – 0.45 (m, 1H). **^{13}C NMR (151 MHz, $CDCl_3$)** δ/ppm 162.8 (dd, *J* = 250, 12 Hz, CF), 159.7 (dd, *J* = 250, 13 Hz, CF), 157.7 (C), 145.6 (C), 130.0 (C), 129.9 (2CH), 129.7 (2CH), 129.6 (dd, *J* = 10, 5 Hz, CH), 123.9 (dd, *J* = 13.3, 4.0 Hz, C), 111.9 (dd, *J* = 21.4, 4.0 Hz, CH), 103.9 (dd, *J* = 25, 25 Hz, CH), 94.4 (CH), 63.2 (d, *J* = 4 Hz, CH), 21.9 (CH_3), 6.8 (CH), 6.3 (CH_2), 6.0 (CH_2). **^{19}F NMR (376 MHz, $CDCl_3$)** δ/ppm -110.5 (m), -115.5 (m). **IR (neat)** ν/cm^{-1}: 3080 (w), 3012 (w), 2923 (w), 1686 (m), 1502 (s), 1429 (m), 1355 (m), 1272 (m), 1168 (s), 1089 (s), 682 (m).

3-(4-Bromophenyl)-5-cyclopropyl-2-tosyl-2,3-dihydroisoxazole, 11m

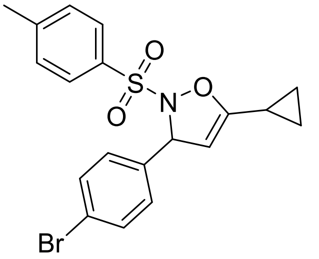

Chemical Formula: $C_{19}H_{18}BrNO_3S$
Exact Mass: 419.0191

Yield: 72% (303 mg, 0.72 mmol)

Appearance: White solid

HR-MS (QTOF) m/z: $[M+H]^+$ Calcd for $C_{19}H_{18}BrNO_3SH^+$ 420.0264; Found 420.0263

Prepared according to General Procedure 2 and purified by flash chromatography (EtOAc:cyclohexane, 3:7, R_f = 0.47). **^{1}H-NMR (500 MHz, $CDCl_3$)** δ/ppm 7.86 (d, *J* = 8.3 Hz, 2H), 7.48 – 7.46 (m, 2H), 7.36 (d, *J* = 7.8 Hz, 2H), 7.24 – 7.21 (m, 2H), 5.73 (d, *J* = 2.4 Hz, 1H), 4.49 (d, *J* = 2.9 Hz, 1H), 2.47 (s, 3H), 1.36 – 1.33 (m, 1H), 0.79 – 0.70 (m, 2H), 0.64 – 0.60 (m, 1H), 0.54 – 0.49 (m, 1H). **^{13}C-NMR (126 MHz, $CDCl_3$)** δ/ppm 158.0 (C), 145.4 (C), 139.2 (C), 131.8 (2CH), 131.0 (C), 129.7 (2CH), 129.5 (2CH), 128.8 (2CH), 122.3 (C), 93.9 (CH), 68.6 (CH), 21.8 (CH_3), 6.5 (CH), 6.3 (CH_2), 5.9 (CH_2). **IR (neat) ν/cm^{-1}:** 3015 (w), 2919 (w), 1682 (m), 1404 (m), 1356 (m), 1279 (m), 1163 (s), 1024 (m), 687 (s), 558 (s).

5-Cyclopropyl-3-(naphthalen-2-yl)-2-tosyl-2,3-dihydroisoxazole, 11n

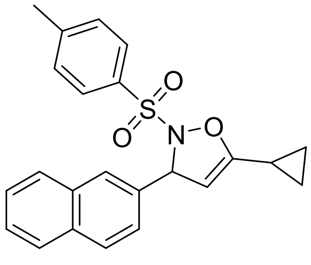

Chemical Formula: $C_{23}H_{21}NO_3S$
Exact Mass: 391.1242

Yield: 80% (313 mg, 0.80 mmol)

Appearance: White solid

HR-MS (QTOF) m/z: $[M+H]^+$ Calcd for $C_{23}H_{21}NO_3SH^+$ 392.1315; Found 392.1316

Prepared according to General Procedure 2 and purified by flash chromatography (EtOAc:cyclohexane, 3:7, R_f = 0.46). **^{1}H NMR (500 MHz, $CDCl_3$)** δ/ppm 7.92 (d, *J* = 8.3 Hz, 2H), 7.86 – 7.82 (m, 3H), 7.79 (s, 1H), 7.50 – 7.46 (m, 3H), 7.37 (d, *J* = 8.3 Hz, 2H), 5.96 (d, *J* = 2.4 Hz, 1H), 4.60 (d, *J* = 2.9 Hz, 1H), 2.47 (s, 3H), 1.44 – 1.37 (m, 1H), 0.81 – 0.73 (m, 2H), 0.69 – 0.64 (m, 1H), 0.58 – 0.53 (m, 1H). **^{13}C NMR (151 MHz, $CDCl_3$)** δ/ppm 157.9 (C), 145.5 (C), 137.5 (C), 133.4 (C), 133.3 (C), 131.4 (C), 129.8 (2CH), 129.7 (2CH), 128.9 (CH), 128.3 (CH), 127.8 (CH), 126.4 (CH), 126.4 (CH), 126.0 (CH), 125.1 (CH), 94.7 (CH), 69.5 (CH), 21.9 (CH_3), 6.7 (CH), 6.4 (CH_2), 6.0 (CH_2). **IR (neat)** ν/cm^{-1}: 2942 (m), 2923 (m), 2852 (m), 1668 (m), 1506 (m), 1406 (m), 1368 (s), 1169 (s), 1077 (m), 671 (s).

3-(2,4-Difluorophenyl)-5-phenyl-2-tosyl-2,3-dihydroisoxazole, 11o

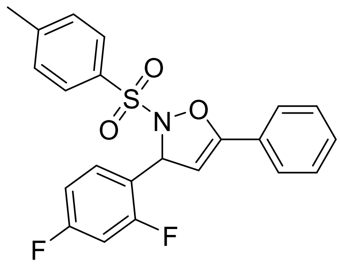

Chemical Formula: $C_{22}H_{17}F_2NO_3S$
Exact Mass: 413.0897

Yield: 80% (330 mg, 0.80 mmol)

Appearance: Pale yellow oil

HR-MS (QTOF) m/z: $[M+H]^+$ Calcd for $C_{22}H_{17}F_2NO_3SH^+$ 414.0970; Found 414.0969

Prepared according to General Procedure 2 and purified by flash chromatography (EtOAc:cyclohexane, 3:7, R_f = 0.40). **^{1}H NMR (500 MHz, $CDCl_3$)** δ/ppm 7.88 (d, *J* = 8.3 Hz, 2H), 7.53 (td, *J* = 8.6, 6.4 Hz, 1H), 7.41 – 7.39 (m, 2H), 7.36 – 7.32 (m, 3H), 7.22 (d, *J* = 7.8 Hz, 2H), 6.90 – 6.87 (m, 1H), 6.83 (ddd, *J* = 11.2, 8.8, 2.4 Hz, 1H), 6.19 (d, *J* = 2.9 Hz, 1H), 5.16 (dd, *J* = 2.9, 1.0 Hz, 1H), 2.37 (s, 3H). **^{13}C NMR (126 MHz, $CDCl_3$)** δ/ppm 162.8 (dd, *J* = 249, 12 Hz, CF), 159.5 (dd, *J* = 249, 12 Hz, CF), 153.6 (C), 145.6 (C), 130.5 (C), 129.9 (CH), 129.7 (2CH), 129.6 (dd, *J* = 10, 5 Hz, CH), 129.3 (2CH), 128.5 (2CH), 126.5 (C), 125.6 (2CH), 123.1 (dd, *J* = 13, 4 Hz, C), 111.9 (dd, *J* = 21, 4 Hz, CH), 103.9 (dd, *J* = 25, 25 Hz, CH), 95.7 (d, *J* = 2 Hz, CH), 63.5 (CH), 21.7 (CH_3). **^{19}F NMR (376 MHz, $CDCl_3$)** δ/ppm -110.0 (m), -115.2 (m). **IR (neat)** ν/cm^{-1}: 3060 (w), 3027 (w), 2917 (w), 1666 (w), 1500 (m), 1433 (m), 1265 (w), 1170 (s), 1088 (m), 678 (s).

3-(4-Fluorophenyl)-5-phenyl-2-tosyl-2,3-dihydroisoxazole, 11p

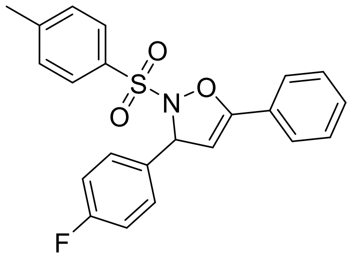

Chemical Formula: $C_{22}H_{18}FNO_3S$
Exact Mass: 395.0991

Yield: 76% (300 mg, 0.76 mmol)

Appearance: White solid

HR-MS (QTOF) m/z: $[M+H]^+$ Calcd for $C_{22}H_{18}FNO_3SH^+$ 396.1064; Found 396.1065

Prepared according to General Procedure 2 and purified by flash chromatography (EtOAc:cyclohexane, 3:7, R_f = 0.38). **^{1}H NMR (500 MHz, CDCl$_3$)** δ/ppm 7.87 (d, *J* = 8.3 Hz, 2H), 7.44 – 7.33 (m, 7H), 7.22 (d, *J* = 7.8 Hz, 2H), 7.07 – 7.03 (m, 2H), 5.94 (d, *J* = 2.9 Hz, 1H), 5.18 (d, *J* = 2.9 Hz, 1H), 2.37 (s, 3H). **^{13}C NMR (126 MHz, CDCl$_3$)** δ/ppm 162.8 (d, *J* = 247 Hz, CF), 153.9 (C), 145.5 (C), 135.4 (d, *J* = 4 Hz, C), 130.7 (C), 129.9 (CH), 129.7 (2CH), 129.3 (2CH), 129.1 (d, *J* = 9 Hz, 2CH), 128.5 (2CH), 126.7 (C), 125.6 (2CH), 115.7 (d, *J* = 22 Hz, 2CH), 95.8 (d, *J* = 3 Hz, CH), 69.1 (CH), 21.7 (CH_3). **^{19}F-NMR (376 MHz, CDCl$_3$)** δ/ppm -113.6 (m). **IR (neat)** ν/cm^{-1}: 2972 (w), 2925 (w), 1688 (m), 1509 (s), 1448 (m), 1383 (s), 1232 (s), 1178 (s), 1088 (m), 691 (m).

3-(4-Bromophenyl)-5-phenyl-2-tosyl-2,3-dihydroisoxazole, 11q

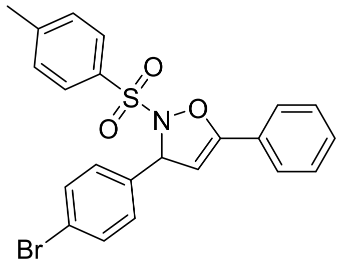

Chemical Formula: $C_{22}H_{18}BrNO_3S$
Exact Mass: 455.0191

Yield: 74% (338 mg, 0.74 mmol)

Appearance: Pale yellow oil

HR-MS (QTOF) m/z: $[M+H]^+$ Calcd for $C_{22}H_{18}BrNO_3SH^+$ 456.0264; Found 456.0263

Prepared according to General Procedure 2 and purified by flash chromatography (EtOAc:cyclohexane, 3:7, R_f = 0.42). **^{1}H NMR (500 MHz, CDCl$_3$)** δ/ppm 7.87 (d, *J* = 8.3 Hz, 2H), 7.51 – 7.48 (m, 2H), 7.43 (dd, *J* = 8.1, 1.7 Hz, 2H), 7.38 – 7.33 (m, 3H), 7.31 – 7.29 (m, 2H), 7.22 (d, *J* = 7.8 Hz, 2H), 5.91 (d, *J* = 2.9 Hz, 1H), 5.17 (d, *J* = 2.4 Hz, 1H), 2.37 (s, 3H). **^{13}C NMR (101 MHz, CDCl$_3$)** δ/ppm 154.2 (C), 145.7 (C), 138.7 (C), 132.1 (2CH), 130.9 (C), 130.1 (CH), 129.8 (2CH), 129.5 (2CH), 129.1 (2CH), 128.7 (2CH), 126.8 (C), 125.8 (2CH), 122.8 (C), 95.7 (CH), 69.3 (CH), 21.8 (CH_3). **IR (neat)** ν/cm^{-1}: 3116 (w), 3064 (w), 2922 (w), 1485 (m), 1360 (m), 1167 (s), 1071 (m), 1010 (m), 686 (s), 568 (s).

3-(Naphthalen-2-yl)-5-phenyl-2-tosyl-2,3-dihydroisoxazole, 11r

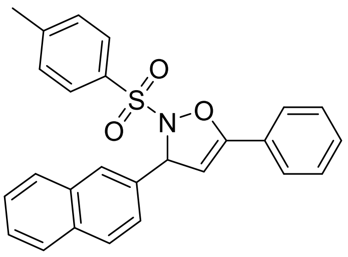

Chemical Formula: $C_{26}H_{21}NO_3S$
Exact Mass: 427.1242

Yield: 49% (209 mg, 0.49 mmol)

Appearance: White solid

HR-MS (QTOF) m/z: $[M+H]^+$ Calcd for $C_{26}H_{21}NO_3SH^+$ 428.1315; Found 428.1316

Prepared according to General Procedure 2 and purified by flash chromatography (EtOAc:cyclohexane, 3:7, R_f = 0.39). **^{1}H NMR (500 MHz, $CDCl_3$)** δ/ppm 7.92 (d, *J* = 8.3 Hz, 2H), 7.87 – 7.81 (m, 4H), 7.56 (dd, *J* = 8.6, 1.7 Hz, 1H), 7.50 – 7.46 (m, 4H), 7.40 – 7.34 (m, 3H), 7.23 (d, *J* = 8.3 Hz, 2H), 6.13 (d, *J* = 2.9 Hz, 1H), 5.29 (d, *J* = 2.9 Hz, 1H), 2.37 (s, 3H). **^{13}C NMR (126 MHz, $CDCl_3$)** δ/ppm 153.8 (C), 145.4 (C), 136.8 (C), 133.3 (C), 133.2 (C), 130.9 (C), 129.8 (CH), 129.7 (2CH), 129.4 (2CH), 128.9 (CH), 128.5 (2CH), 128.2 (CH), 127.7 (CH), 126.9 (C), 126.4 (2CH), 126.1 (CH), 125.7 (2CH), 125.0 (CH), 96.2 (CH), 70.0 (CH), 21.7 (CH_3). **IR (neat)** ν/cm^{-1}: 2966 (w), 2928 (w), 1922 (w), 1667 (m), 1493 (m), 1356 (m), 1276 (w), 1169 (s), 1089 (m), 682 (s).

4.4 Aziridines

1-((2*R**,3*R**)-3-(2,4-Difluorophenyl)-1-tosylaziridin-2-yl)pentan-1-one, 12a

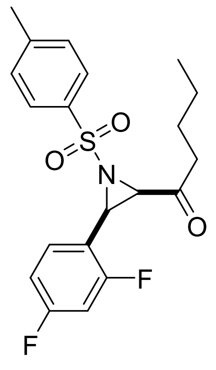

Chemical Formula: $C_{20}H_{21}F_2NO_3S$
Exact Mass: 393.1210

Yield: 82% (0.123 mg, 0.31 mmol)

Appearance: Pale yellow oil

HR-MS (QTOF) m/z: $[M+H]^+$ Calcd for $C_{20}H_{21}F_2NO_3SH^+$ 394.1283; Found 394.1285

Prepared according to General Procedure 4 and purified by flash chromatography (EtOAc:cyclohexane, 3:7, R_f = 0.53). **^{1}H-NMR (400 MHz, $CDCl_3$)** δ/ppm 7.91 (d, *J* = 8.2 Hz, 2H), 7.38 (d, *J* = 7.9 Hz, 2H), 7.27 – 7.21 (m, 1H), 6.81 – 6.73 (m, 2H), 4.17 (d, *J* = 7.9 Hz, 1H), 3.74 (d, *J* = 7.9, 1H), 2.46 (s, 3H), 2.30 – 2.11 (m, 2H), 1.35 – 1.24 (m, 2H), 1.03 (sext, *J* = 7.4 Hz, 2H), 0.73 (t, *J* = 7.4 Hz, 3H). **^{13}C-NMR (101MHz, $CDCl_3$)** δ/ppm 200.8 (C), 163.0 (dd, *J* = 251, 12 Hz, CF), 161.2 (dd, *J* = 251, 12 Hz, CF), 145.5 (C), 133.7 (C), 130.5 (dd, *J* = 10, 5 Hz, CH), 130.0 (2CH), 128.2 (2CH), 115.0 (dd, *J* = 14, 4 Hz, C), 111.4 (dd, *J* = 10, 5 Hz, CH), 103.8 (dd, *J* = 26, 24 Hz, CH), 47.8 (CH), 41.4 (CH_2), 40.9 (d, *J* = 4 Hz, CH), 24.8 (CH_2),

21.9 (CH_2), 21.7 (CH_3), 13.5 (CH_3). **^{19}F-NMR (376 MHz, $CDCl_3$)** δ/ppm -113.4 (m), -108.5 (m). **IR (neat) v/cm^{-1}:** 2958 (w), 2929 (w), 2872 (w), 1718 (m), 1598 (m), 1507 (s), 1334 (s), 1162 (s), 1091 (s), 816 (m).

1-((2*R,3*R**)-3-(4-Fluorophenyl)-1-tosylaziridin-2-yl)pentan-1-one, 12b**

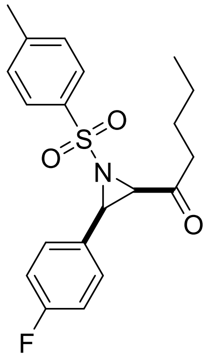

Chemical Formula: $C_{20}H_{22}FNO_3S$
Exact Mass: 375.1304

Yield: 68% (204 mg, 0.54 mmol)

Appearance: Pale yellow solid

HR-MS (QTOF) m/z: $[M+H]^+$ Calcd for $C_{20}H_{22}FNO_3SH^+$ 376.1377; Found 376.1378

Melting point: 77 – 78 ^{0}C

Prepared according to General Procedure 4 and purified by flash chromatography (EtOAc:cyclohexane, 3:7, R_f = 0.45). **^{1}H NMR (500 MHz, $CDCl_3$)** δ/ppm 7.91 (d, *J* = 8.3 Hz, 2H), 7.38 (d, *J* = 7.8 Hz, 2H), 7.22 – 7.19 (m, 2H), 6.98 – 6.94 (m, 2H), 4.11 (d, *J* = 7.8 Hz, 1H), 3.62 (d, *J* = 7.8 Hz, 1H), 2.46 (s, 4H), 2.17 – 2.10 (m, 1H), 1.99 – 1.93 (m, 1H), 1.26 – 1.15 (m, 2H), 1.00 – 0.93 (m, 2H), 0.68 (t, *J* = 7.3 Hz, 3H). **^{13}C NMR (126 MHz, $CDCl_3$)** δ/ppm 202.4 (C), 162.9 (d, *J* = 248.0 Hz, CF), 145.6 (C), 134.0 (C), 130.2 (2CH), 129.4 (d, *J* = 8.6 Hz, 2CH), 128.3 (2CH), 127.4 (d, *J* = 3.8 Hz, C), 115.8 (d, *J* = 21.9 Hz, 2CH), 48.9 (CH), 45.2 (CH), 41.0 (CH_2), 24.8 (CH_2), 22.0 (CH_2), 21.9 (CH_3), 13.7 (CH_3). **^{19}F NMR (376 MHz, $CDCl_3$)** δ/ppm -112.7 (m). **IR (neat) v/cm^{-1}:** 2960 (m), 2931 (m), 2874 (m), 1725 (m), 1596 (m), 1511 (s), 1337 (s), 1156 (s), 1086 (m), 820 (s).

1-((2*R,3*R**)-3-(4-Bromophenyl)-1-tosylaziridin-2-yl)pentan-1-one, 12c**

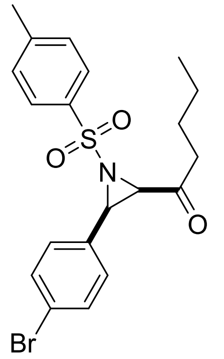

Chemical Formula: $C_{20}H_{22}BrNO_3S$
Exact Mass: 435.0504

Yield: 80% (240 mg, 0.55 mmol)

Appearance: Pale yellow oil

HR-MS (QTOF) m/z: [M+H]+ Calcd for $C_{20}H_{22}BrNO_3SH^+$ 436.0577; Found 436.0578

Prepared according to General Procedure 4 and purified by flash chromatography (EtOAc:cyclohexane, 3:7, R_f = 0.48). **^{1}H-NMR (400 MHz, $CDCl_3$)** δ/ppm 7.90 (d, *J* = 8.3 Hz, 2H) 7.40 – 7.36 (m, 4H), 7.10 (d, *J* = 8.3, 2H), 4.07 (d, *J* = 7.9 Hz, 1H), 3.65 (d, *J* = 7.9 Hz, 1H), 2.45 (s, 3H), 2.14 (ddd, *J* = 17.4, 8.2, 6.3 Hz, 1H), 1.98 (ddd, *J* = 17.4, 8.2, 6.3 Hz, 1H), 1.30 – 1.15 (m, 2H), 1.03 – 0.92 (m, 2H), 0.68 (t, *J* = 7.4 Hz, 3H). **^{13}C-NMR (101 MHz, $CDCl_3$)**

δ/ppm 201.9 (C), 145.5 (C), 133.8 (C), 131.7 (2CH), 130.4 (C), 130.0 (2CH), 129.1 (2CH), 128.2 (2CH), 122.8 (C), 48.7 (CH), 45.1 (CH), 40.9 (CH_2), 24.7 (CH_2), 21.8 (CH_2), 21.7 (CH_3), 13.5 (CH_3). **IR (neat) ν/cm^{-1}:** 2957 (w), 2930 (w), 2871 (w), 1713 (m), 1596 (m), 1490 (m), 1455 (m), 1331 (s), 1091 (m), 678 (m).

1-((2*R,3*R**)-3-(2-Chlorophenyl)-1-tosylaziridin-2-yl)pentan-1-one, 12d**

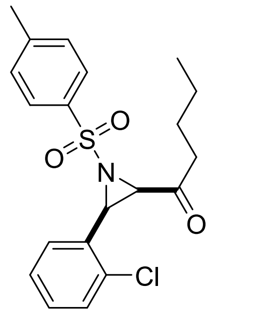

Chemical Formula: $C_{20}H_{22}ClNO_3S$
Exact Mass: 391.1009

Yield: 81% (243 mg, 0.62 mmol)

Appearance: Pale yellow oil

HR-MS (QTOF) m/z: $[M+H]^+$ Calcd for $C_{20}H_{22}ClNO_3SH^+$ 392.1082; Found 392.1086

Prepared according to General Procedure 4 and purified by flash chromatography (EtOAc:cyclohexane, 3:7, R_f = 0.59). **^{1}H NMR (500 MHz, $CDCl_3$)** δ/ppm 7.93 (d, *J* = 8.3 Hz, 2H), 7.38 (d, *J* = 7.8 Hz, 2H), 7.31 (ddd, *J* = 13.2, 7.8, 1.5 Hz, 2H), 7.24 – 7.16 (m, 2H), 4.25 (d, *J* = 7.8 Hz, 1H), 3.79 (d, *J* = 7.8 Hz, 1H), 2.45 (s, 3H), 2.25 (ddd, *J* = 17.6, 7.8, 6.4 Hz, 1H), 2.06 (ddd, *J* = 17.1, 7.8, 6.6 Hz, 1H), 1.30 – 1.20 (m, 2H), 1.01 – 0.88 (m, 2H), 0.68 (t, *J* = 7.3 Hz, 3H). **^{13}C NMR (101 MHz, $CDCl_3$) δ/ppm** 201.0 (C), 145.5 (C), 134.0 (C), 133.6 (C), 130.1 (2CH), 129.9 (CH), 129.7 (CH), 129.6 (CH), 129.3 (CH), 128.3 (2CH), 126.9 (C), 48.2 (CH), 44.8 (CH), 40.9 (CH_2), 24.9 (CH_2), 21.9 (CH_2), 21.8 (CH_3), 13.7 (CH_3). **IR (neat) ν/cm^{-1}:** 2957 (w), 2930 (w), 2871 (w), 1714 (m), 1596 (m), 1494 (m), 1333 (s), 1162 (s), 1091 (m), 759 (m).

1-((2*R,3*R**)-3-(3-Chloro-4-fluorophenyl)-1-tosylaziridin-2-yl)pentan-1-one, 12e**

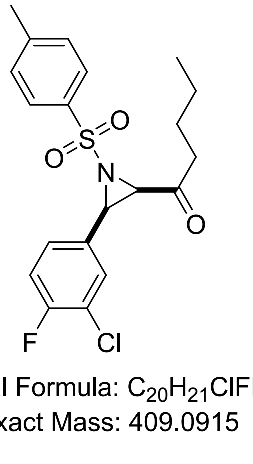

Chemical Formula: $C_{20}H_{21}ClFNO_3S$
Exact Mass: 409.0915

Yield: 82% (246 mg, 0.60 mmol)

Appearance: White solid

HR-MS (QTOF) m/z: $[M+Na]^+$ Calcd for Na^+ 432.0807; Found 432.0807

Prepared according to General Procedure 4 and purified by flash chromatography (EtOAc:cyclohexane, 3:7, R_f = 0.51). **^{1}H NMR (500 MHz, $CDCl_3$)** δ/ppm 7.91 (d, *J* = 8.3 Hz, 2H), 7.39 (d, *J* = 8.3 Hz, 2H), 7.28 (dd, *J* = 6.8, 2.0 Hz, 1H), 7.13 – 7.10 (m, 1H), 7.04 (t, *J* = 8.6 Hz, 1H), 4.06 (d, *J* = 7.8 Hz, 1H), 3.63 (d, *J* = 7.8 Hz, 1H), 2.46 (s, 3H), 2.19 – 2.13 (m, 1H), 2.06 – 2.00 (m, 1H), 1.30 – 1.20 (m, 2H), 0.99 (h, *J* = 7.1 Hz, 2H), 0.70 (t, *J* = 7.3 Hz, 3H). **^{13}C NMR (126 MHz, $CDCl_3$)** δ/ppm 201.7 (C), 158.2 (d, *J* = 251 Hz, CF), 145.7 (C), 133.8

(C), 130.2 (2CH), 130.0 (CH), 128.7 (d, *J* = 5 Hz, C), 128.3 (2CH), 127.5 (d, *J* = 8 Hz, CH), 121.5 (d, *J* = 18 Hz, C), 116.9 (d, *J* = 22 Hz, CH), 48.9 (CH), 44.5 (CH), 41.2 (CH_2), 24.8 (CH_2), 22.0 (CH_2), 21.9 (CH_3), 13.6 (CH_3). **^{19}F NMR (376 MHz, $CDCl_3$)** δ/ppm -114.7 (m). **IR (neat) v/cm^{-1}:** 2958 (m), 2930 (m), 2872 (w), 1719 (m), 1596 (m), 1502 (m), 1331 (m), 1162 (s), 1087 (m), 673 (s).

1-((2*R,3*R**)-1-Tosyl-3-(4-(trifluoromethyl)phenyl)aziridin-2-yl)pentan-1-one, 12f**

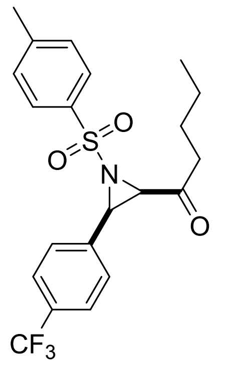

Chemical Formula: $C_{21}H_{22}F_3NO_3S$
Exact Mass: 425.1272

Yield: 67% (201 mg, 0.47 mmol)

Appearance: Pale yellow oil

HR-MS (QTOF) m/z: $[M+Na]^+$ Calcd for $C_{21}H_{22}F_3NO_3SNa^+$ 448.1165; Found 448.1163

Prepared according to General Procedure 4 and purified by flash chromatography (EtOAc:cyclohexane, 3:7, R_f = 0.40). **^{1}H NMR (400 MHz, $CDCl_3$)** δ/ppm 7.92 (d, *J* = 8.3 Hz, 2H), 7.54 (d, *J* = 8.2 Hz, 2H), 7.39 (d, *J* = 8.0 Hz, 2H), 7.36 (d, *J* = 8.7 Hz, 2H), 4.16 (d, *J* = 7.9 Hz, 1H), 3.70 (d, *J* = 7.9 Hz, 1H), 2.47 (s, 3H), 2.15 (ddd, *J* = 17.5, 8.0, 6.4 Hz, 1H), 2.00 (ddd, *J* = 17.4, 7.9, 6.6 Hz, 1H), 1.29 – 1.15 (m, 2H), 0.95 (s, *J* = 7.2 Hz, 2H), 0.66 (t, *J* = 7.3 Hz, 3H). **^{13}C NMR (101 MHz, $CDCl_3$)** δ/ppm 201.8 (C), 145.8 (C), 135.6 (C), 133.9 (C), 131.0 (q, *J* = 33 Hz, C), 130.2 (2CH), 128.4 (2CH), 128.1 (2CH), 126.6 (q, *J* = 272 Hz, CF_3), 125.7 (q, *J* = 4 Hz, 2CH), 48.8 (CH), 45.2 (CH), 41.1 (CH_2), 24.8 (CH_2), 21.9 (CH_2), 21.9 (CH_3), 13.6 (CH_3). **^{19}F NMR (376 MHz, $CDCl_3$)** δ/ppm -62.8 (s). **IR (neat) v/cm^{-1}:** 2962 (w), 2935 (w), 2877 (w), 1726 (m), 1595 (w), 1397 (m), 1322 (s), 1154 (s), 1067 (s), 808 (m).

1-((2*R,3*R**)-3-Phenyl-1-tosylaziridin-2-yl)pentan-1-one, 12g**

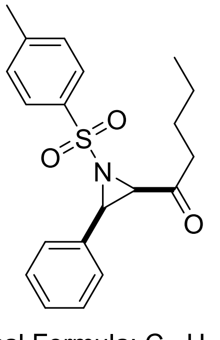

Chemical Formula: $C_{20}H_{23}NO_3S$
Exact Mass: 357.1399

Yield: 84% (252 mg, 0.71 mmol)

Appearance: White solid

HR-MS (QTOF) m/z: $[M+H]^+$ Calcd for $C_{20}H_{23}NO_3SH^+$ 358.1471; Found 358.1471

Melting point: 88 – 89 ^{0}C

Prepared according to General Procedure 4 and purified by flash chromatography (EtOAc:cyclohexane, 3:7, R_f = 0.51). **^{1}H NMR (500 MHz, $CDCl_3$)** δ/ppm 7.92 (d, *J* = 8.3 Hz, 2H), 7.38 (d, *J* = 7.8 Hz, 2H), 7.27 – 7.25 (m, 3H), 7.22 – 7.20 (m, 2H), 4.14 (d, *J* = 7.8 Hz, 1H), 3.63 (d, *J* = 7.8 Hz, 1H), 2.46 (s, 3H), 2.13 (ddd, *J* = 17.6, 8.1, 6.4 Hz, 1H), 1.92 (ddd, *J* = 17.6, 8.1, 6.6 Hz, 1H), 1.27 – 1.11 (m, 2H), 0.98 – 0.87 (m, 2H), 0.65 (t, *J* = 7.3 Hz, 3H). **^{13}C**

NMR (126 MHz, $CDCl_3$) δ/ppm 202.8 (C), 145.5 (C), 134.1 (C), 131.5 (C), 130.1 (2CH), 128.7 (CH), 128.7 (2CH), 128.4 (2CH), 127.6 (2CH), 48.9 (CH), 45.9 (CH), 40.8 (CH_2), 24.8 (CH_2), 21.9 (CH_2), 21.9 (CH_3), 13.7 (CH_3). **IR (neat) v/cm^{-1}:** 2957 (m), 2928 (m), 2872 (m), 1724 (m), 1595 (m), 1497 (m), 1339 (s), 1154 (s), 1086 (m), 822 (m).

1-((2*R,3*R**)-3-(3,4-Dimethylphenyl)-1-tosylaziridin-2-yl)pentan-1-one, 12h**

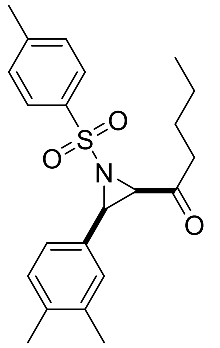

Chemical Formula: $C_{22}H_{27}NO_3S$
Exact Mass: 385.1712

Yield: 82% (246 mg, 0.64 mmol)

Appearance: Pale yellow oil

HR-MS (QTOF) m/z: $[M+Na]^+$ Calcd for $C_{22}H_{27}NO_3SNa^+$ 408.1604; Found 408.1606

Prepared according to General Procedure 4 and purified by flash chromatography (EtOAc:cyclohexane, 3:7, R_f = 0.54). **^{1}H NMR (600 MHz, $CDCl_3$)** δ/ppm 7.92 (d, *J* = 8.2 Hz, 2H), 7.38 (d, *J* = 8.2 Hz, 2H), 7.01 (d, *J* = 7.6 Hz, 1H), 6.95 (s, 1H), 6.91 (d, *J* = 7.6 Hz, 1H), 4.07 (d, *J* = 8.2 Hz, 1H), 3.58 (d, *J* = 8.2 Hz, 1H), 2.46 (s, 3H), 2.19 (s, 3H), 2.17 (s, 3H), 2.15 – 2.11 (m, 1H), 1.95 (ddd, *J* = 17.6, 8.2, 6.5 Hz, 1H), 1.27 – 1.15 (m, 2H), 1.00 – 0.93 (m, 2H), 0.66 (t, *J* = 7.3 Hz, 3H). **^{13}C NMR (151 MHz, $CDCl_3$)** δ/ppm 203.1 (C), 145.4 (C), 137.2 (C), 137.0 (C), 134.2 (C), 130.1 (2CH), 129.8 (CH), 128.8 (C), 128.7 (CH), 128.4 (2CH), 124.9 (CH), 49.0 (CH), 45.9 (CH), 40.8 (CH_2), 24.8 (CH_2), 22.0 (CH_2), 21.9 (CH_3), 19.8 (CH_3), 19.6 (CH_3), 13.7 (CH_3). **IR (neat)** v/cm^{-1}: 2957 (w), 2928 (w), 2870 (w), 1720 (m), 1598 (m), 1497 (w), 1334 (m), 1157 (s), 1090 (m), 813 (m).

1-((2*R,3*R**)-3-(3,4-Dimethoxyphenyl)-1-tosylaziridin-2-yl)pentan-1-one, 12i**

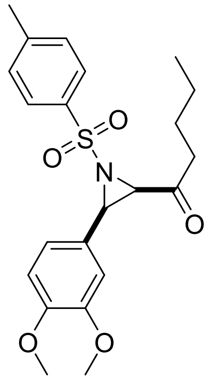

Chemical Formula: $C_{22}H_{27}NO_5S$
Exact Mass: 417.1610

Yield: 60% (251 mg, 0.61 mmol)

Appearance: Yellow oil

HR-MS (QTOF) m/z: $[M+H]^+$ Calcd for $C_{22}H_{27}NO_5SH^+$ 418.1683; Found 418.1683

Prepared according to General Procedure 4 and purified by flash chromatography (EtOAc:cyclohexane, 3:7, R_f = 0.23). **^{1}H NMR (500 MHz, $CDCl_3$)** δ/ppm 7.93 (d, *J* = 8.3 Hz, 2H), 7.38 (d, *J* = 8.3 Hz, 2H), 6.78 – 6.73 (m, 2H), 6.65 (d, *J* = 2.0 Hz, 1H), 4.07 (d, *J* = 7.8

Hz, 1H), 3.83 (s, 3H), 3.77 (s, 3H), 3.61 (d, *J* = 7.8 Hz, 1H), 2.46 (s, 3H), 2.17 (dd, *J* = 17.6 8.1, 6.1 Hz, 1H), 2.00 – 1.93 (m, 1H), 1.27 – 1.20 (m, 2H), 1.03 – 0.96 (m, 2H), 0.68 (t, *J* = 7.3 Hz, 3H). **^{13}C NMR (126 MHz, CDCl$_3$)** δ/ppm 203.0 (C), 149.3 (C), 149.0 (C), 145.4 (C), 134.2 (C), 130.1 (2CH), 128.4 (2CH), 123.9 (C), 120.1 (CH), 111.1 (CH), 110.4 (CH), 56.0 (CH$_3$), 56.0 (CH$_3$), 49.0 (CH), 45.8 (CH), 40.9 (CH$_2$), 24.8 (CH$_2$), 22.0 (CH$_2$), 21.9 (CH$_3$), 13.7 (CH$_3$). **IR (neat) v/cm^{-1}:** 2956 (m), 2932 (m), 2871 (m), 1715 (m), 1595 (m), 1513 (s), 1337 (m), 1161 (s), 1089 (m), 813 (m).

1-((2*R,3*R**)-3-(Naphthalen-2-yl)-1-tosylaziridin-2-yl)pentan-1-one, 12k**

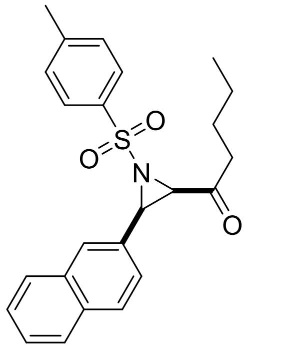

Chemical Formula: $C_{24}H_{25}NO_3S$
Exact Mass: 407.1555

Yield: 94% (282 mg, 0.74 mmol)

Appearance: Pale yellow oil

HR-MS (QTOF) m/z: [M+H]$^+$ Calcd for $C_{24}H_{25}NO_3SH^+$ 408.1628; found 408.1628.

Prepared according to General Procedure 4 and purified by flash chromatography (EtOAc:cyclohexane, 3:7, R_f = 0.42). **^{1}H NMR (500 MHz, CDCl$_3$)** δ/ppm 7.96 (d, *J* = 8.3 Hz, 2H), 7.79 – 7.74 (m, 3H), 7.70 (s, 1H), 7.48 – 7.46 (m, 2H), 7.40 (d, *J* = 8.3 Hz, 2H), 7.31 (dd, *J* = 8.6, 1.7 Hz, 1H), 4.29 (d, *J* = 8.3 Hz, 1H), 3.70 (d, *J* = 7.9 Hz, 1H), 2.47 (s, 3H), 2.16 – 2.11 (m, 1H), 1.97 – 1.91 (m, 1H), 1.22 – 1.10 (m, 2H), 0.90 – 0.85 (m, 2H), 0.56 (t, *J* = 7.3 Hz, 3H). Compound too unstable on silica to obtain ^{13}C NMR. **IR (neat) v/cm^{-1}:** 3054 (w), 3009 (w), 2863 (w), 1617 (m), 1508 (m), 1358 (m), 1284 (m), 1152 (m), 1121 (m), 818 (s).

((2*R,3*R**)-3-(2,4-Difluorophenyl)-1-tosylaziridin-2-yl)(cyclopropyl)methanone, 12l**

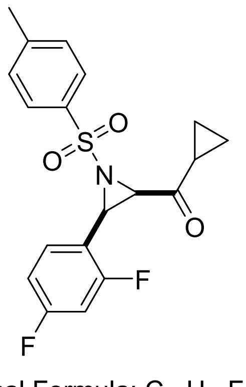

Chemical Formula: $C_{19}H_{17}F_2NO_3S$
Exact Mass: 377.0897

Yield: 70% (210 mg, 0.56 mmol)

Appearance: White solid

HR-MS (QTOF) m/z: [M+H]$^+$ Calcd for $C_{19}H_{17}F_2NO_3SH^+$ 378.0970; Found 378.0971

Melting point: 97 – 99 ^{0}C

Prepared according to General Procedure 4 and purified by flash chromatography (EtOAc:cyclohexane, 3:7, R_f = 0.46). **^{1}H NMR (500 MHz, CDCl$_3$)** δ/ppm 7.92 (d, *J* = 8.3 Hz, 2H), 7.38 (d, *J* = 7.8 Hz, 2H), 7.23 (td, *J* = 8.6, 6.1 Hz, 1H), 6.78 – 6.74 (m, 2H), 4.17 (d, *J* = 7.3 Hz, 1H), 3.83 (d, *J* = 7.8 Hz, 1H), 2.46 (s, 3H), 1.97 – 1.92 (m, 1H), 0.84 – 0.74 (m, 2H),

0.70 – 0.65 (m, 1H), 0.63 – 0.58 (m, 1H). **^{13}C NMR (126 MHz, $CDCl_3$)** δ/ppm 201.0 (C), 163.2 (dd, *J* = 252, 12 Hz, CF), 161.2 (dd, *J* = 252, 13 Hz, CF), 145.6 (C), 133.8 (C), 130.5 (dd, *J* = 10, 5 Hz, CH), 130.2 (2CH), 128.4 (2CH), 115.5 (dd, *J* = 14, 4 Hz, C), 111.4 (dd, *J* = 21, 4 Hz, CH), 103.9 (dd, *J* = 25, 24 Hz, CH), 48.9 (CH), 40.9 (d, *J* = 4 Hz, CH), 21.9 (CH_3), 19.3 (CH), 12.2 (CH_2), 12.2 (CH_2). **^{19}F NMR (376 MHz, $CDCl_3$)** δ/ppm -108.7 (m), -113.3 (m). **IR (neat) v/cm^{-1}:** 3080 (w), 3071 (w), 3011 (w), 1694 (m), 1598 (m), 1507 (m), 1335 (s), 1163 (s), 1092 (s), 816 (m).

((2*R,3*R**)-3-(4-Bromophenyl)-1-tosylaziridin-2-yl)(cyclopropyl)methanone, 12m**

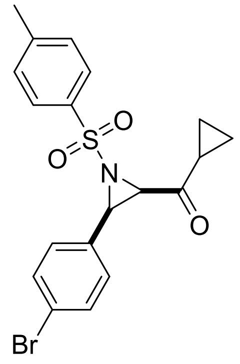

Chemical Formula: $C_{19}H_{18}BrNO_3S$
Exact Mass: 419.0191

Yield: 70% (210 mg, 0.50 mmol)

Appearance: White solid

HR-MS (QTOF) m/z: $[M+Na]^+$ Calcd for $C_{19}H_{18}{}^{81}BrNO_3SNa^+$ 444.0064; Found 444.0063

Melting point: 134 – 136 ^{0}C

Prepared according to General Procedure 4 and purified by flash chromatography (EtOAc:cyclohexane, 3:7, R_f = 0.40). **^{1}H NMR (500 MHz, $CDCl_3$)** δ/ppm 7.92 (d, *J* = 8.3 Hz, 2H), 7.40 – 7.37 (m, 4H), 7.10 (d, *J* = 8.3 Hz, 2H), 4.07 (d, *J* = 7.8 Hz, 1H), 3.75 (d, *J* = 7.8 Hz, 1H), 2.46 (s, 3H), 1.93 (tt, *J* = 7.8, 4.6 Hz, 1H), 0.88 – 0.83 (m, 1H), 0.77 – 0.72 (m, 1H), 0.60 – 0.51 (m, 2H). **^{13}C NMR (126 MHz, $CDCl_3$)** δ/ppm 201.9 (C), 145.6 (C), 133.9 (C), 131.7 (2CH), 130.9 (C), 130.2 (2CH), 129.3 (2CH), 128.3 (2CH), 122.7 (C), 49.7 (CH), 45.4 (CH), 21.9 (CH_3), 19.3 (CH), 12.6 (CH_2), 12.4 (CH_2). **IR (neat) v/cm^{-1}:** 2993 (w), 2922 (w), 2852 (w), 1703 (m), 1595 (m), 1489 (m), 1337 (s), 1154 (s), 1081 (s), 660 (m).

((2*R,3*R**)-3-(Naphthalen-2-yl)-1-tosylaziridin-2-yl)(cyclopropyl)methanone, 12n**

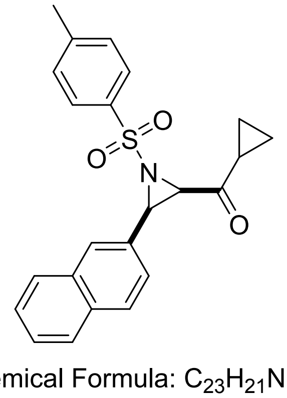

Chemical Formula: $C_{23}H_{21}NO_3S$
Exact Mass: 391.1242

Yield: 78% (234 mg, 0.60 mmol)

Appearance: White solid

HR-MS (QTOF) m/z: $[M+Na]^+$ Calcd for $C_{23}H_{21}NO_3SNa^+$ 414.1134; Found 414.1140

Prepared according to General Procedure 4 and purified by flash chromatography (EtOAc:cyclohexane, 3:7, R_f = 0.37). **^{1}H NMR (500 MHz, $CDCl_3$)** δ/ppm 7.97 (d, *J* = 8.3 Hz, 2H), 7.80 – 0.76 (m, 1H), 7.74 – 7.73 (m, 2H), 7.71 (s, 1H), 7.48 – 7.44 (m, 2H), 7.39 (d, *J* = 8.3 Hz, 2H), 7.31 (dd, *J* = 8.6, 1.7 Hz, 1H), 4.29 (d, *J* = 7.8 Hz, 1H), 3.81 (d, *J* = 8.3 Hz, 1H),

2.46 (s, 3H), 2.01 – 1.96 (m, 1H), 0.83 – 0.78 (m, 1H), 0.67 (qd, J = 7.3, 3.4 Hz, 1H), 0.46 – 0.41 (m, 1H), 0.34 (qd, J = 8.3, 3.9 Hz, 1H). **^{13}C NMR (126 MHz, $CDCl_3$)** δ/ppm 202.5 (C), 145.5 (C), 134.1 (C), 133.2 (C), 133.0 (C), 130.2 (2CH), 129.2 (C), 128.4 (2CH), 128.4 (CH), 128.0 (CH), 127.9 (CH), 127.1 (CH), 126.6 (CH), 126.6 (CH), 124.8 (CH), 49.9 (CH), 46.2 (CH), 21.9 (CH_3), 19.3 (CH), 12.6 (CH_2), 12.3 (CH_2). **IR (neat) v/cm^{-1}:** 2954 (w), 2924 (w), 2855 (w), 1703 (m), 1595 (m), 1509 (m), 1341 (s), 1154 (s), 1081 (s), 820 (s).

((2*R**,3*R**)-3-(4-Fluorophenyl)-1-tosylaziridin-2-yl)(phenyl)methanone, 12p

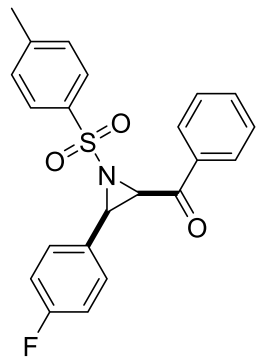

Chemical Formula: $C_{22}H_{18}FNO_3S$
Exact Mass: 395.0991

Yield: 78% (234 mg, 0.59 mmol)

Appearance: Pale yellow oil

HR-MS (QTOF) m/z: $[M+Na]^+$ Calcd for $C_{22}H_{18}FNO_3SNa^+$ 418.0884; Found 418.0882

Melting point: 138 – 140 ^{0}C

Prepared according to General Procedure 4 and purified by flash chromatography (EtOAc:cyclohexane, 3:7, R_f = 0.31). **^{1}H NMR (400 MHz, $CDCl_3$)** δ/ppm 7.97 (d, J = 8.2 Hz, 2H), 7.86 – 7.84 (m, 2H), 7.55 (t, J = 7.4 Hz, 1H), 7.42 – 7.37 (m, 3H), 7.35 (s, 1H), 7.24 – 7.19 (m, 2H), 6.89 – 6.83 (m, 2H), 4.40 (d, J = 7.4 Hz, 1H), 4.33 (d, J = 7.8 Hz, 1H), 2.44 (s, 3H). **^{13}C NMR (101 MHz, $CDCl_3$)** δ/ppm 188.9 (C), 162.9 (d, J = 248 Hz, CF), 145.4 (C), 135.7 (C), 134.4 (C), 134.1 (CH), 130.1 (CH), 129.3 (d, J = 8 Hz, 2CH), 128.9 (3CH), 128.5 (2CH), 128.2 (2CH), 127.1 (d, J = 3 Hz, C), 115.6 (d, J = 22 Hz, 2CH), 48.3 (CH), 45.9 (CH), 21.9 (CH_3). **^{19}F NMR (376 MHz, $CDCl_3$)** δ/ppm -112.9 (m). **IR (neat) v/cm^{-1}:** 2955 (w), 2924 (m), 2854 (w), 1691 (m), 1597 (m), 1511 (m), 1327 (m), 1160 (s), 1091 (m), 814 (m).

This data is consistent with published work. [SI16]

((2*R**,3*R**)-3-(4-Bromophenyl)-1-tosylaziridin-2-yl)(phenyl)methanone, 12q

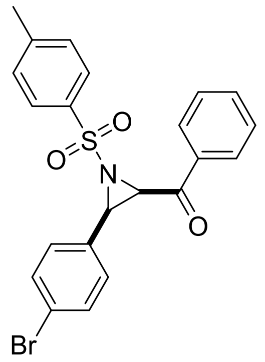

Chemical Formula: $C_{22}H_{18}BrNO_3S$
Exact Mass: 455.0191

Yield: 61% (183 mg, 0.40 mmol)

Appearance: Pale yellow oil

HR-MS (QTOF) m/z: $[M+Na]^+$ Calcd for $C_{22}H_{18}{}^{81}BrNO_3SNa^+$ 480.0064; Found 480.0059

Prepared according to General Procedure 4 and purified by flash chromatography (EtOAc:cyclohexane, 3:7, R_f = 0.39). **^{1}H NMR (500 MHz, CDCl$_3$)** δ/ppm 7.96 (d, *J* = 8.3 Hz, 2H), 7.85 (dd, *J* = 8.1, 1.2 Hz, 2H), 7.57 – 7.53 (m, 1H), 7.43 – 7.39 (m, 2H), 7.36 (d, *J* = 7.8 Hz, 2H), 7.31 – 7.29 (m, 2H), 7.11 (d, *J* = 8.3 Hz, 2H), 4.42 (d, *J* = 7.8 Hz, 1H), 4.29 (d, *J* = 7.8 Hz, 1H), 2.44 (s, 3H). **^{13}C NMR (126 MHz, CDCl$_3$)** δ/ppm 188.8 (C), 145.5 (C), 135.6 (C), 134.3 (C), 134.2 (CH), 131.7 (2CH), 130.4 (C), 130.1 (2CH), 129.2 (2CH), 128.9 (2CH), 128.5 (2CH), 128.2 (2CH), 122.9 (C), 48.2 (CH), 45.9 (CH), 21.9 (CH$_3$). **IR (neat) v/cm^{-1}:** 2988 (w), 2923 (w), 1690 (m), 1596 (m), 1489 (m), 1327 (m), 1157 (s), 1089 (m), 813 (m), 676 (s).

This data is consistent with published work.[SI17]

((2*R,3*R**)-3-(Naphthalen-2-yl)-1-tosylaziridin-2-yl)(phenyl)methanone, 12r**

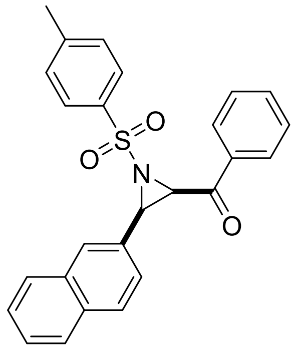

Chemical Formula: $C_{26}H_{21}NO_3S$
Exact Mass: 427.1242

Yield: 38% (114 mg, 0.27 mmol)

Appearance: White solid

HR-MS (QTOF) m/z: [M+Na]$^+$ Calcd for $C_{26}H_{21}NO_3SNa^+$ 450.1134; Found 450.1138

Prepared according to General Procedure 4 and purified by flash chromatography (EtOAc:cyclohexane, 3:7, R_f = 0.36). **^{1}H NMR (500 MHz, CDCl$_3$)** δ/ppm 8.01 (d, *J* = 8.3 Hz, 2H), 7.86 (dd, *J* = 8.6, 1.2 Hz, 2H), 7.73 (s, 1H), 7.72 – 7.68 (m, 2H), 7.64 (d, *J* = 8.8 Hz, 1H), 7.52 – 7.49 (m, 1H), 7.42 – 7.35 (m, 6H), 7.31 (dd, *J* = 8.6, 1.7 Hz, 1H), 4.51 (d, *J* = 7.8 Hz, 1H), 4.48 (d, *J* = 7.8 Hz, 1H), 2.43 (s, 3H). **^{13}C NMR (126 MHz, CDCl$_3$) δ**/ppm 189.1 (C), 145.3 (C), 135.8 (C), 134.5 (C), 134.0 (CH), 133.3 (C), 132.9 (C), 130.1 (2CH), 128.8 (2CH), 128.5 (2CH), 128.3 (CH), 128.3 (2CH), 128.2 (C), 127.7 (2CH), 127.2 (CH), 126.5 (CH), 126.4 (CH), 124.6 (CH), 48.6 (CH), 46.9 (CH), 21.9 (CH$_3$). **IR (neat) v/cm^{-1}:** 2954 (m), 2923 (m), 2854 (m), 1689 (m), 1596 (m), 1509 (m), 1328 (s), 1158 (s), 1089 (s), 814 (m).

5. Copies of ^{1}H and ^{13}C NMR Spectra

1-(2,4-Difluorophenyl)hept-2-yn-1-ol, 9a

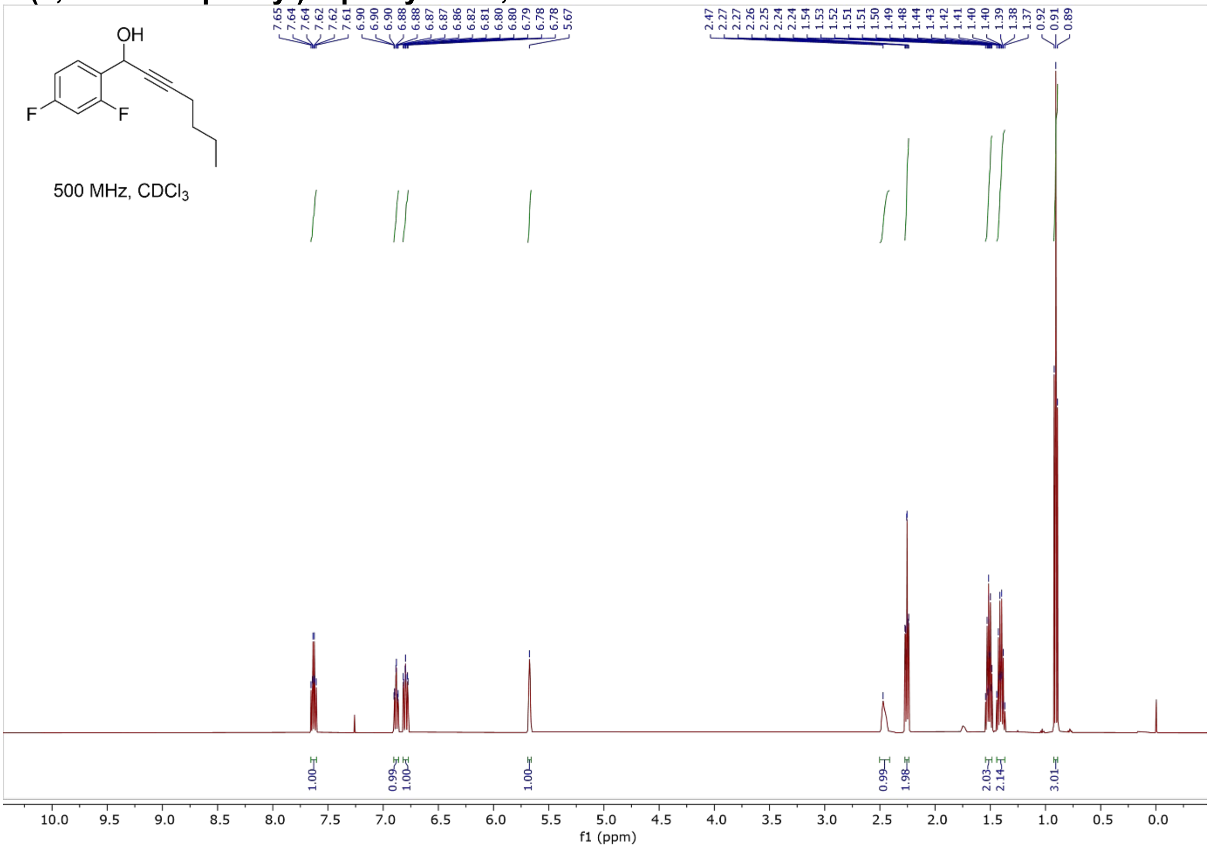

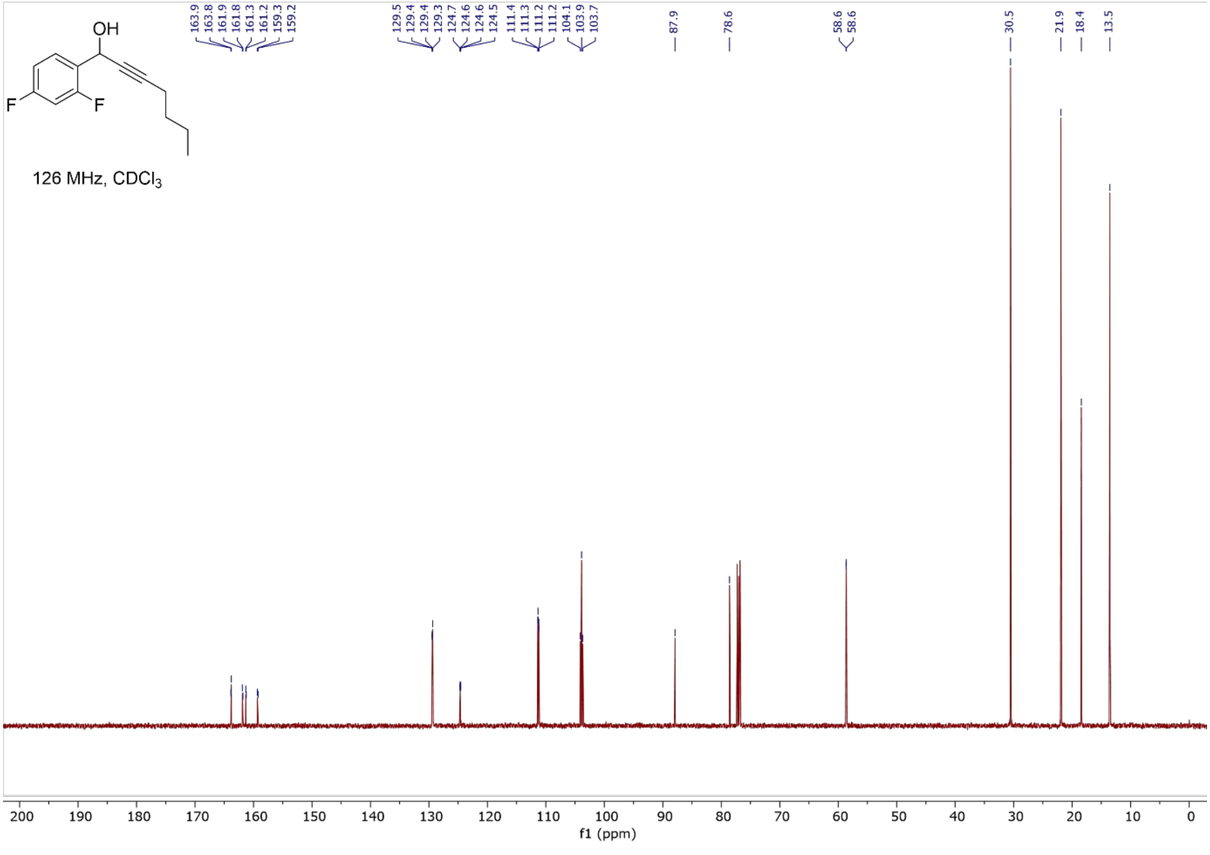

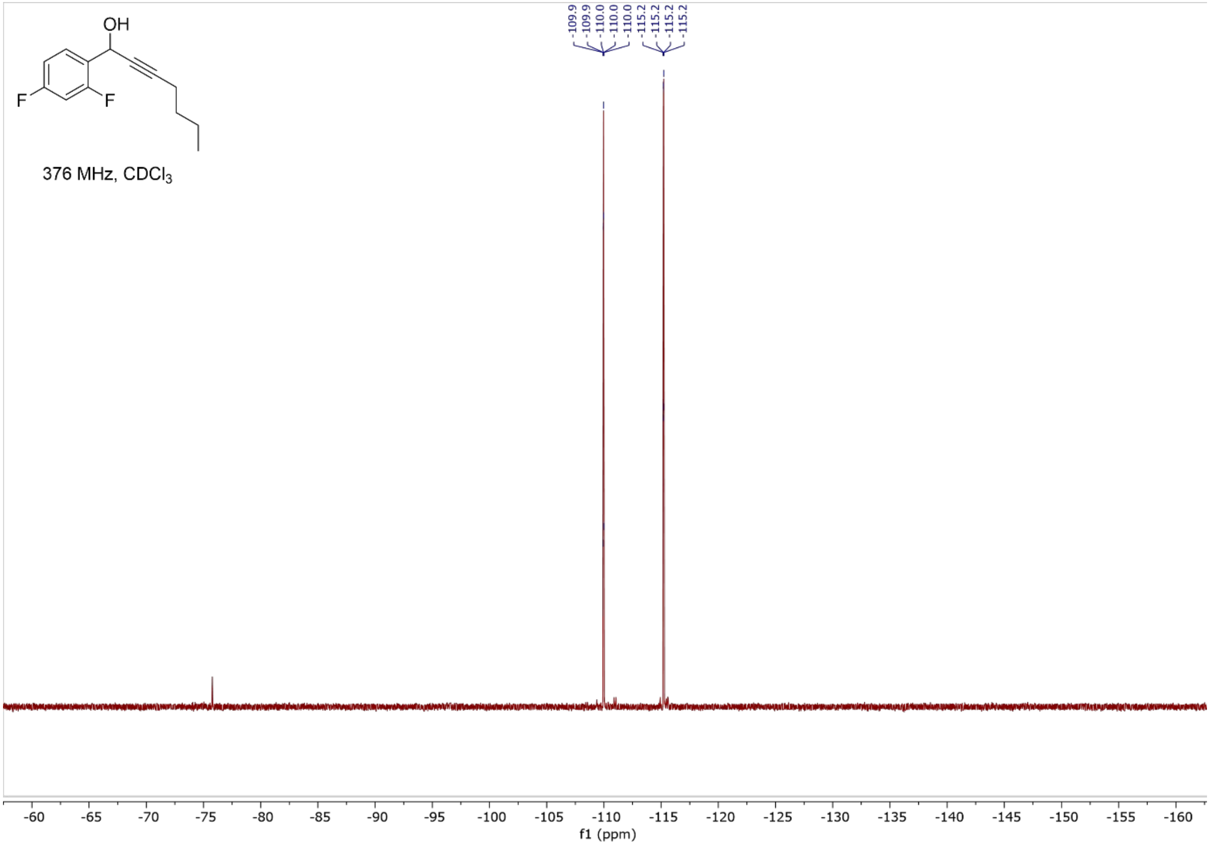

1-(4-Fluorophenyl)hept-2-yn-1-ol, 9b

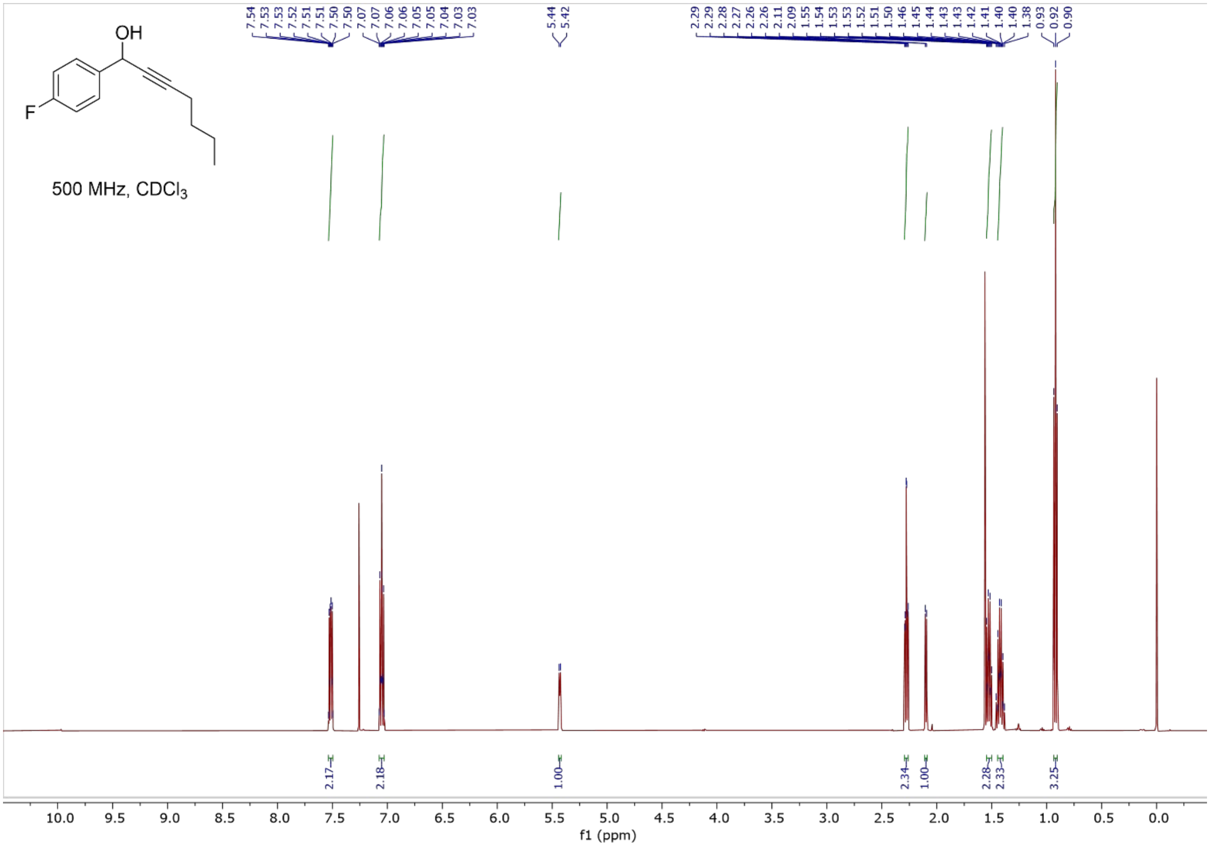

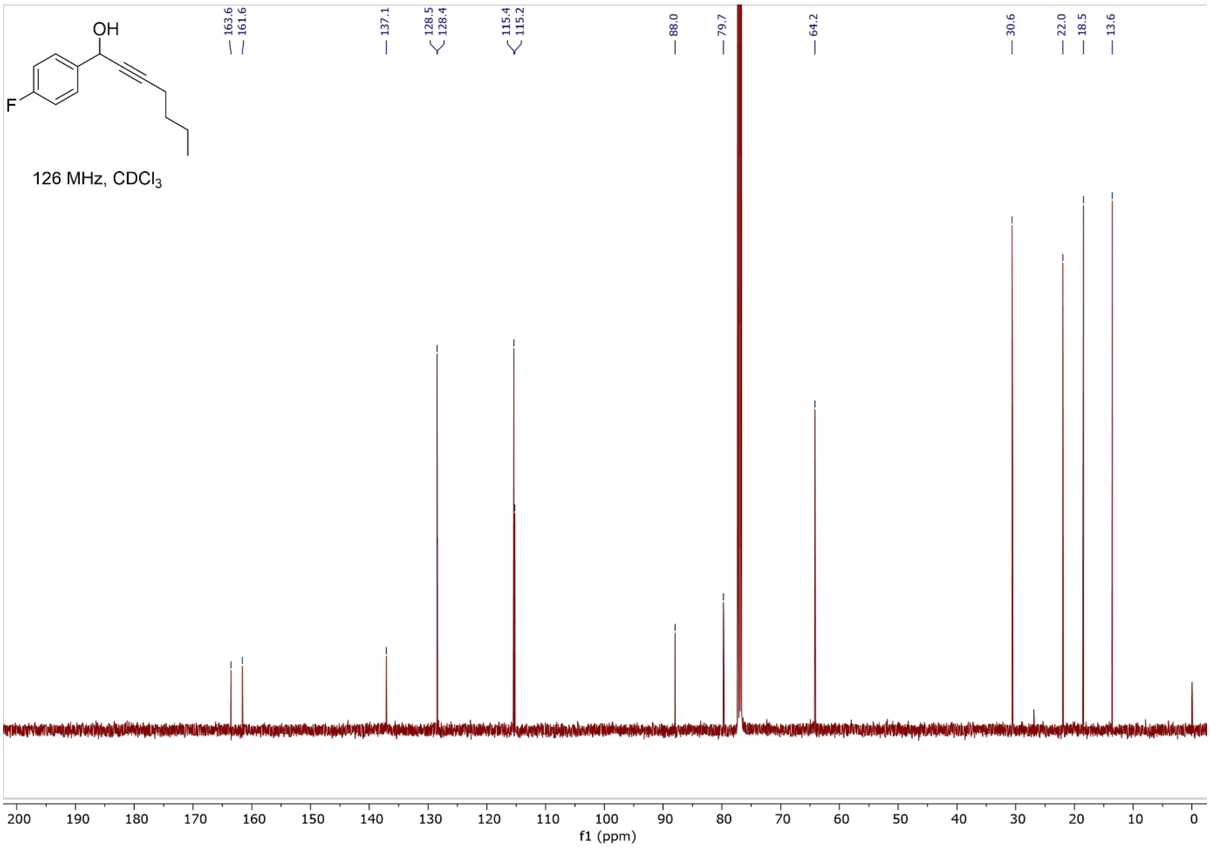
OH
F
126 MHz, CDCl3
163.6
161.6
137.1
128.5
128.4
115.4
115.2
88.0
79.7
64.2
30.6
22.0
18.5
13.6
200 190 180 170 160 150 140 130 120 110 100 90 80 70 60 50 40 30 20 10 0
f1 (ppm)

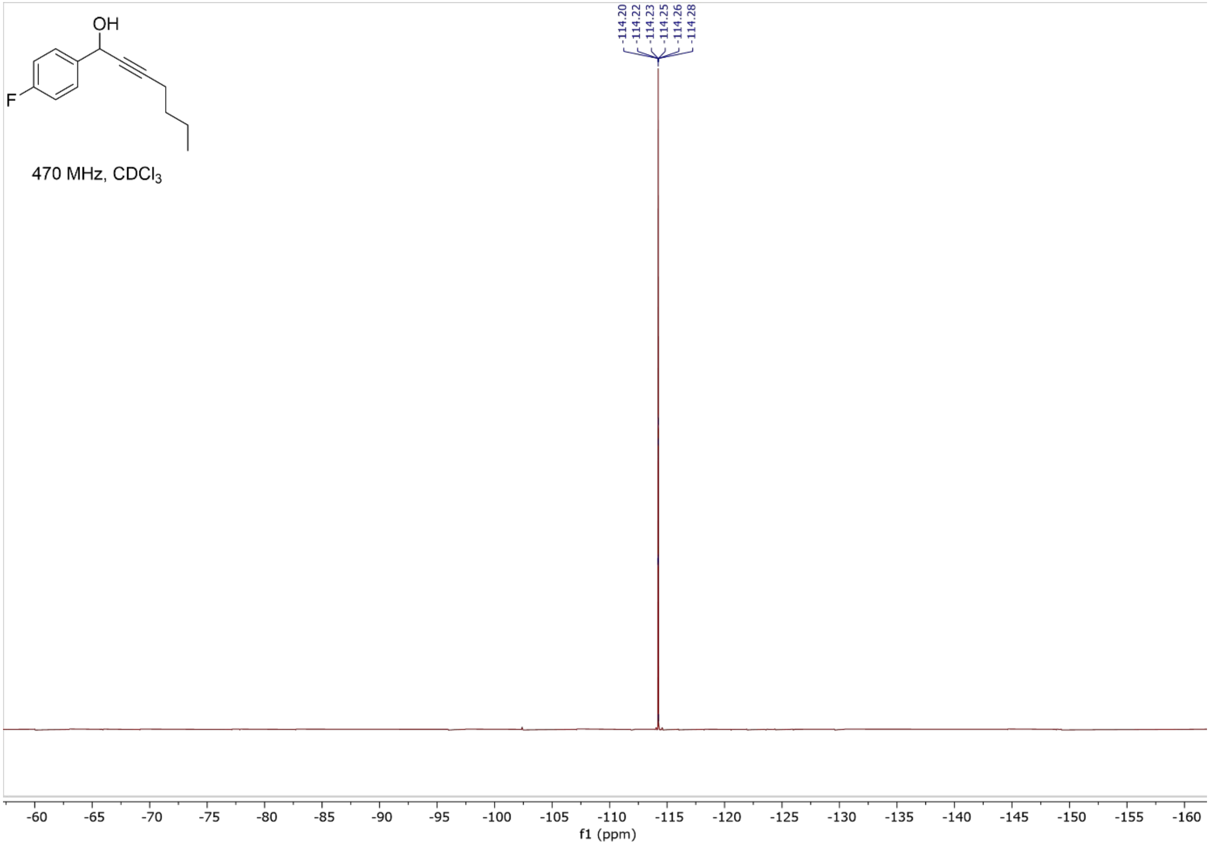
OH
F
470 MHz, CDCl3
-114.20
-114.22
-114.23
-114.25
-114.26
-114.28
-60 -65 -70 -75 -80 -85 -90 -95 -100 -105 -110 -115 -120 -125 -130 -135 -140 -145 -150 -155 -160
f1 (ppm)

1-(4-Bromophenyl)hept-2-yn-1-ol, 9c

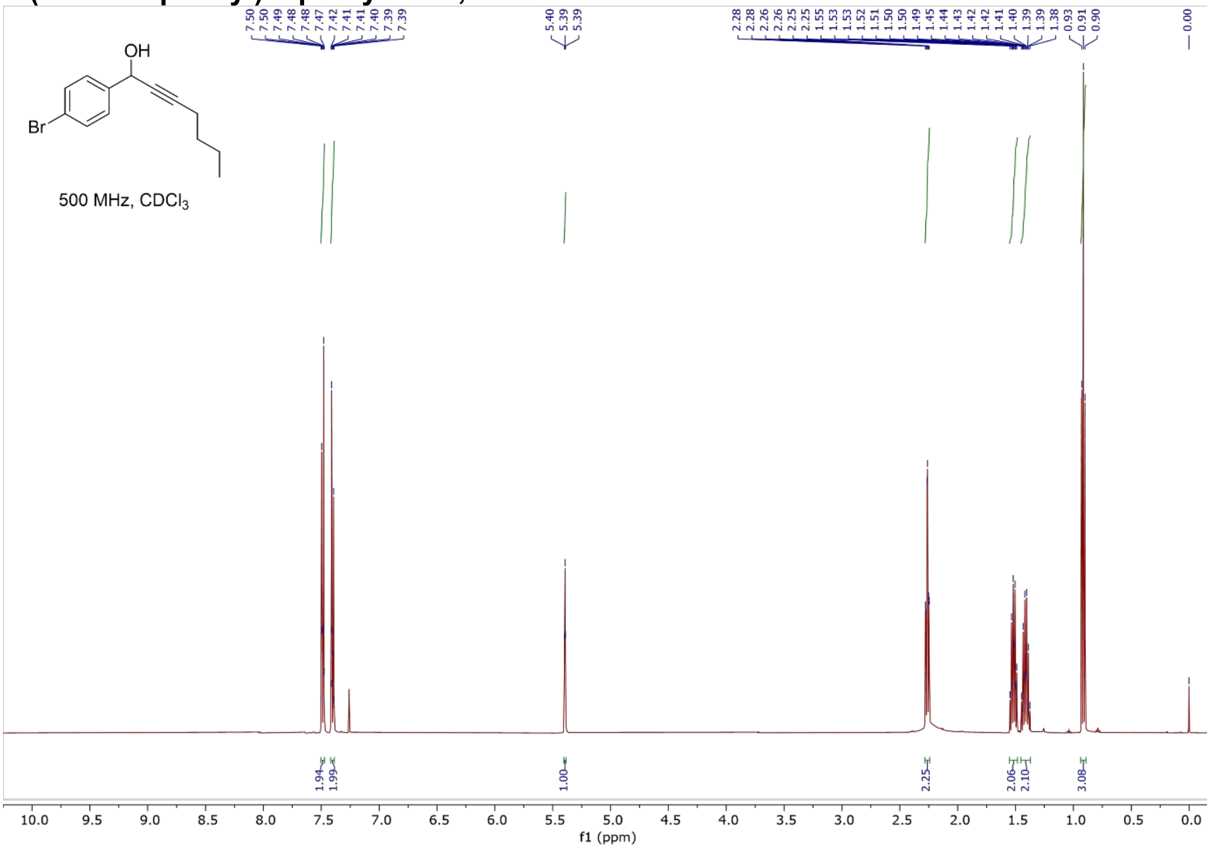

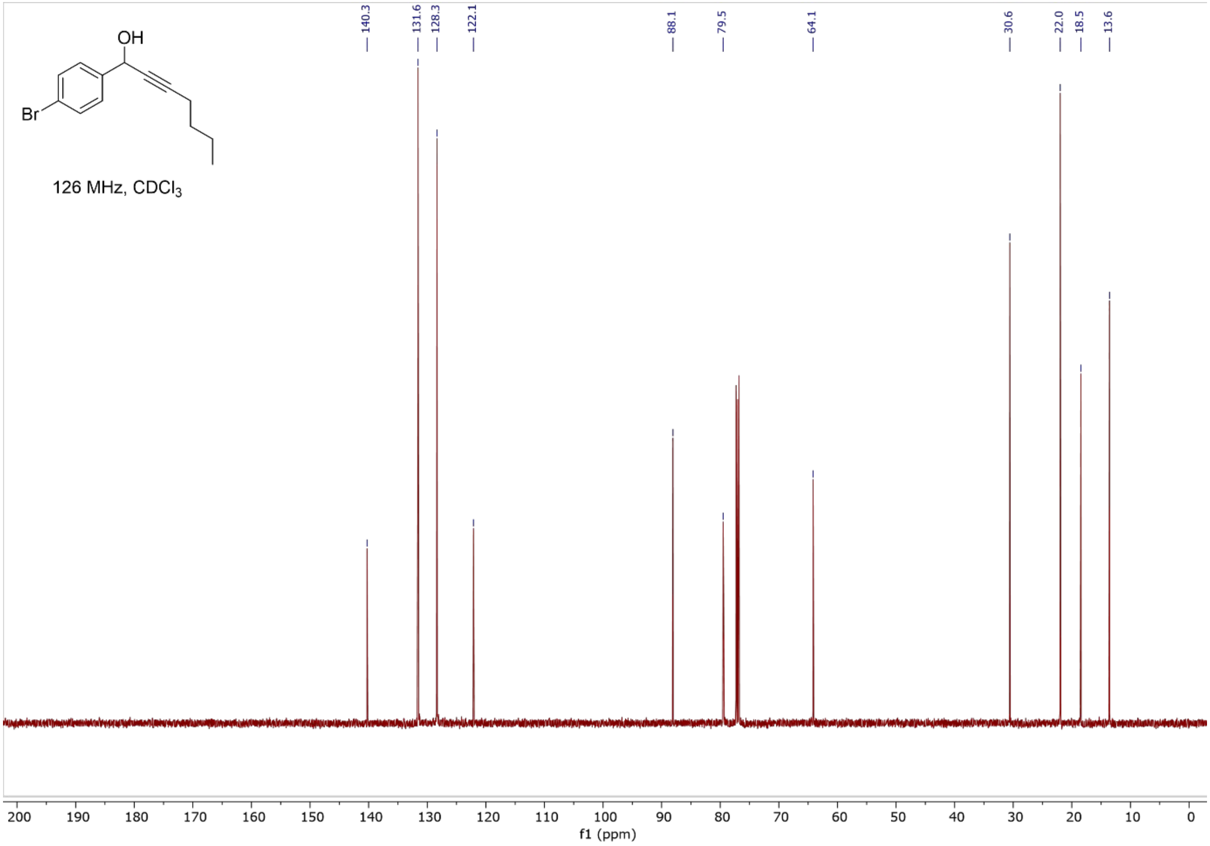

1-(2-Chlorophenyl)hept-2-yn-1-ol, 9d

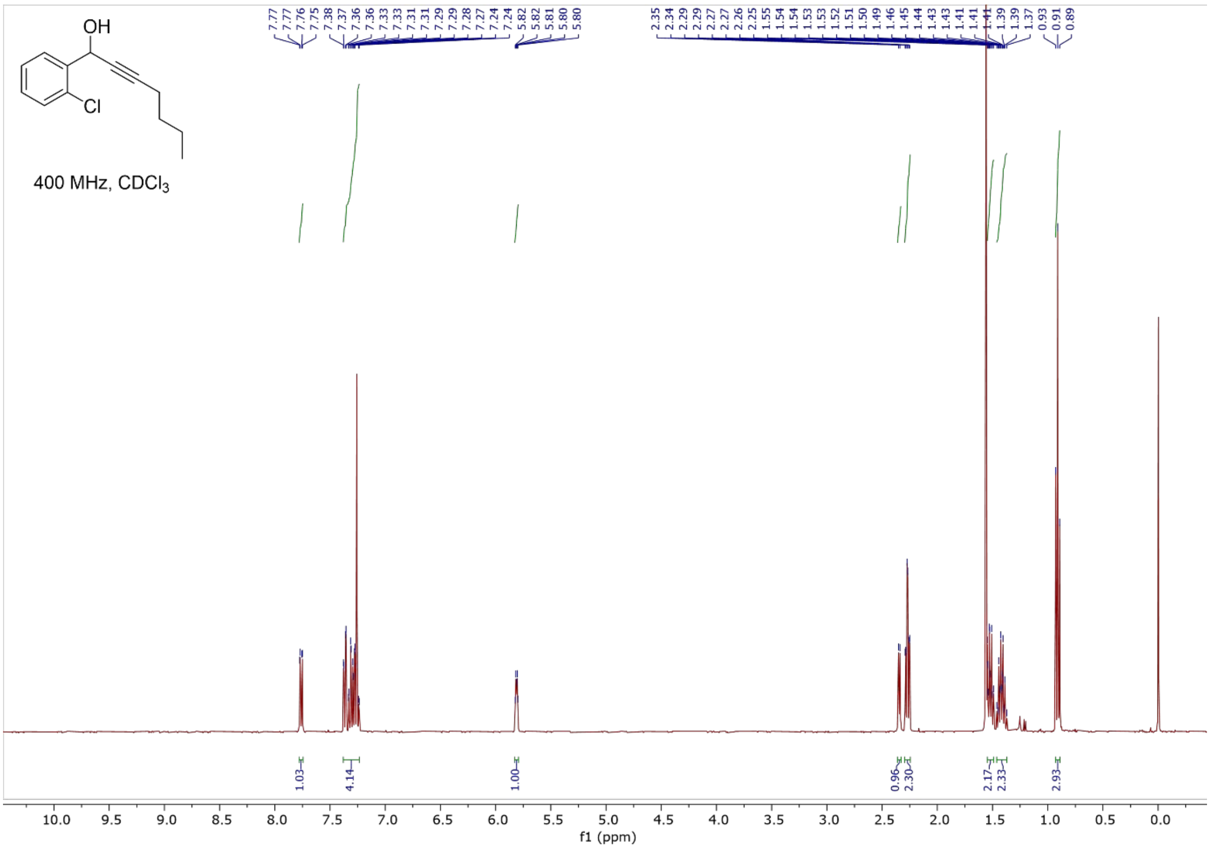

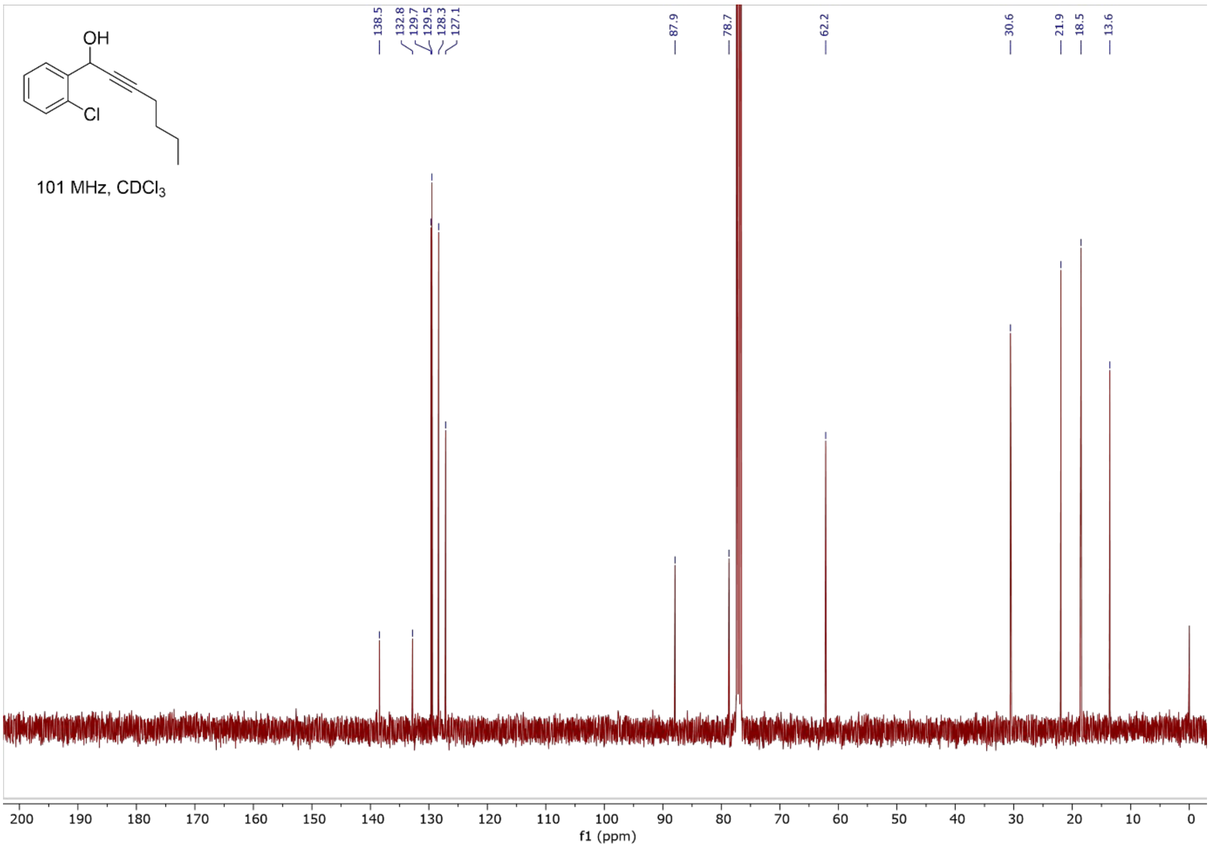

1-(3-Chloro-4-fluorophenyl)hept-2-yn-1-ol, 9e

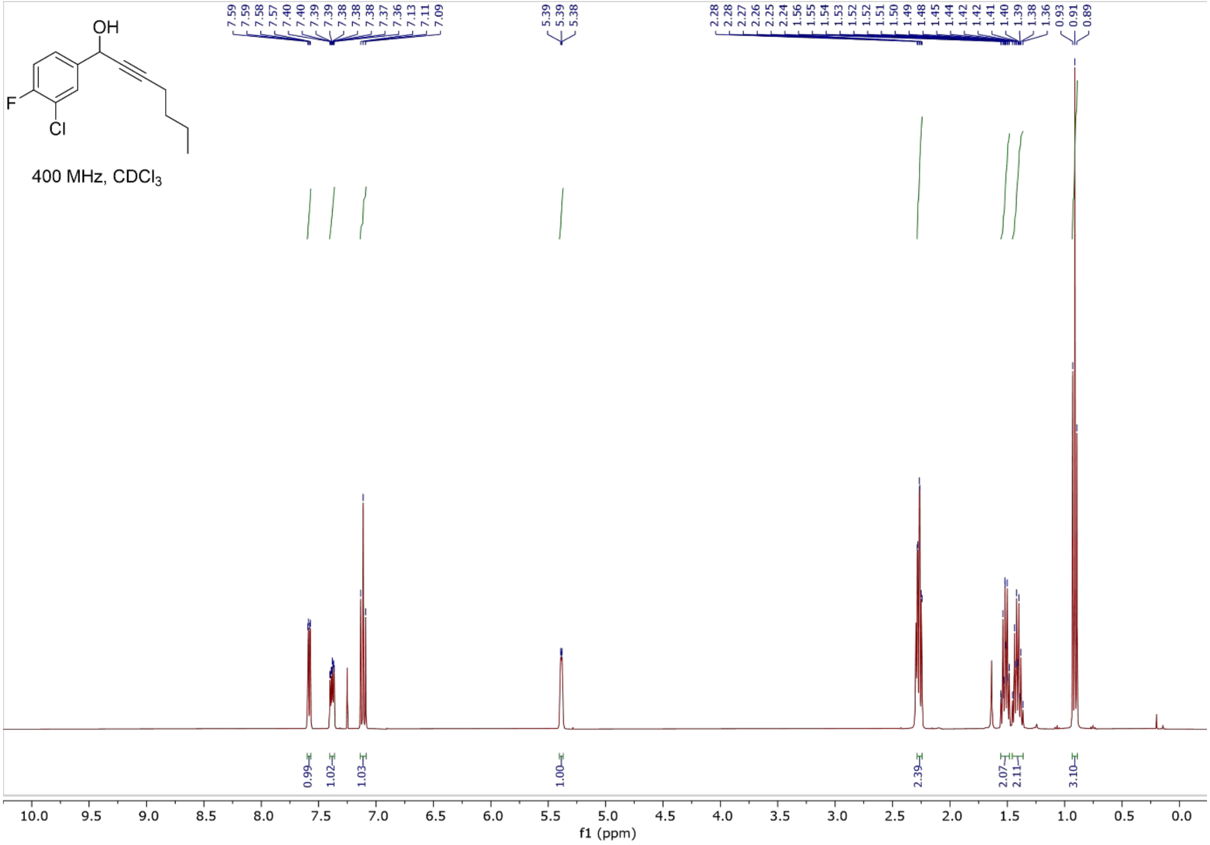

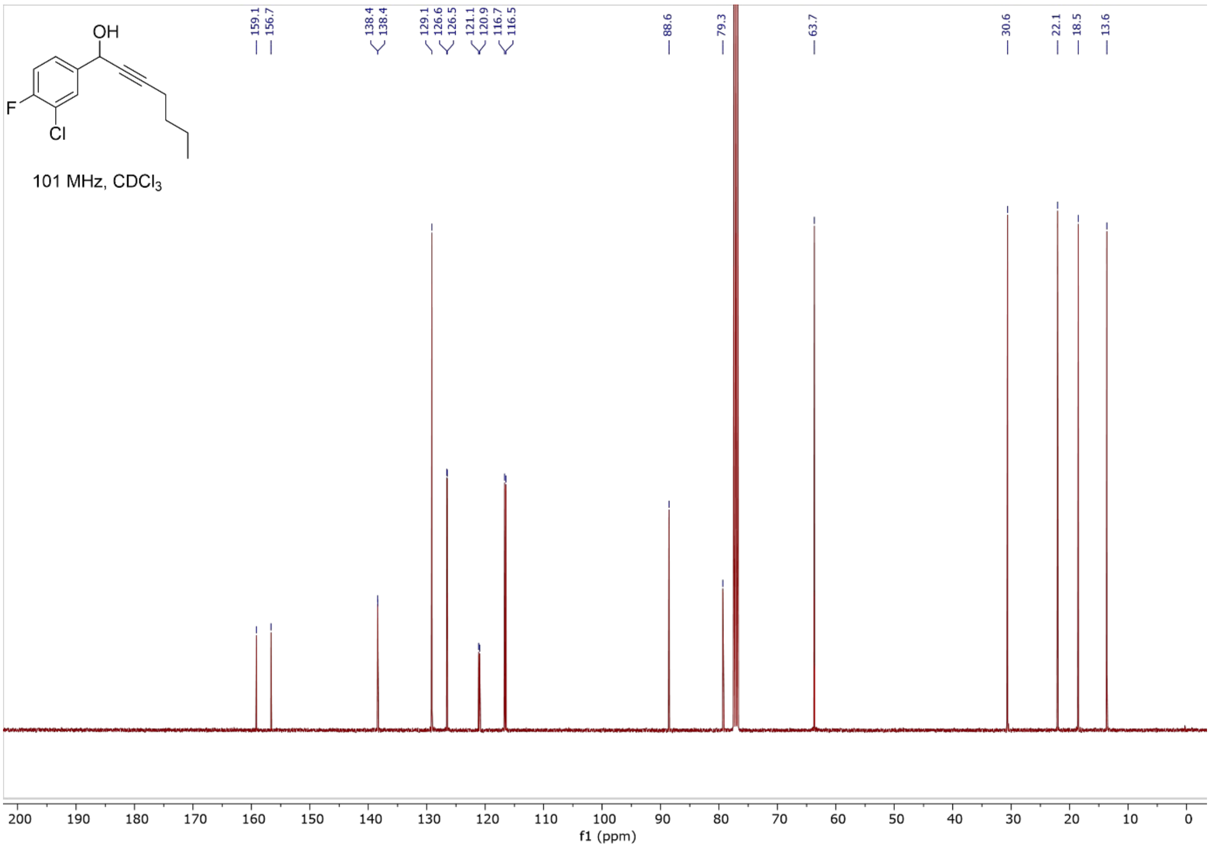

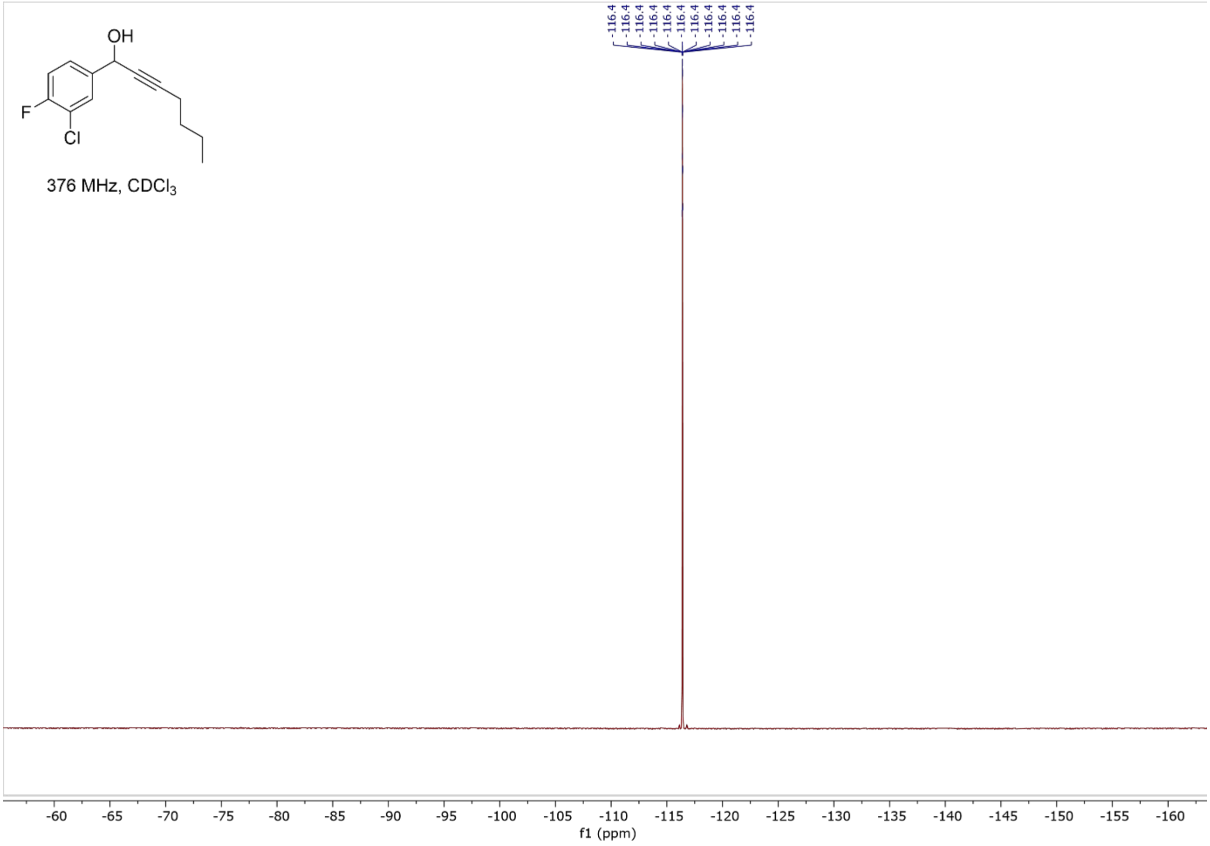

1-(4-(Trifluoromethyl)phenyl)hept-2-yn-1-ol, 9f

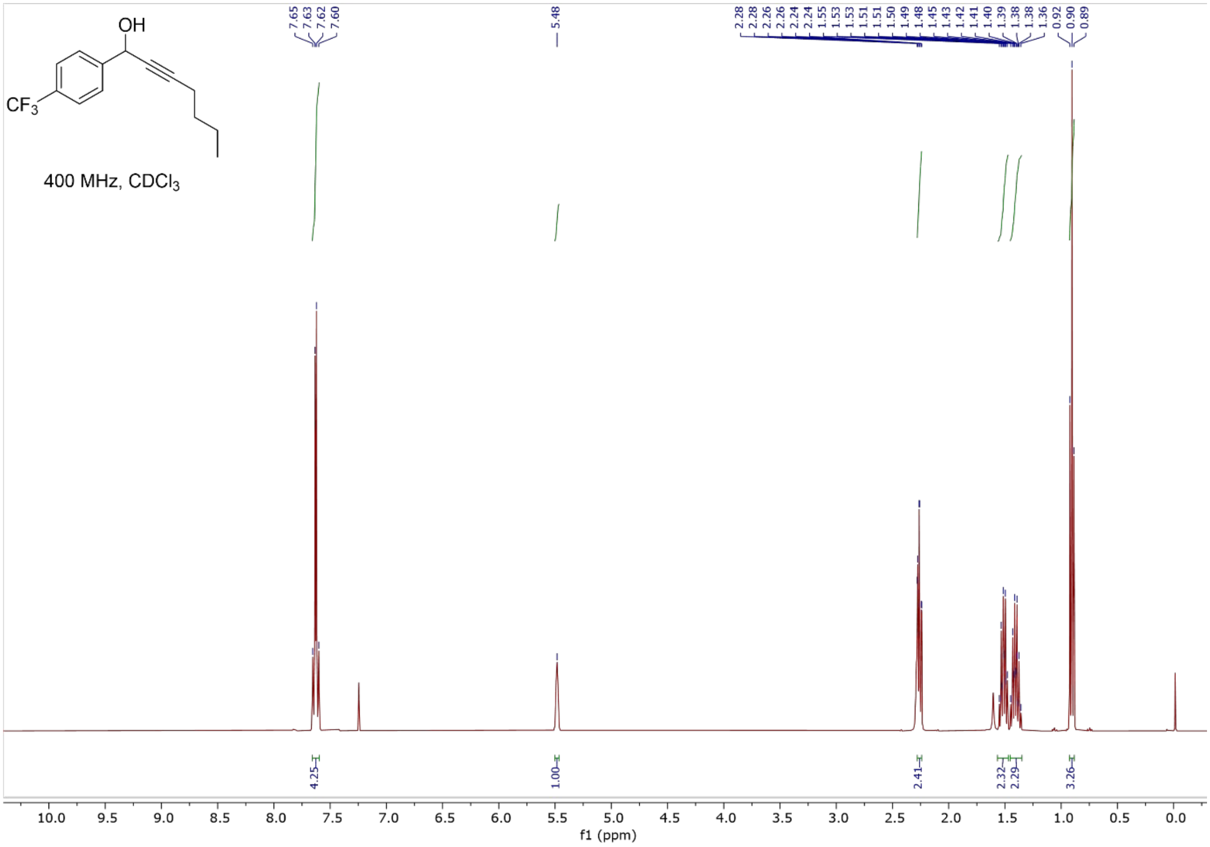

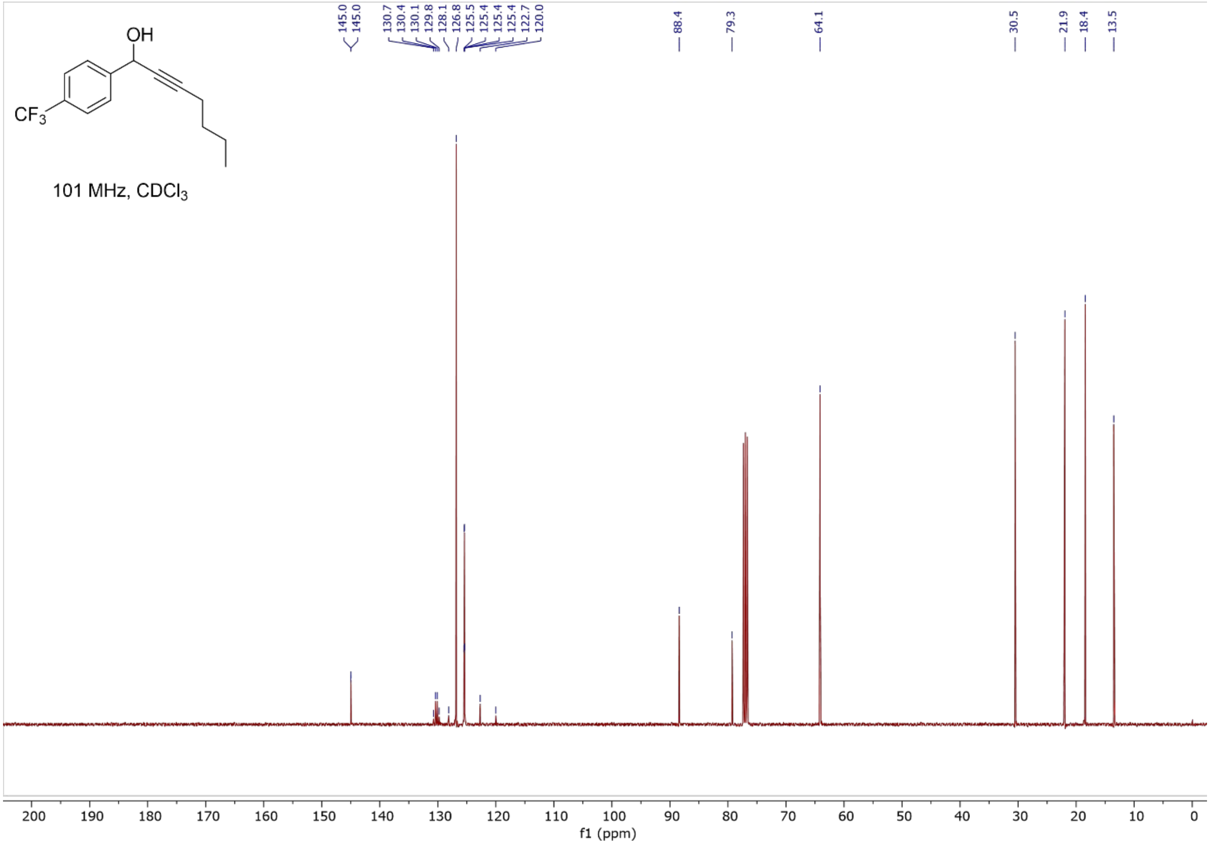

145.0
145.0
130.7
130.4
130.1
129.8
128.1
126.8
125.5
125.4
125.4
125.4
122.7
120.0
88.4
79.3
64.1
30.5
21.9
18.4
13.5
OH
CF3
101 MHz, CDCl3
200 190 180 170 160 150 140 130 120 110 100 90 80 70 60 50 40 30 20 10 0
f1 (ppm)

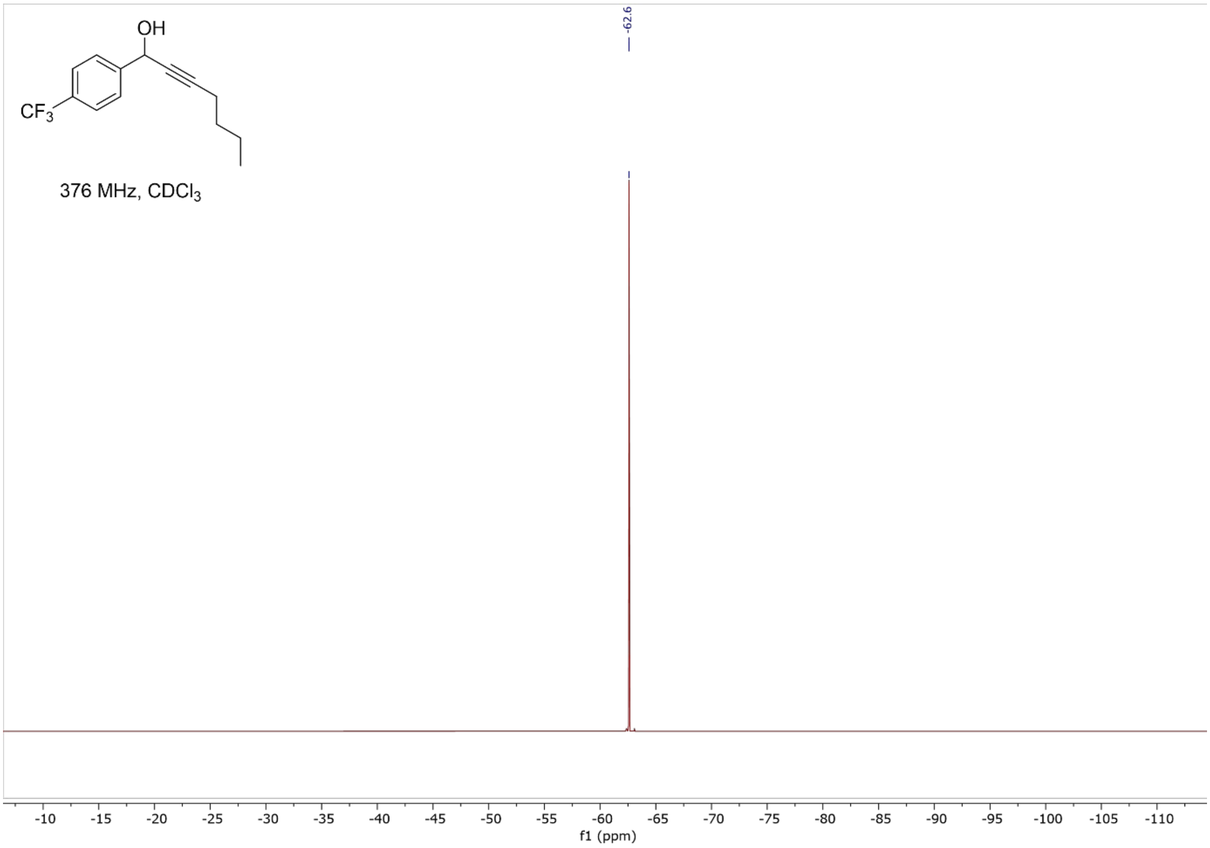

-62.6
OH
CF3
376 MHz, CDCl3
-10 -15 -20 -25 -30 -35 -40 -45 -50 -55 -60 -65 -70 -75 -80 -85 -90 -95 -100 -105 -110
f1 (ppm)

1-Phenylhept-2-yn-1-ol, 9g

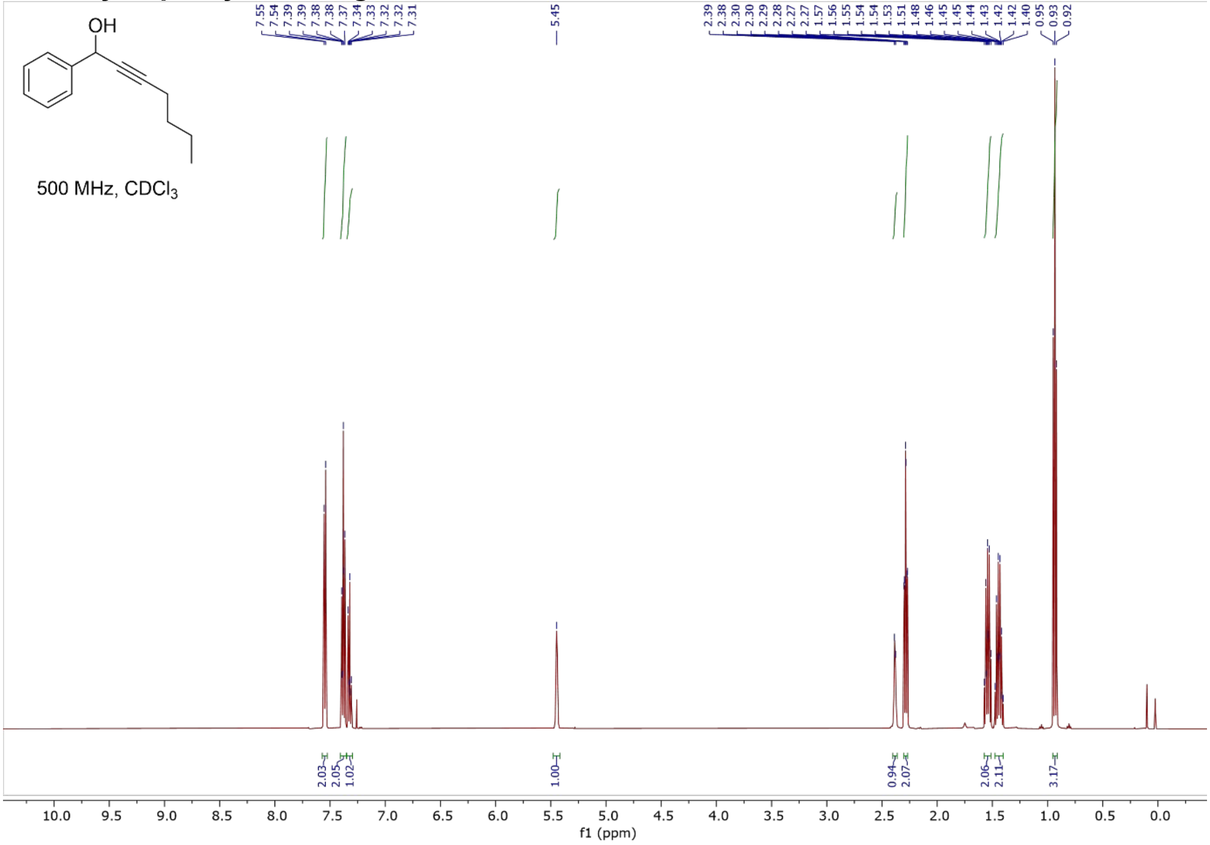

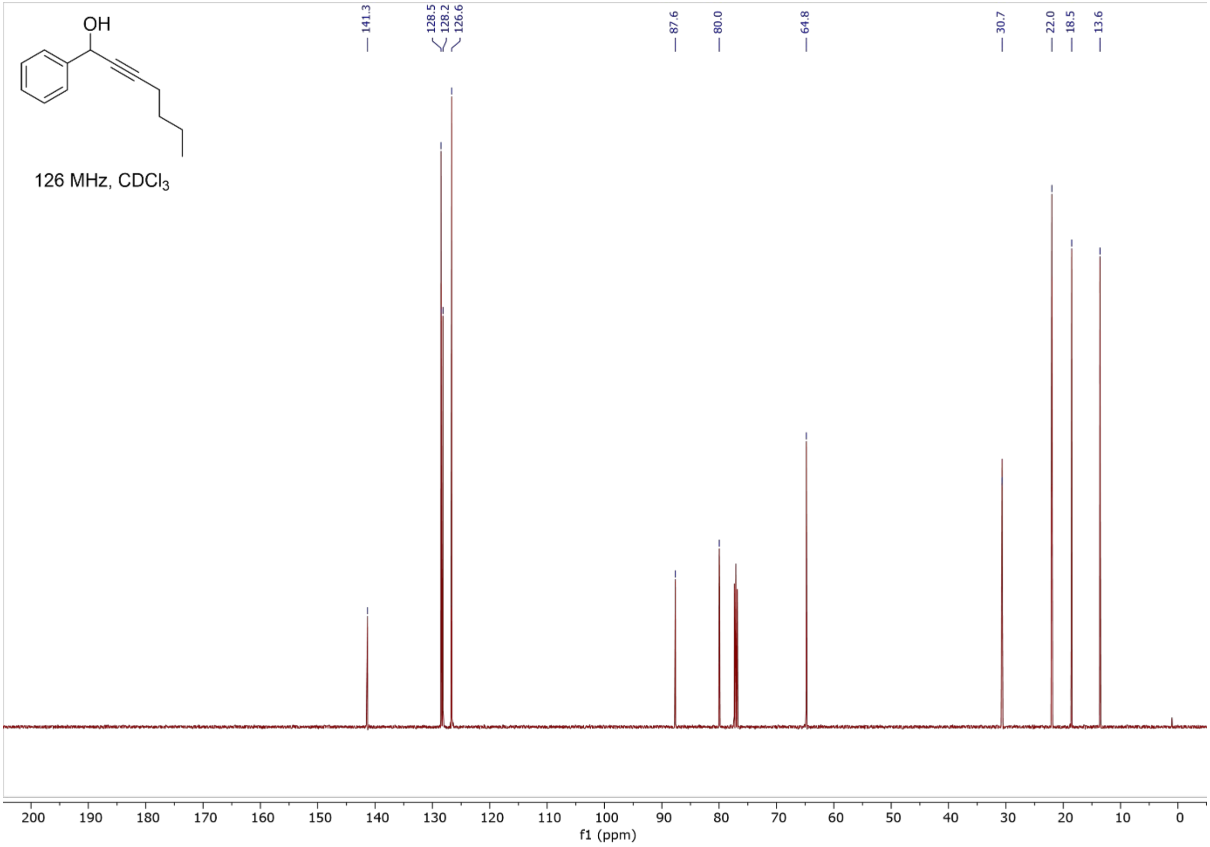

1-(3,4-Dimethylphenyl)hept-2-yn-1-ol, 9h

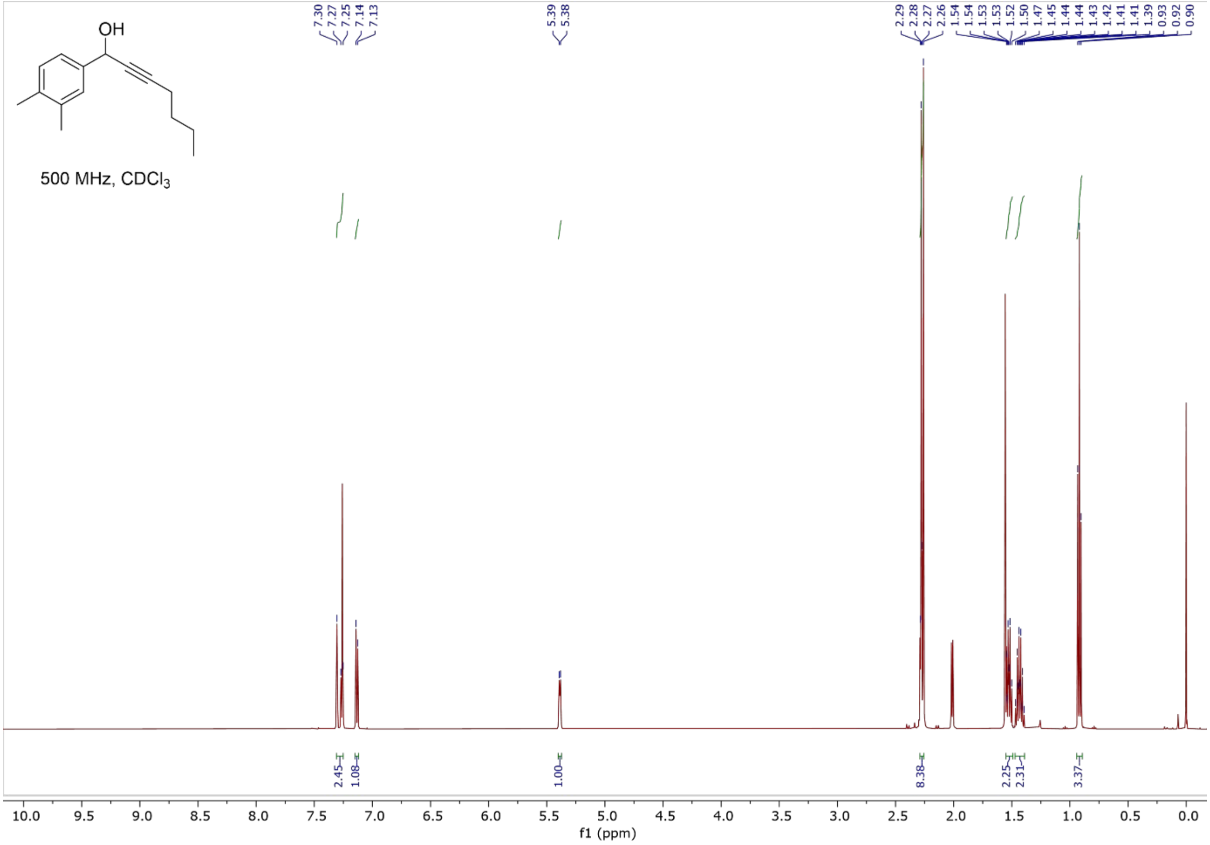

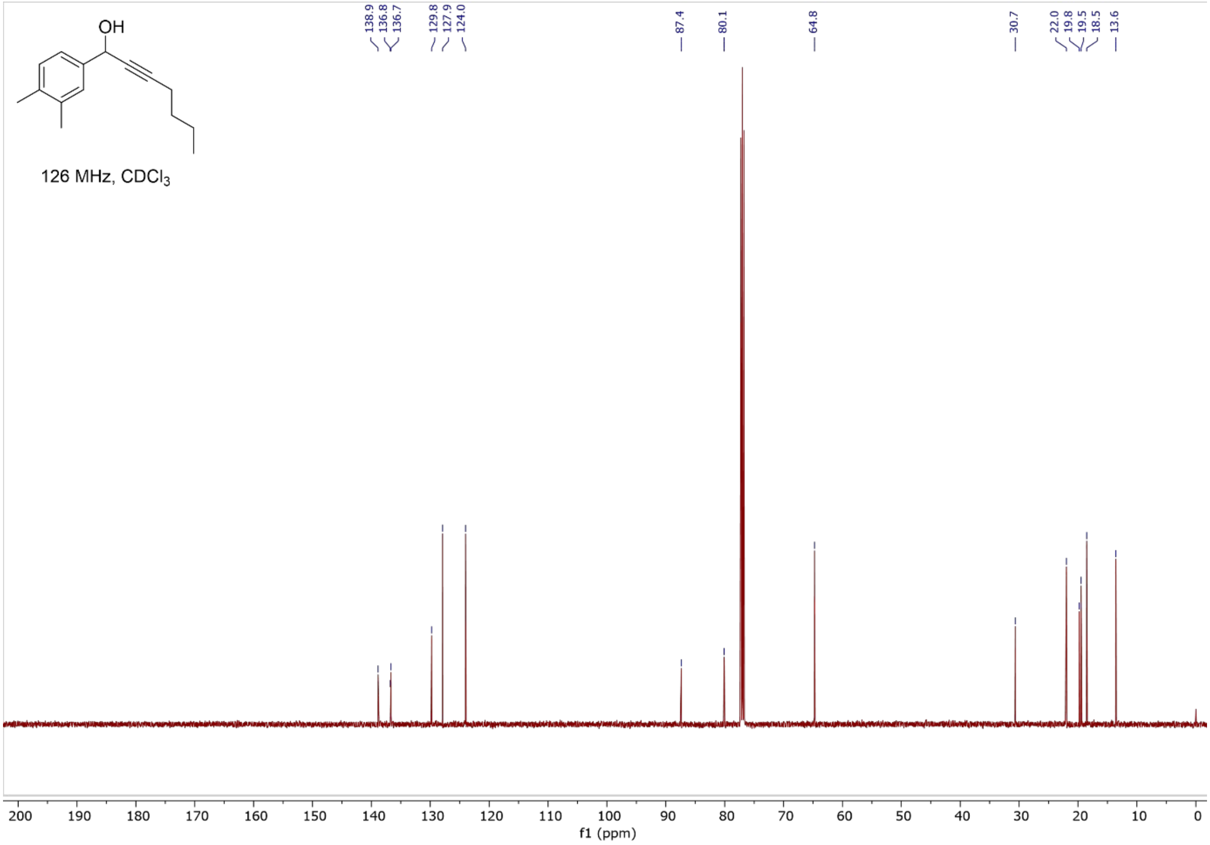

1-(3,4-Dimethoxyphenyl)hept-2-yn-1-ol, 9i

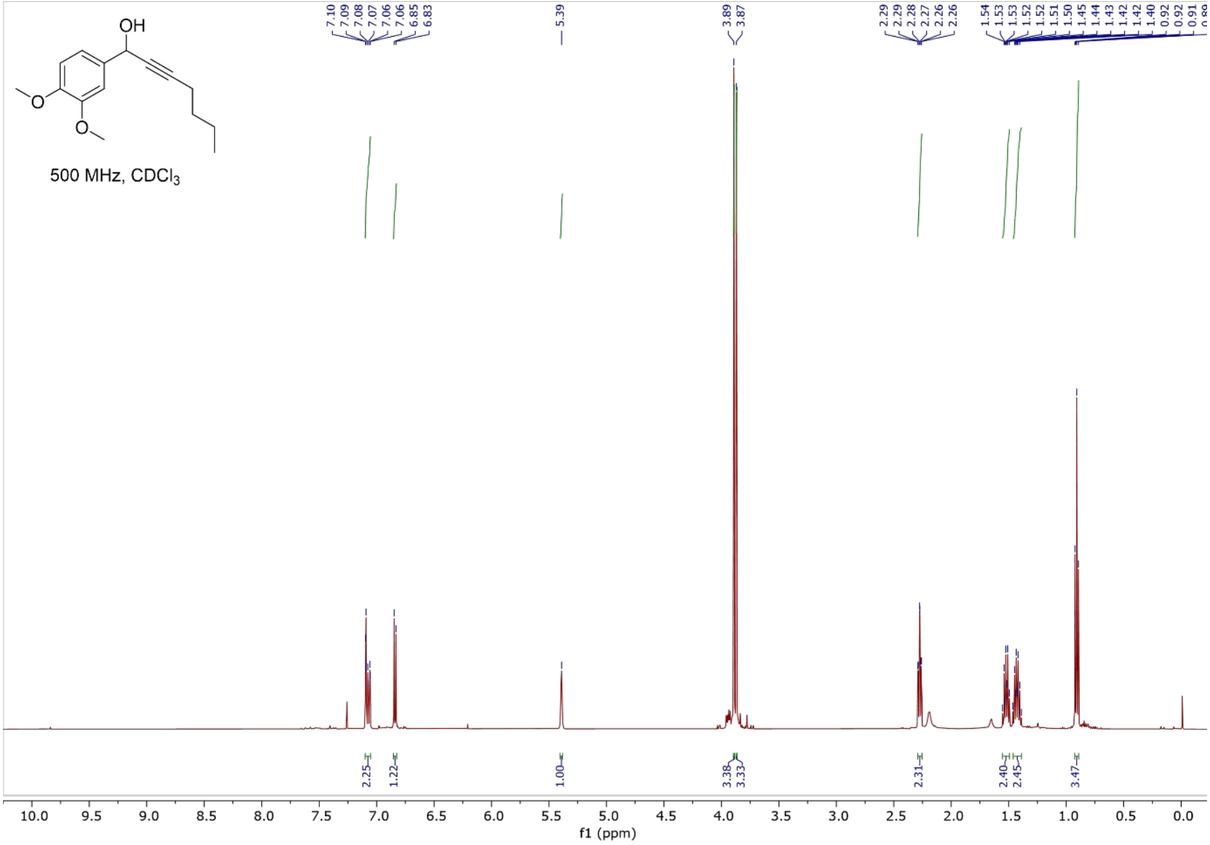

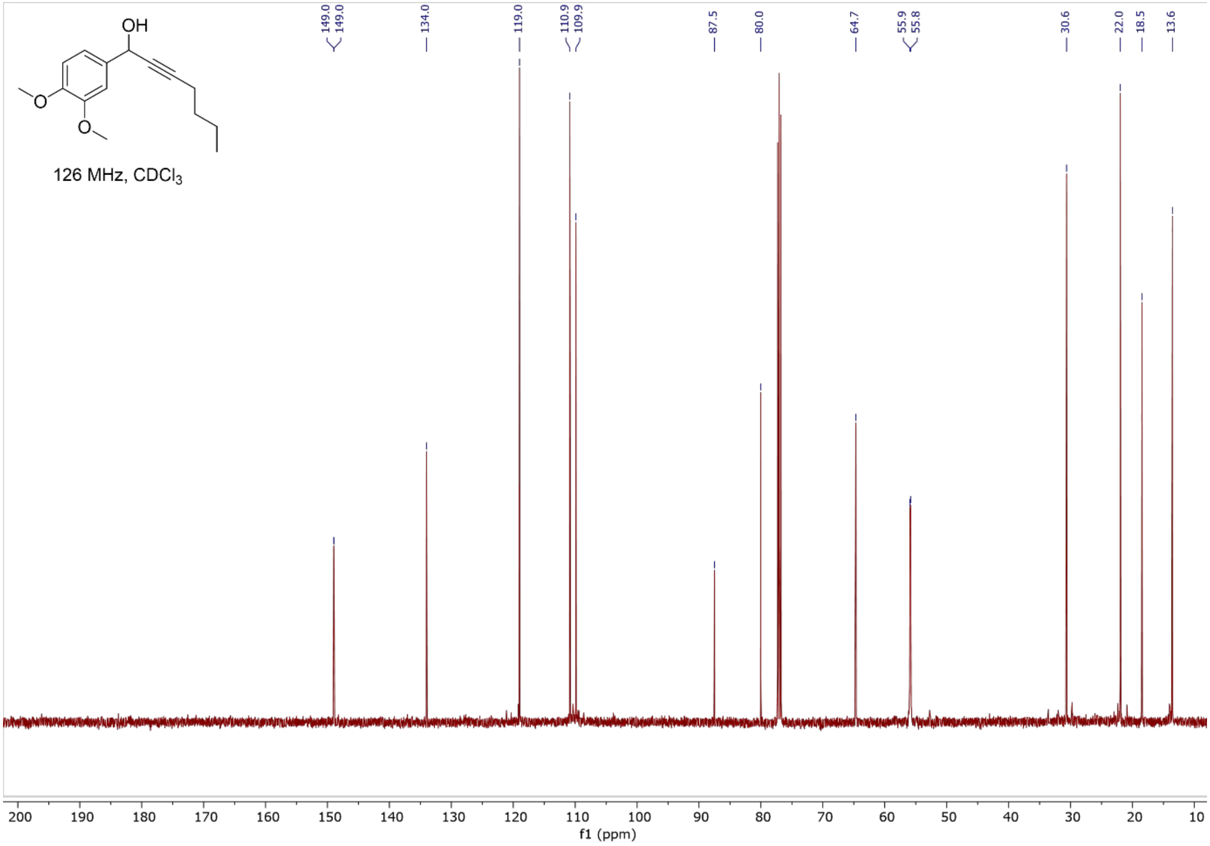

1-(3-Methylthiophen-2-yl)hept-2-yn-1-ol, 9j

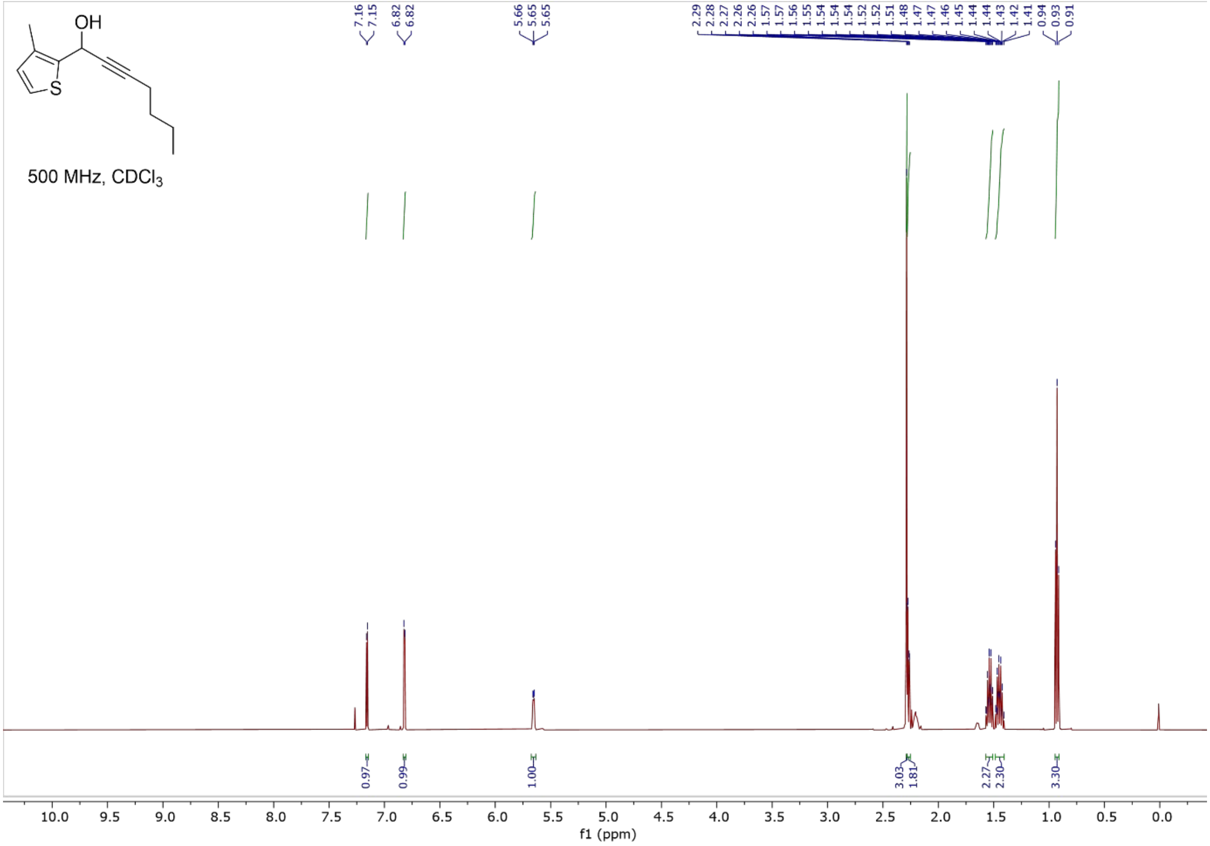

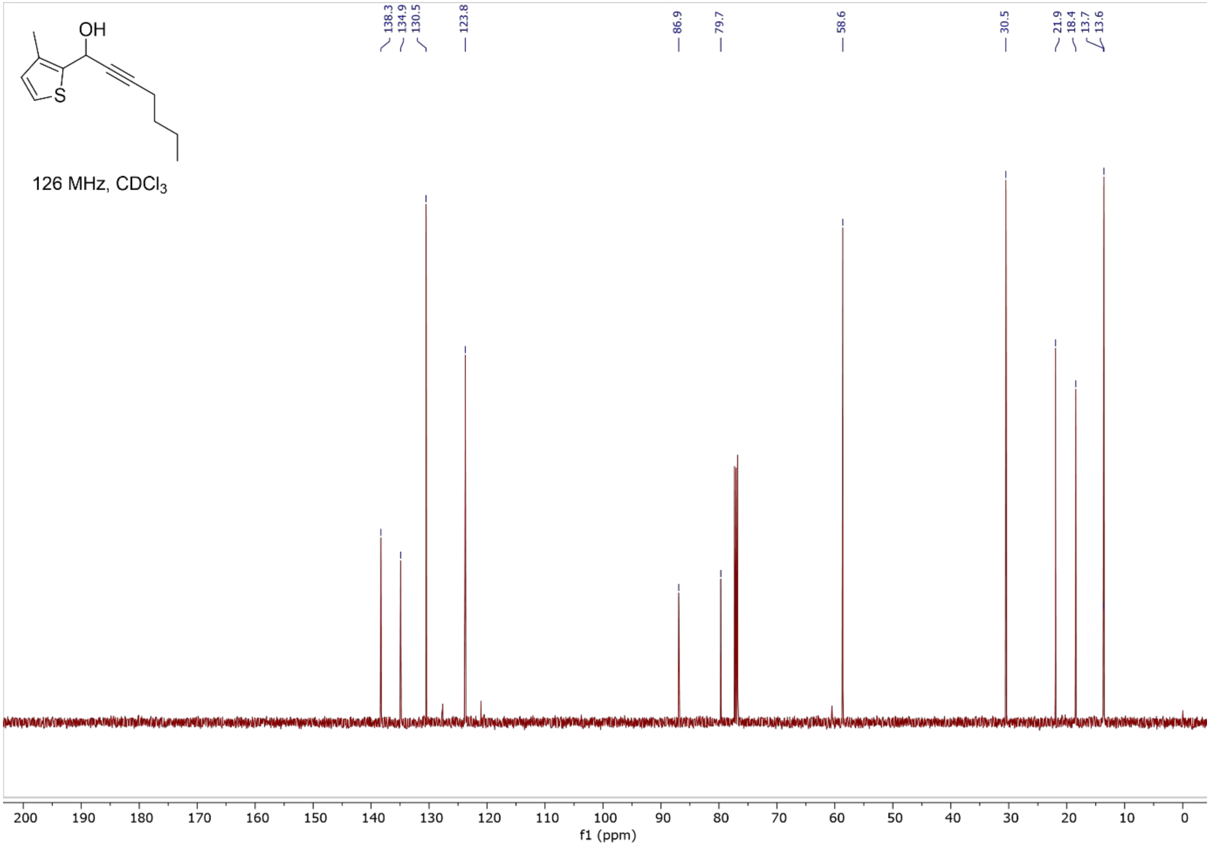

1-(Naphthalen-2-yl)-3-phenylprop-2-yn-1-ol, 9k

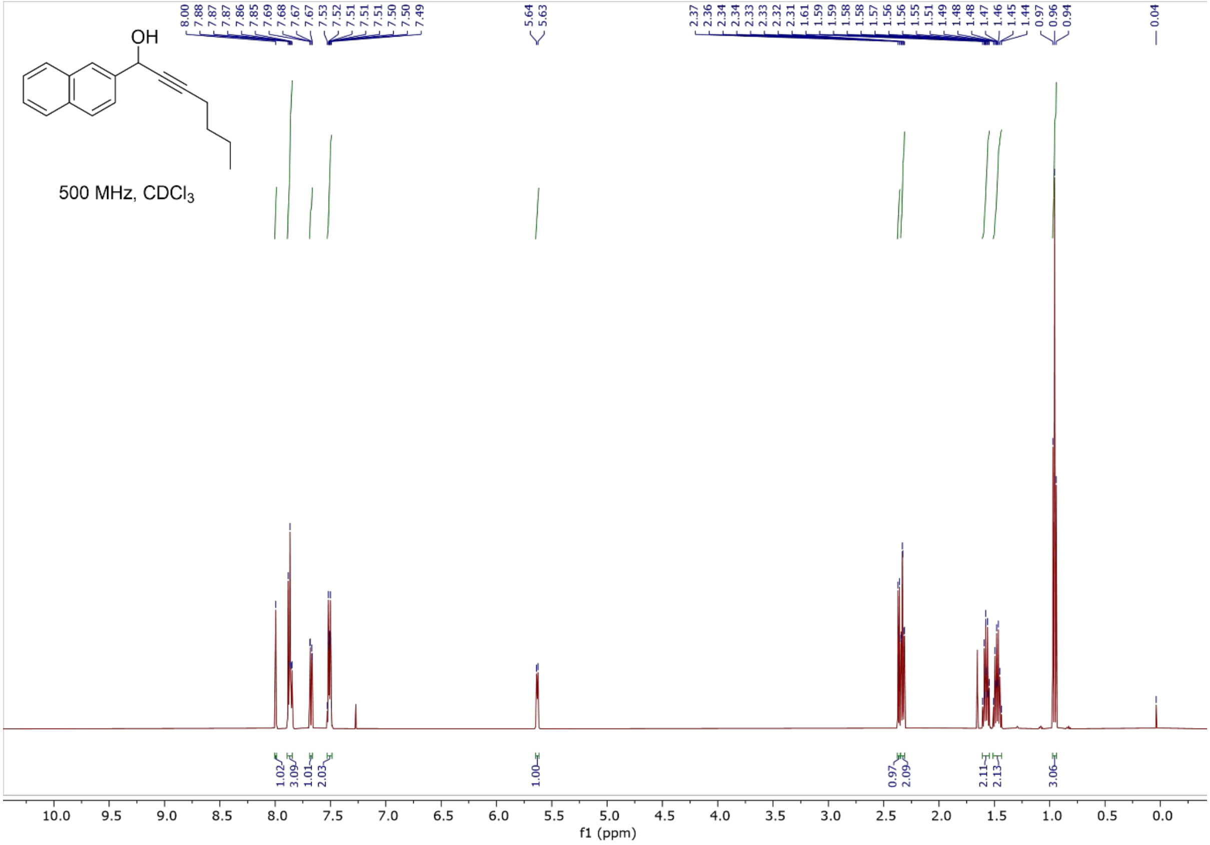

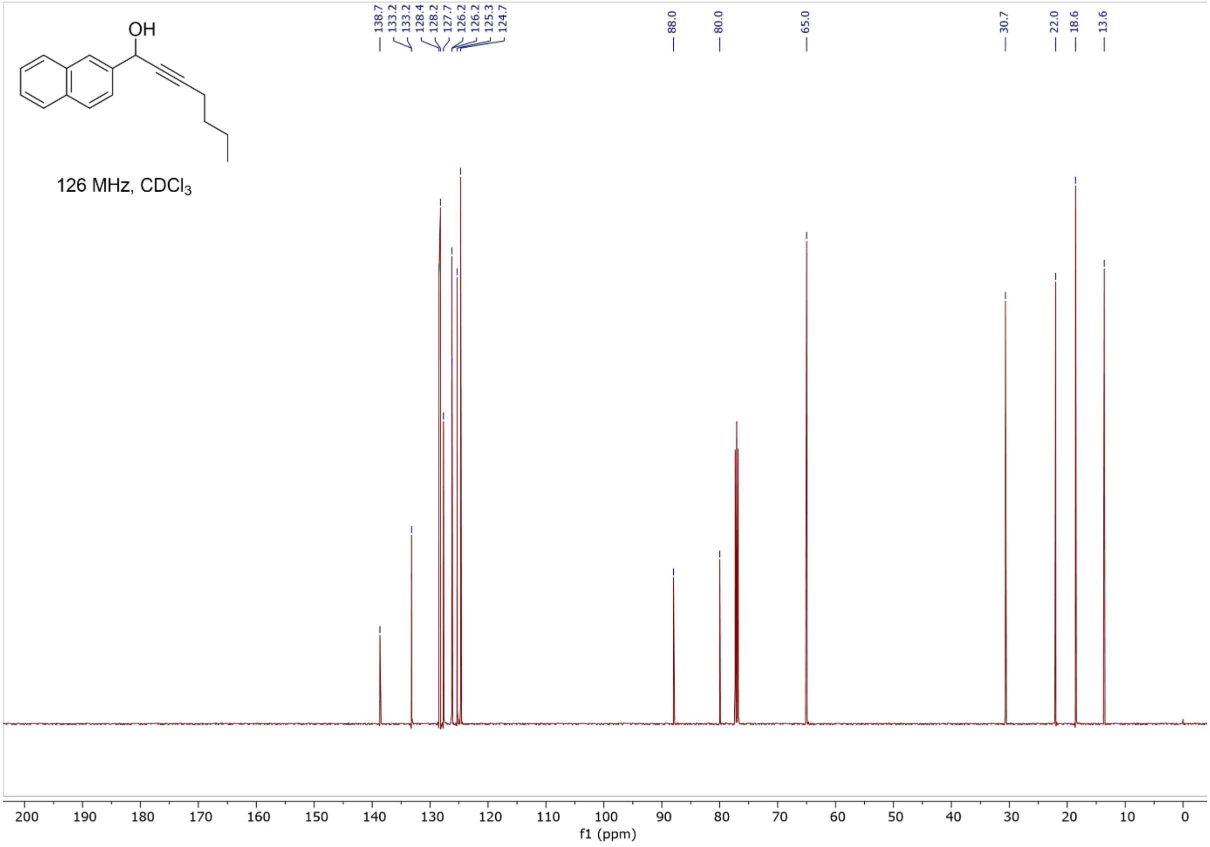

3-Cyclopropyl-1-(2,4-difluorophenyl)prop-2-yn-1-ol, 9l

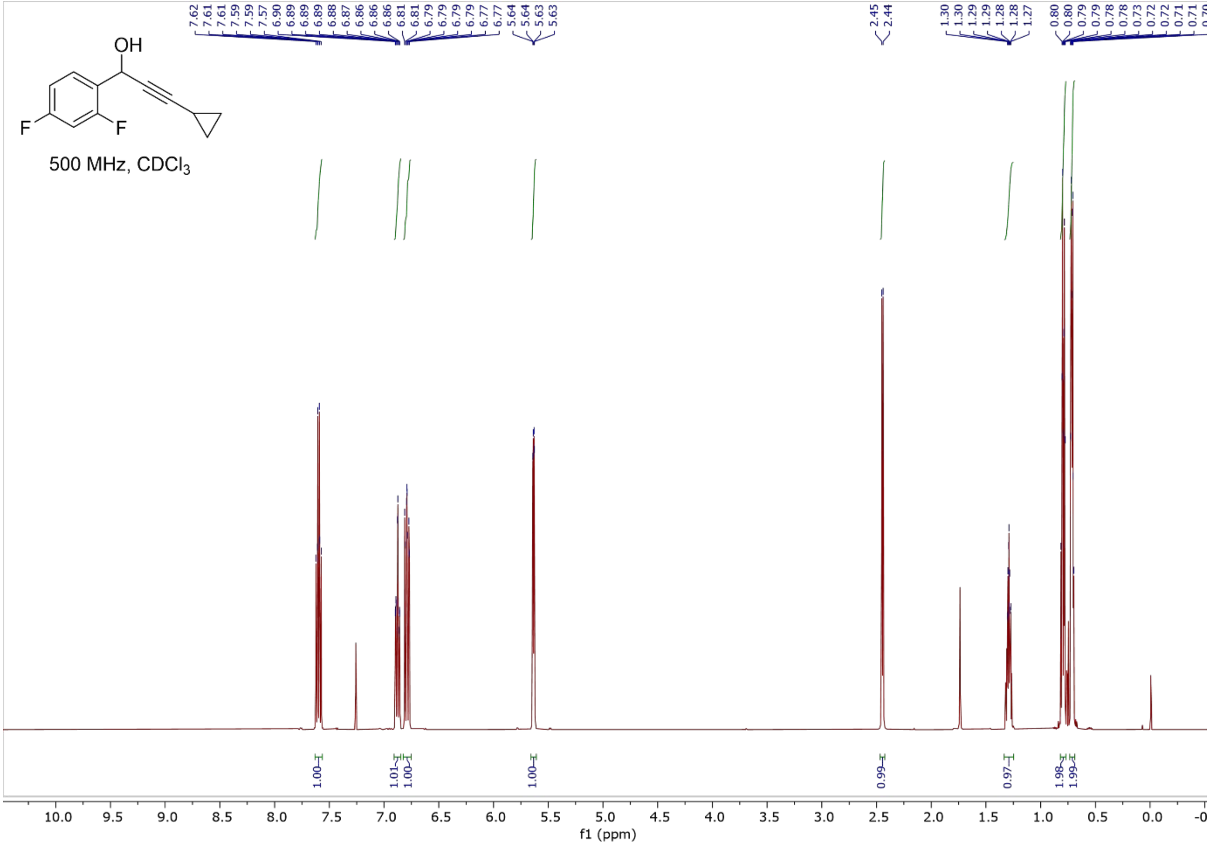

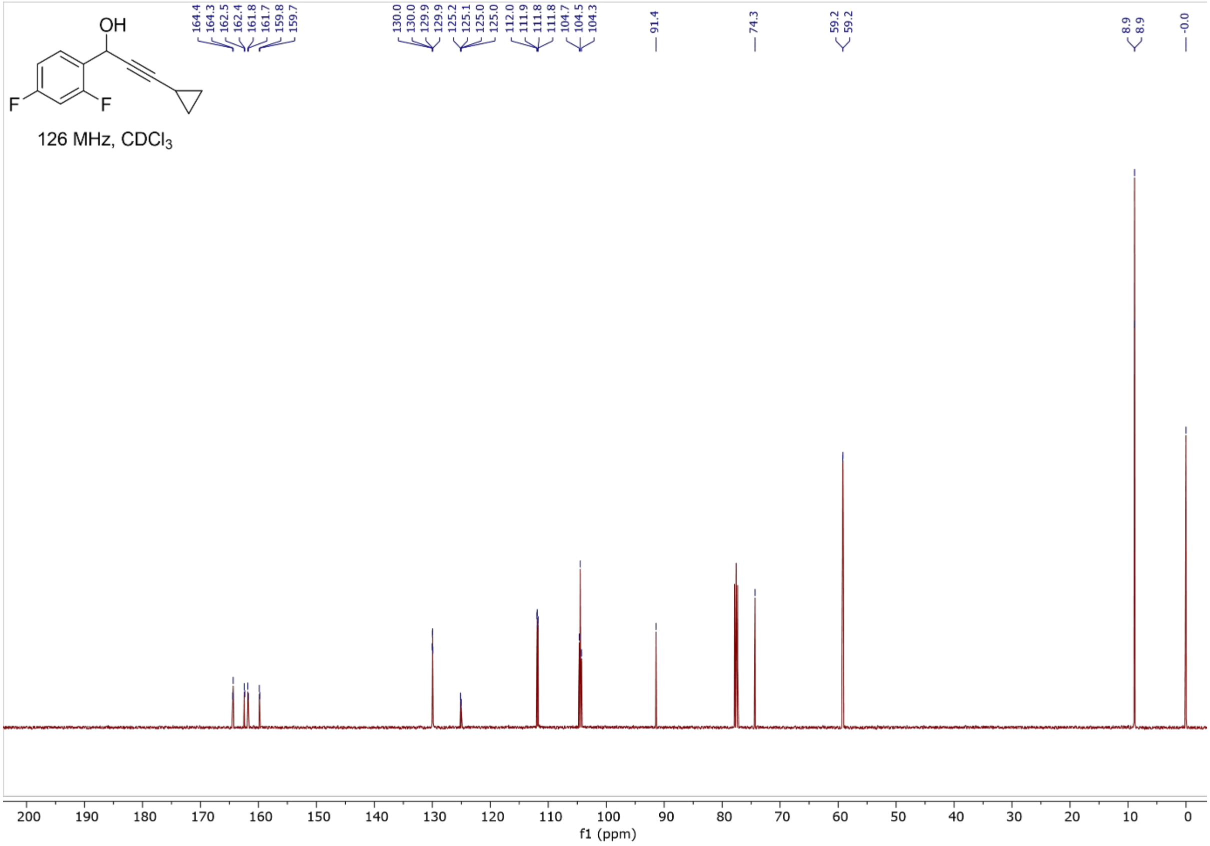

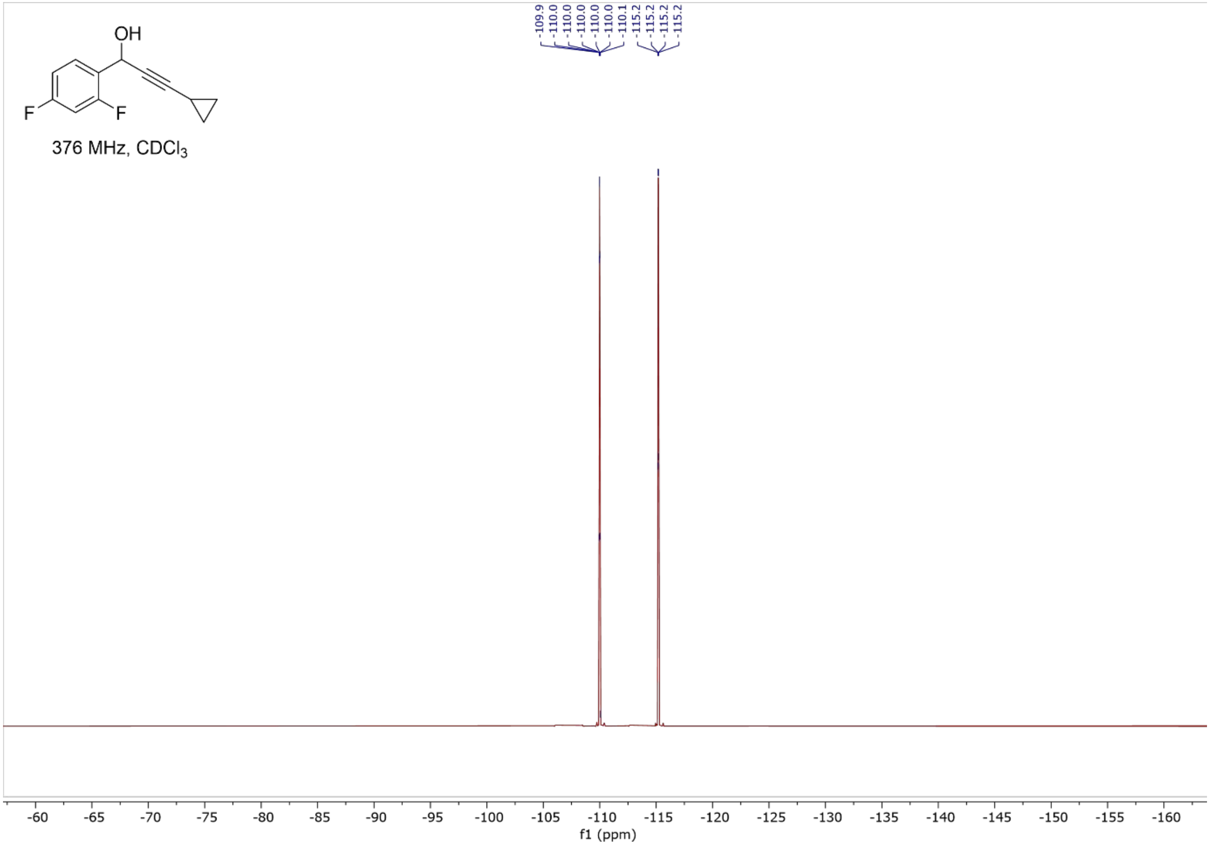

1-(4-Bromophenyl)-3-cyclopropylprop-2-yn-1-ol, 86h, 9m

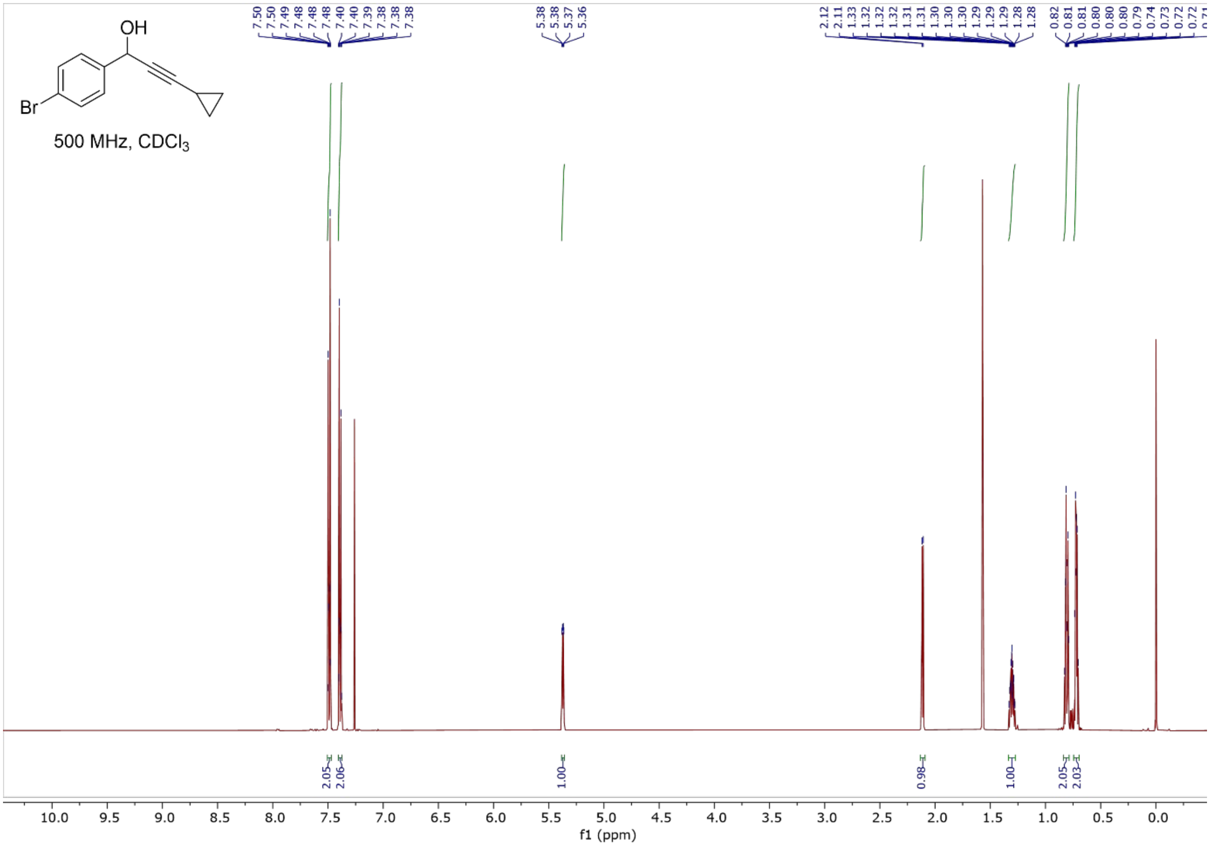

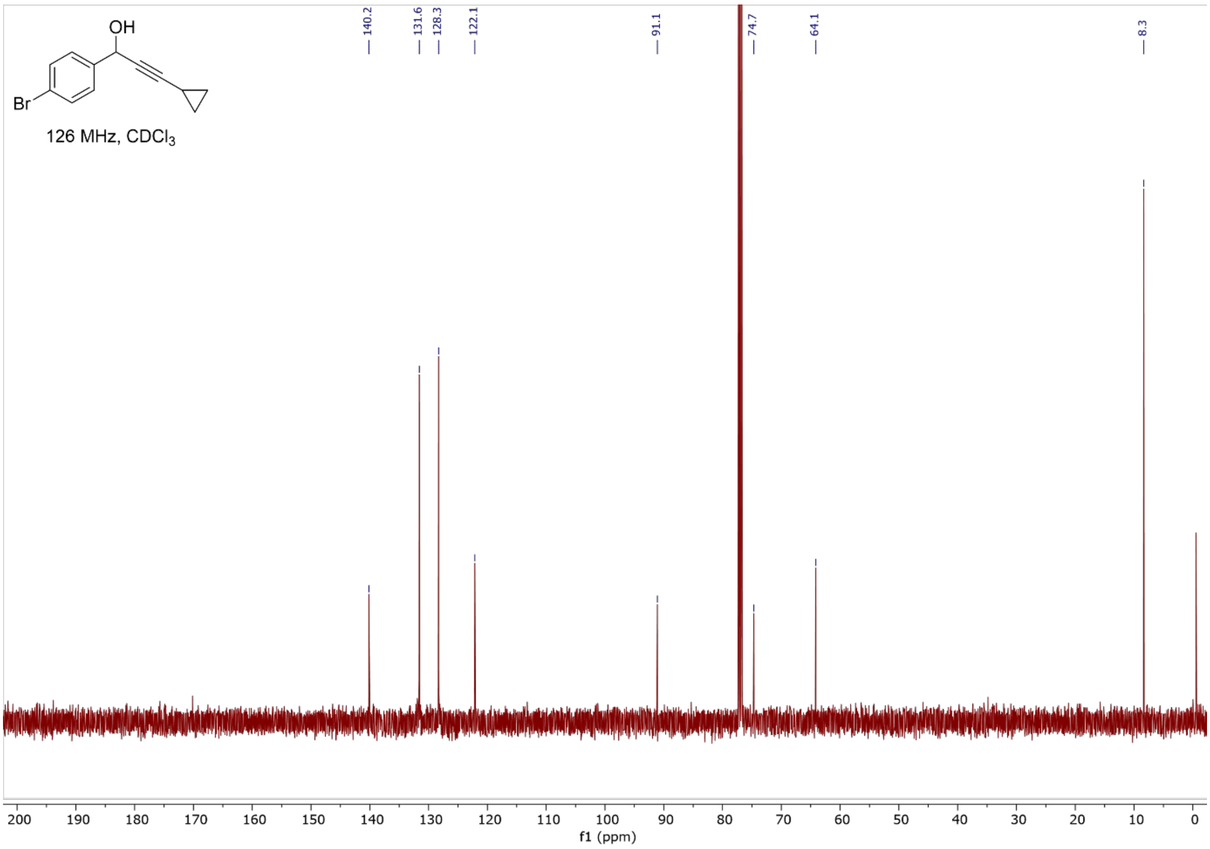

3-Cyclopropyl-1-(naphthalen-2-yl)prop-2-yn-1-ol, 9n

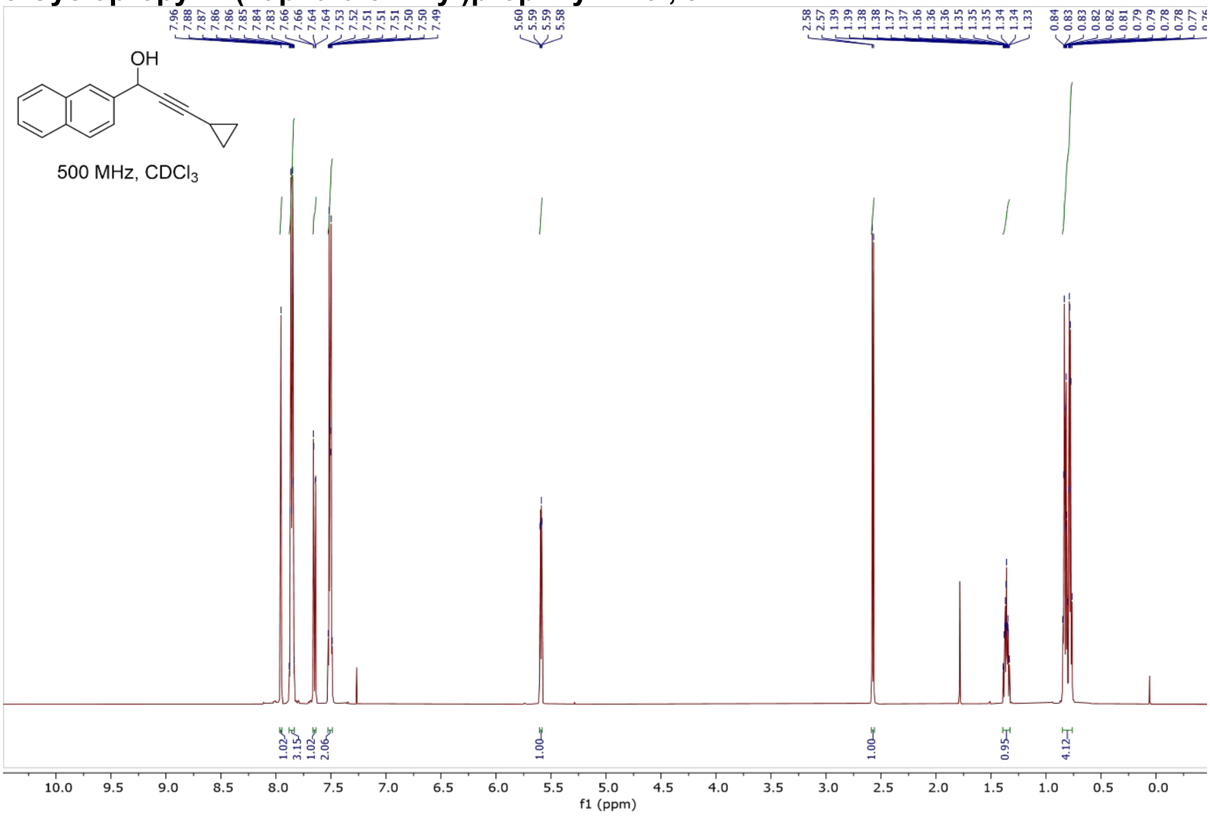

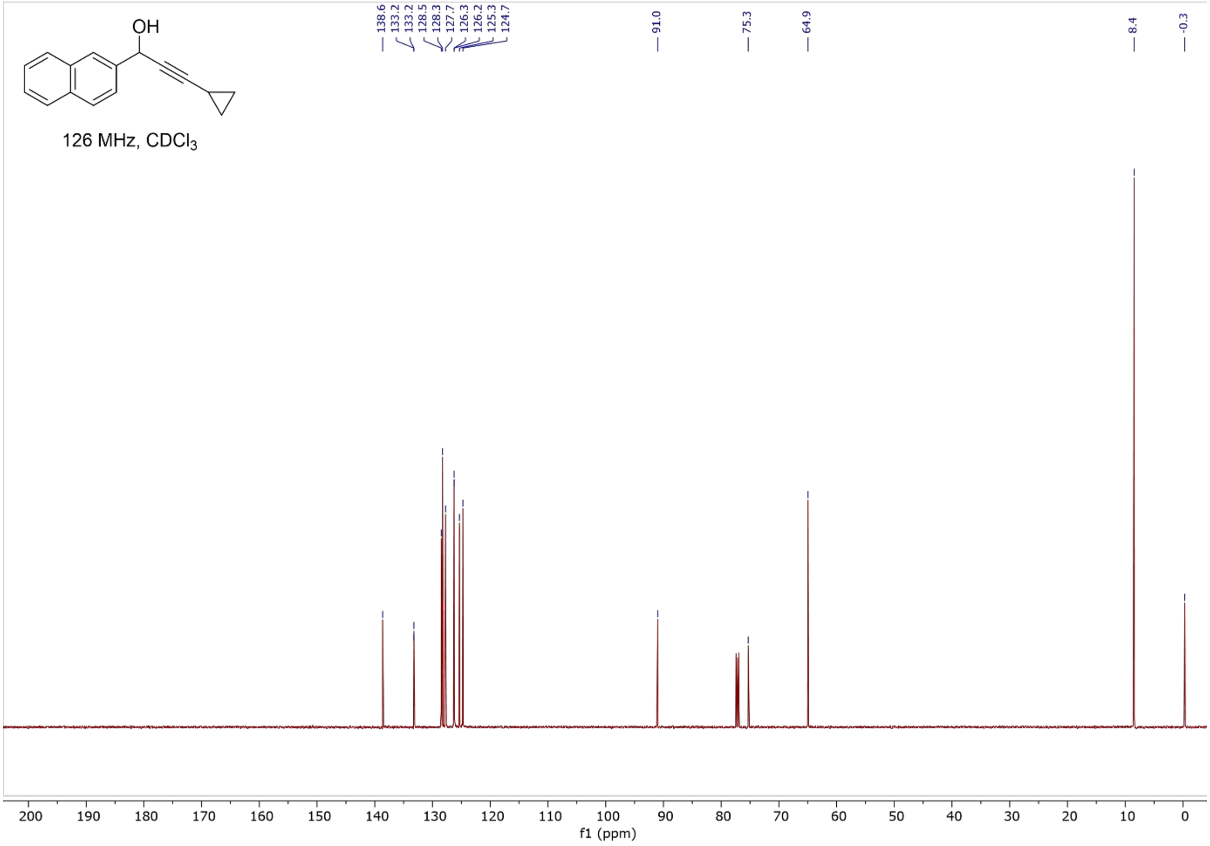

1-(2,4-Difluorophenyl)-3-phenylprop-2-yn-1-ol, 9o

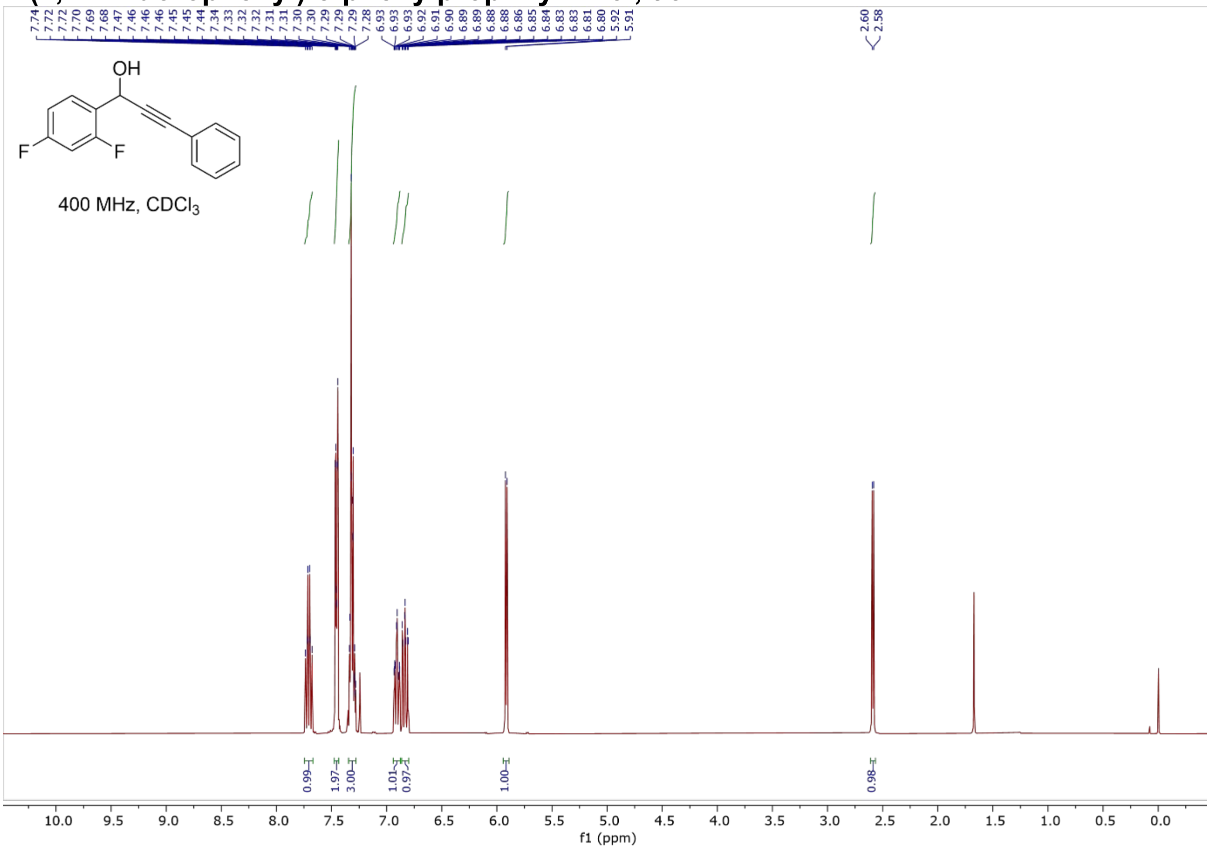

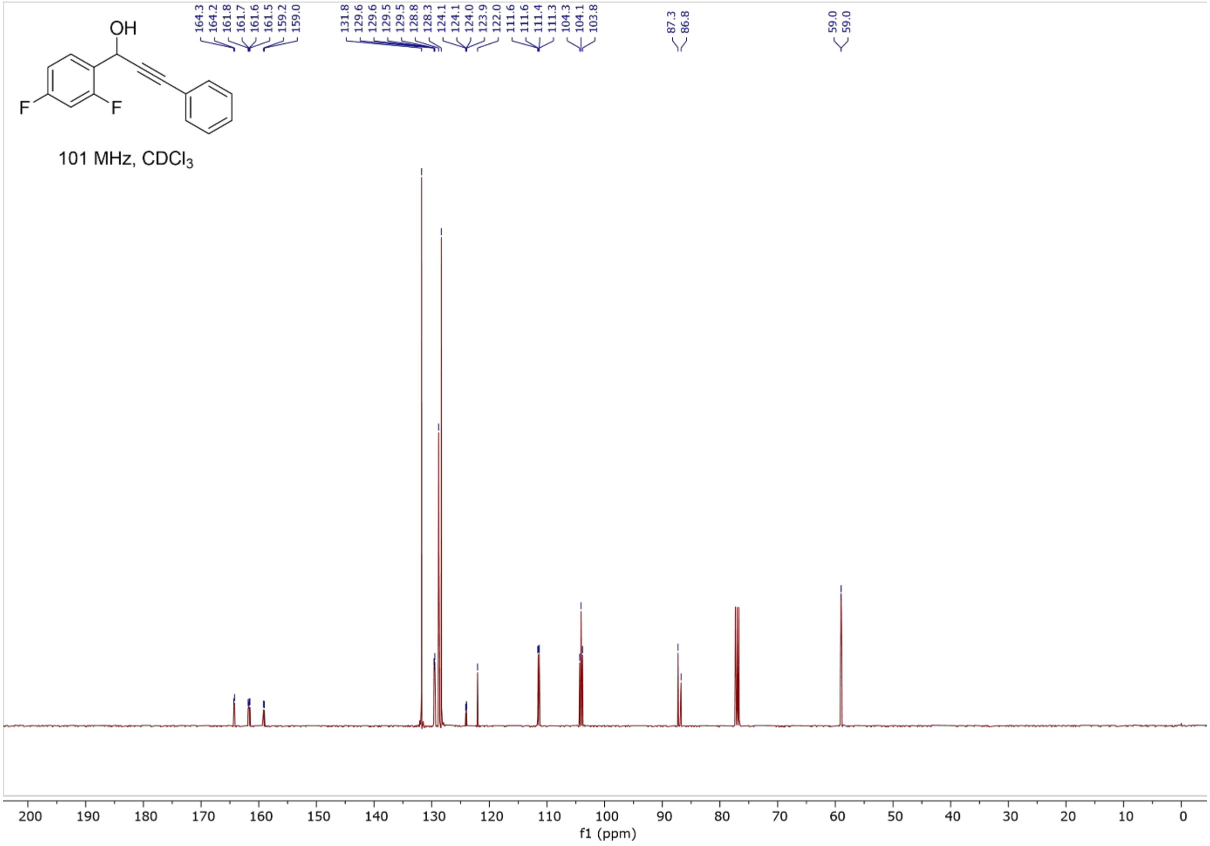
OH
F
F
101 MHz, CDCl3
164.3
164.2
161.8
161.7
161.6
161.5
159.2
159.0
131.8
129.6
129.6
129.5
129.5
128.8
128.3
124.1
124.1
124.0
123.9
122.0
111.6
111.6
111.4
111.3
104.3
104.1
103.8
87.3
86.8
59.0
59.0
f1 (ppm)

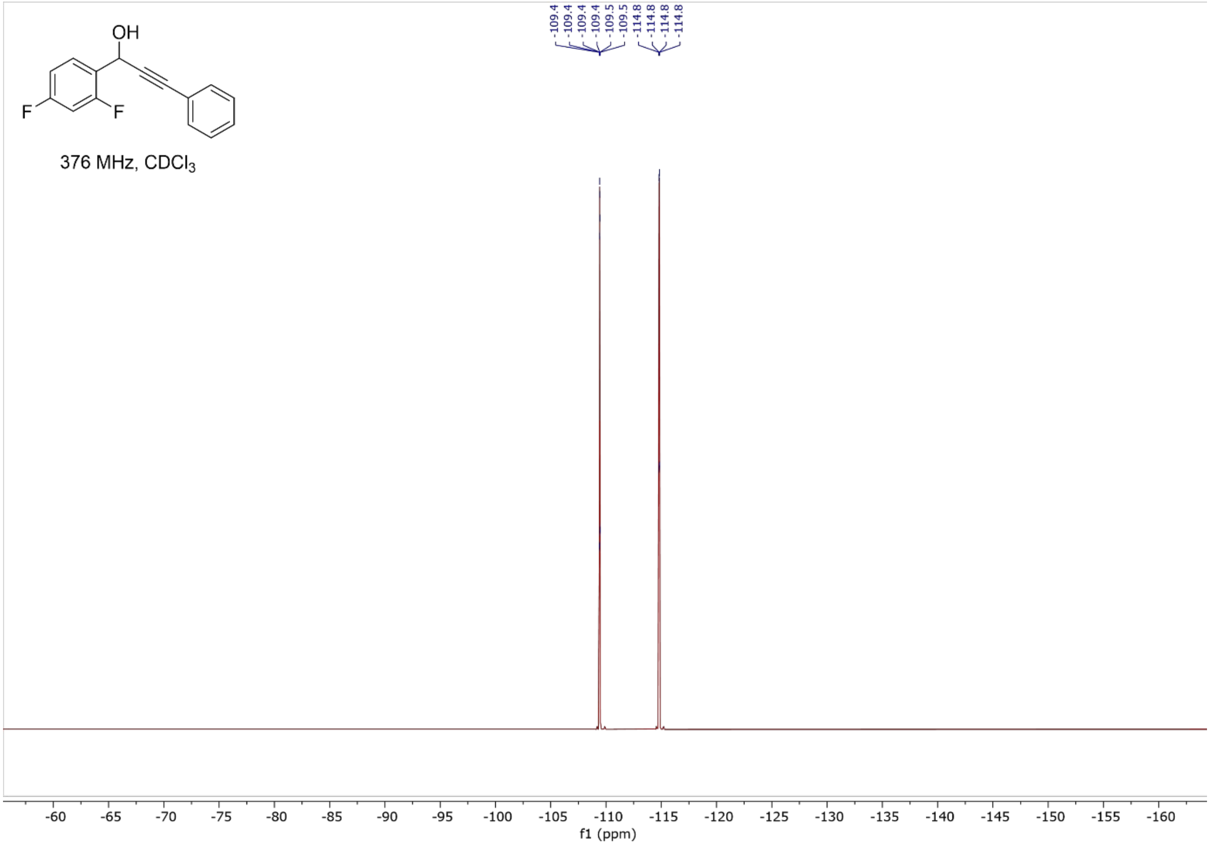
OH
F
F
376 MHz, CDCl3
-109.4
-109.4
-109.4
-109.5
-109.5
-114.8
-114.8
-114.8
-114.8
f1 (ppm)

1-(4-Fluorophenyl)-3-phenylprop-2-yn-1-ol, 9p

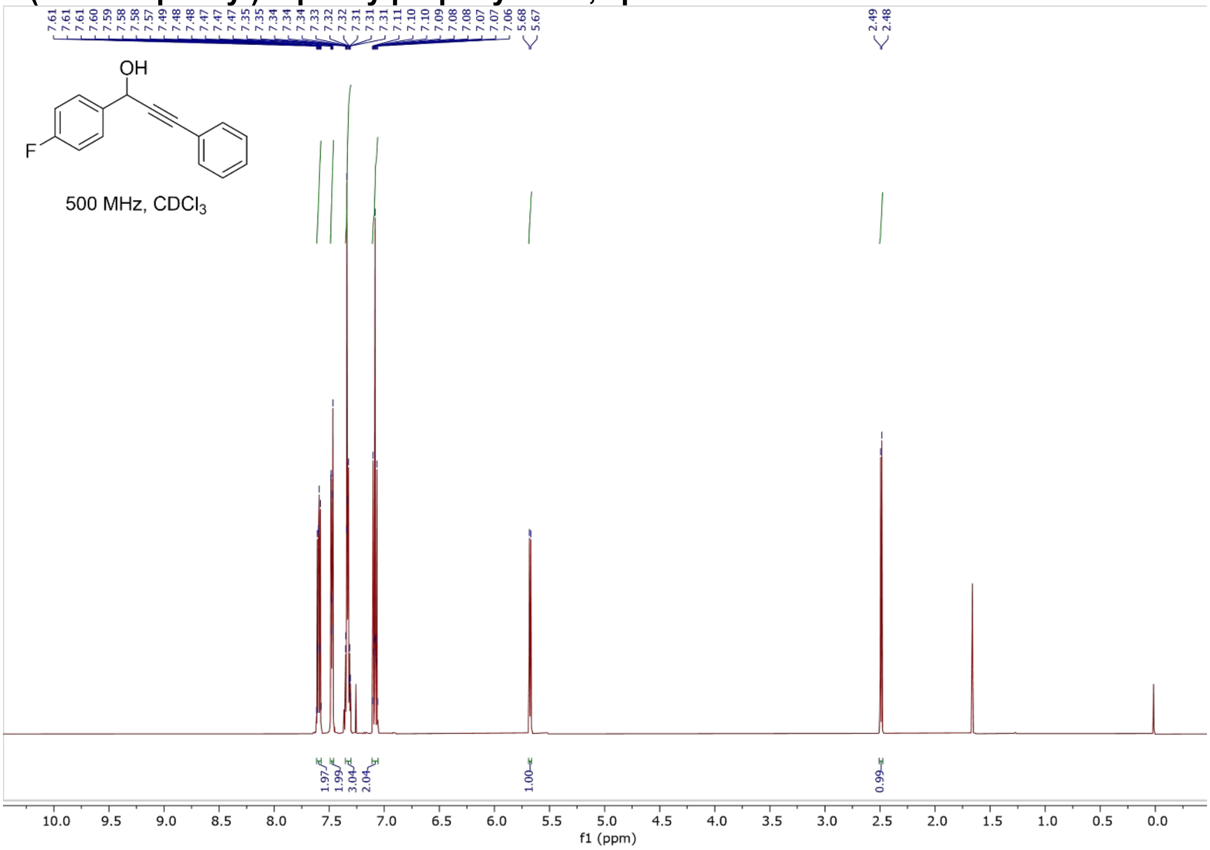

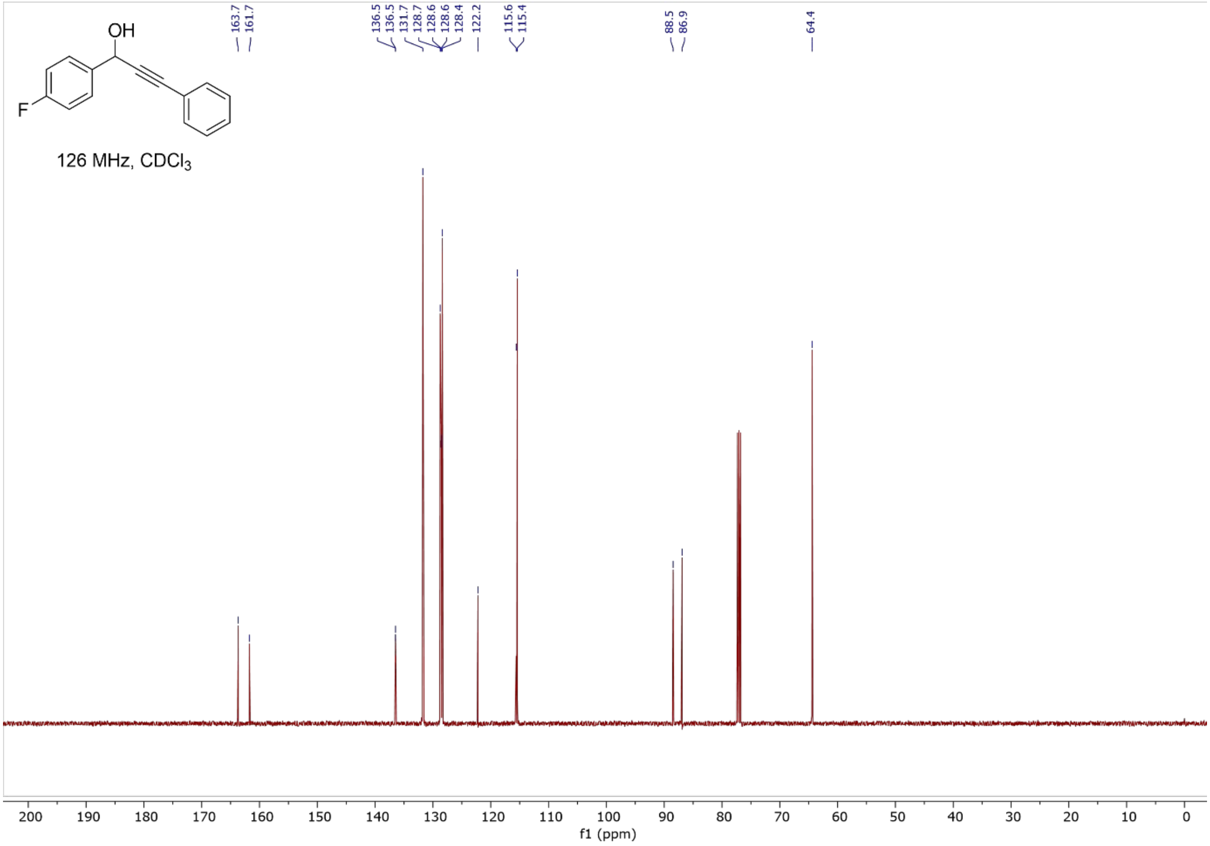

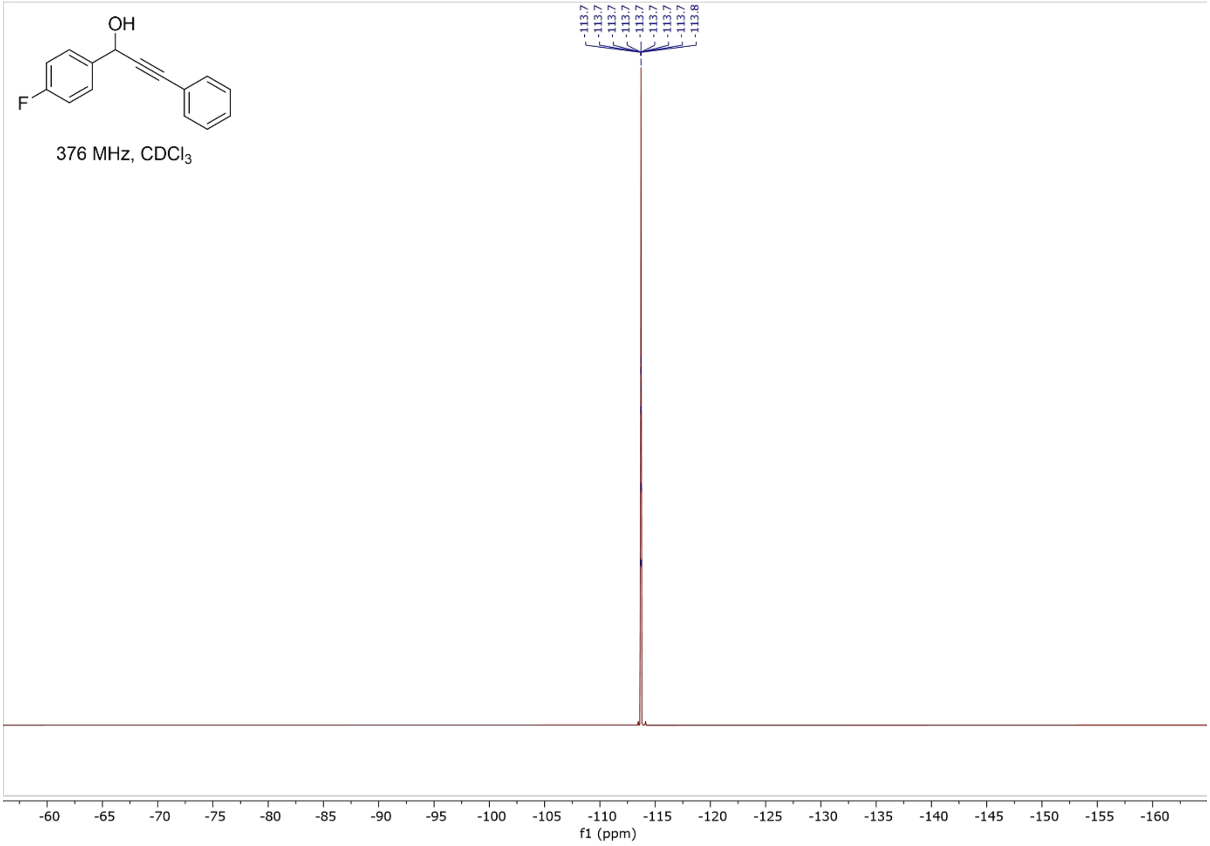

1-(4-Bromophenyl)-3-phenylprop-2-yn-1-ol, 9q

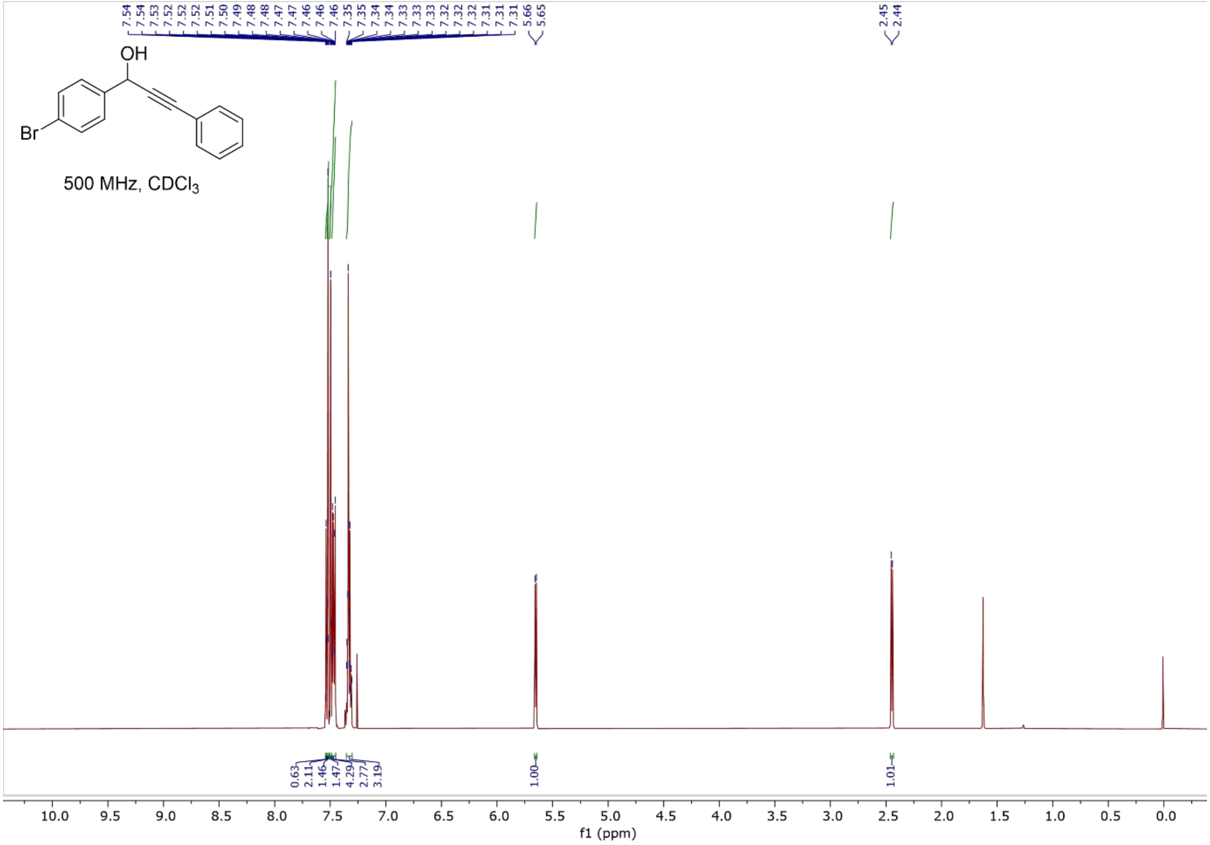

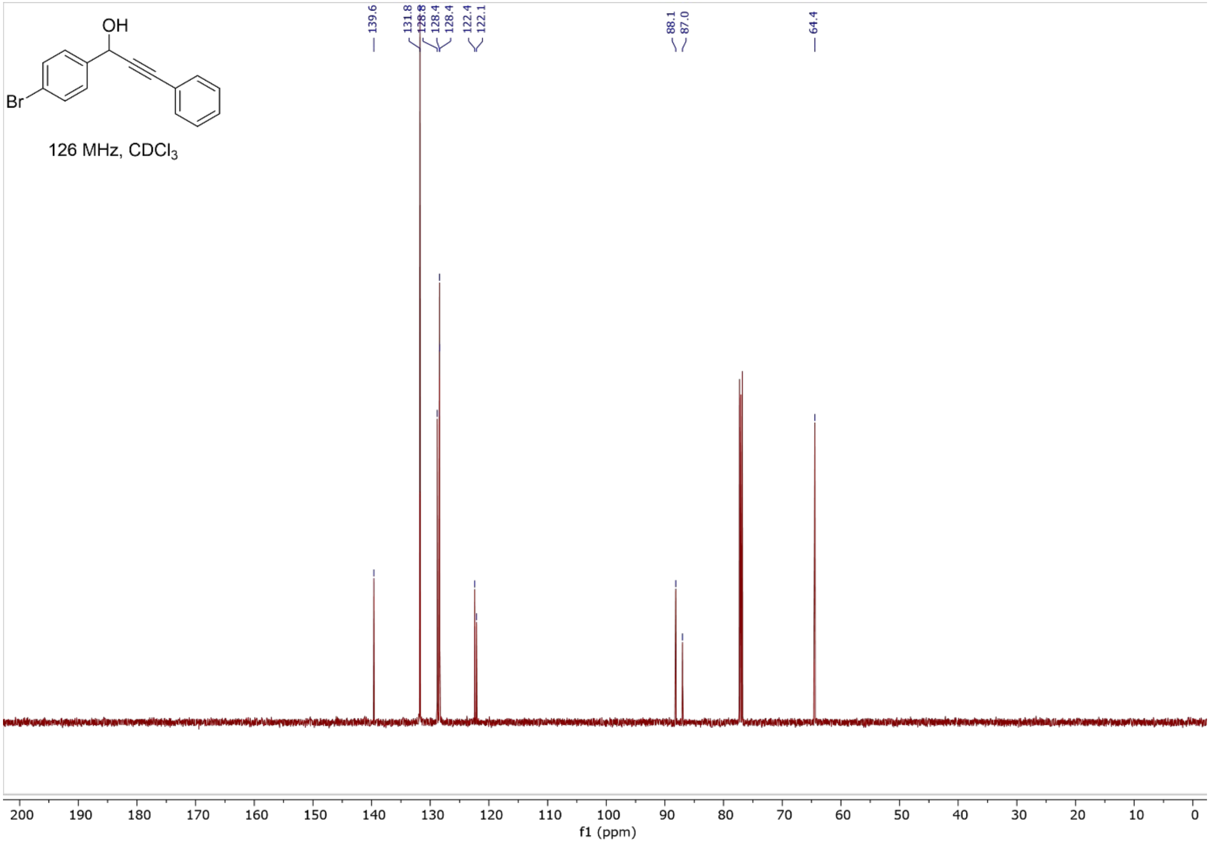

1-(Naphthalen-2-yl)-3-phenylprop-2-yn-1-ol, 9r

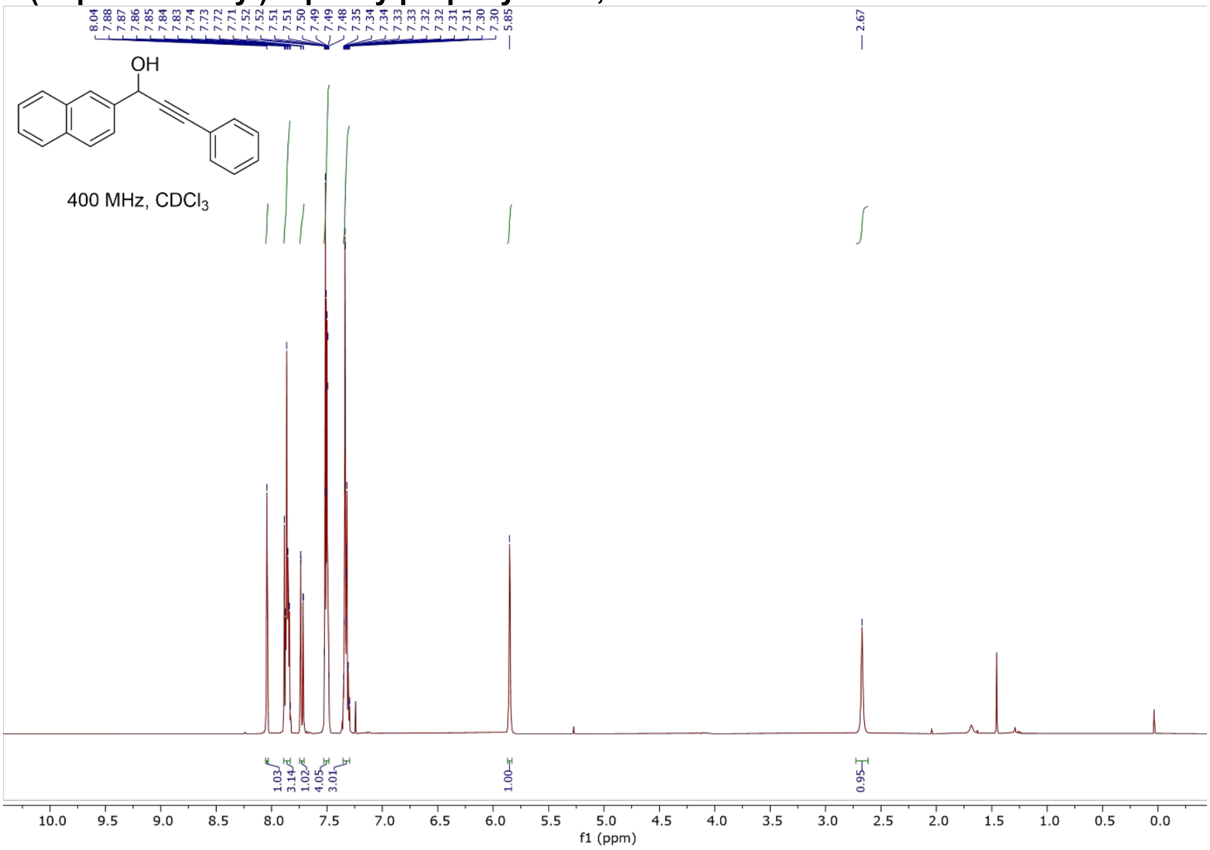

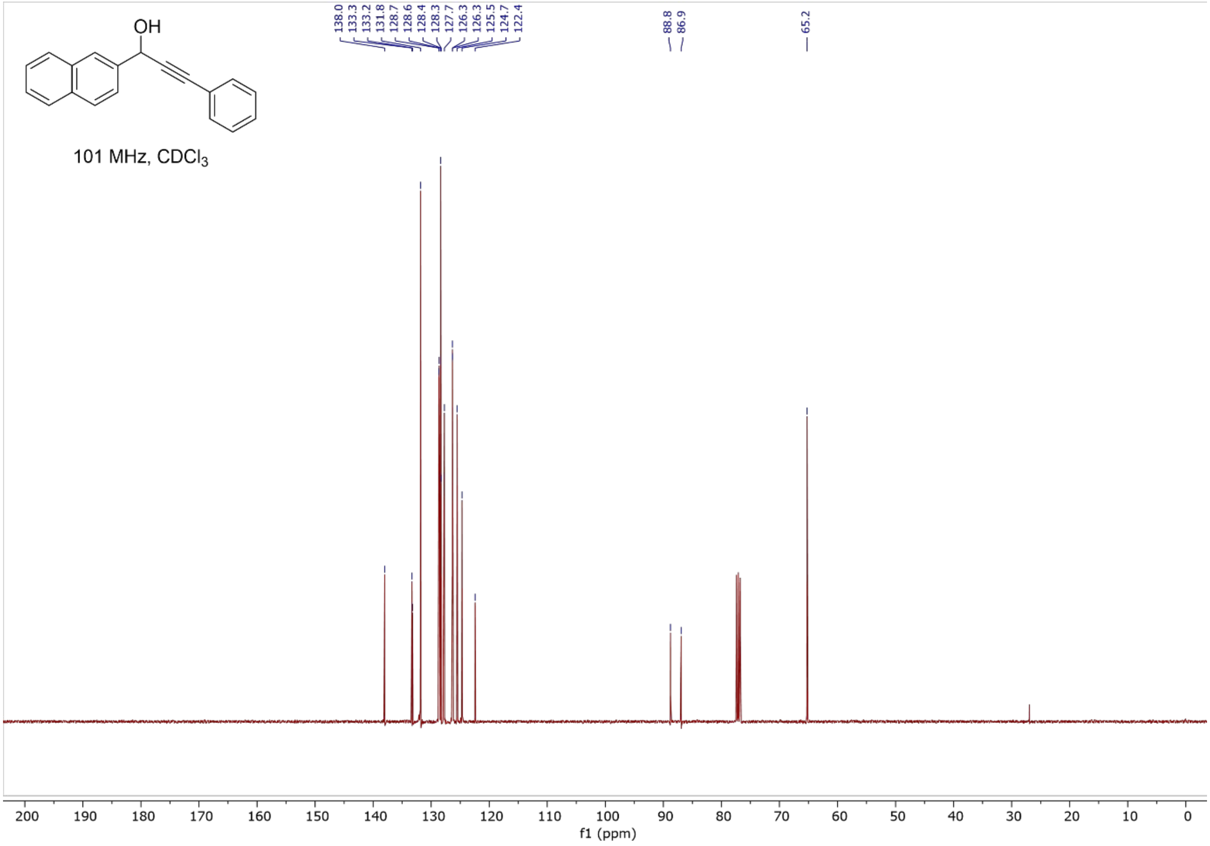

4-(1-Hydroxyhept-2-yn-1-yl)benzonitrile, 9s

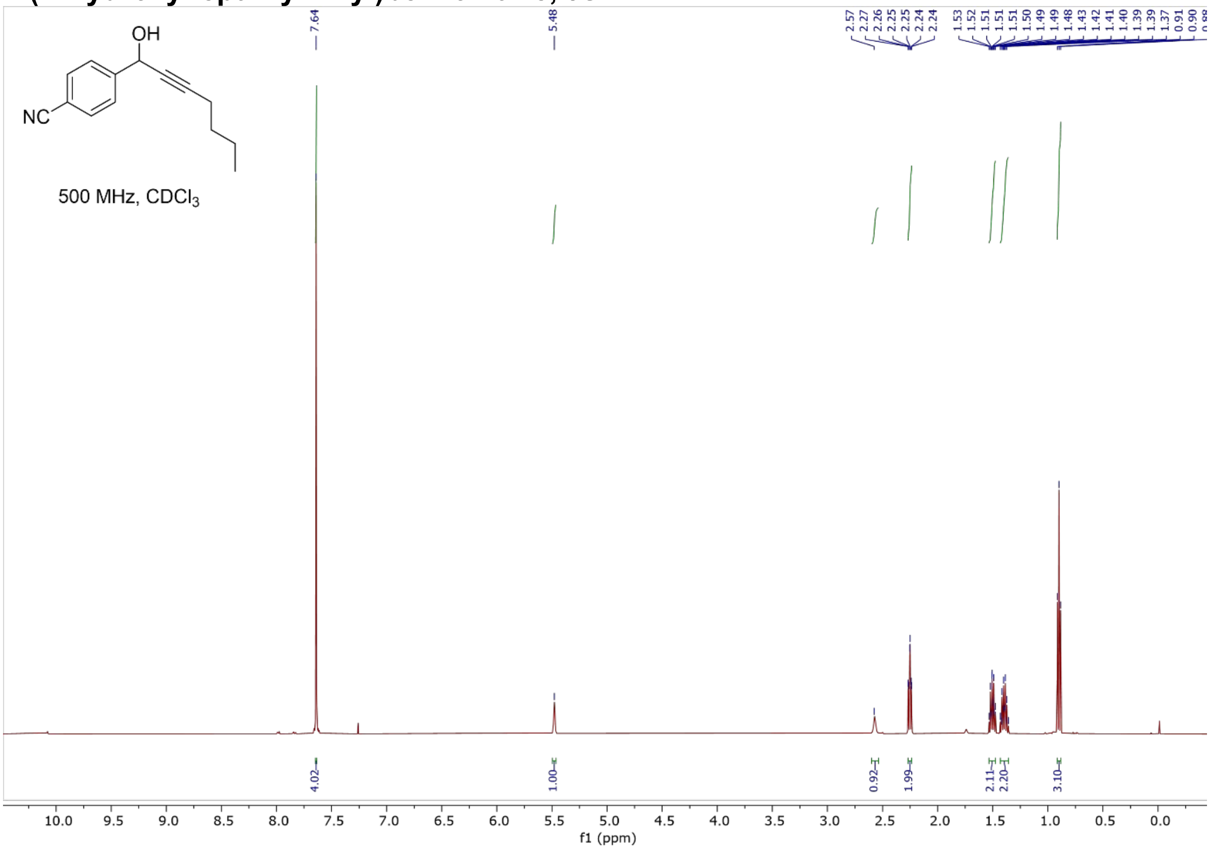

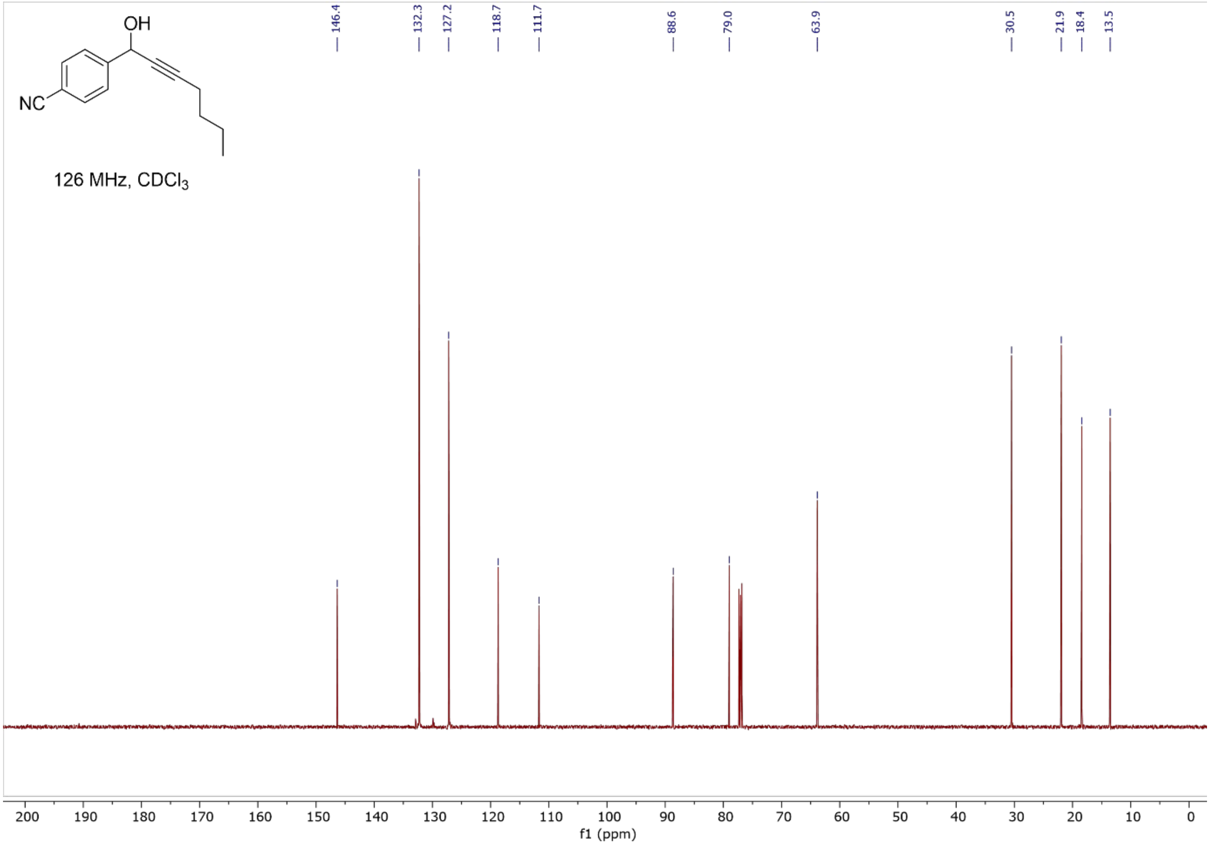

1-(Pyridin-3-yl)hept-2-yn-1-ol, 9t

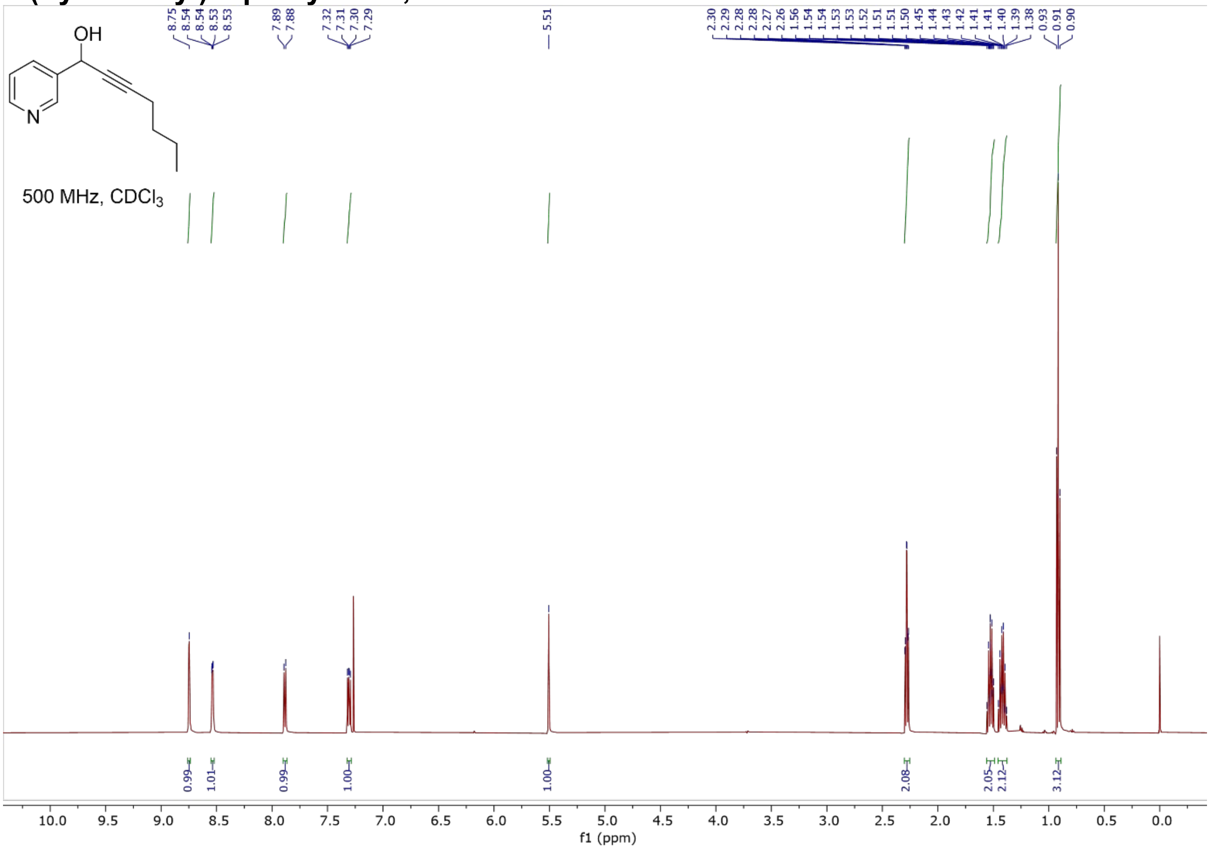

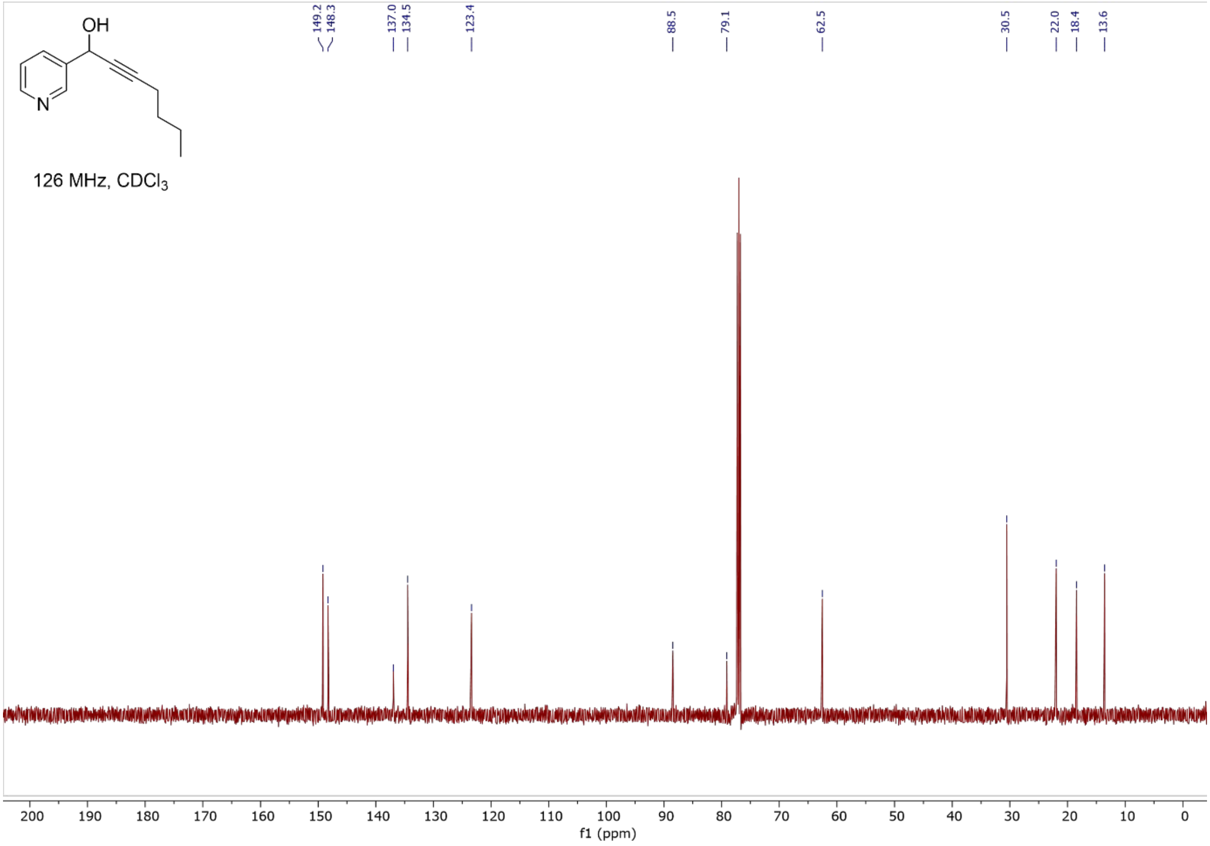

1-Cyclohexylhept-2-yn-1-ol, 9u

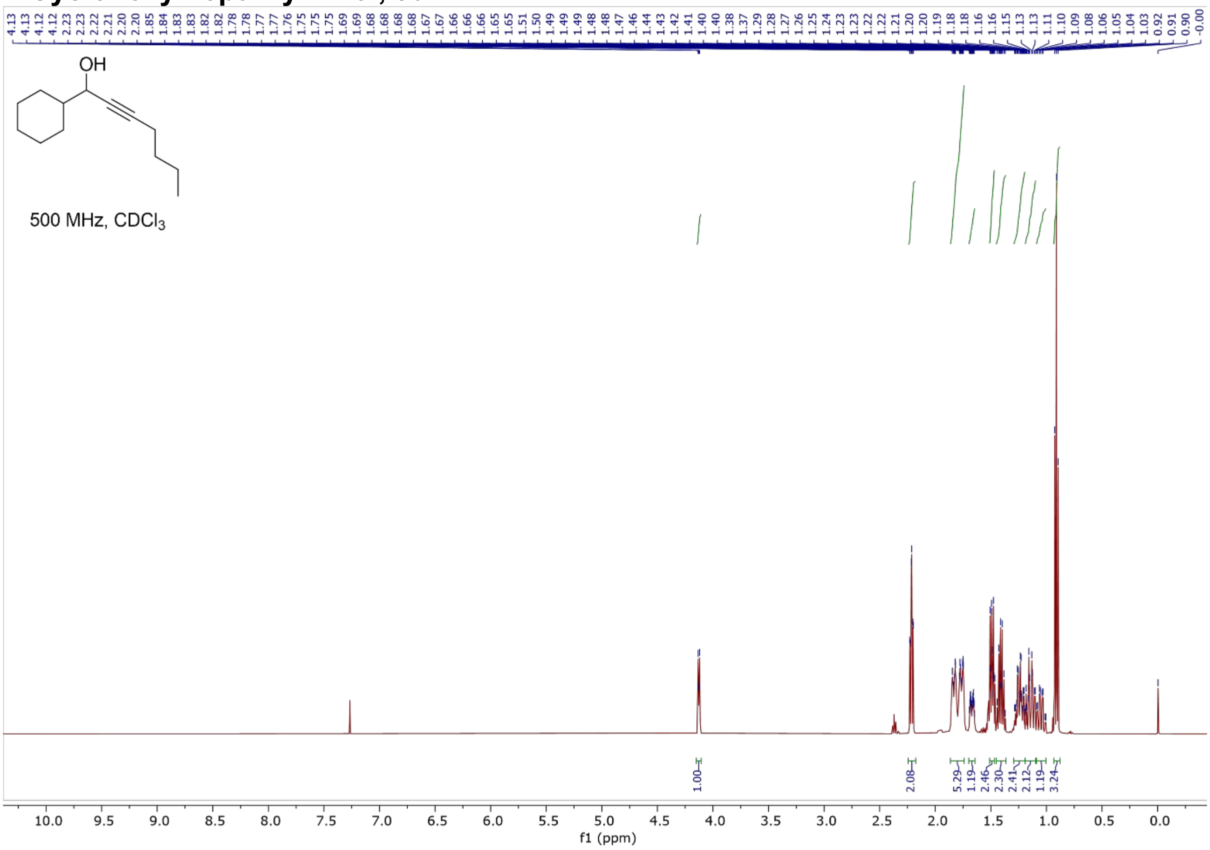

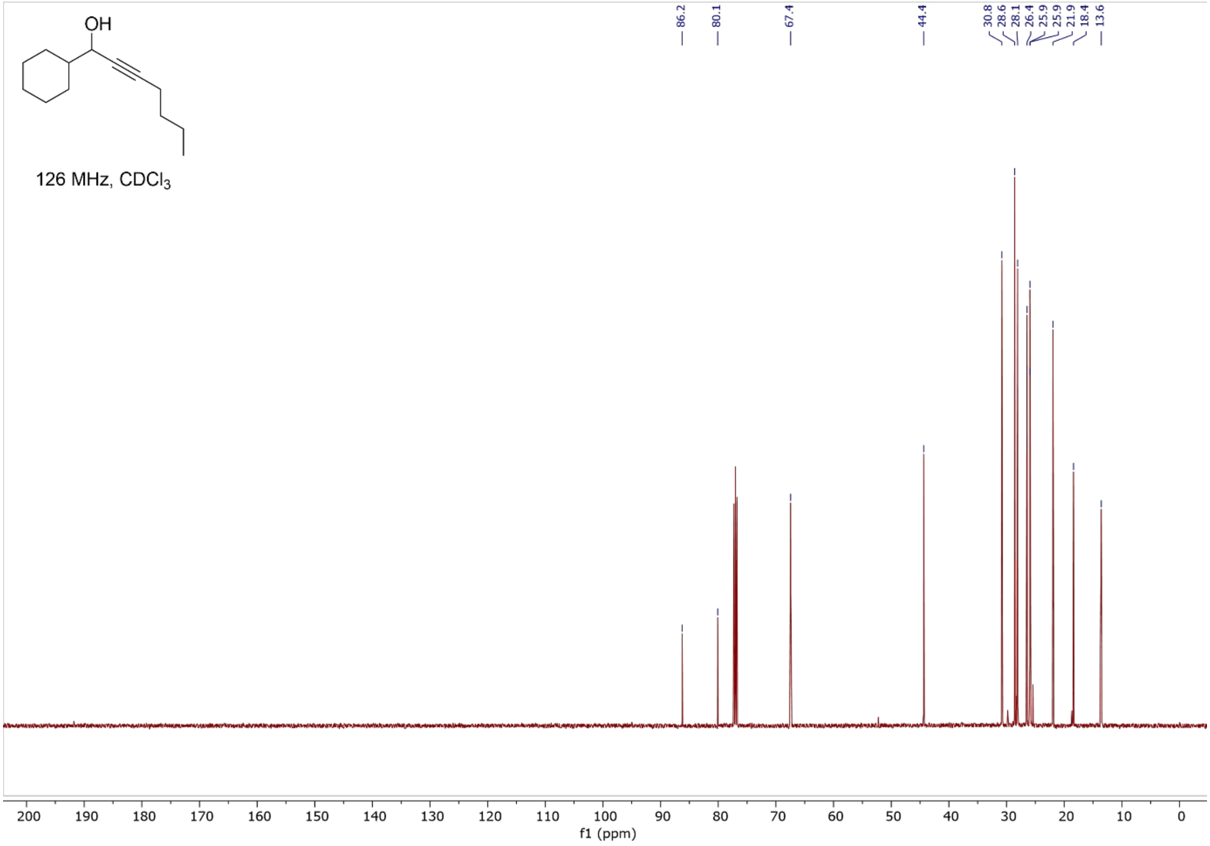

Undec-6-yn-5-ol, 9v

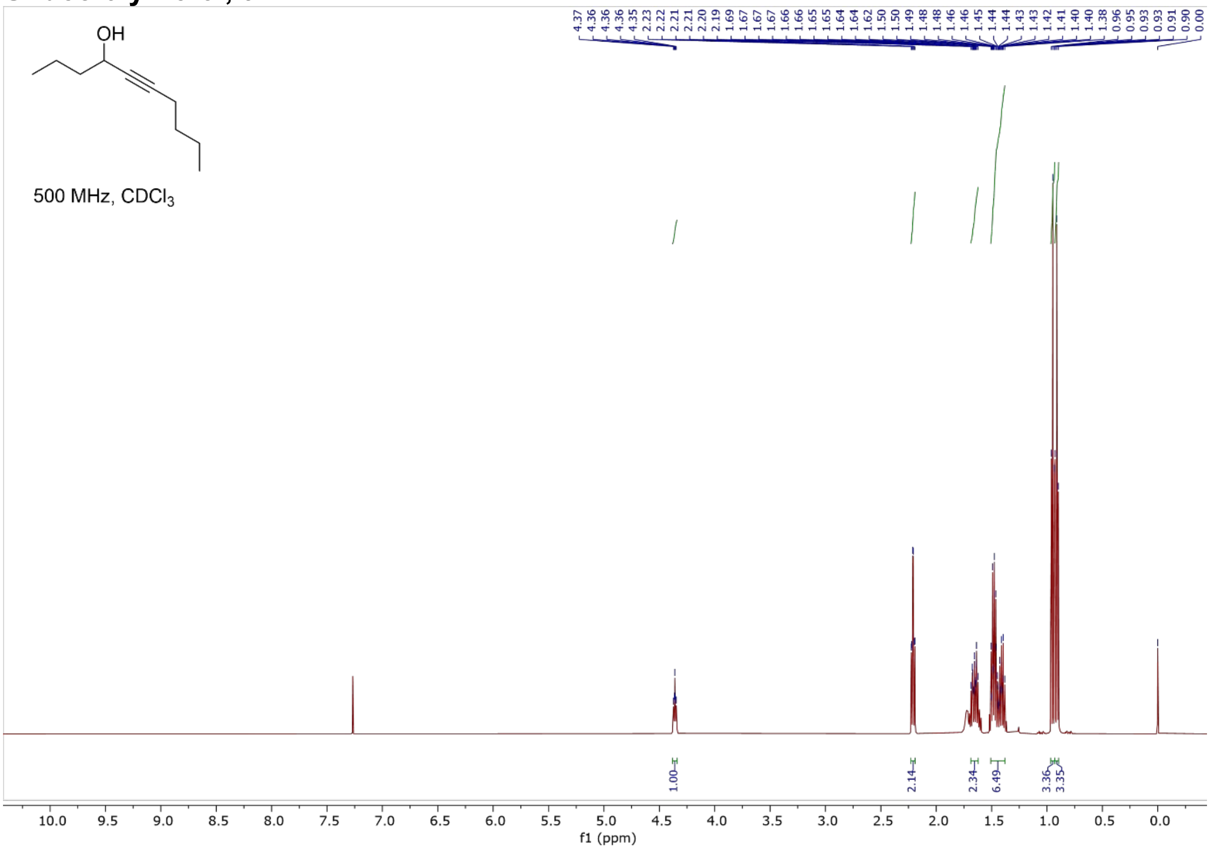

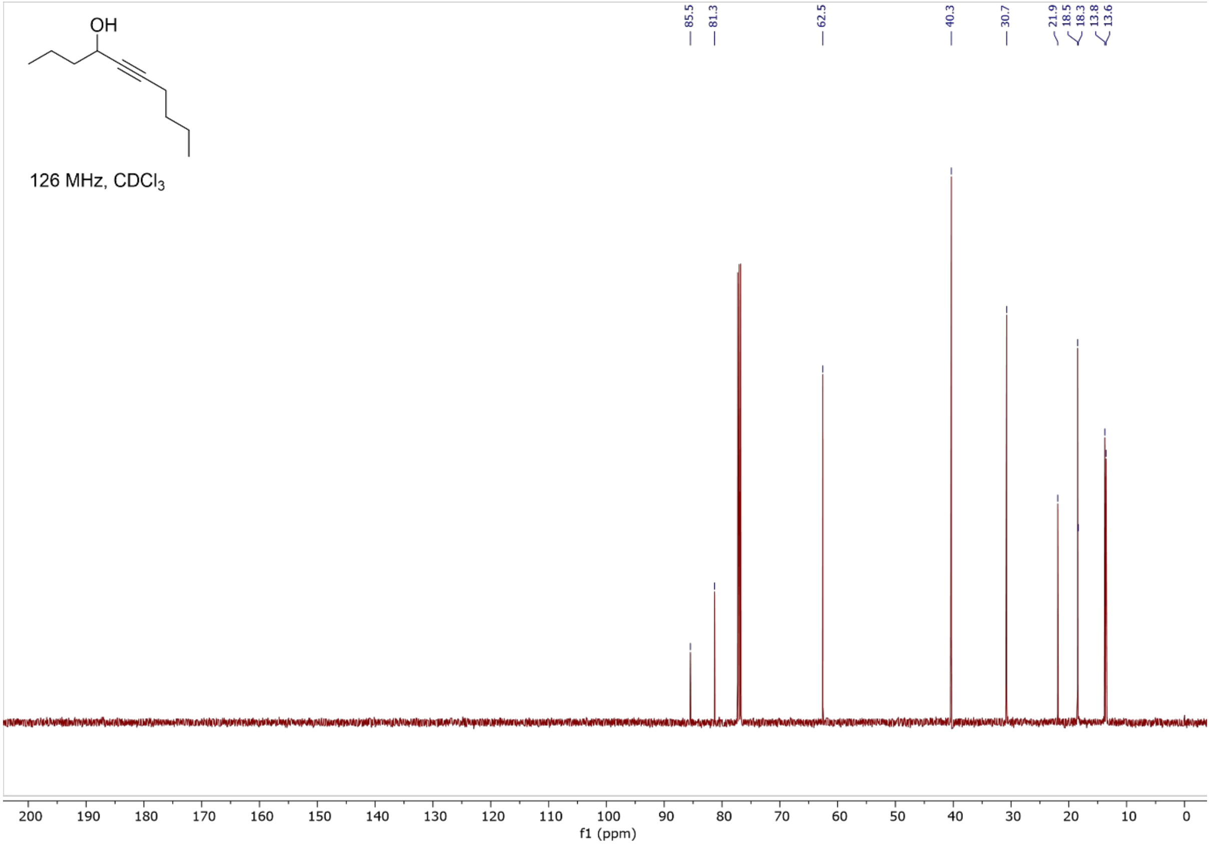

***N*-hydroxy-4-methylbenzenesulfonamide, 10a**

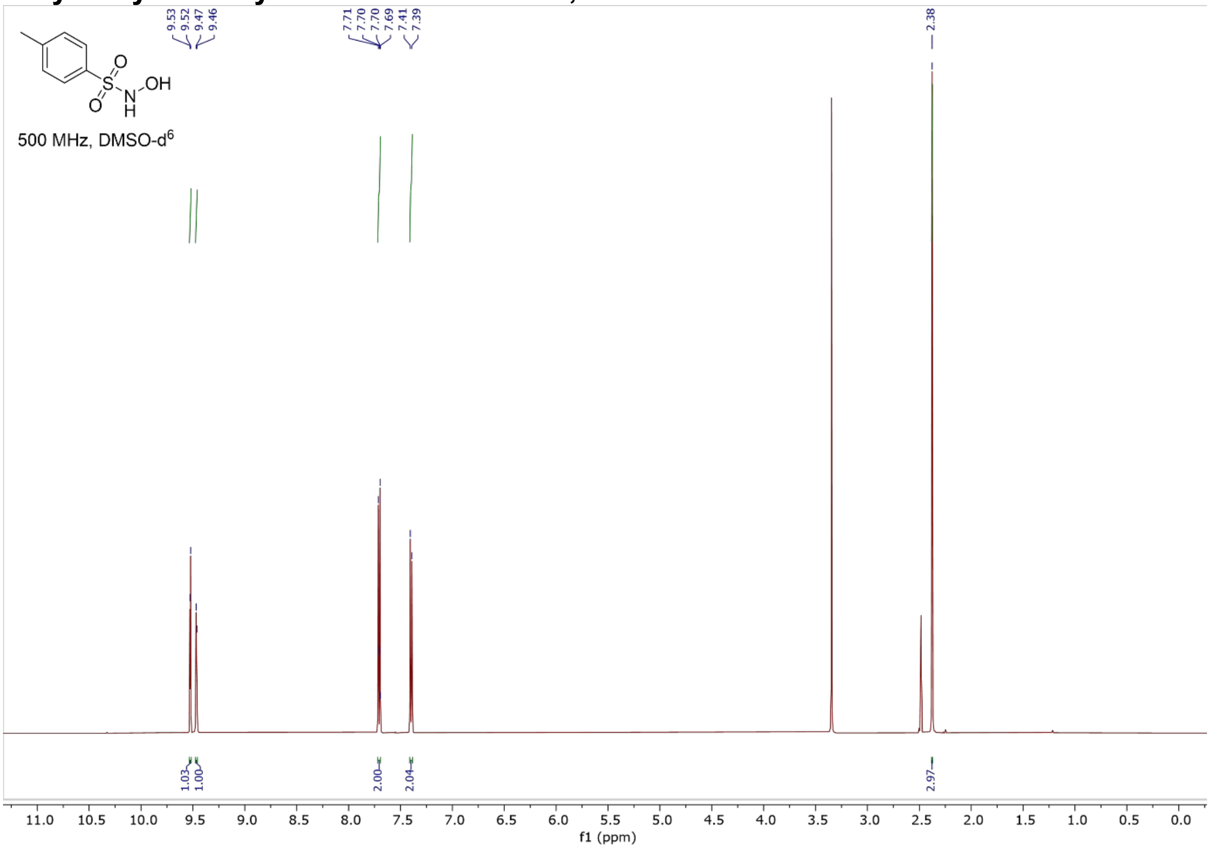

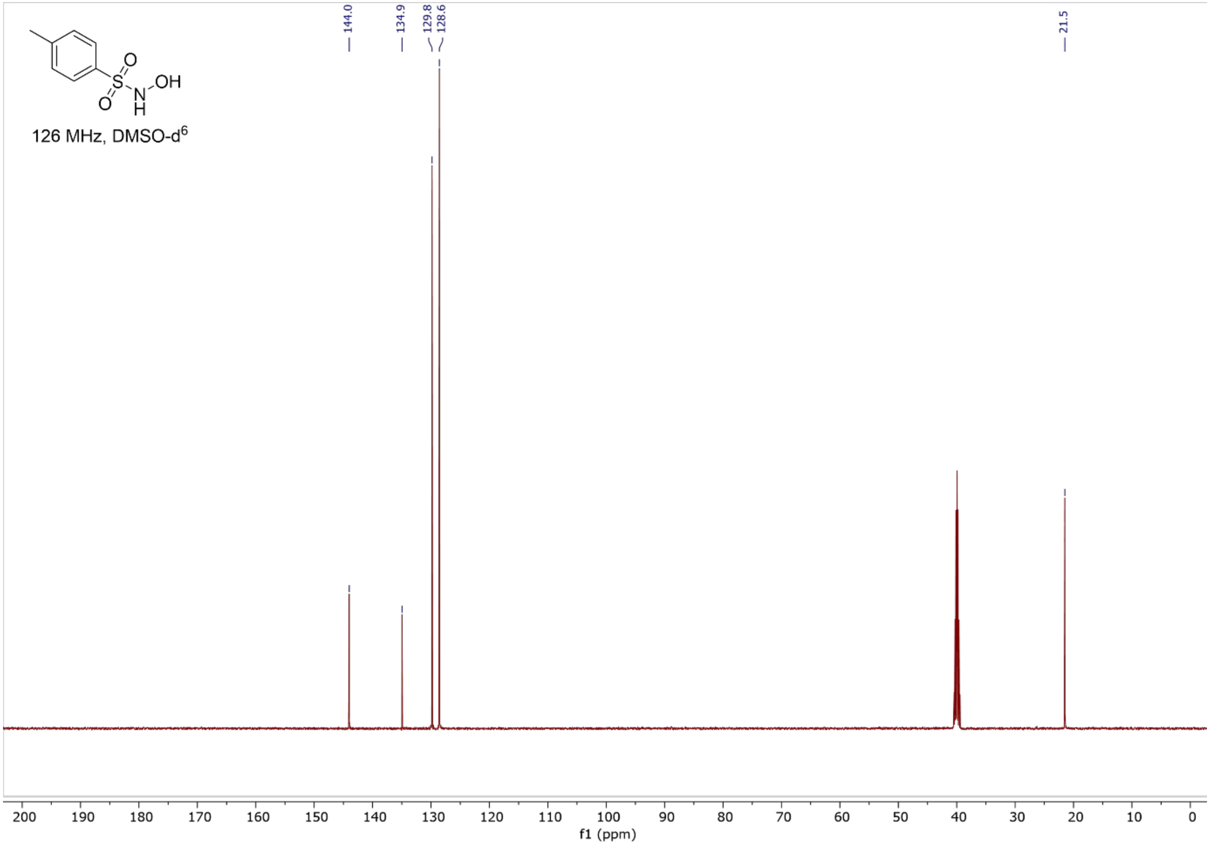

N-hydroxy-4-nitrobenzenesulfonamide, 10b

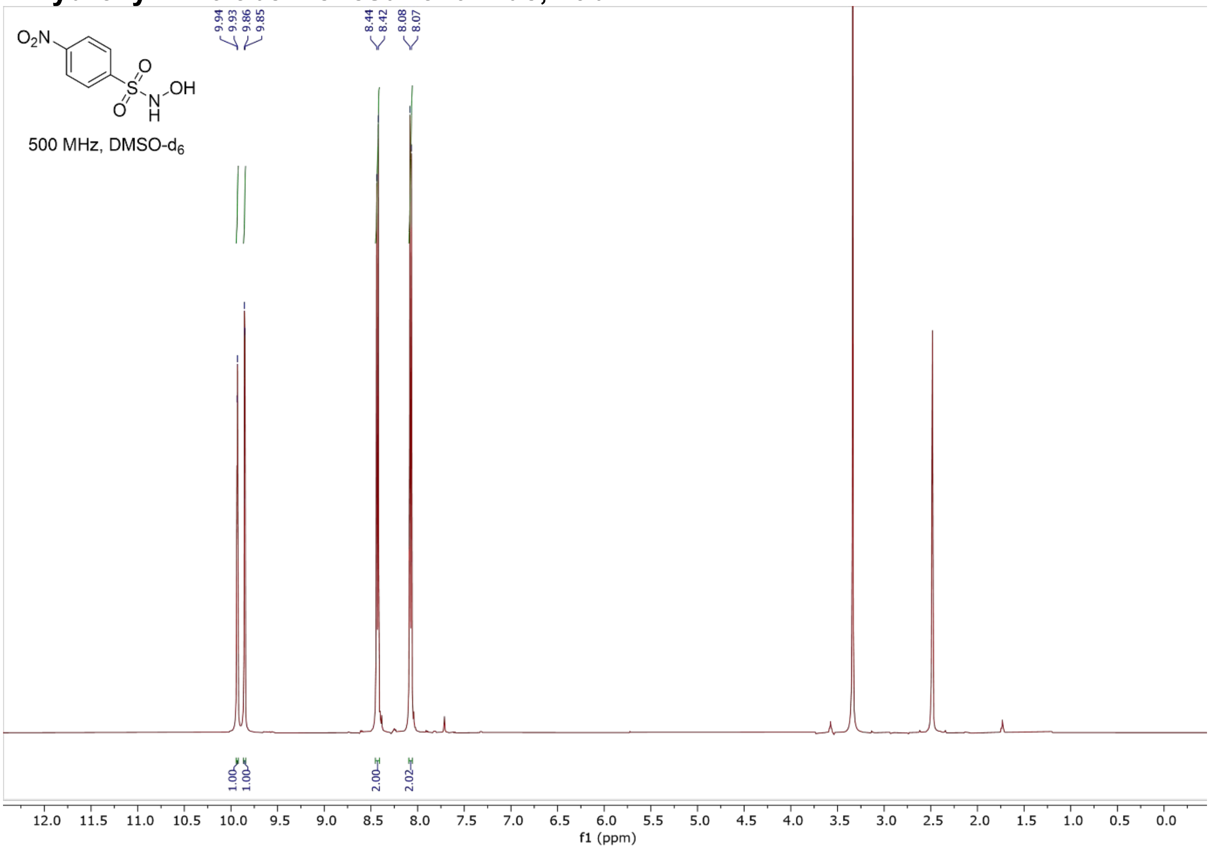

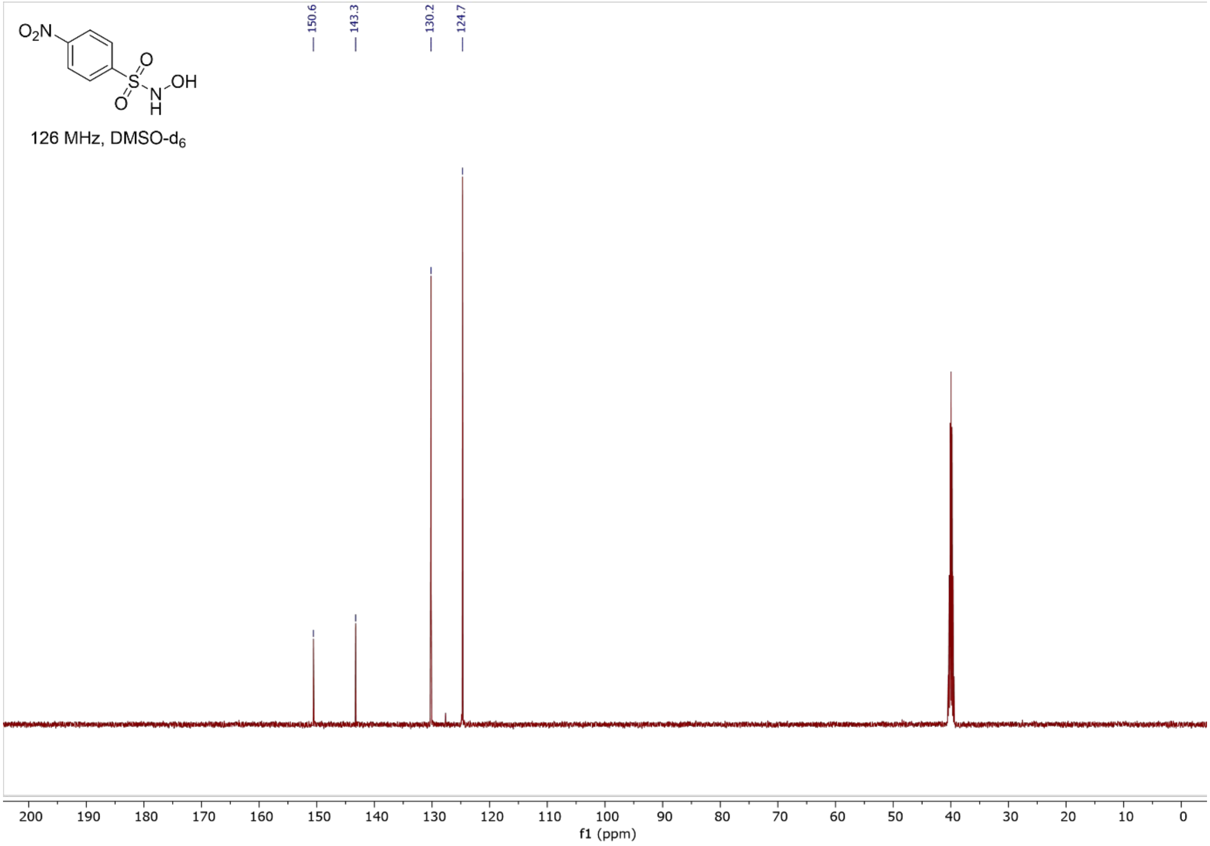

N-hydroxy-4-(trifluoromethyl)benzamide, 10c

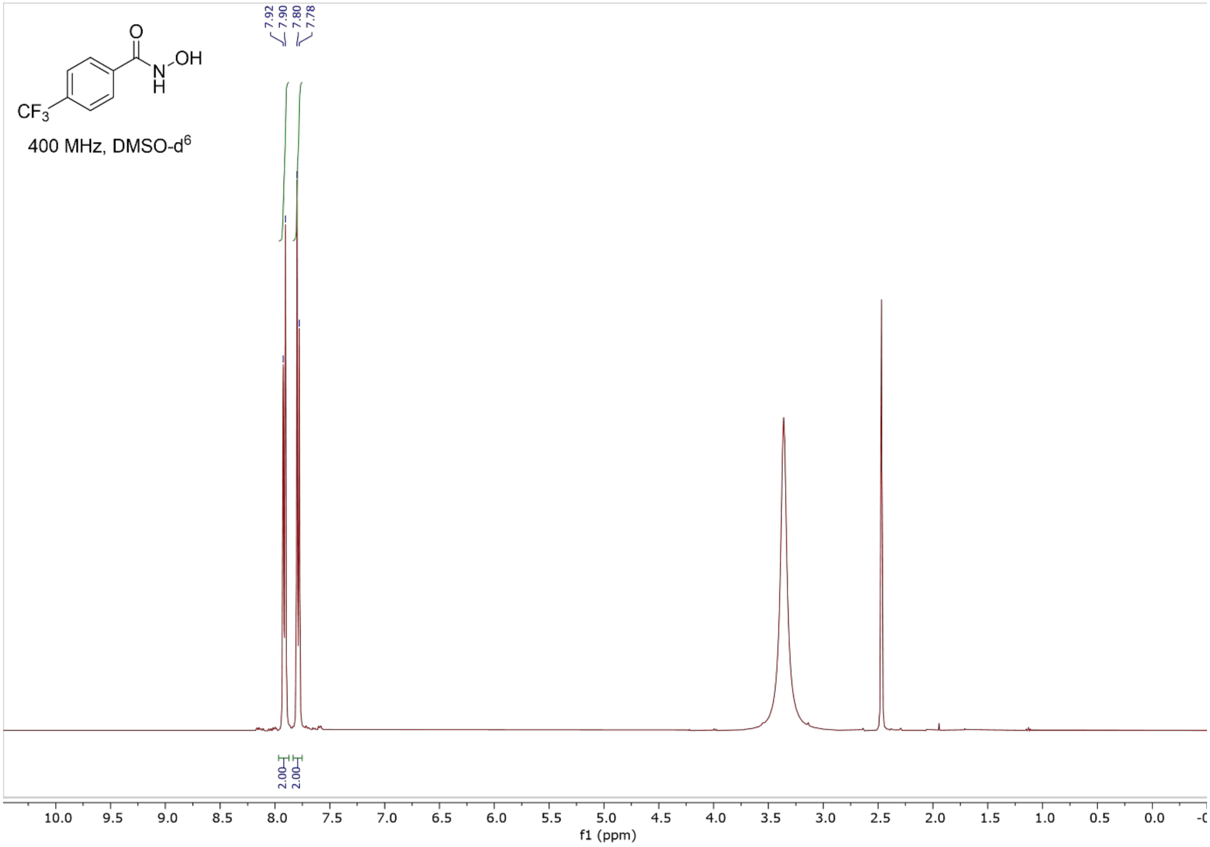

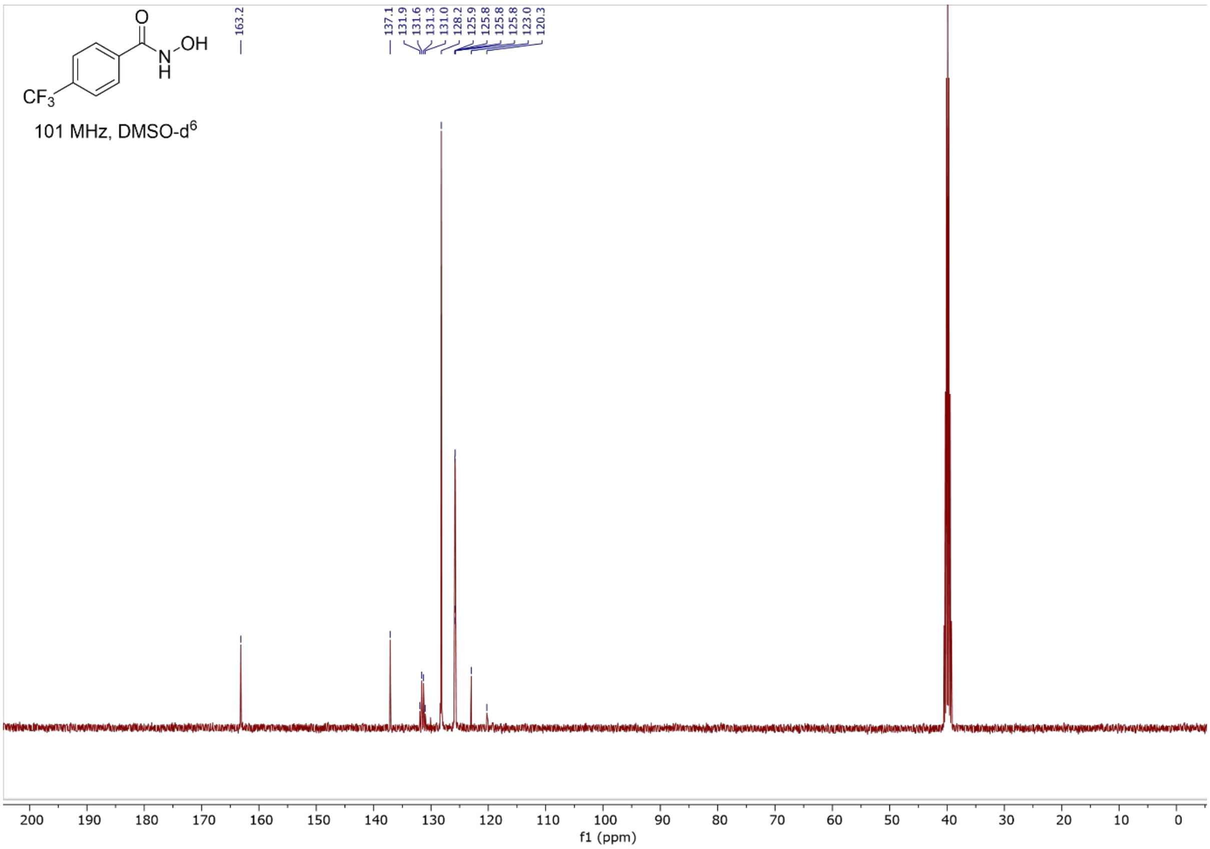
O
N
OH
H
CF3
101 MHz, DMSO-d6
163.2
137.1
131.9
131.6
131.3
131.0
128.2
125.9
125.8
125.8
125.8
123.0
120.3
200 190 180 170 160 150 140 130 120 110 100 90 80 70 60 50 40 30 20 10 0
f1 (ppm)

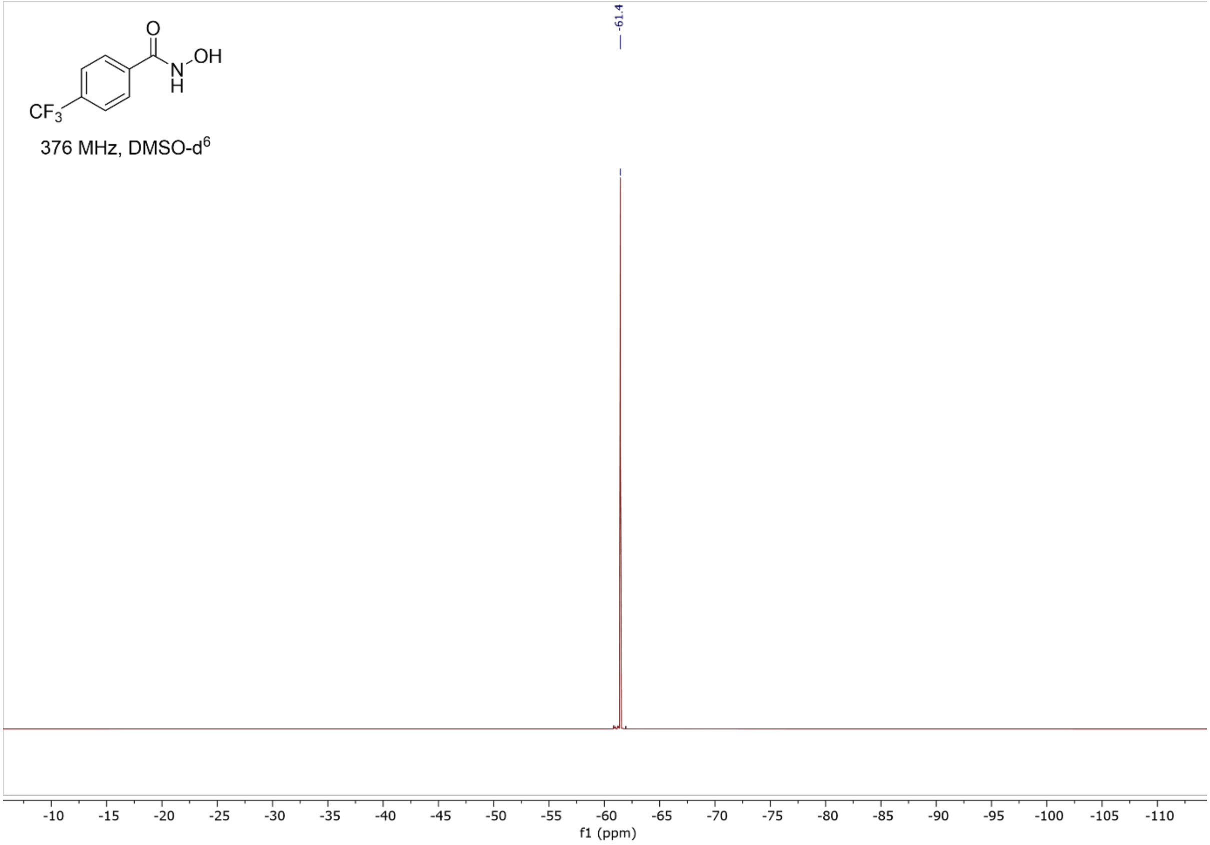
O
N
OH
H
CF3
376 MHz, DMSO-d6
-61.4
-10 -15 -20 -25 -30 -35 -40 -45 -50 -55 -60 -65 -70 -75 -80 -85 -90 -95 -100 -105 -110
f1 (ppm)

5-Butyl-3-(2,4-difluorophenyl)-2-tosyl-2,3-dihydroisoxazole, 11a

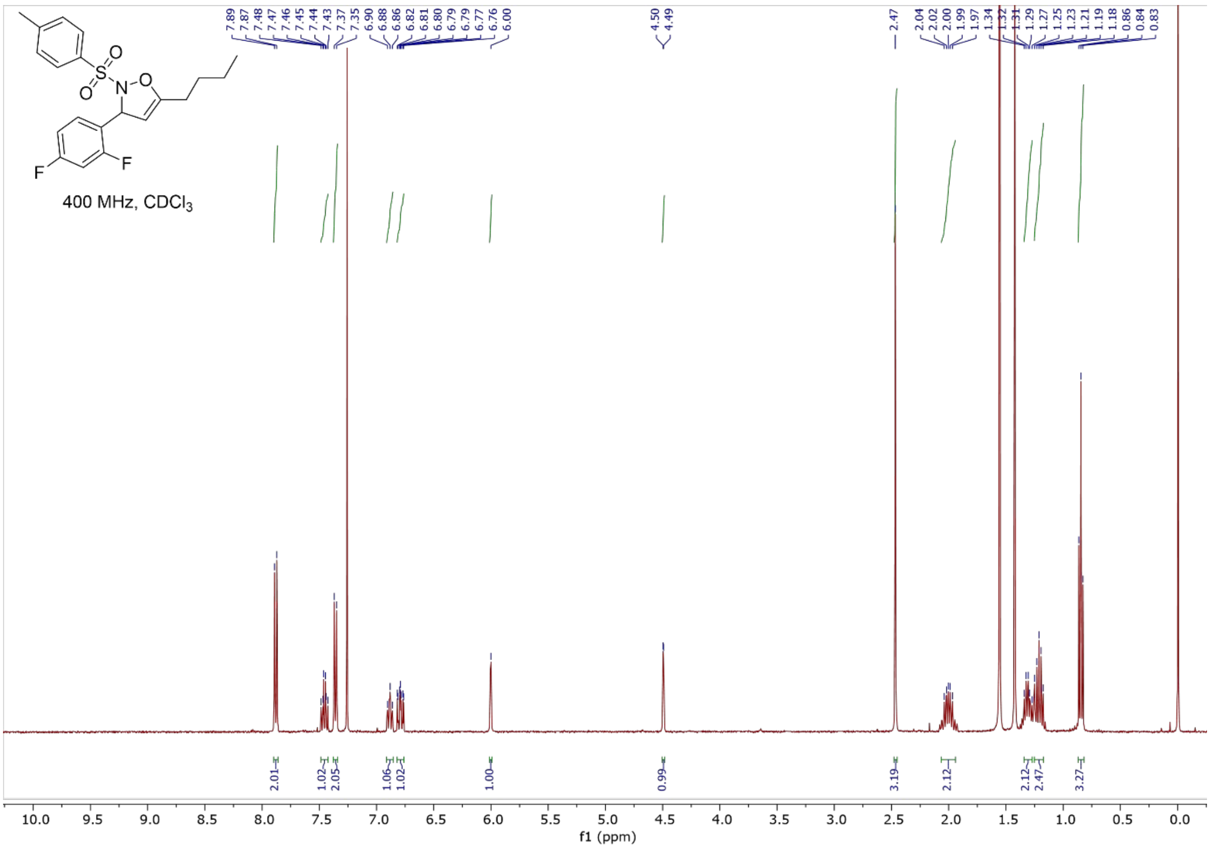

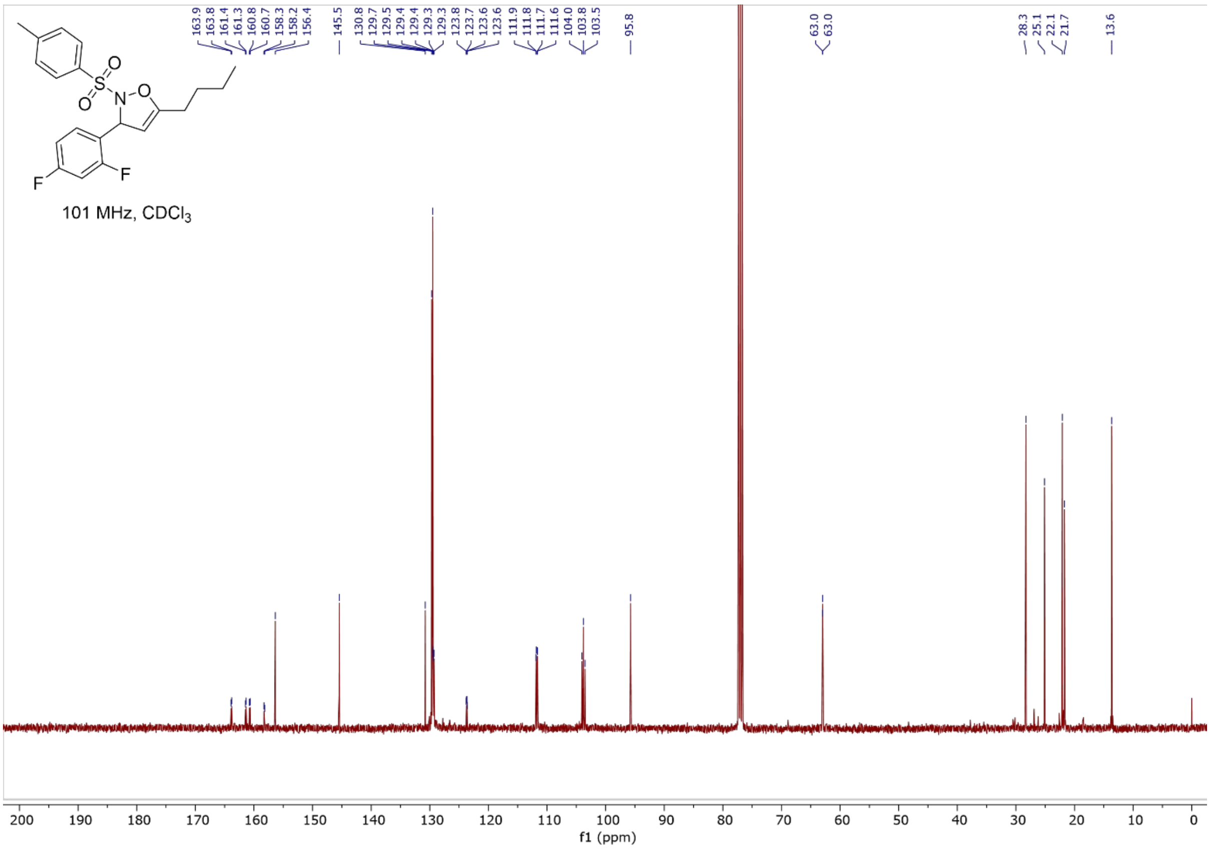

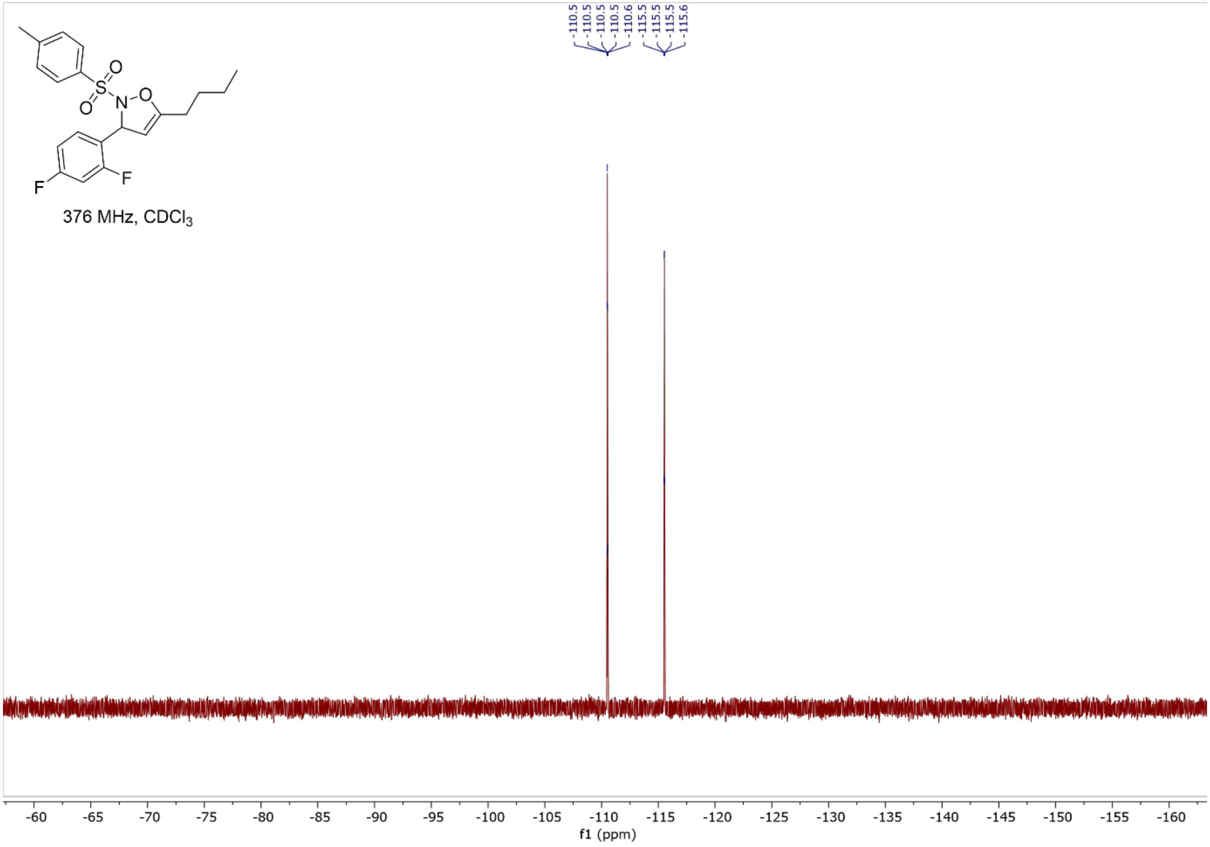

5-Butyl-3-(4-fluorophenyl)-2-tosyl-2,3-dihydroisoxazole, 11b

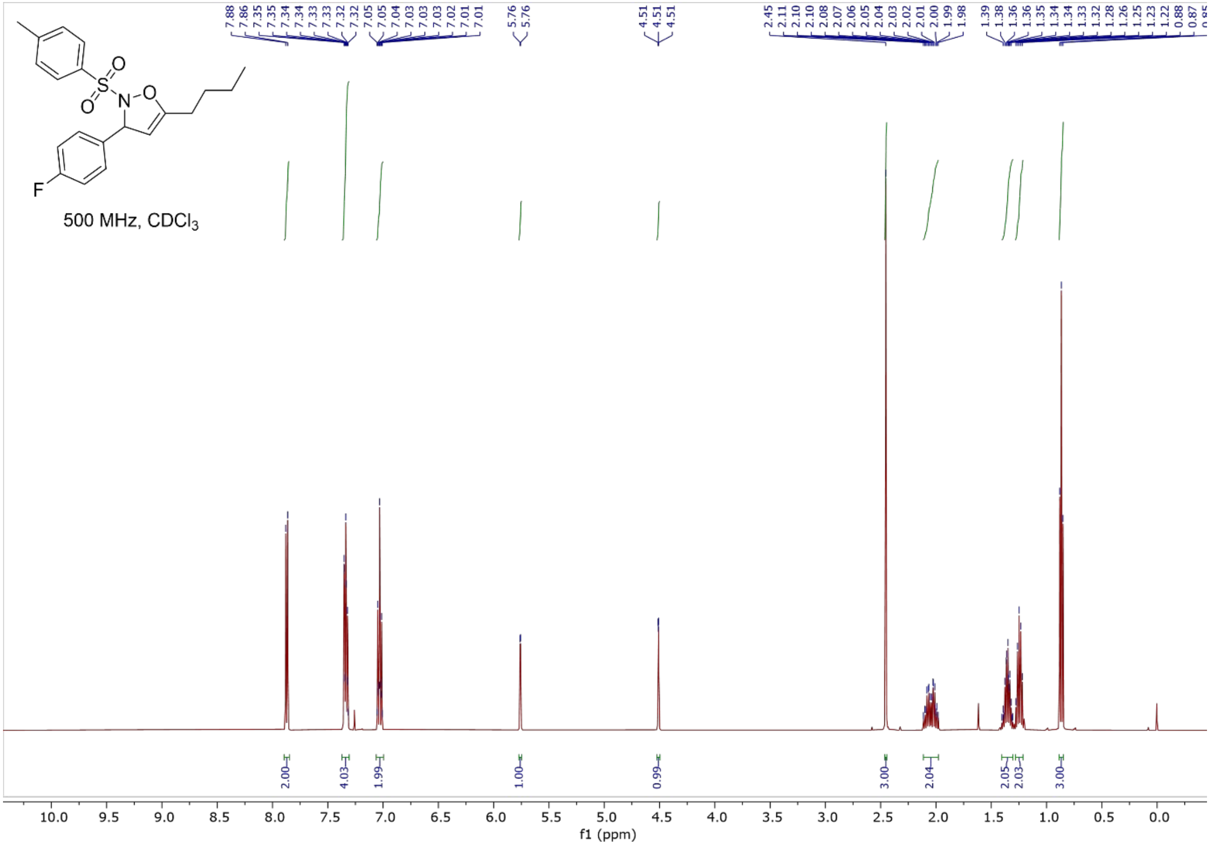

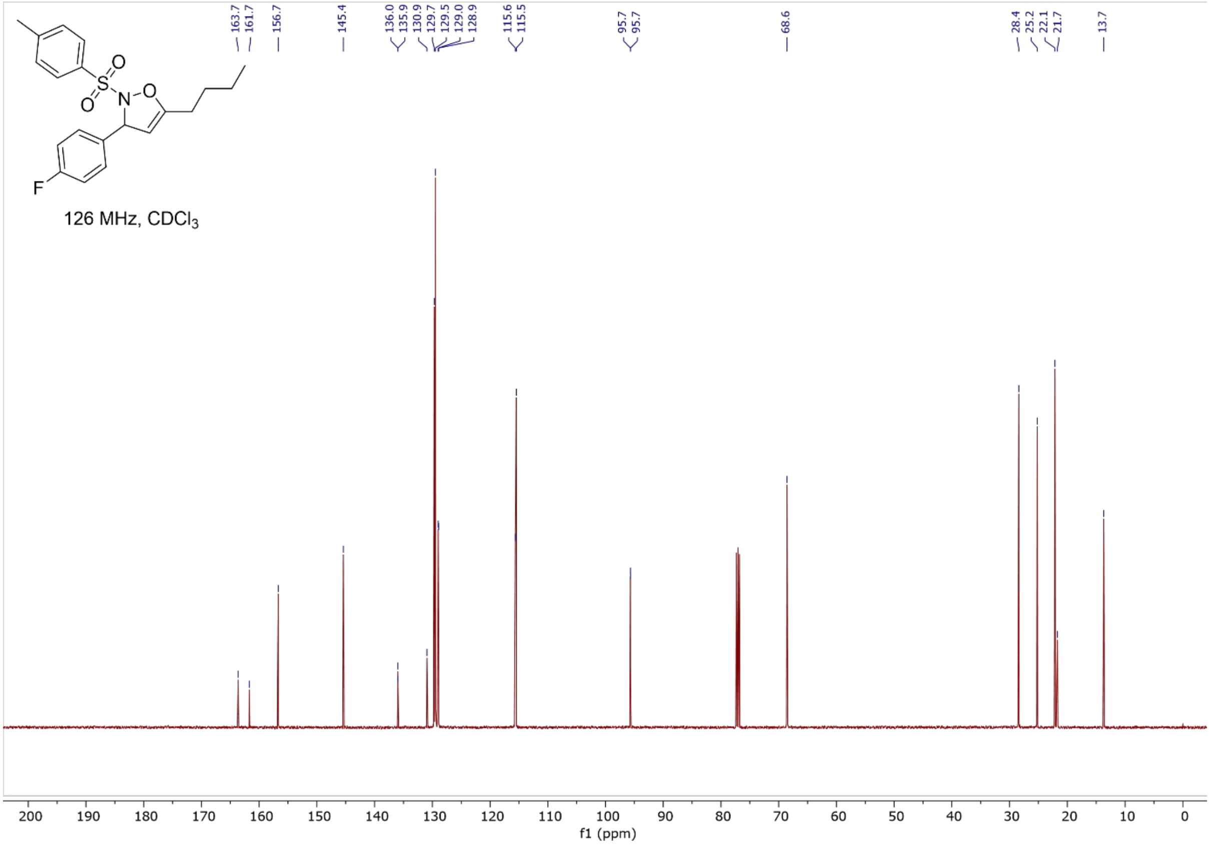

163.7
161.7
156.7
145.4
136.0
135.9
130.9
129.7
129.5
129.0
128.9
115.6
115.5
95.7
95.7
68.6
28.4
25.2
22.1
21.7
13.7
126 MHz, CDCl3
f1 (ppm)

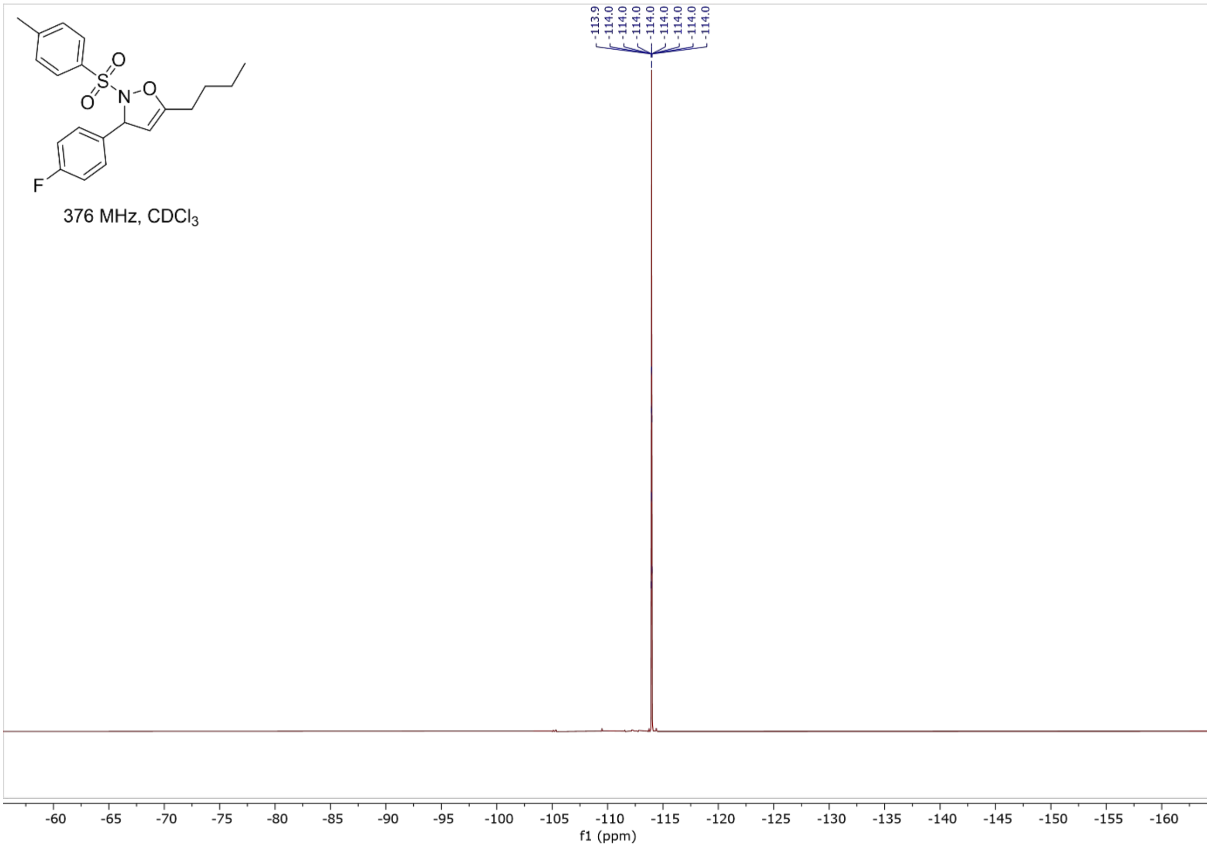

-113.9
-114.0
-114.0
-114.0
-114.0
-114.0
-114.0
-114.0
-114.0
376 MHz, CDCl3
f1 (ppm)

1-(4-Bromophenyl)hept-2-yn-1-ol, 11c

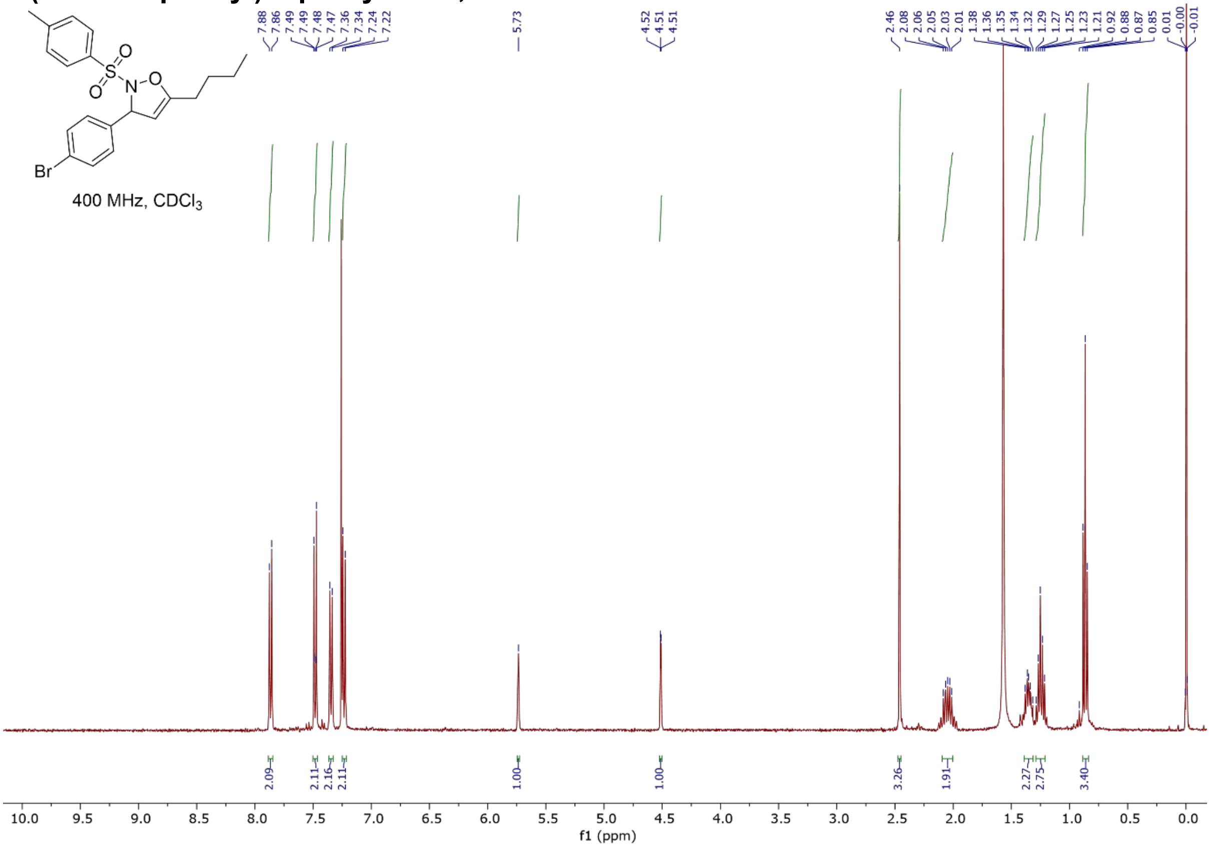

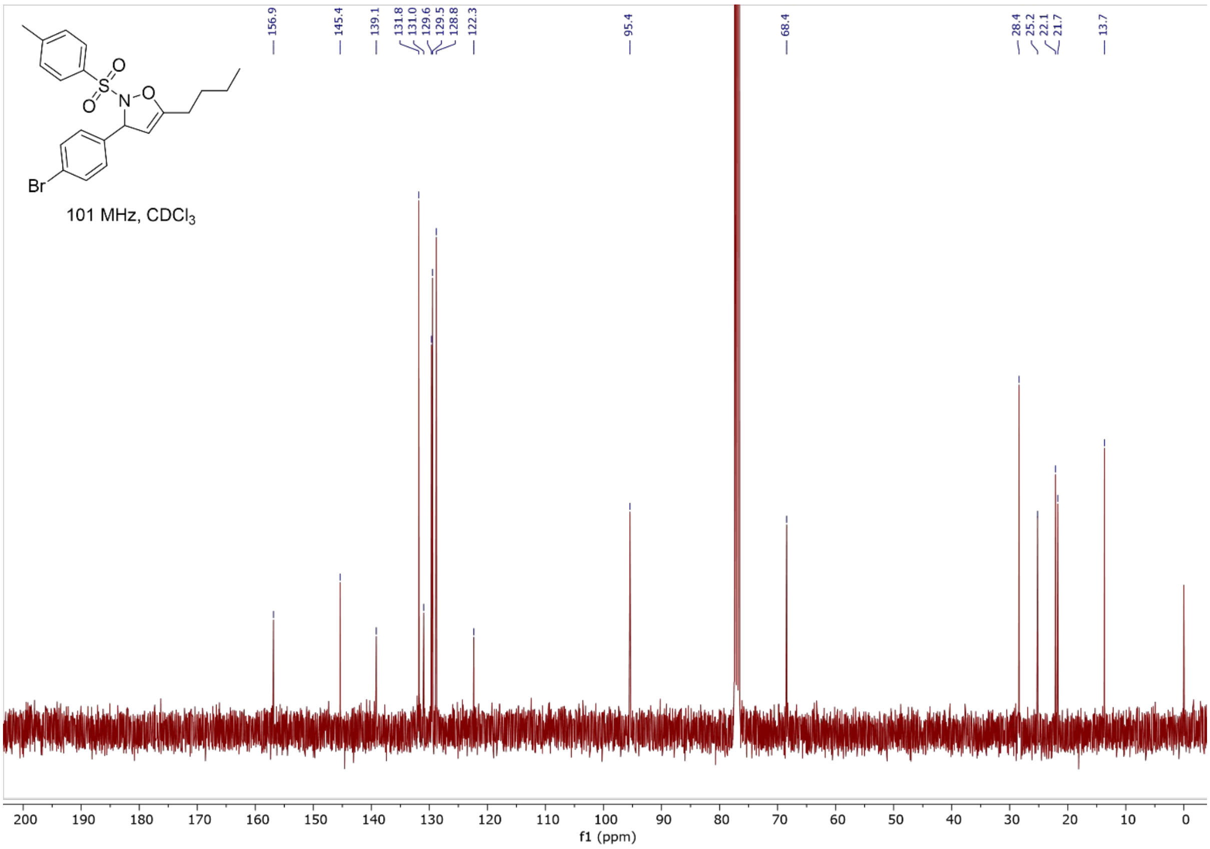

5-Butyl-3-(2-chlorophenyl)-2-tosyl-2,3-dihydroisoxazole, 11d

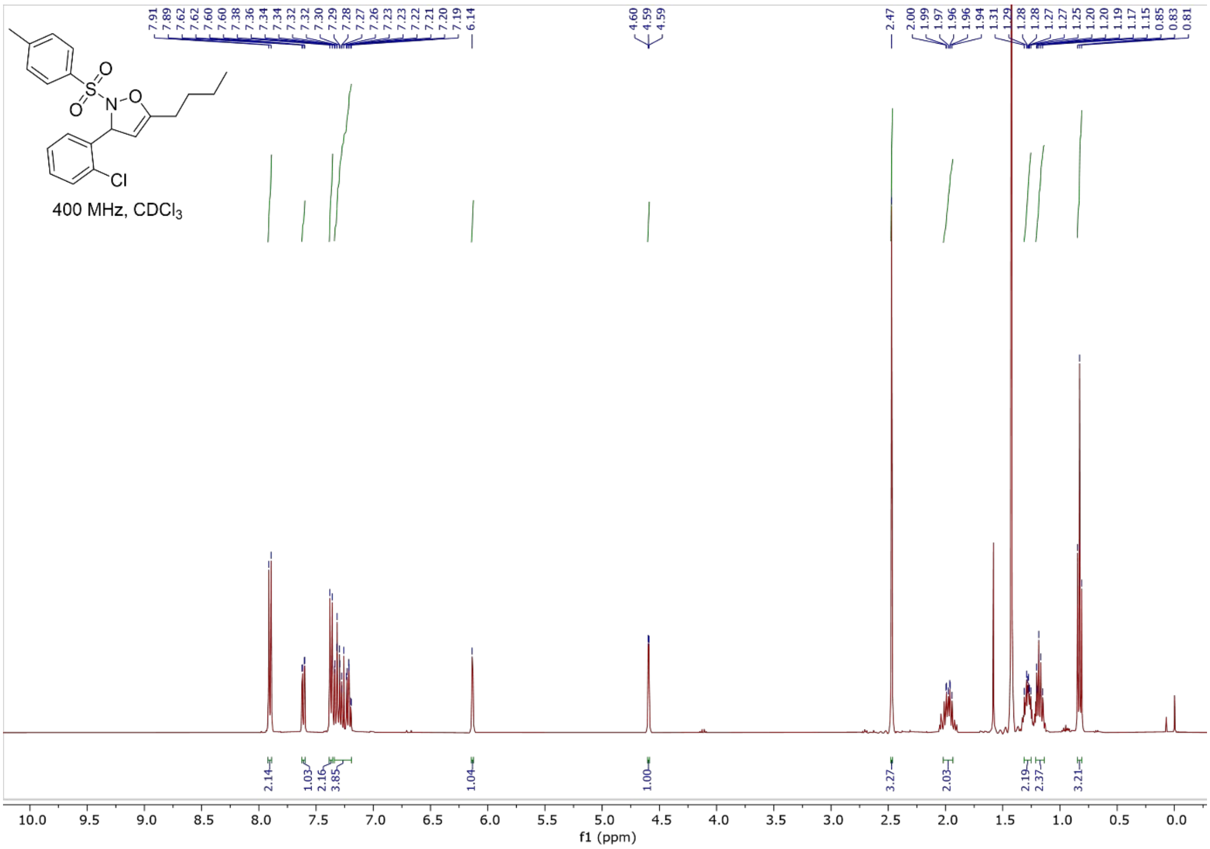

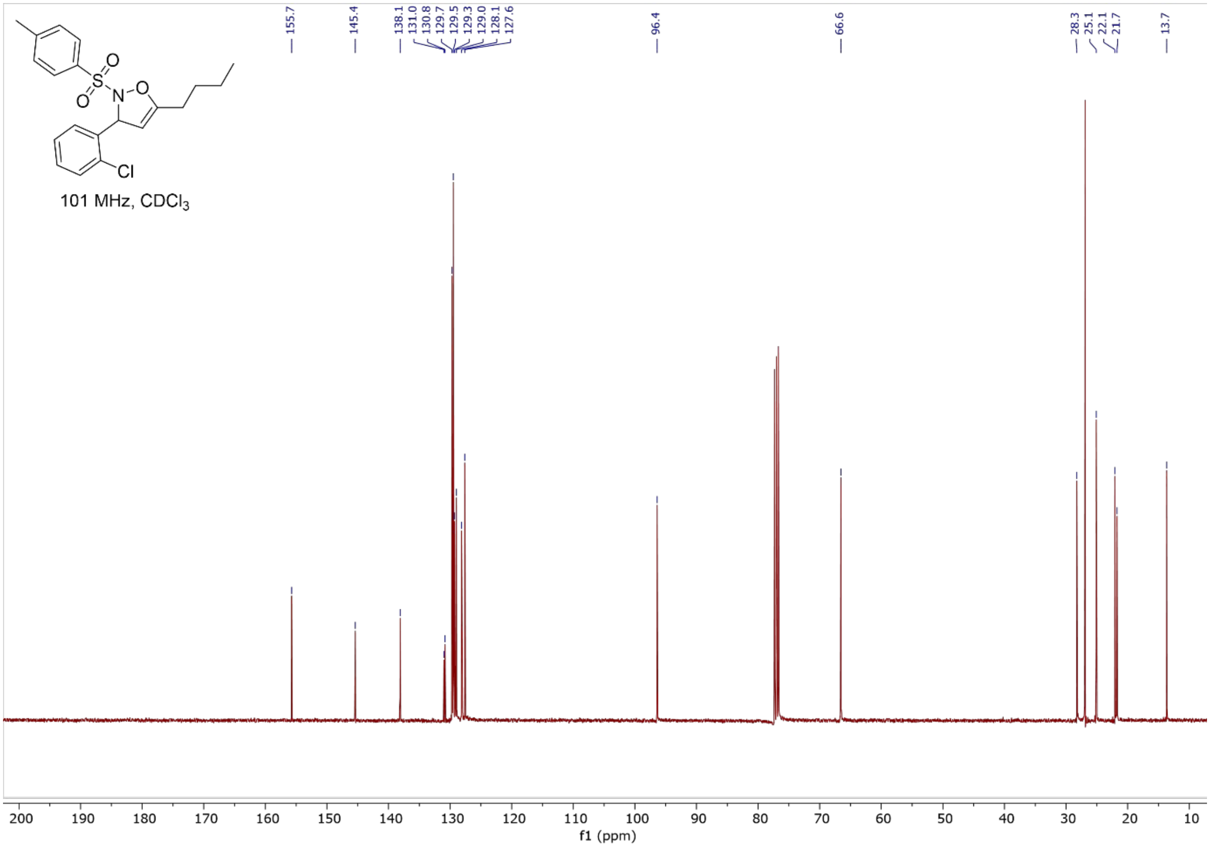

5-Butyl-3-(3-chloro-4-fluorophenyl)-2-tosyl-2,3-dihydroisoxazole, 11e

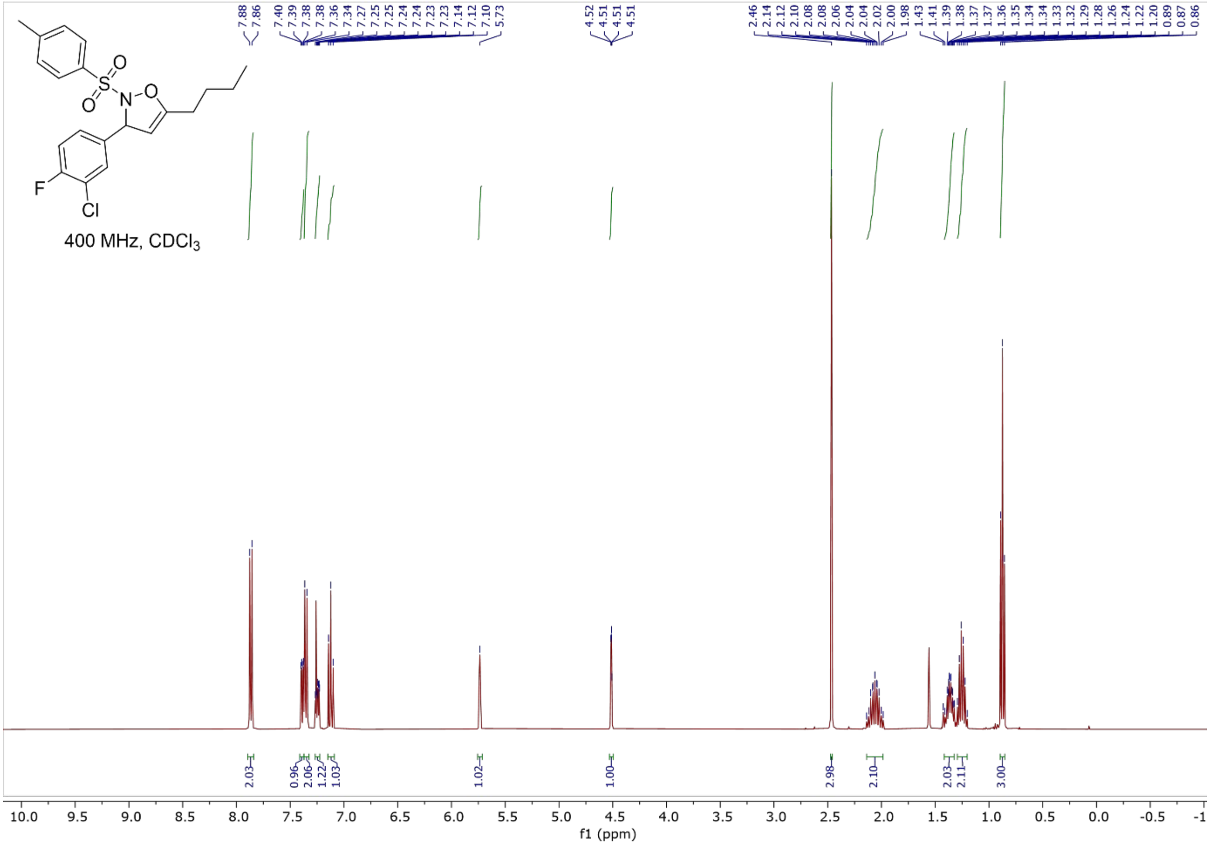

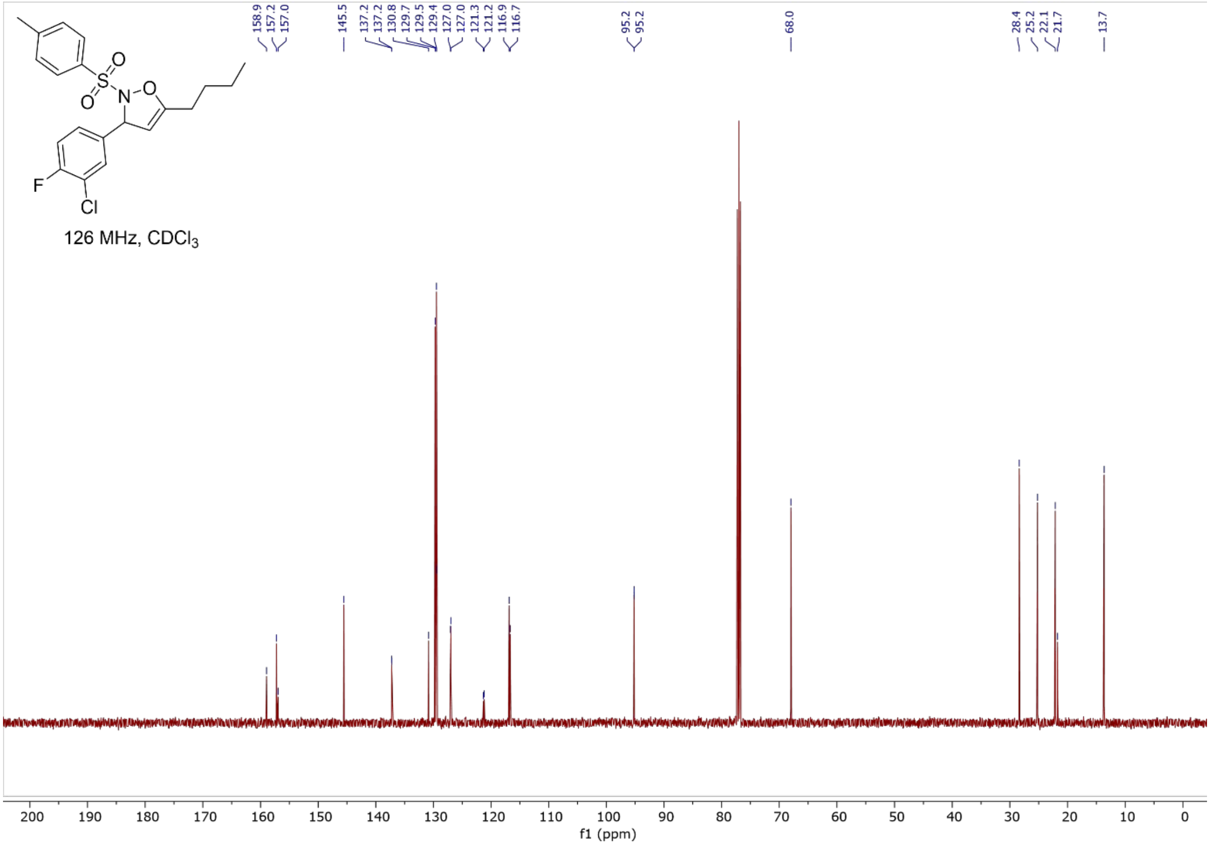

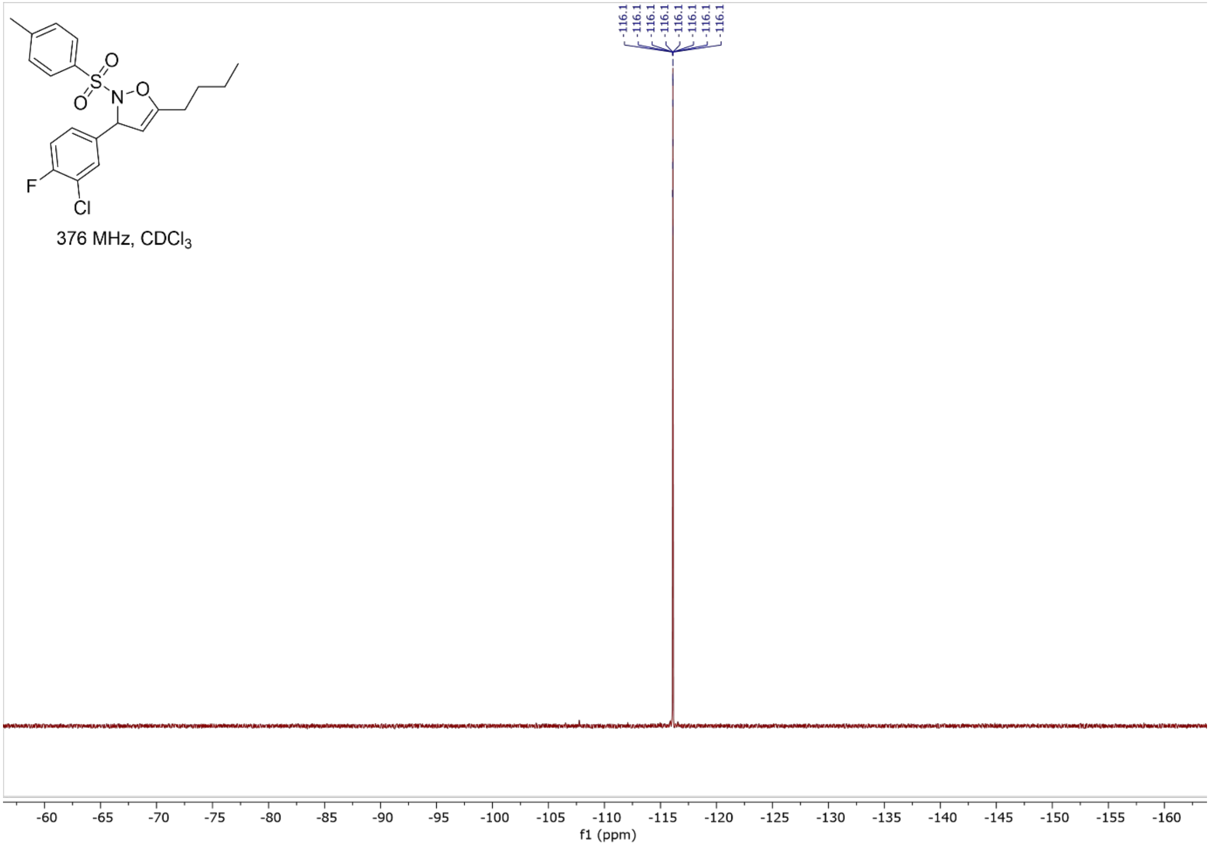

5-Butyl-2-tosyl-3-(4-(trifluoromethyl)phenyl)-2,3-dihydroisoxazole, 11f

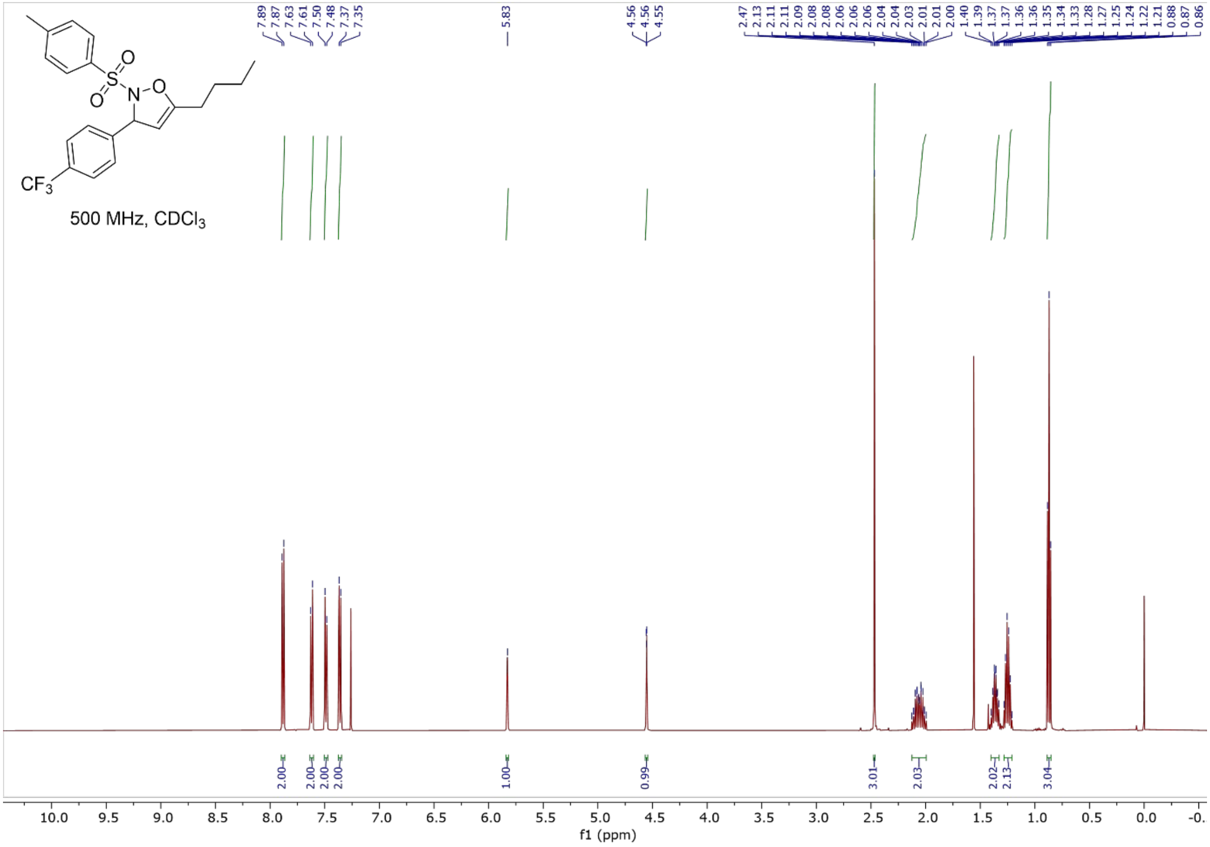

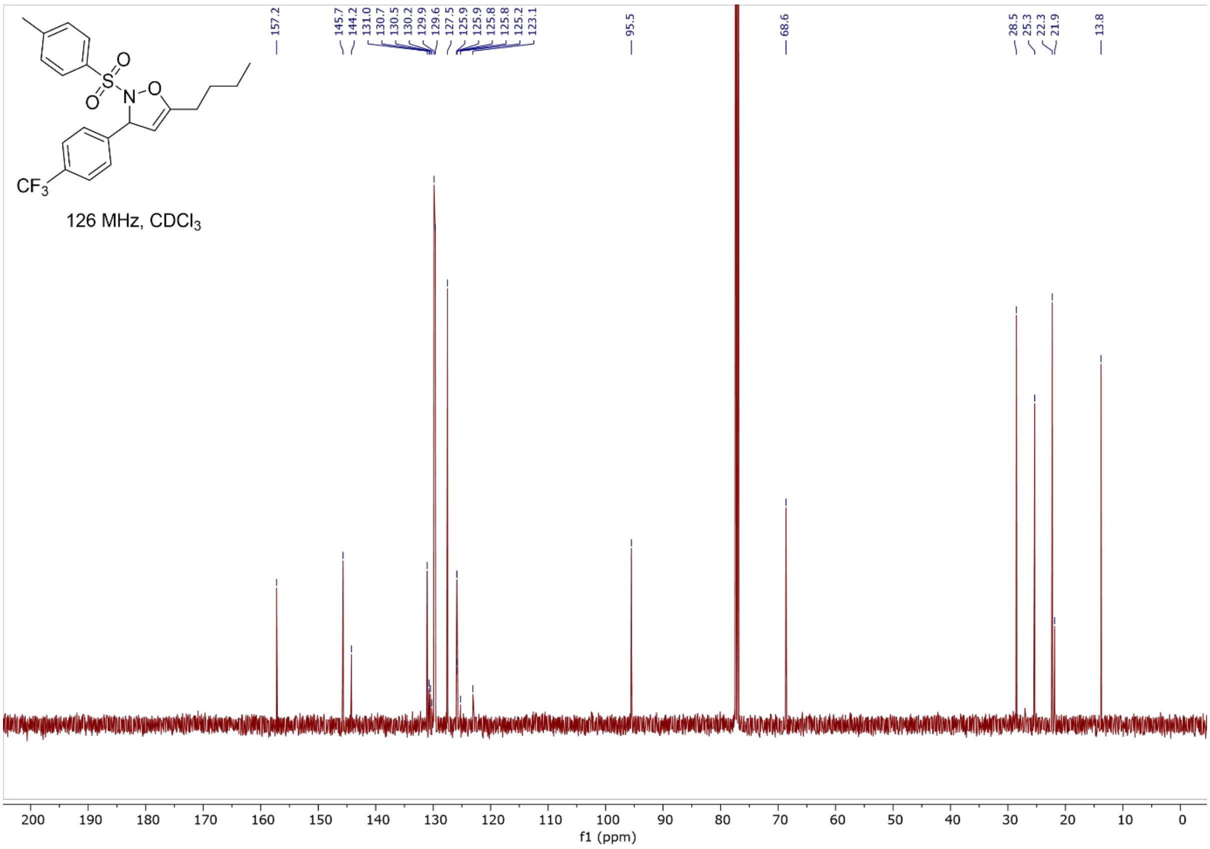
126 MHz, CDCl3
157.2
145.7
144.2
131.0
130.7
130.5
130.2
129.9
129.6
127.5
125.9
125.9
125.8
125.8
125.2
123.1
95.5
68.6
28.5
25.3
22.3
21.9
13.8
f1 (ppm)

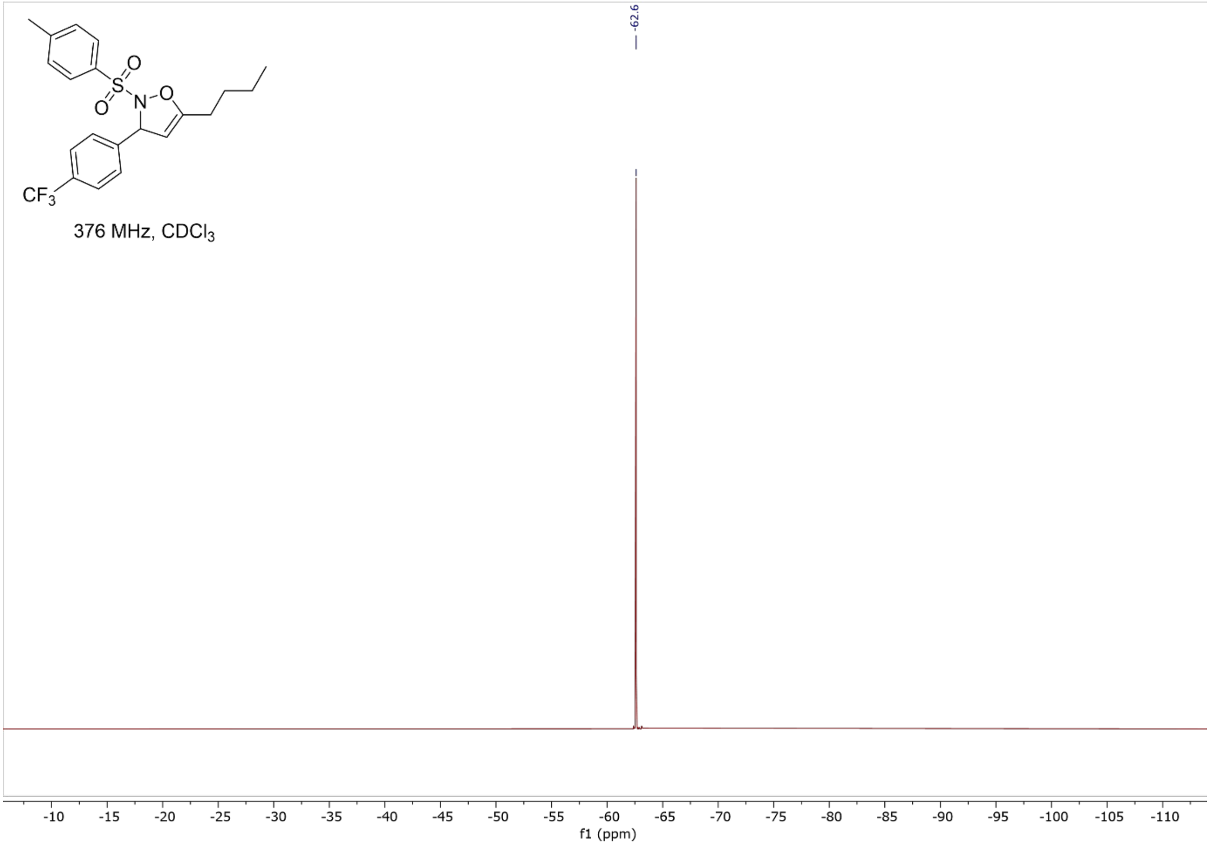
376 MHz, CDCl3
-62.6
f1 (ppm)

5-Butyl-3-phenyl-2-tosyl-2,3-dihydroisoxazole, 11g

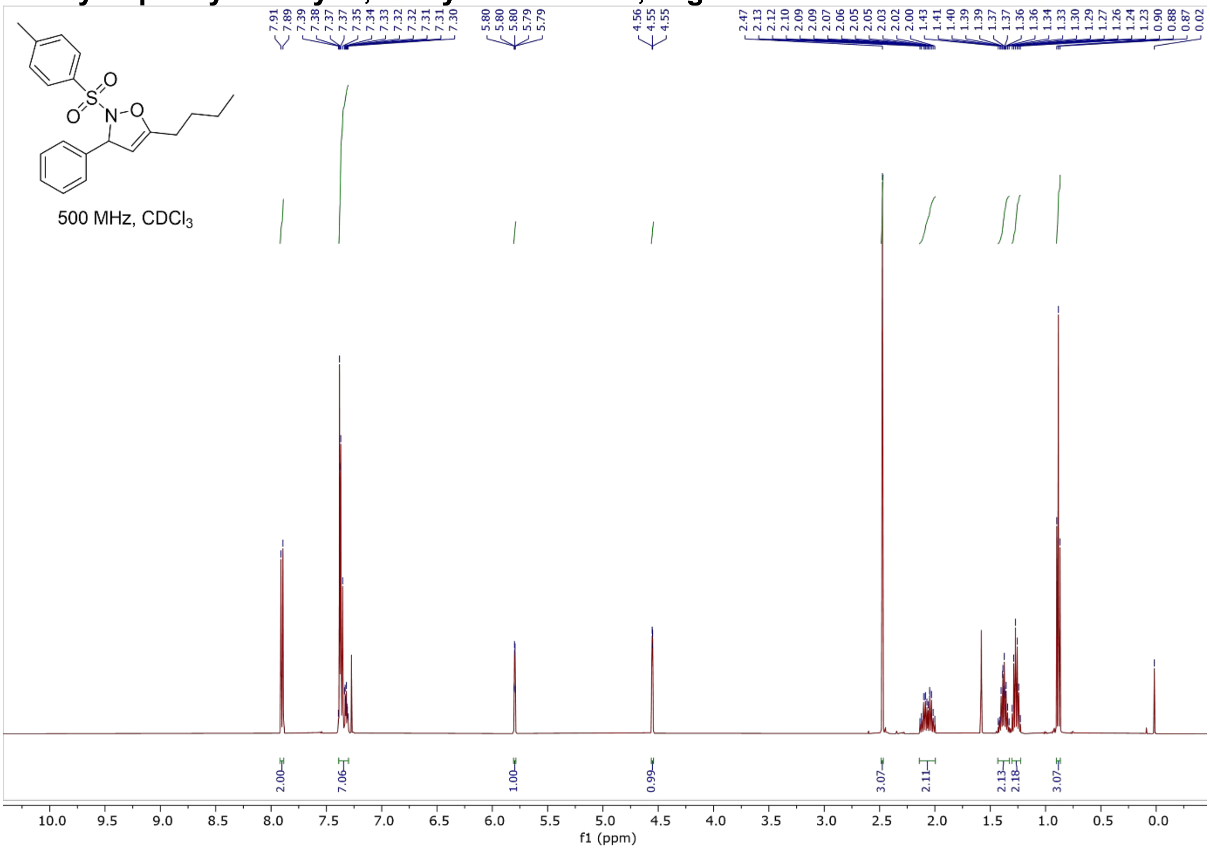

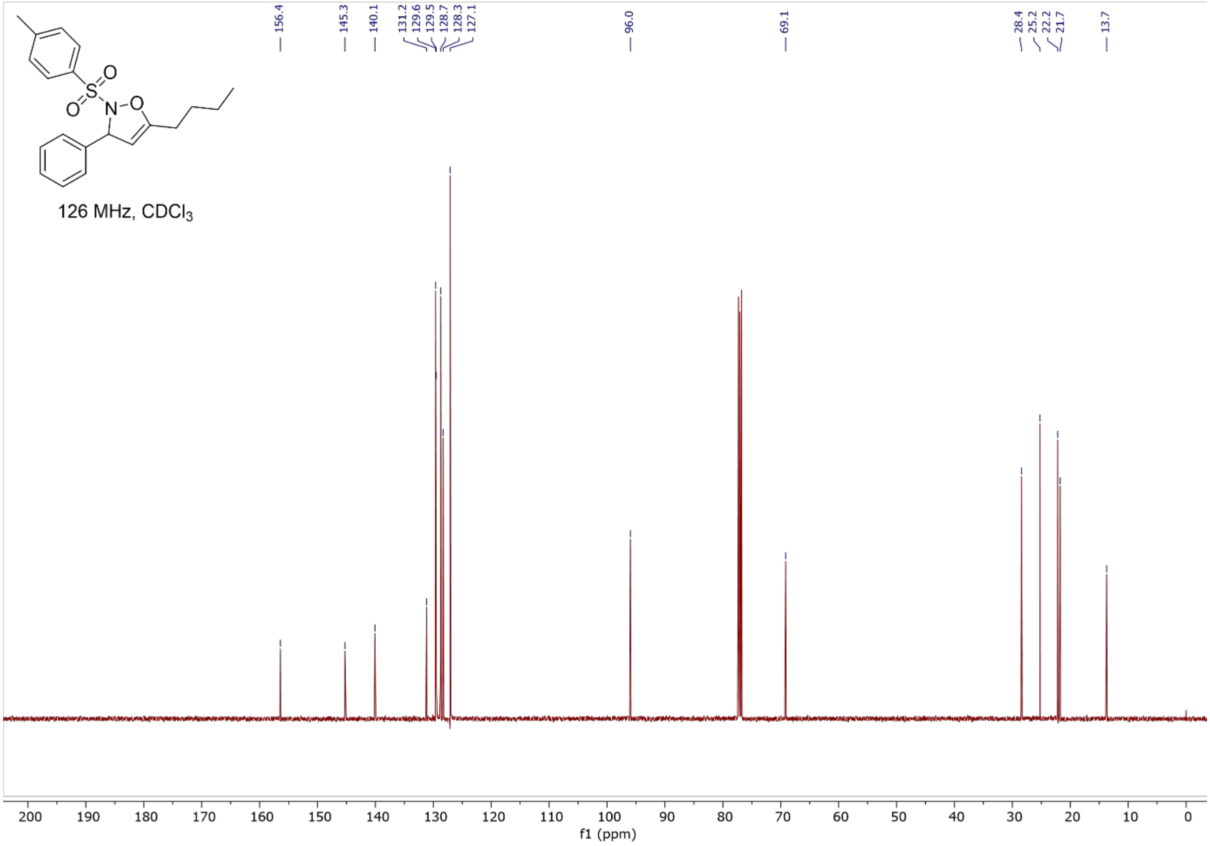

5-Butyl-3-(3,4-dimethylphenyl)-2-tosyl-2,3-dihydroisoxazole, 11h

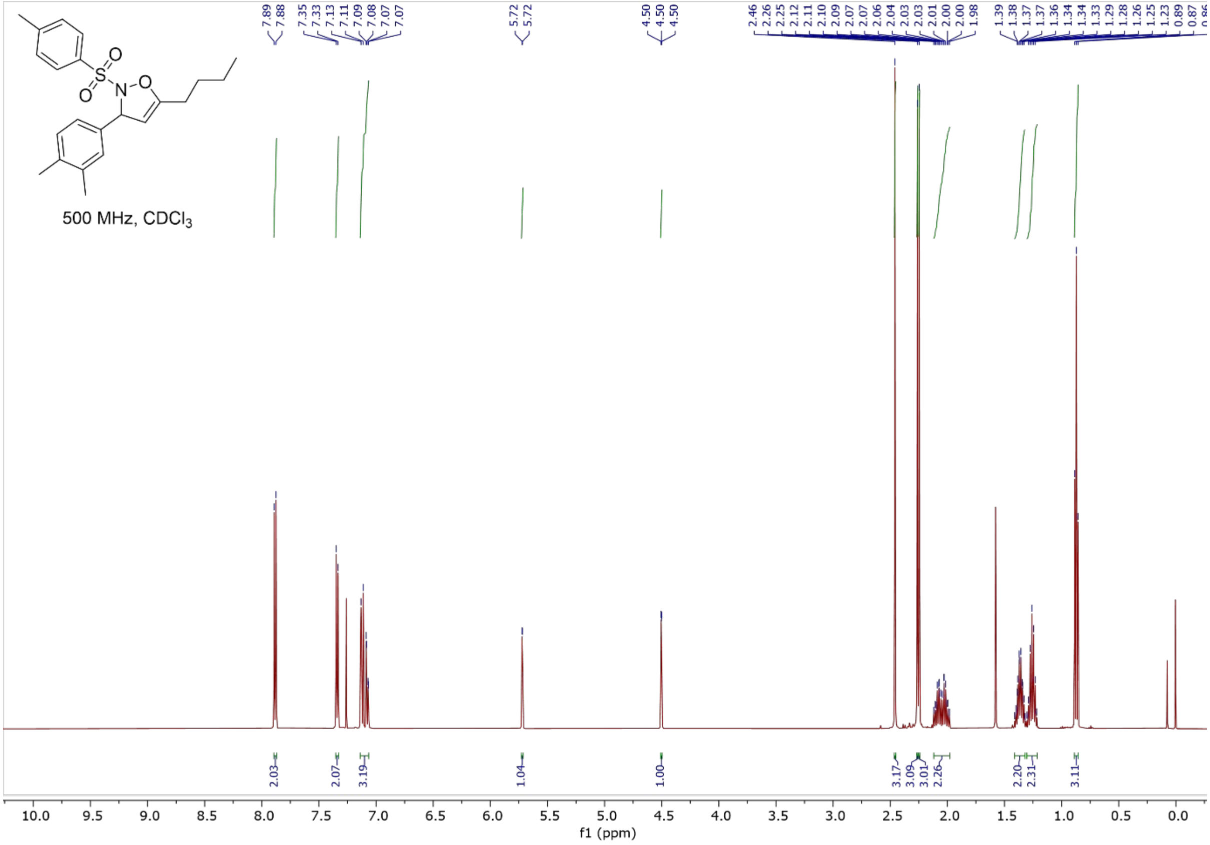

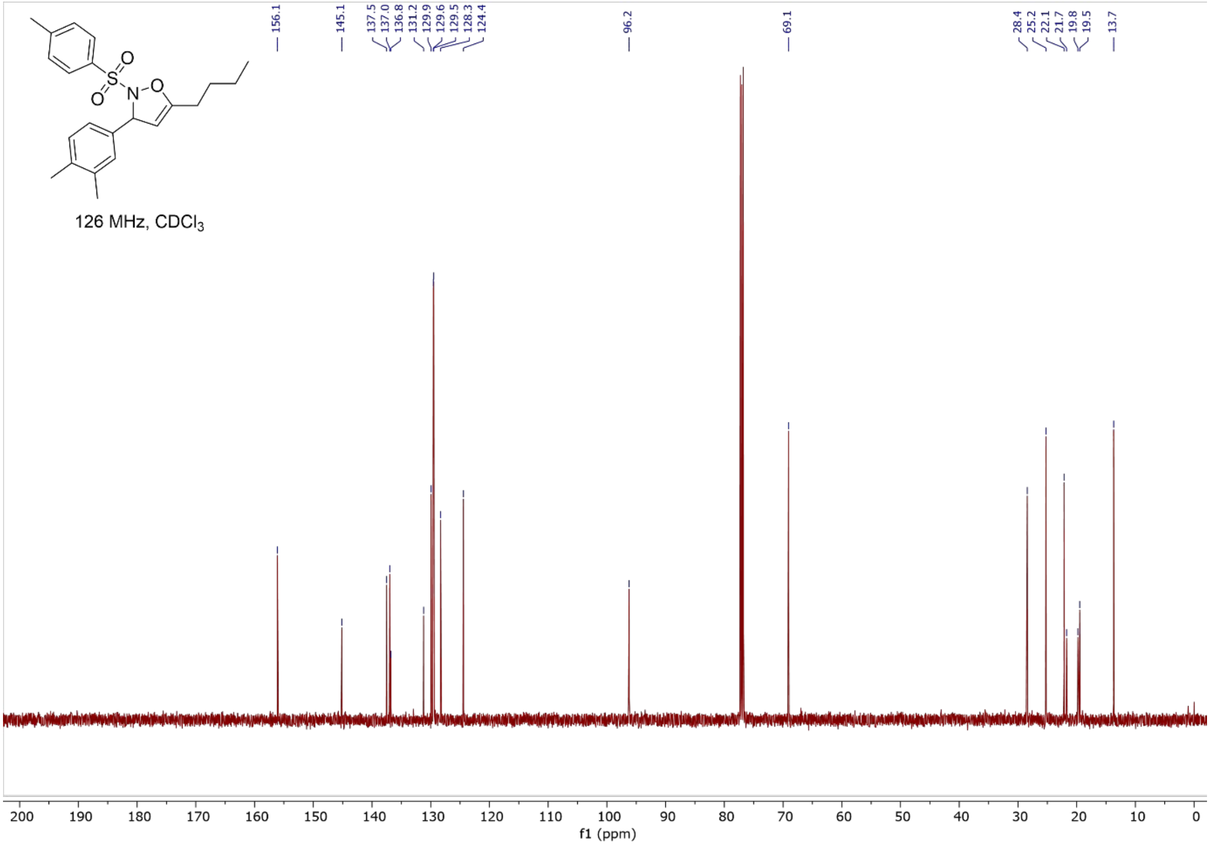

5-Butyl-3-(3,4-dimethoxyphenyl)-2-tosyl-2,3-dihydroisoxazole, 11i

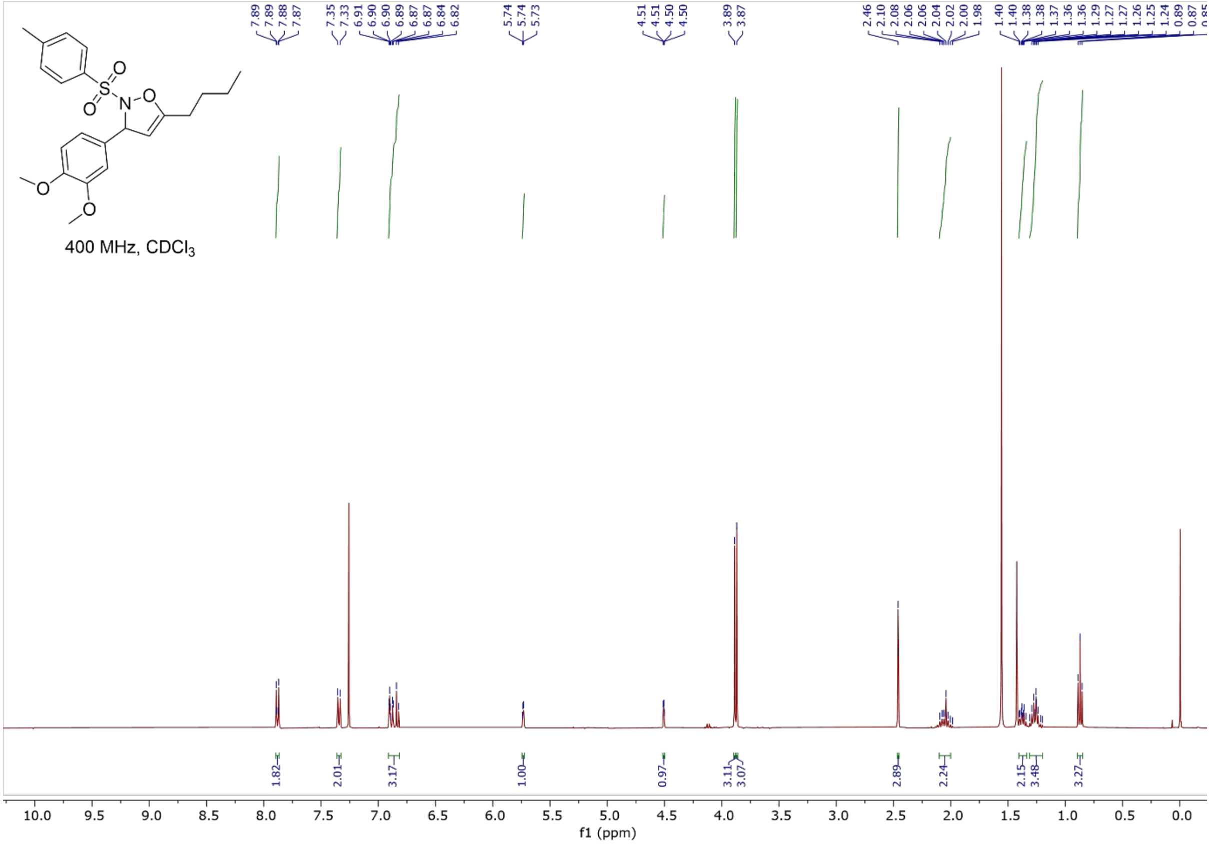

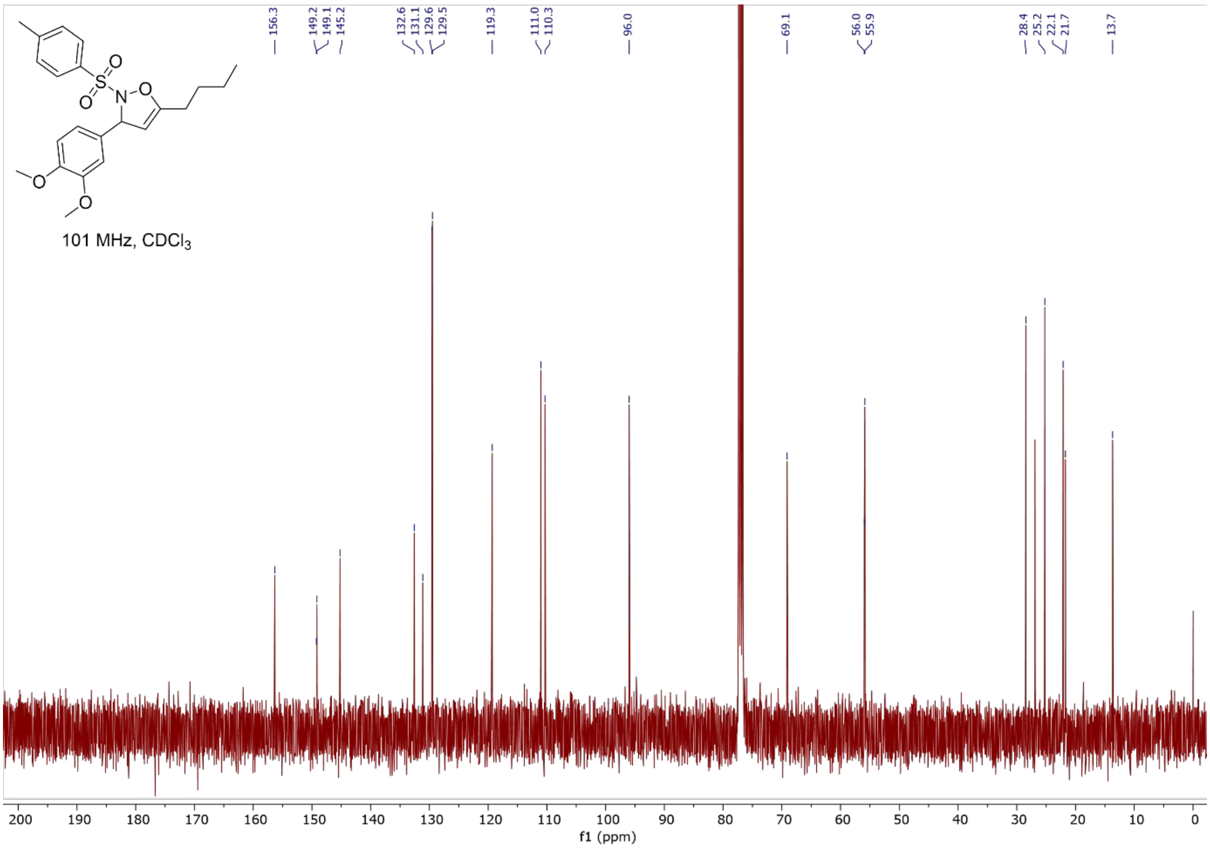

5-Butyl-3-(naphthalen-2-yl)-2-tosyl-2,3-dihydroisoxazole, 11k

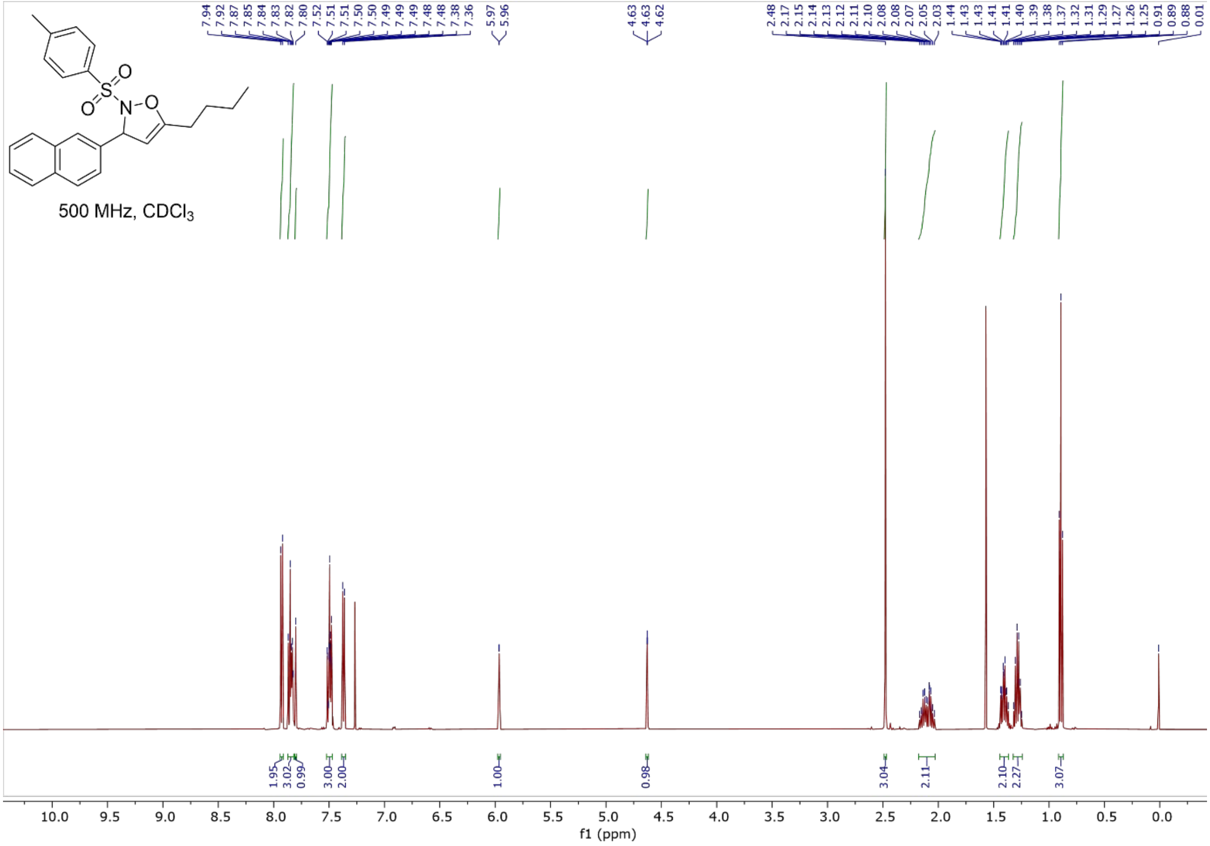

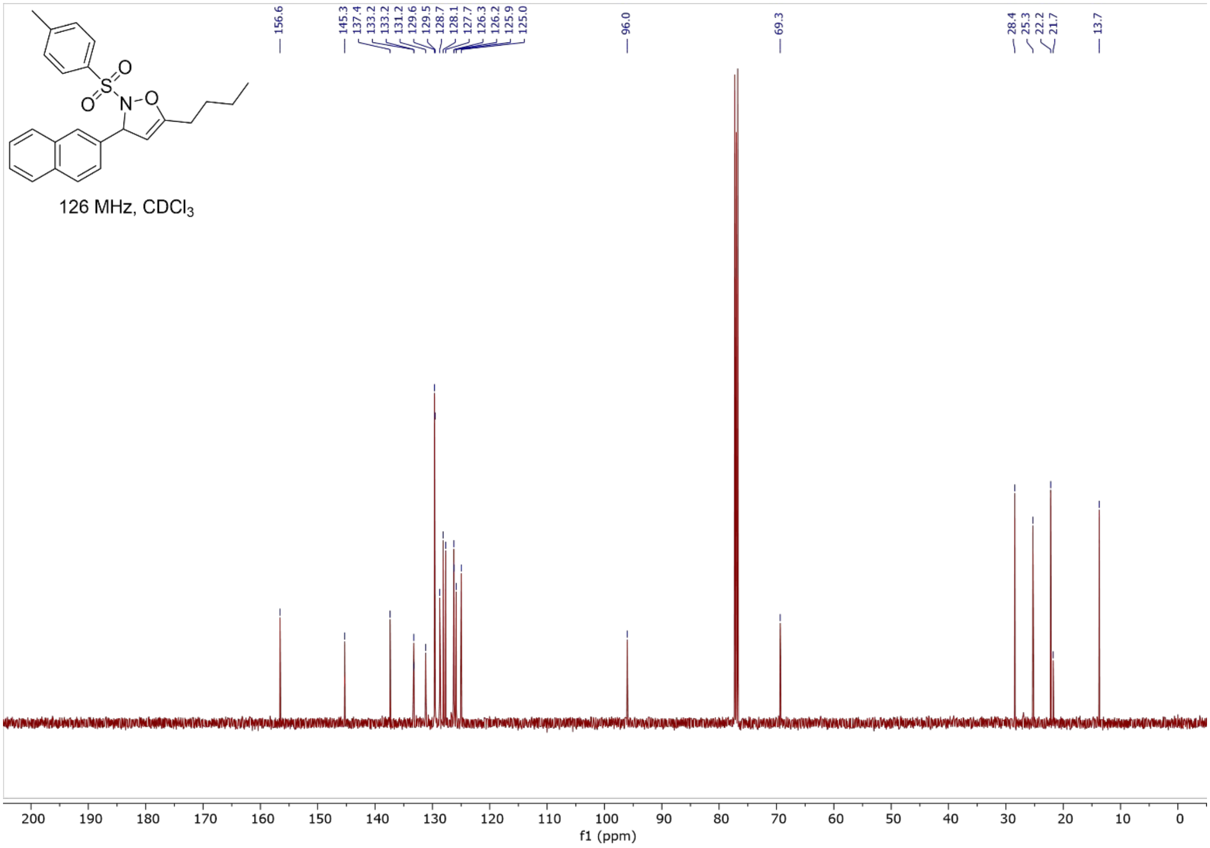

5-Cyclopropyl-3-(2,4-difluorophenyl)-2-tosyl-2,3-dihydroisoxazole, 11l

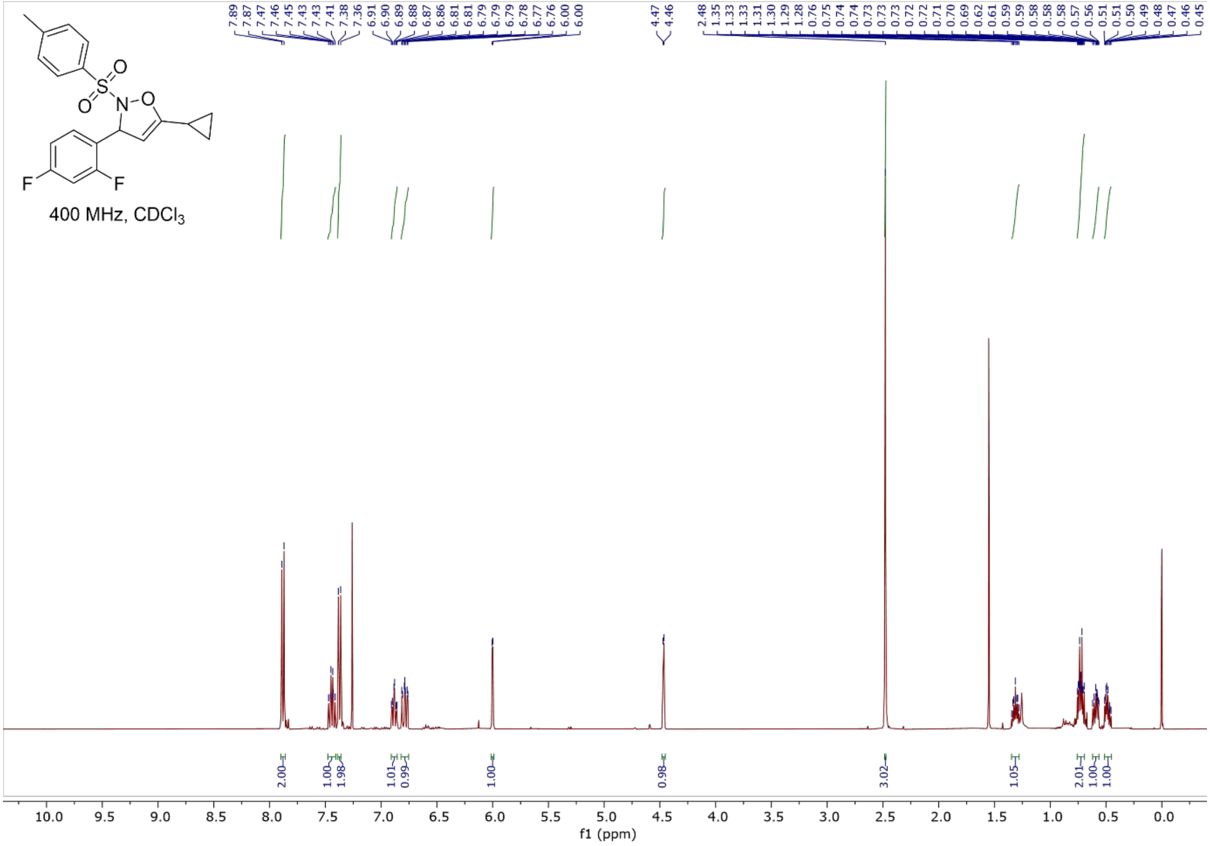

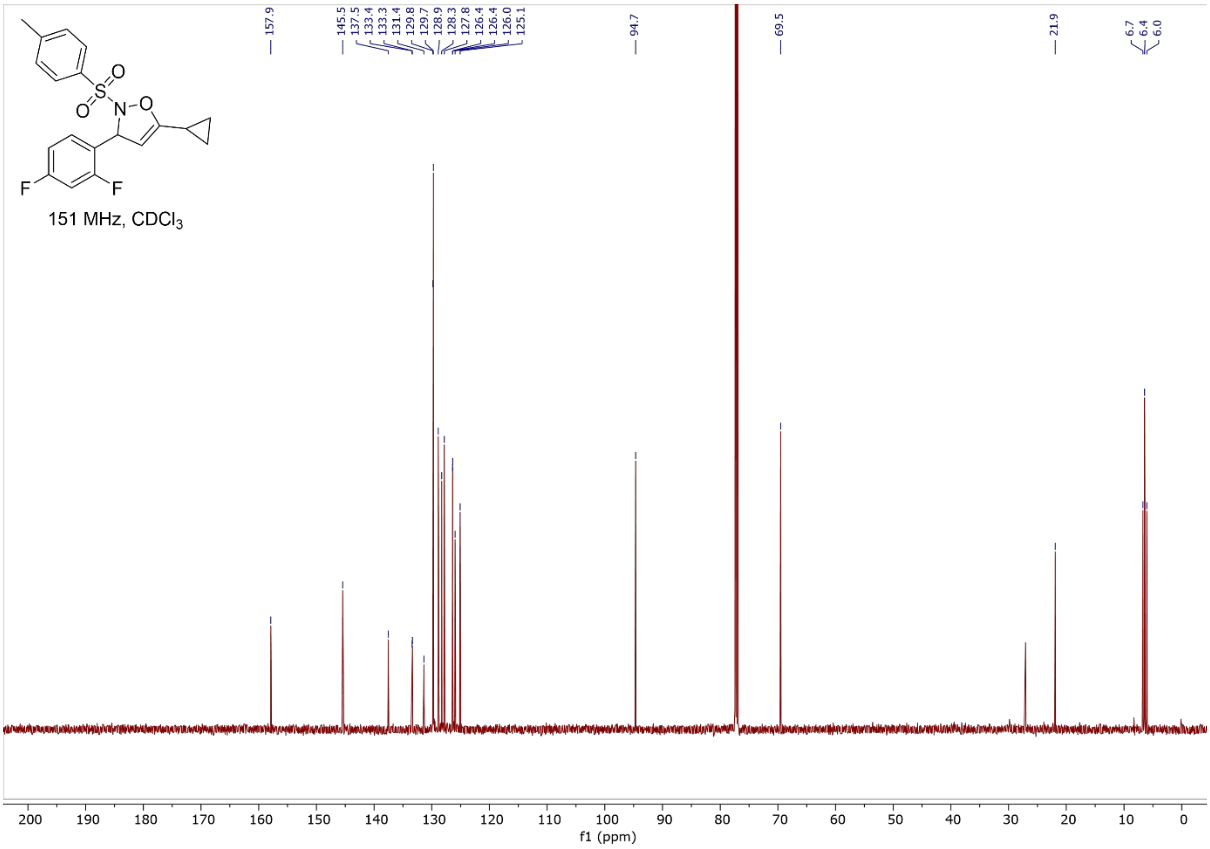

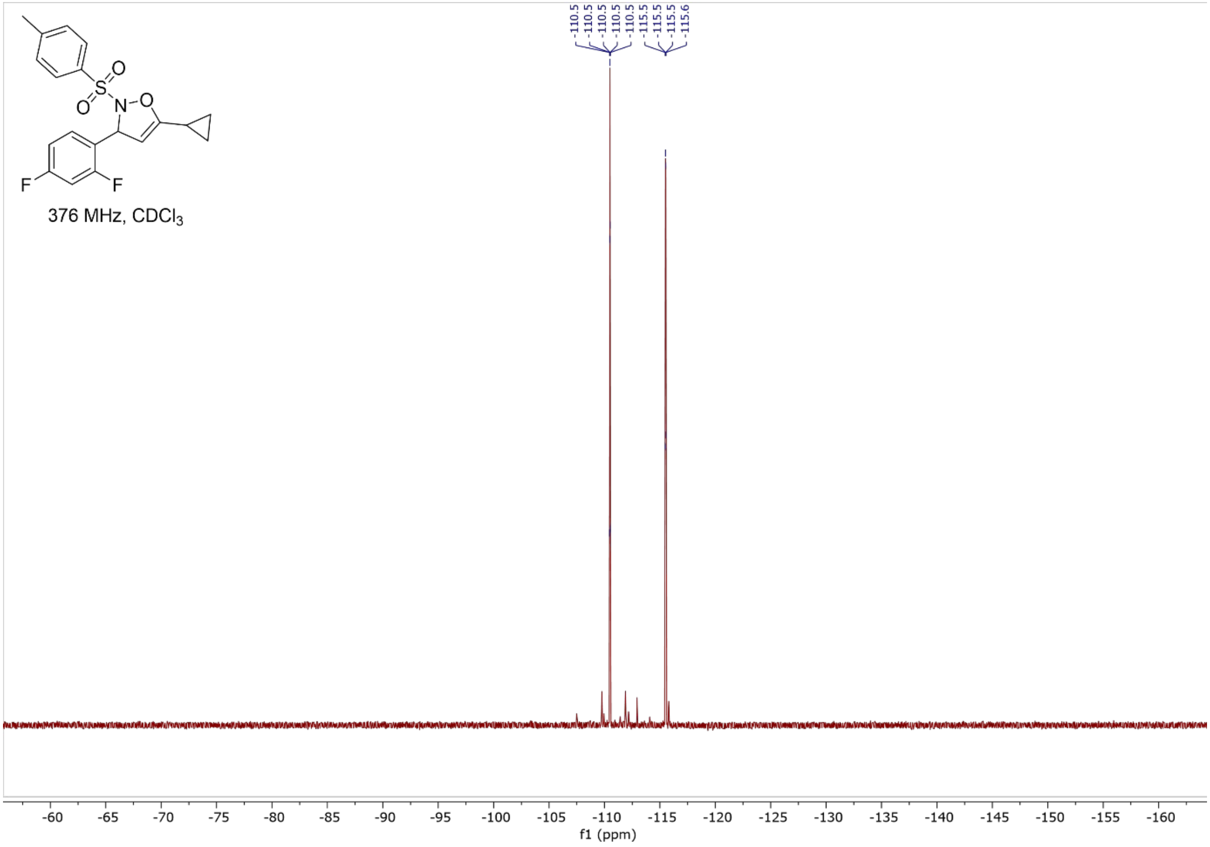

3-(4-Bromophenyl)-5-cyclopropyl-2-tosyl-2,3-dihydroisoxazole, 11m

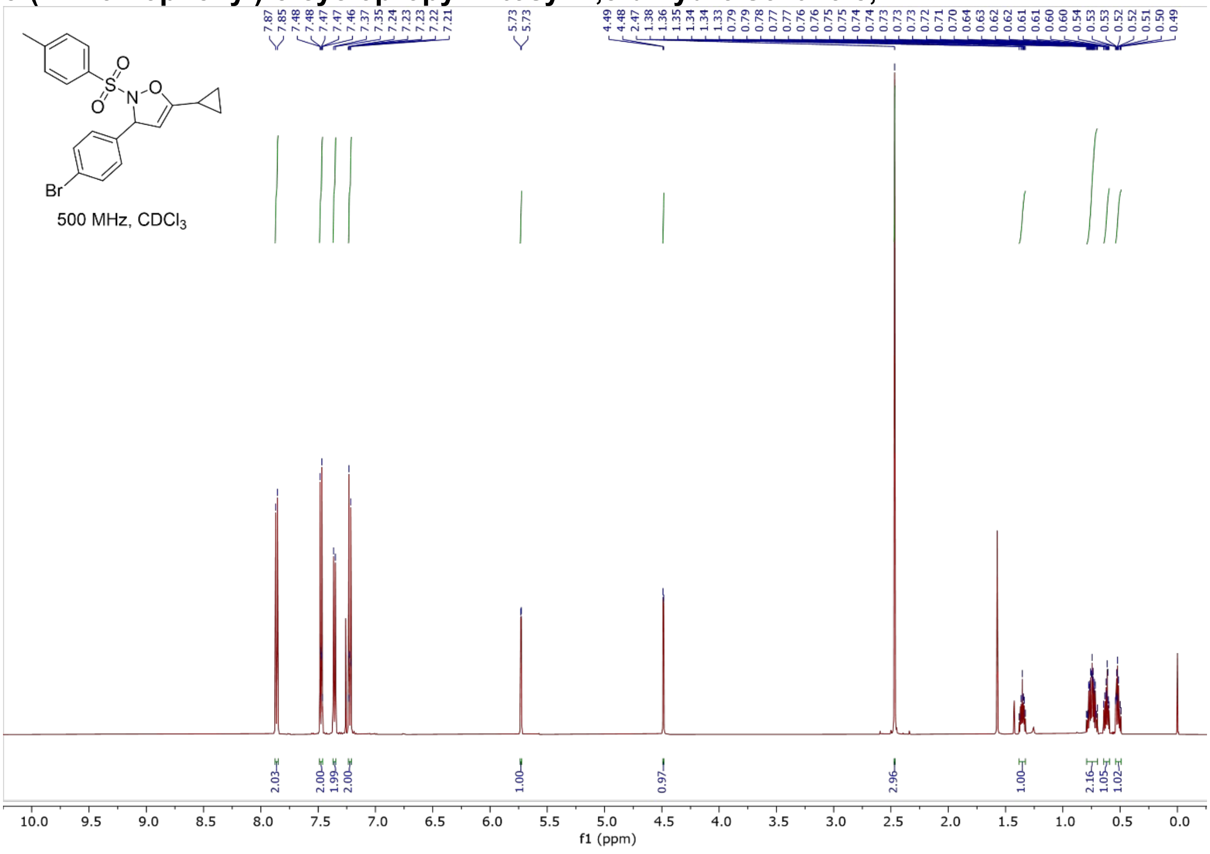

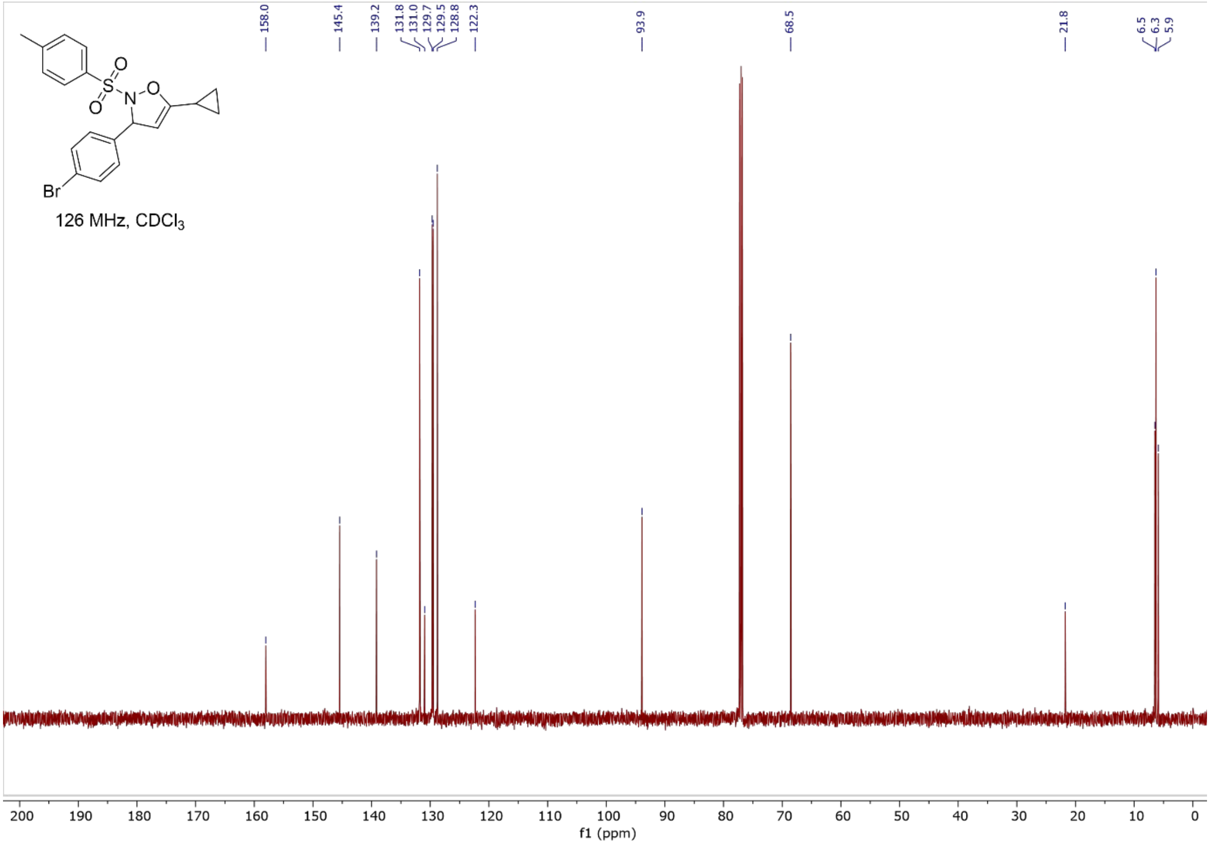

5-Cyclopropyl-3-(naphthalen-2-yl)-2-tosyl-2,3-dihydroisoxazole, 11n

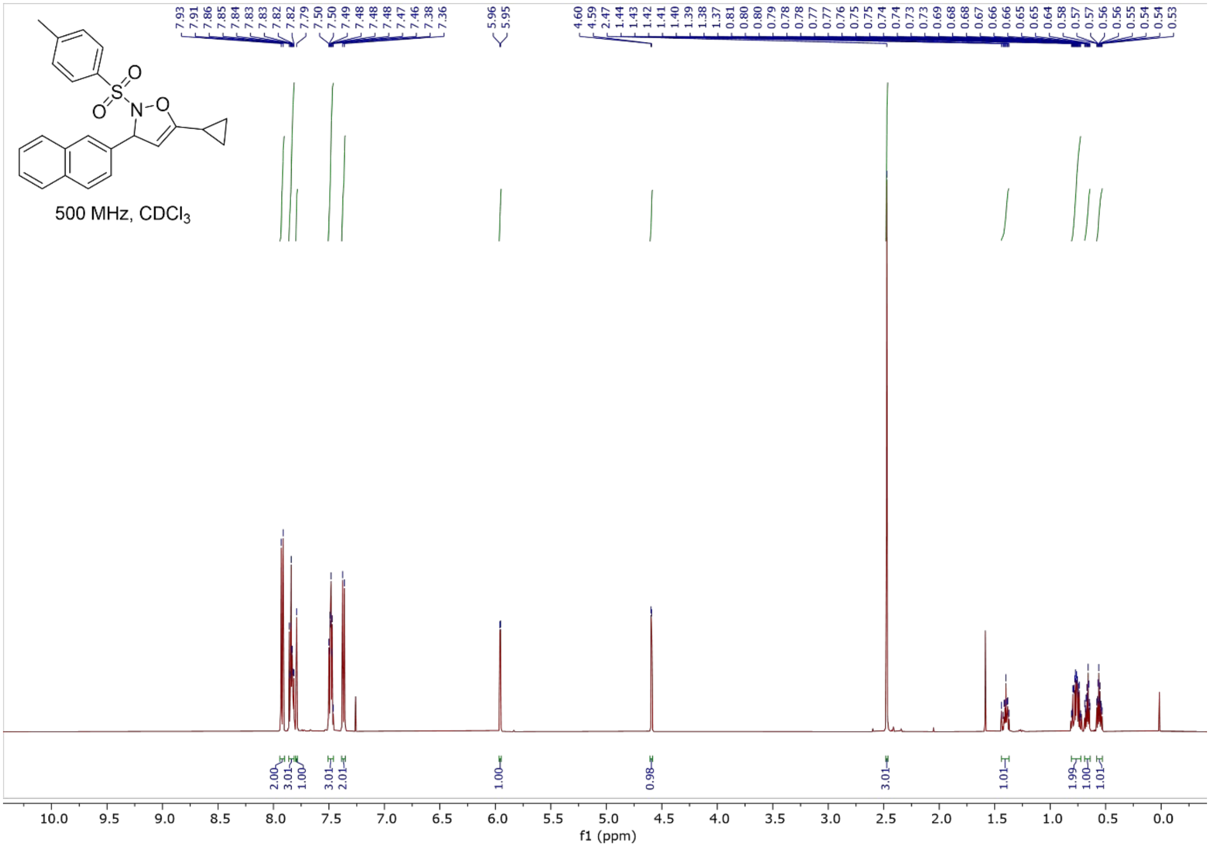

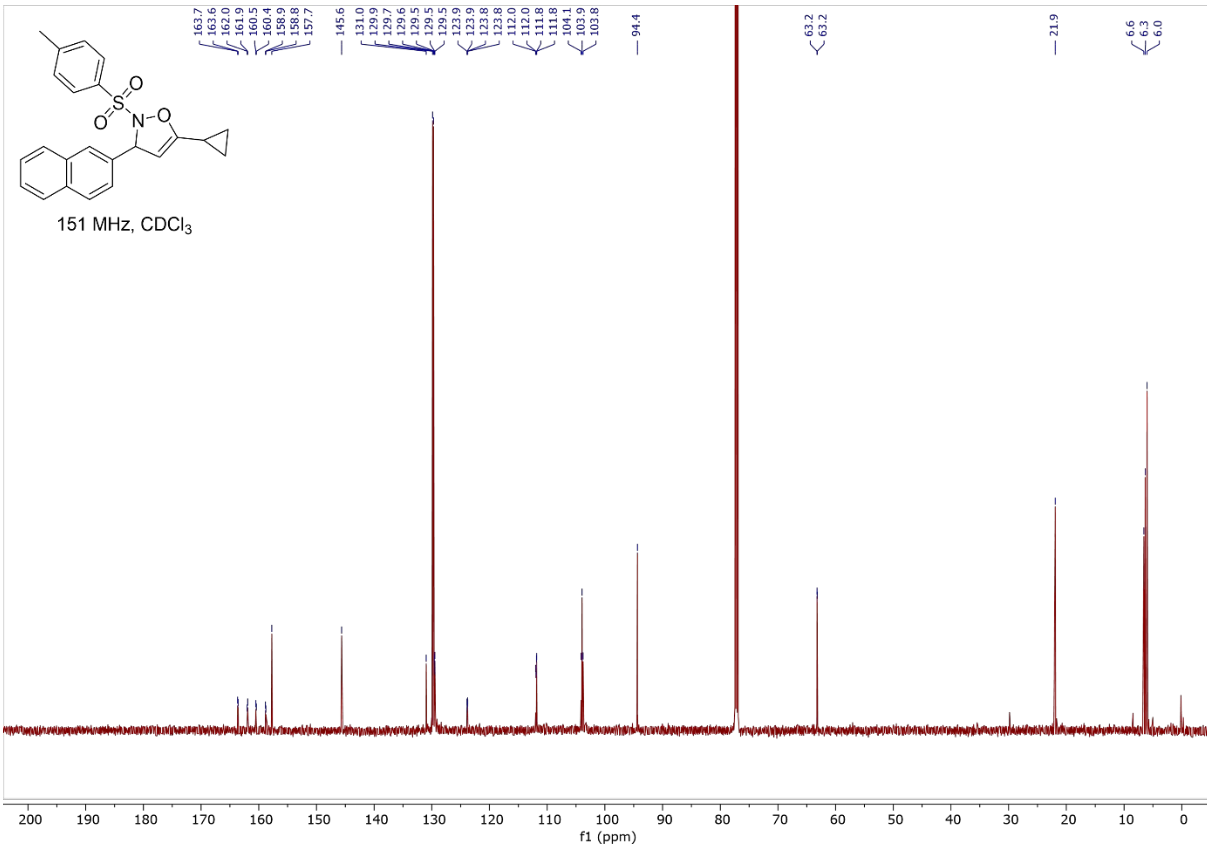

3-(2,4-Difluorophenyl)-5-phenyl-2-tosyl-2,3-dihydroisoxazole, 11o

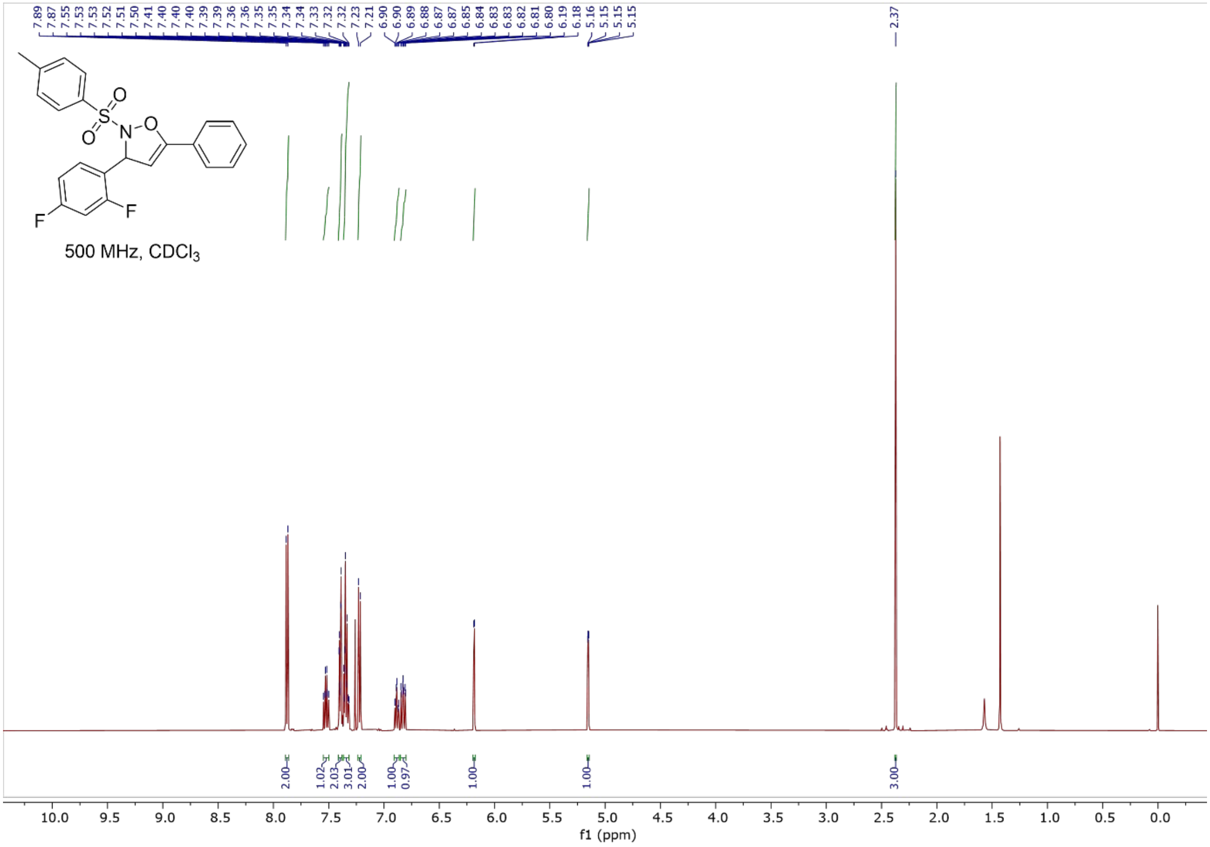

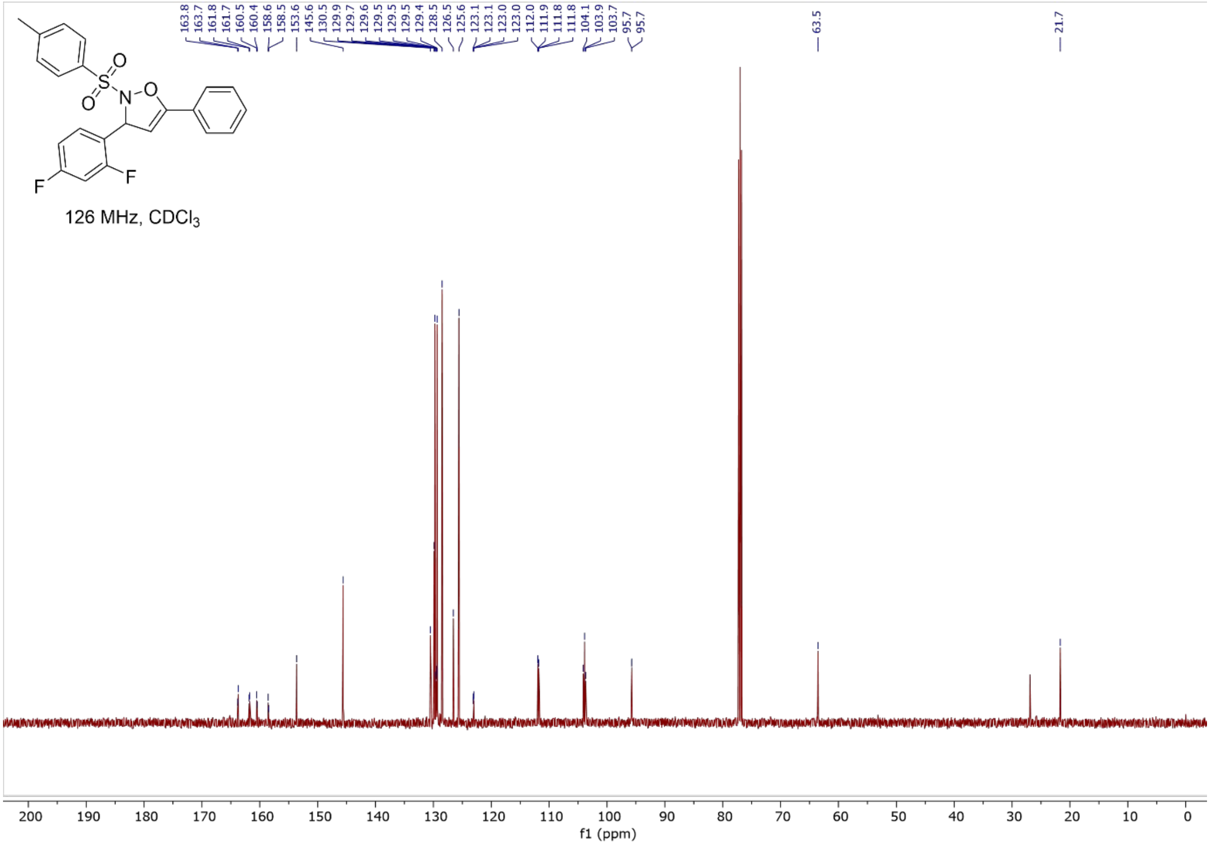
163.8
163.7
161.8
161.7
160.5
160.4
158.6
158.5
153.6
145.6
130.5
129.9
129.7
129.7
129.6
129.5
129.5
129.5
129.4
128.5
126.5
125.6
123.1
123.1
123.0
123.0
112.0
111.9
111.8
111.8
104.1
103.9
103.7
95.7
95.7
63.5
21.7
126 MHz, $CDCl_3$
f1 (ppm)

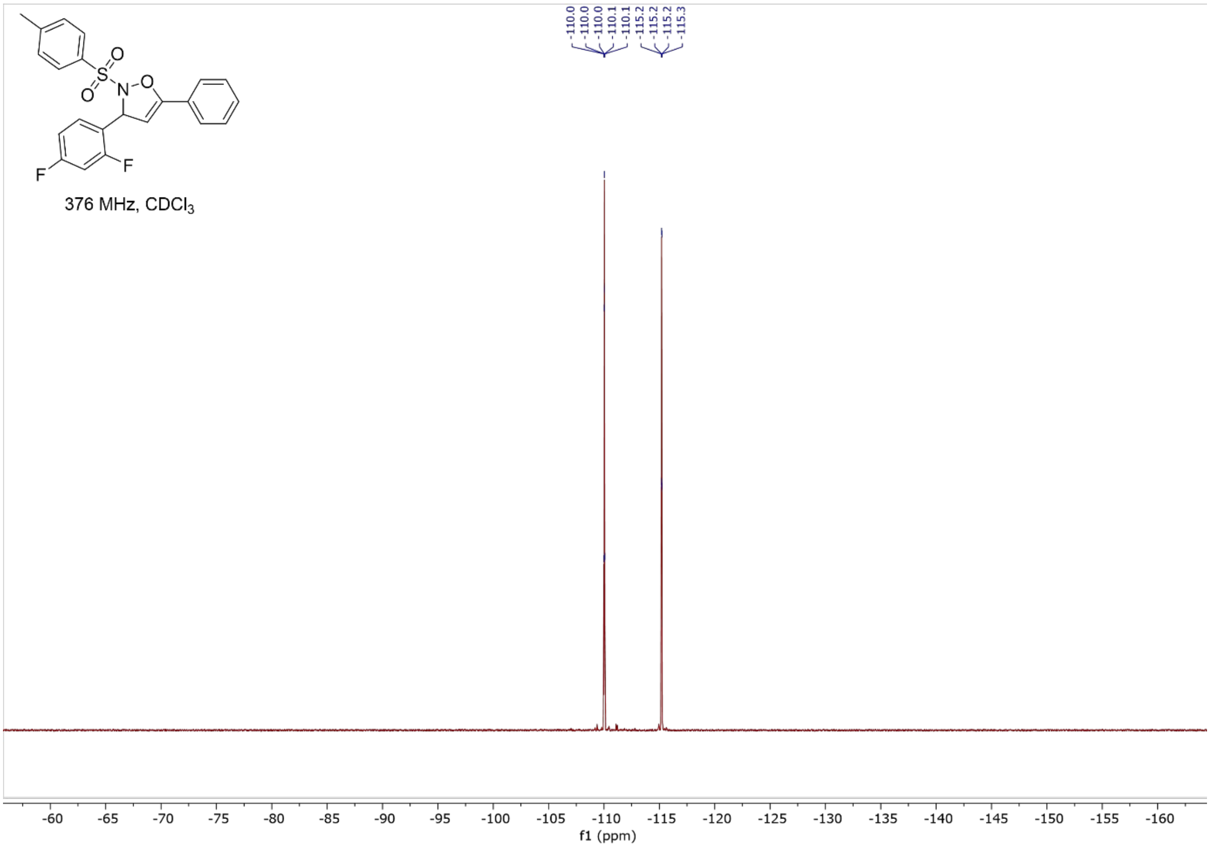
-110.0
-110.0
-110.0
-110.1
-115.2
-115.2
-115.2
-115.3
376 MHz, $CDCl_3$
f1 (ppm)

3-(4-Fluorophenyl)-5-phenyl-2-tosyl-2,3-dihydroisoxazole, 11p

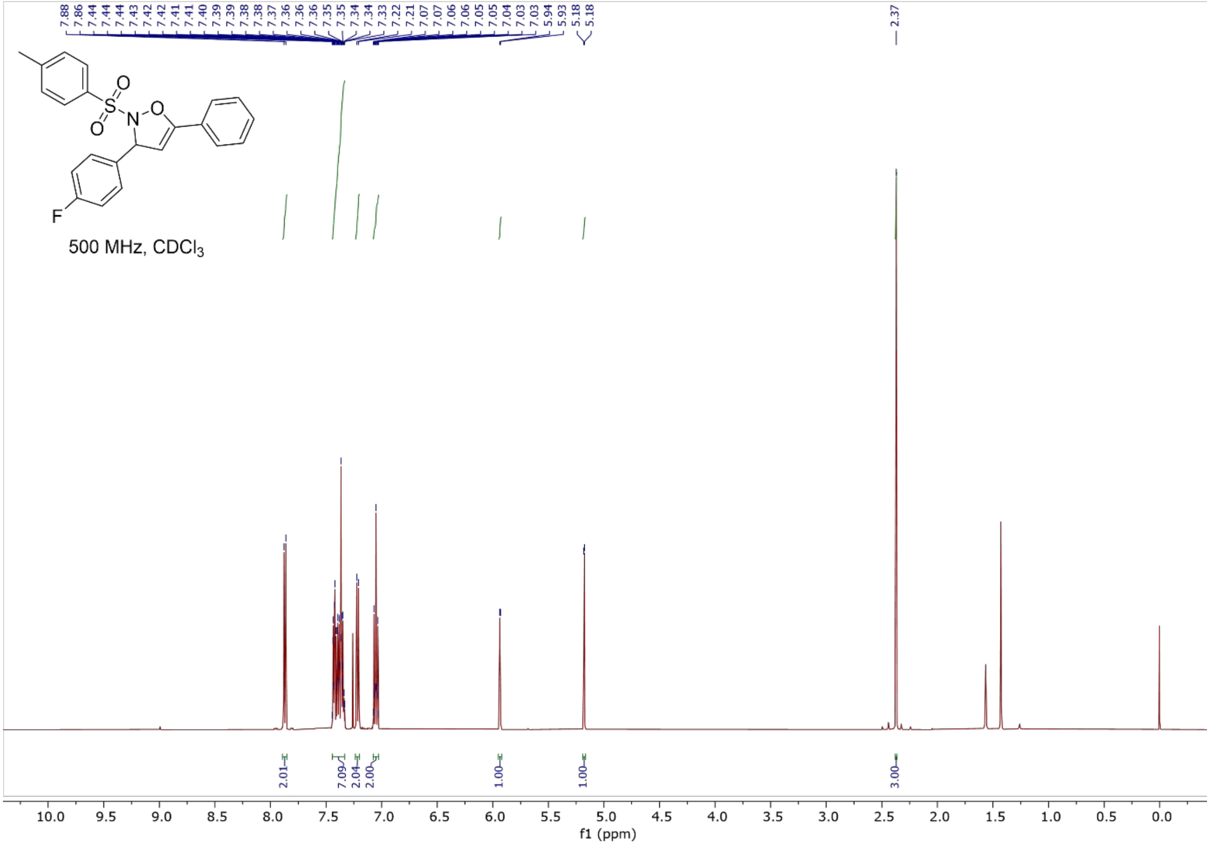

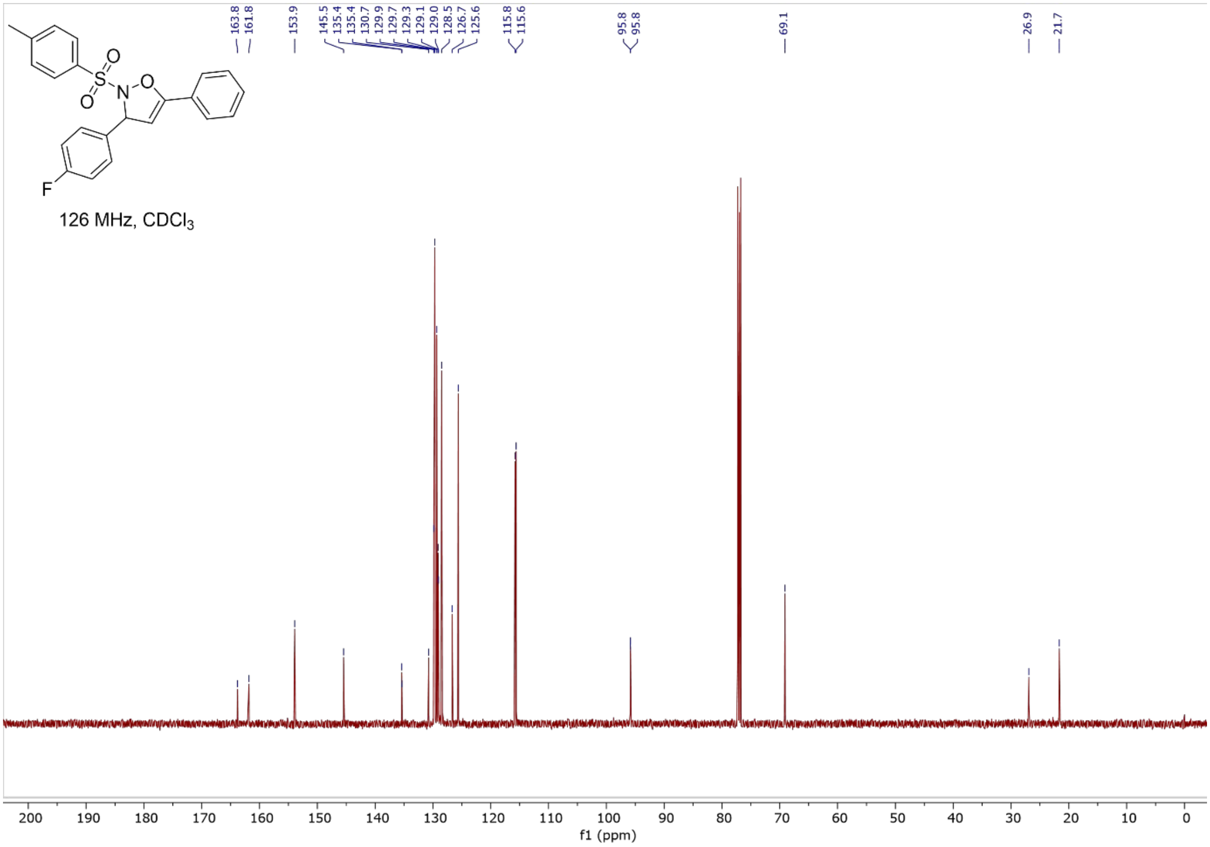

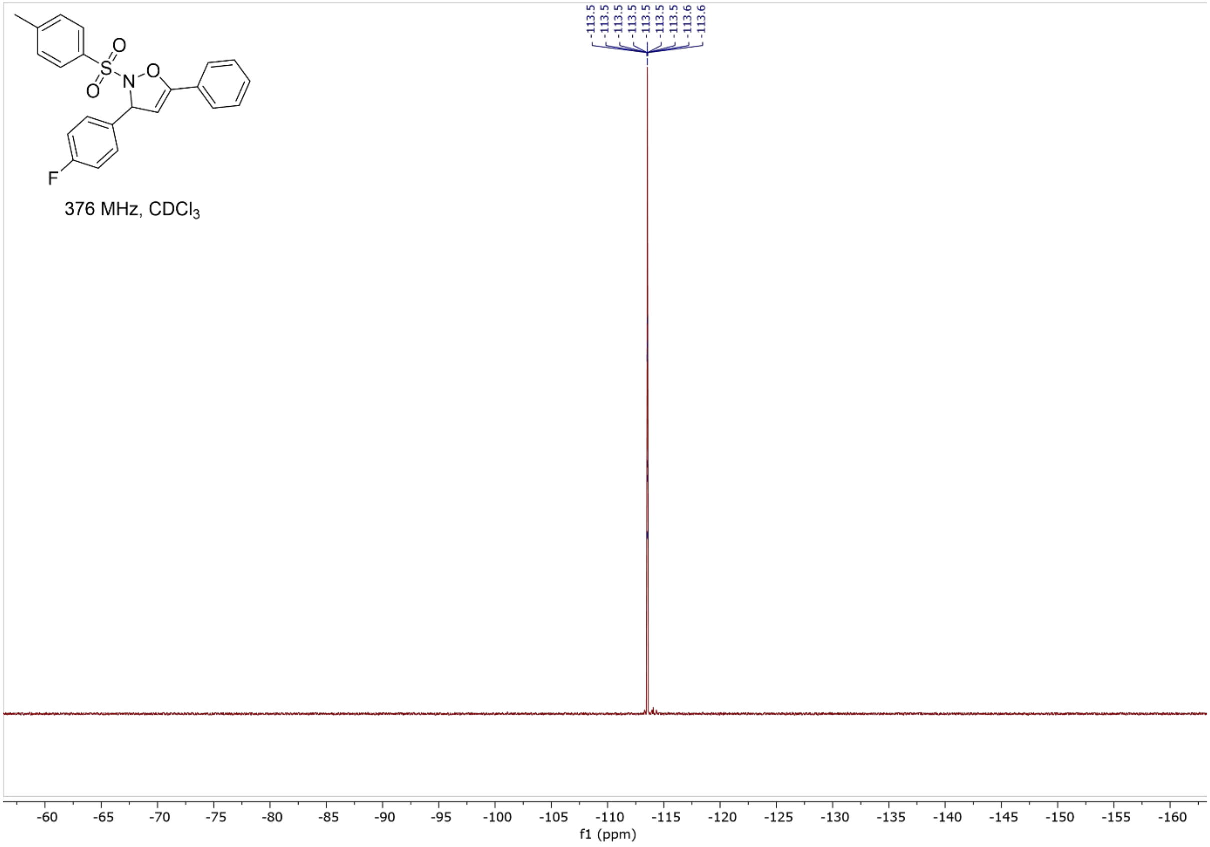

3-(4-Bromophenyl)-5-phenyl-2-tosyl-2,3-dihydroisoxazole, 11q

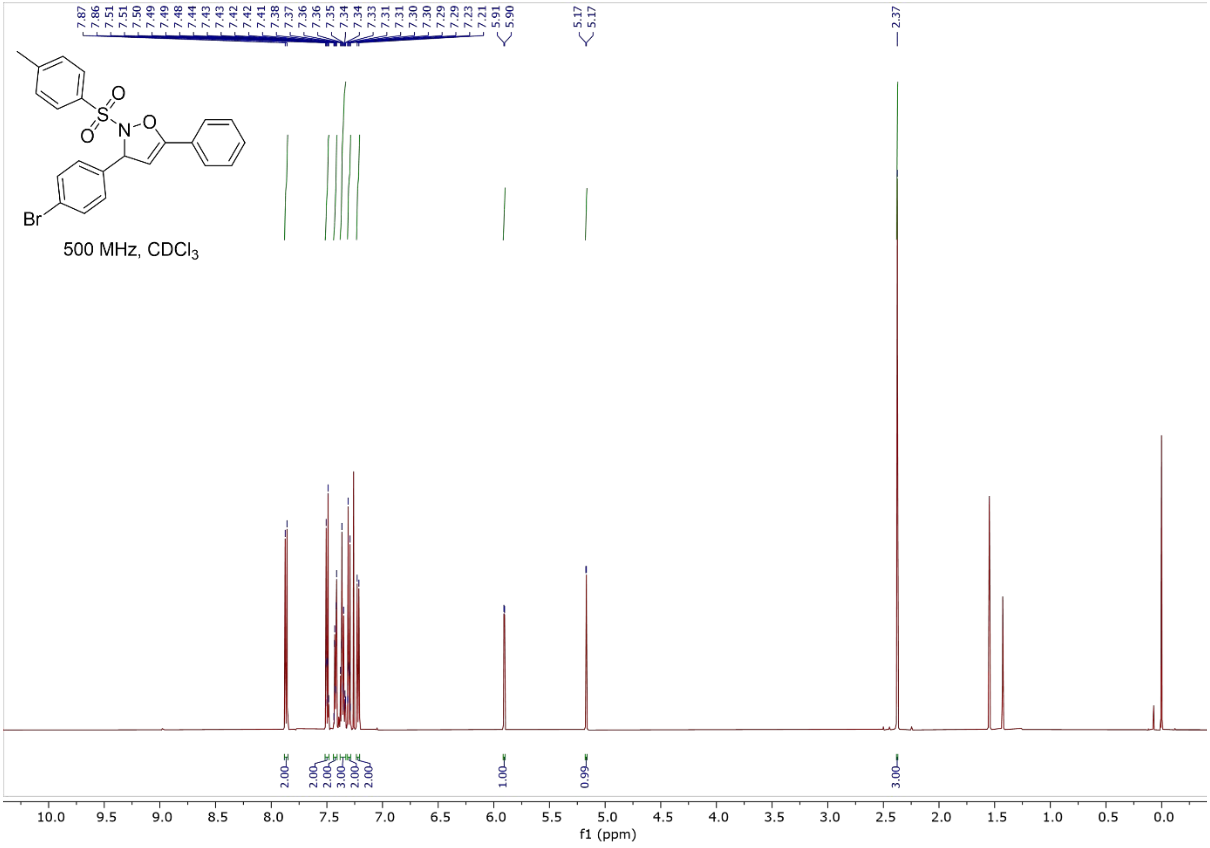

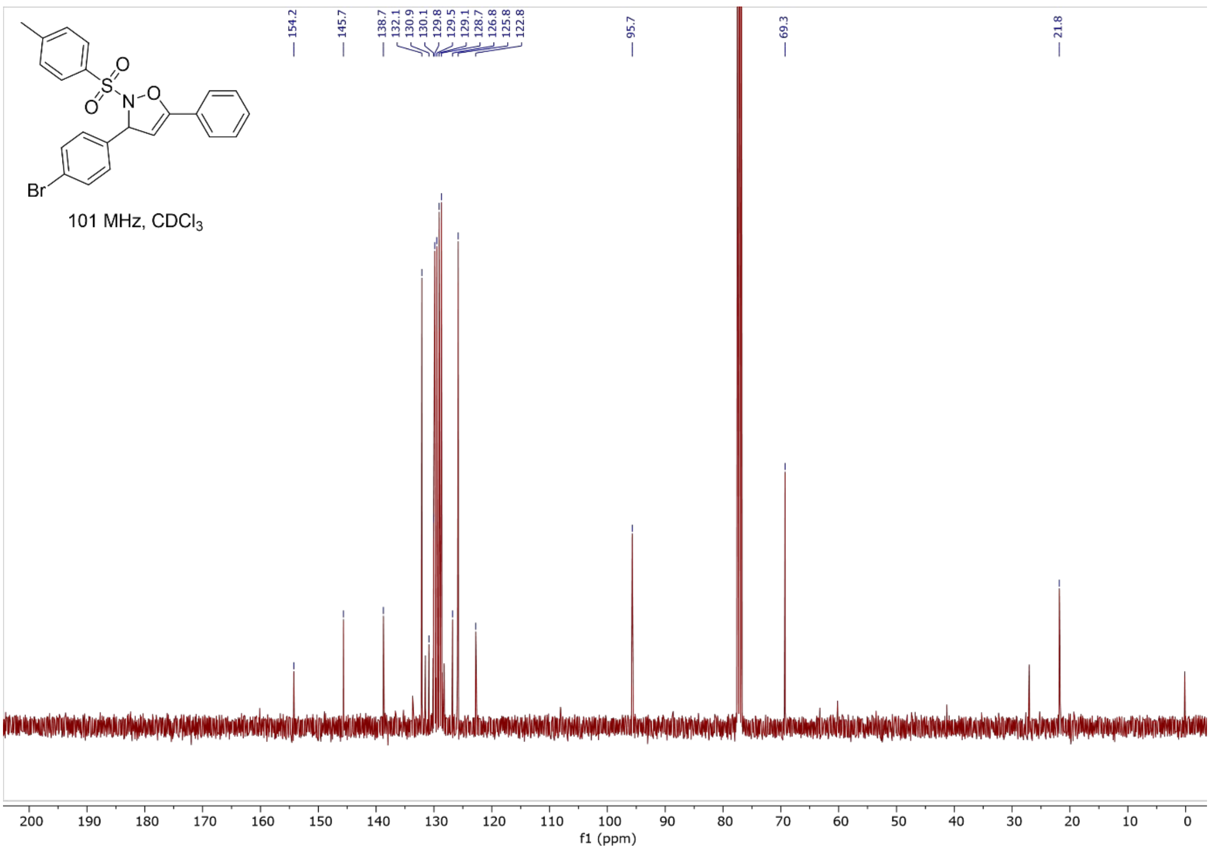

3-(Naphthalen-2-yl)-5-phenyl-2-tosyl-2,3-dihydroisoxazole, 11r

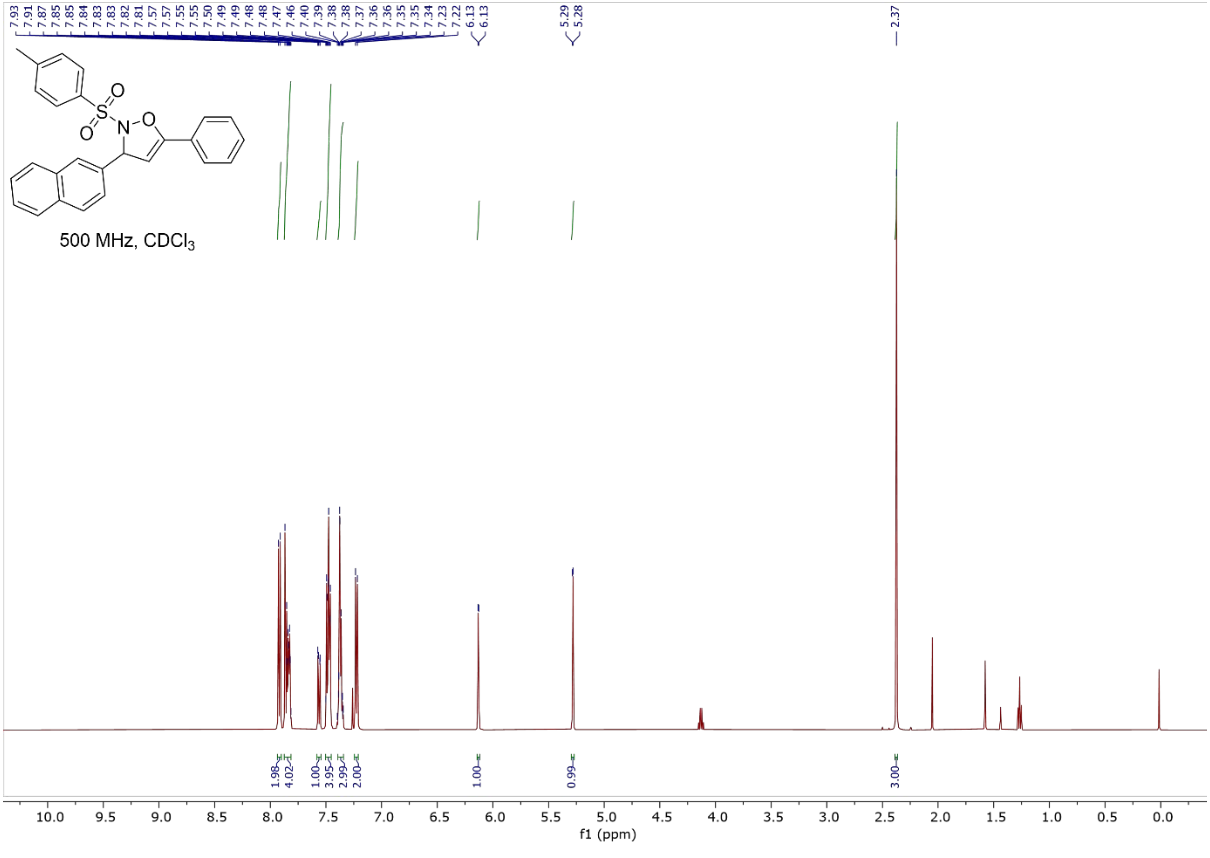

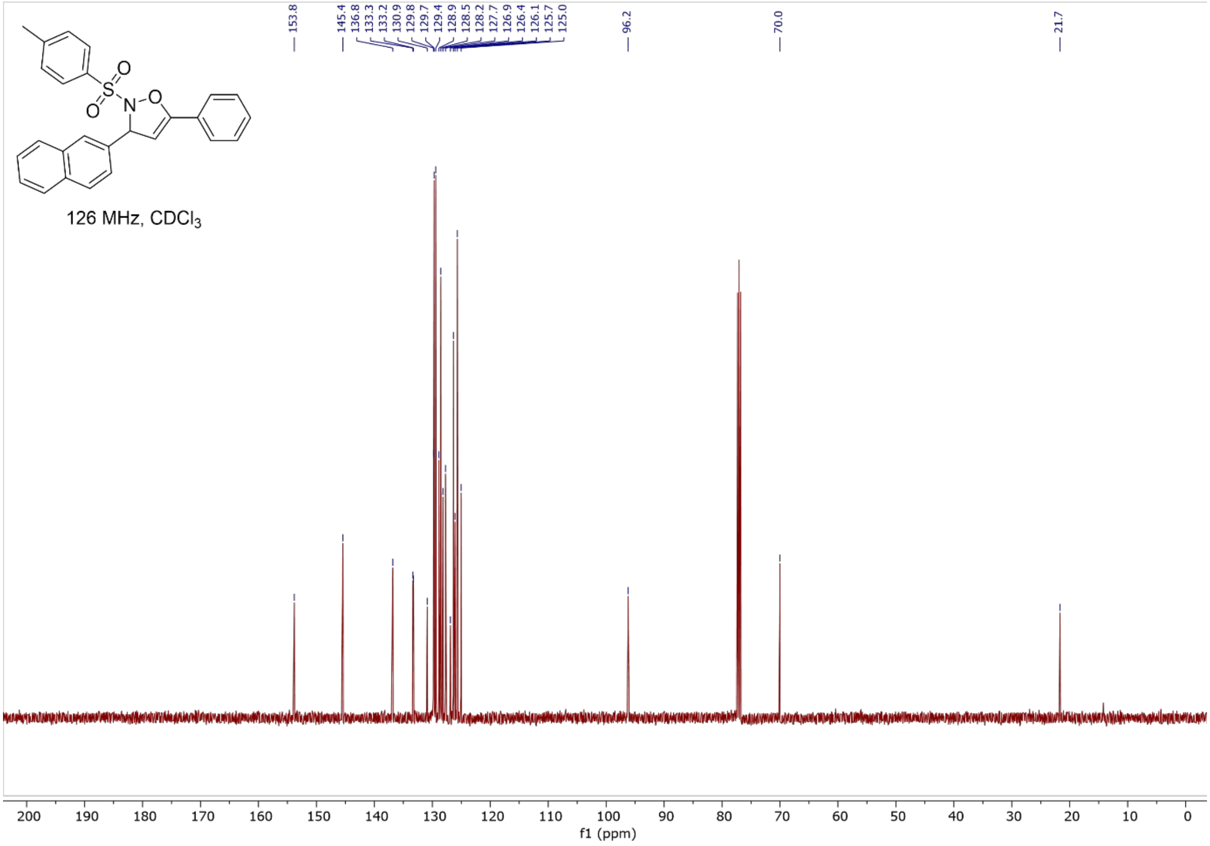

5-Butyl-3-(3-methylthiophen-2-yl)-2-tosyl-2,3-dihydroisoxazole, 11j

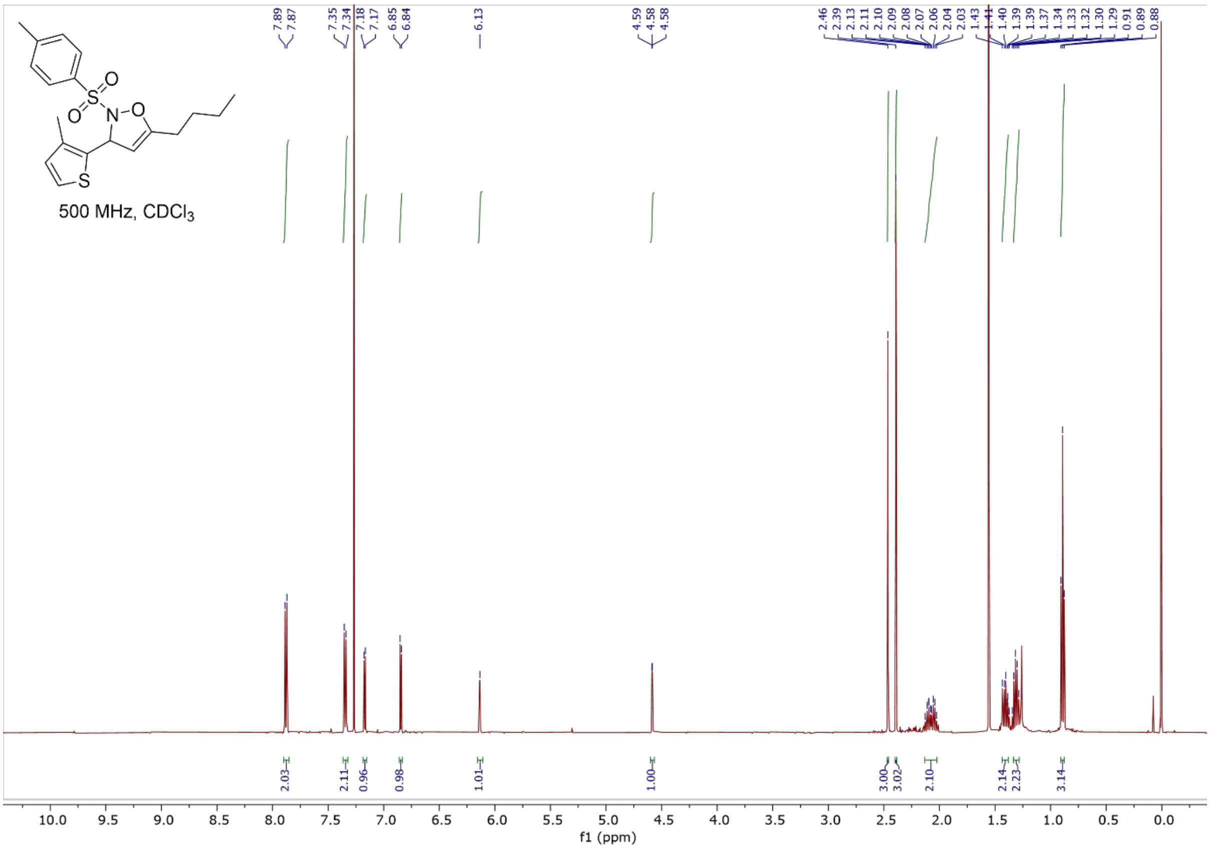

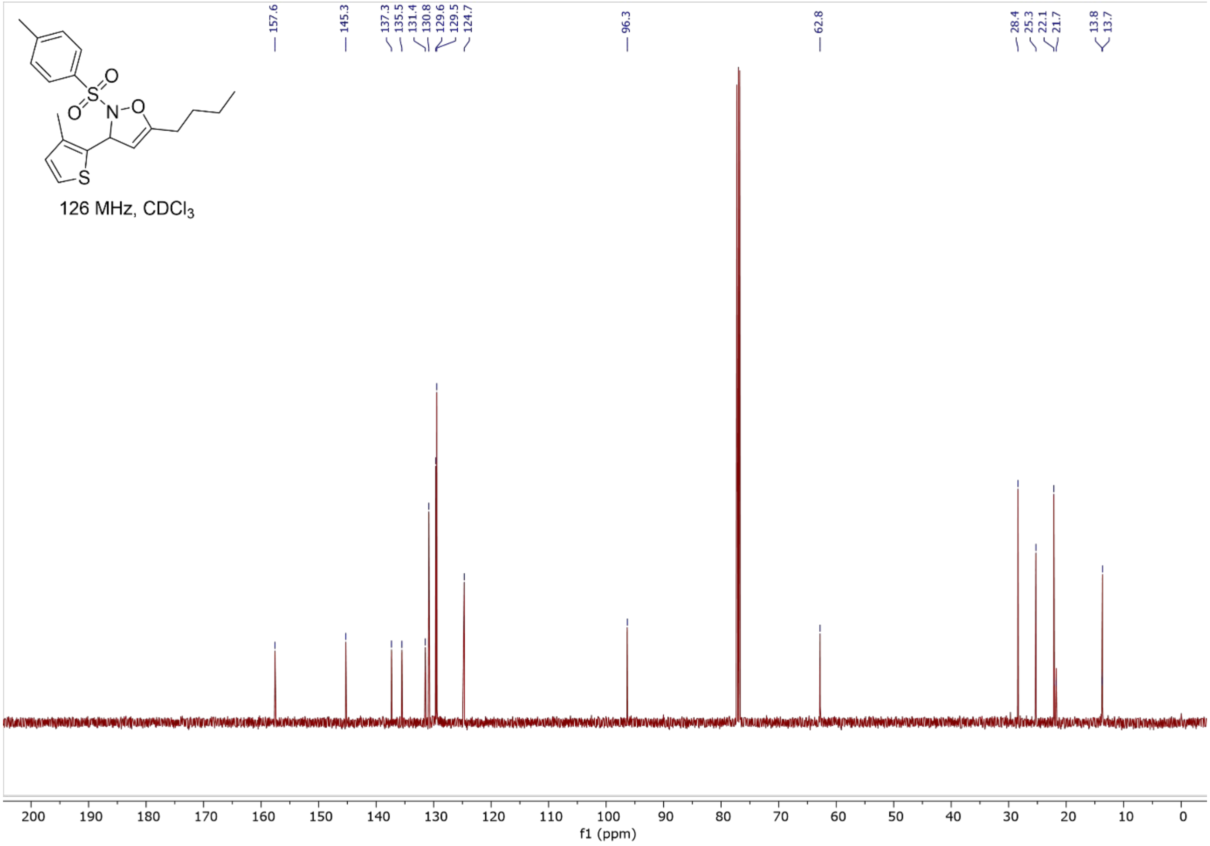

1-((2*R**,3*R**)-3-(2,4-Difluorophenyl)-1-tosylaziridin-2-yl)pentan-1-one, 12a

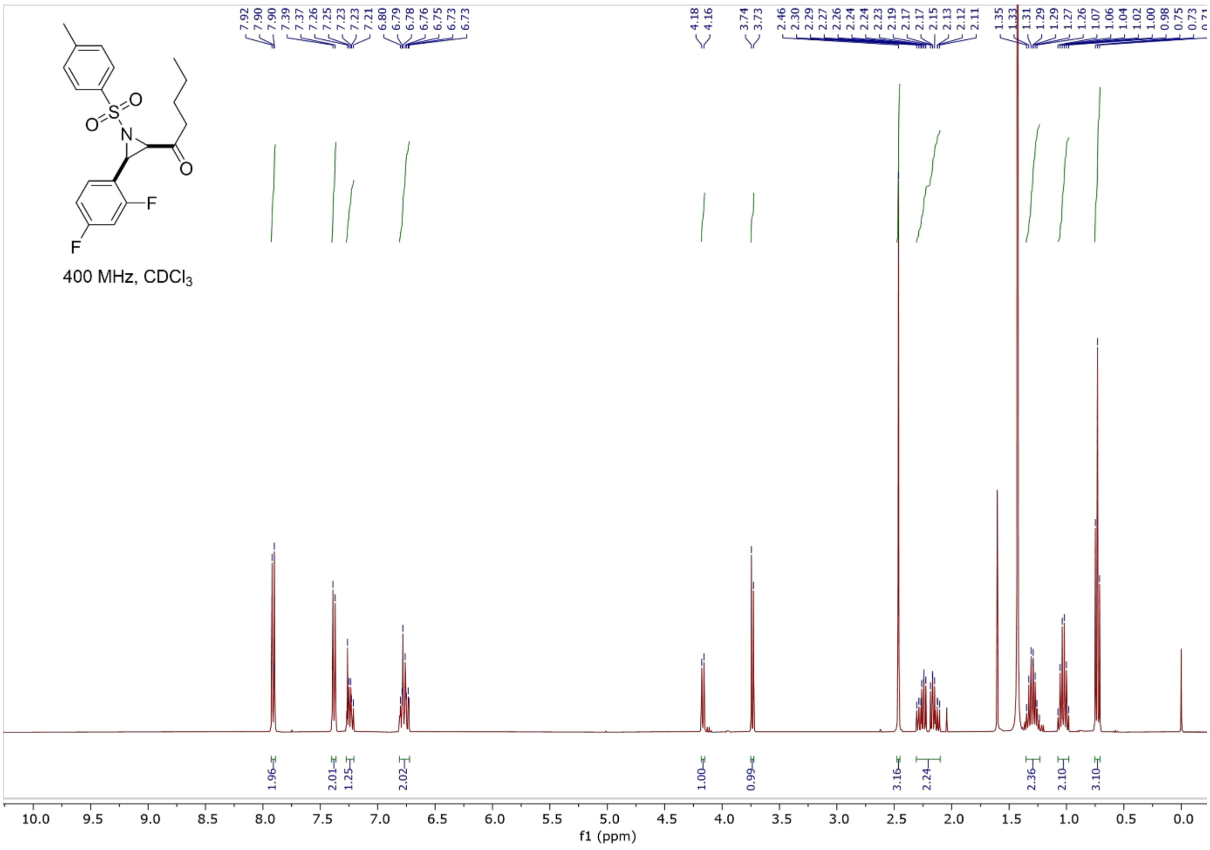

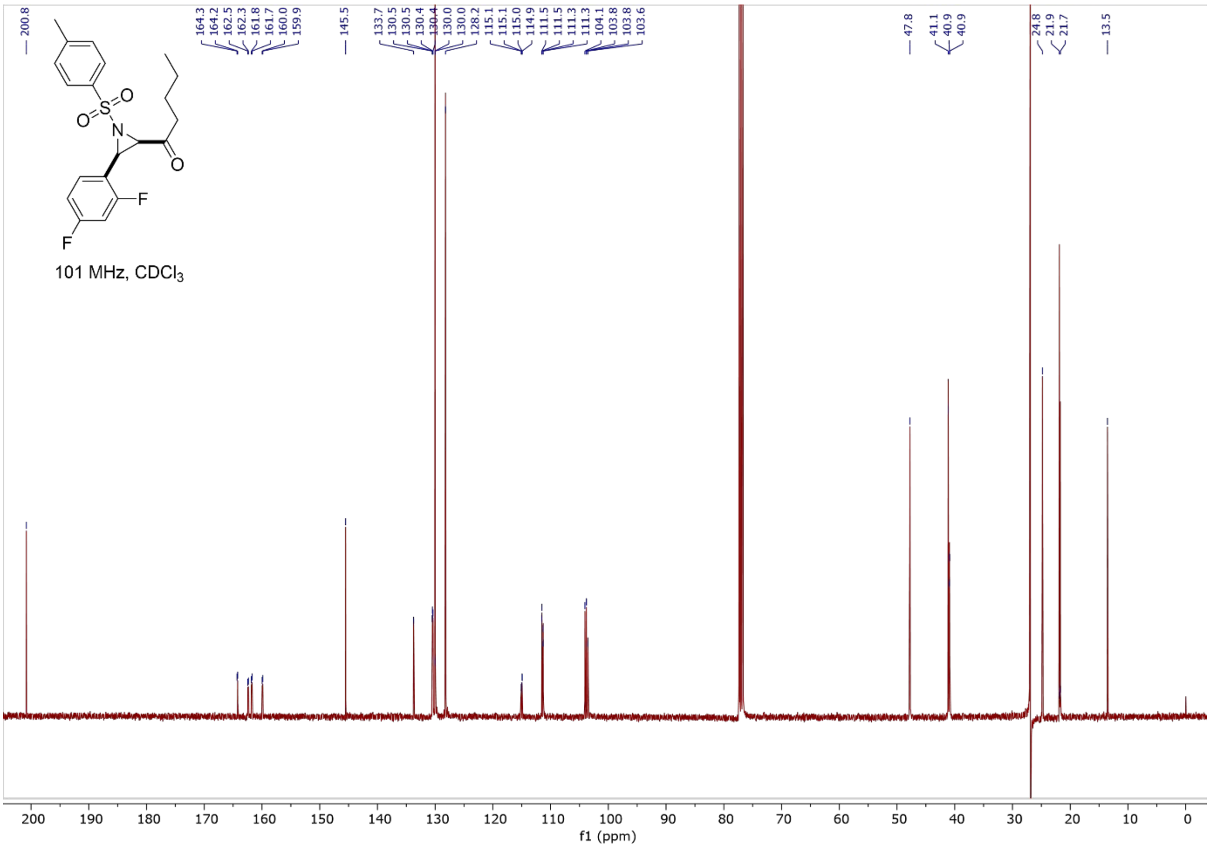
200.8
164.3
164.2
162.5
162.3
161.8
161.7
160.0
159.9
145.5
133.7
130.5
130.5
130.4
130.4
130.0
130.0
128.2
115.1
115.1
115.0
114.9
111.5
111.5
111.3
111.3
104.1
103.8
103.8
103.6
47.8
41.1
40.9
40.9
24.8
21.9
21.7
13.5
O
S
O
N
O
F
F
101 MHz, CDCl3
200
190
180
170
160
150
140
130
120
110
100
90
80
70
60
50
40
30
20
10
0
f1 (ppm)

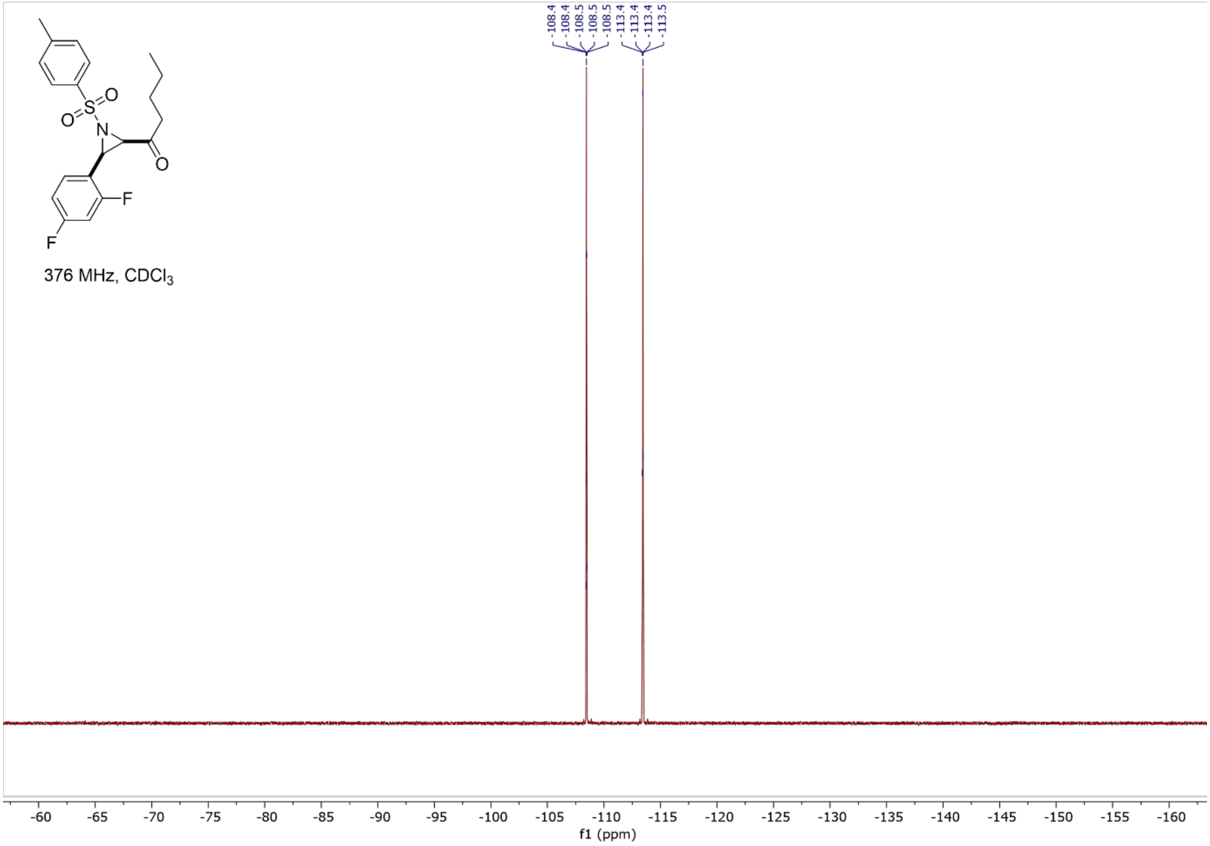
-108.4
-108.4
-108.5
-108.5
-108.5
-113.4
-113.4
-113.4
-113.5
O
S
O
N
O
F
F
376 MHz, CDCl3
-60
-65
-70
-75
-80
-85
-90
-95
-100
-105
-110
-115
-120
-125
-130
-135
-140
-145
-150
-155
-160
f1 (ppm)

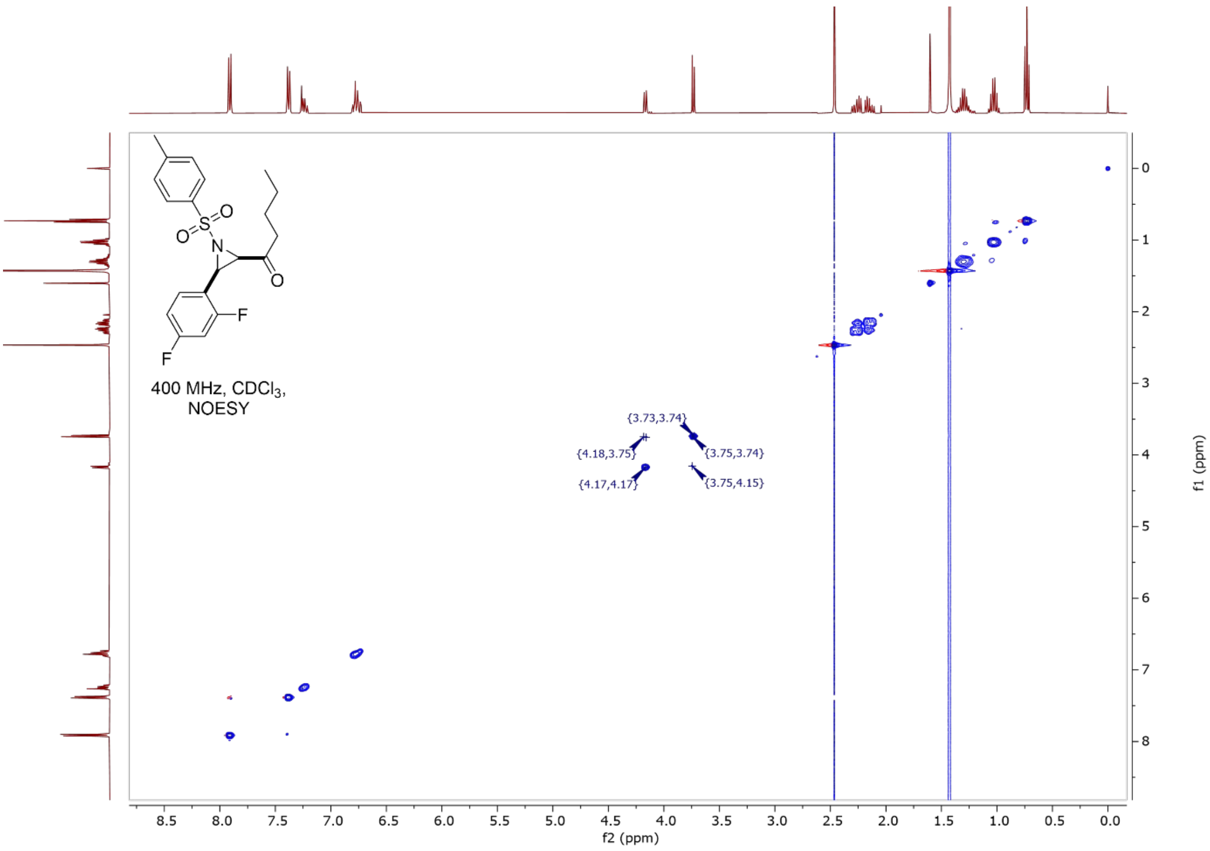

1-((2*R,3*R**)-3-(4-Fluorophenyl)-1-tosylaziridin-2-yl)pentan-1-one, 12b**

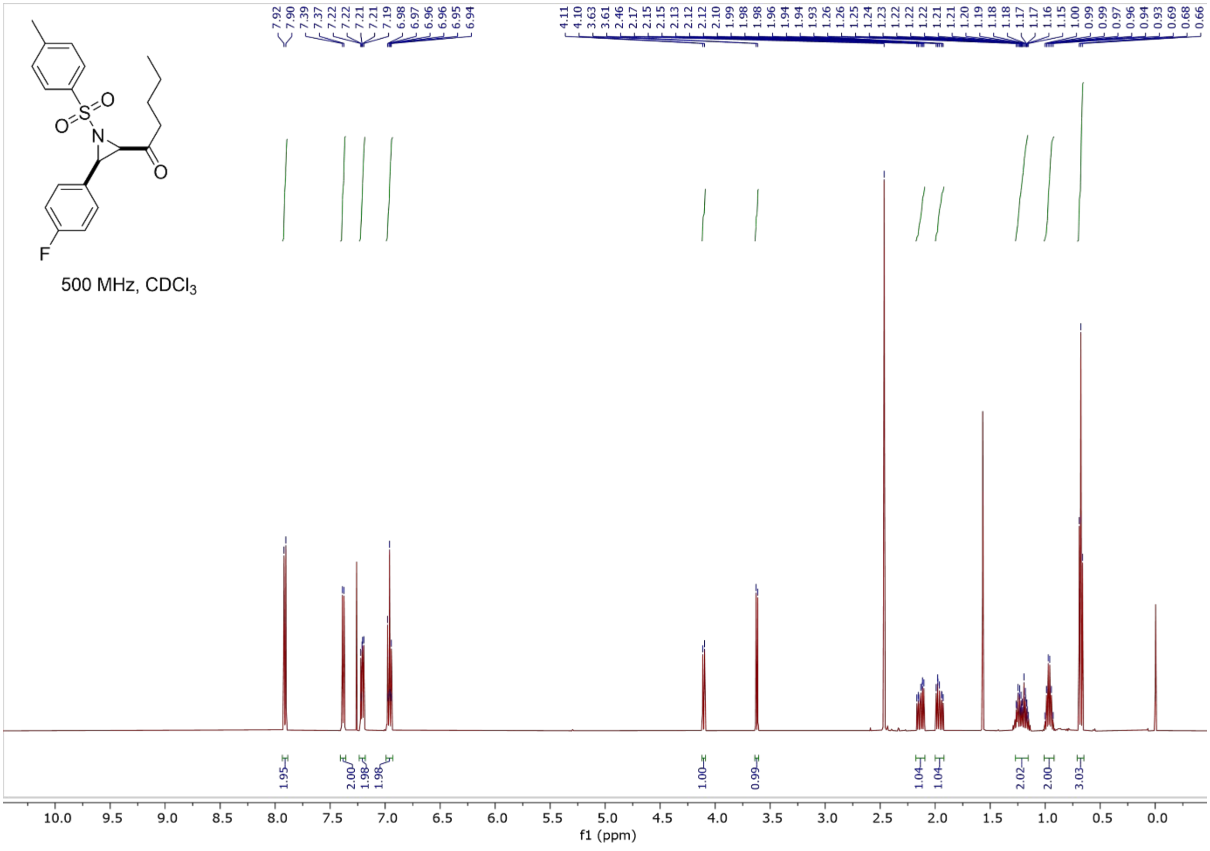

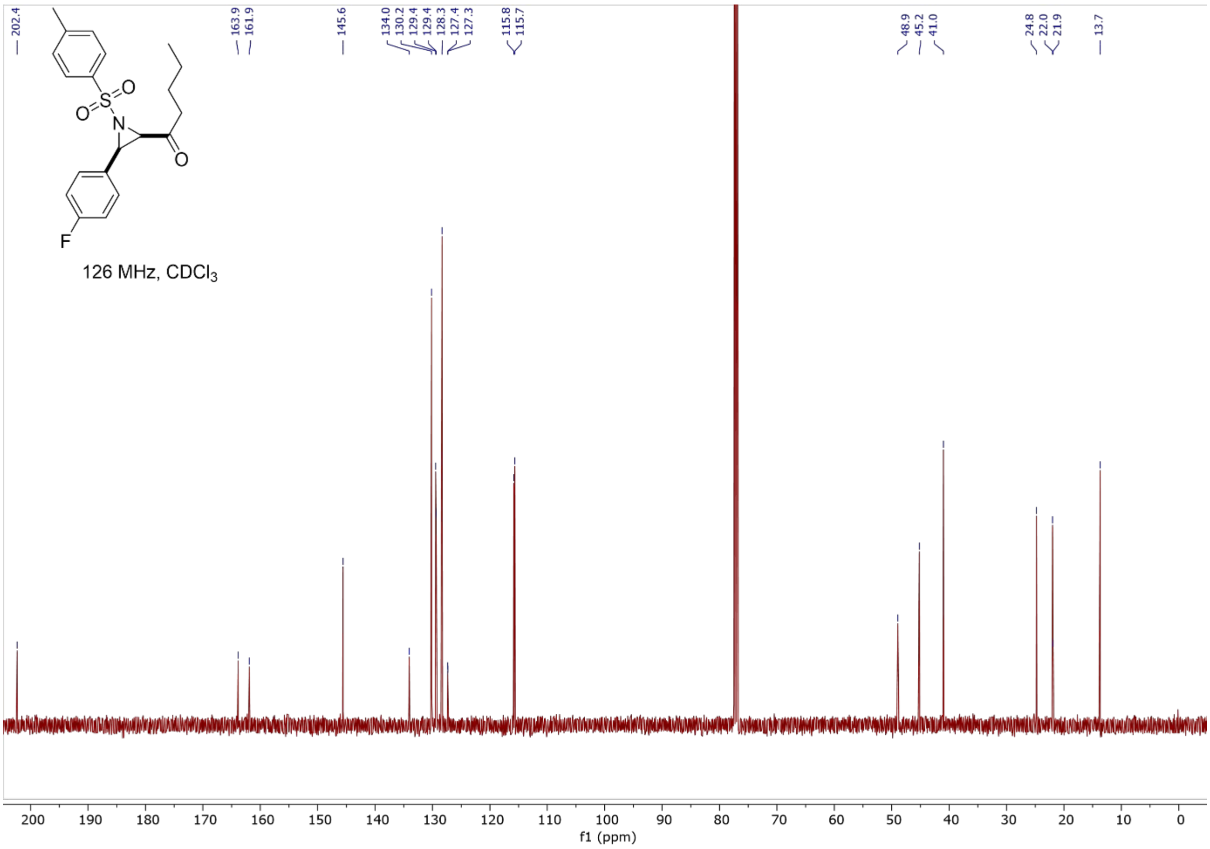
202.4
163.9
161.9
145.6
134.0
130.2
129.4
129.4
128.3
127.4
127.3
115.8
115.7
48.9
45.2
41.0
24.8
22.0
21.9
13.7
126 MHz, CDCl3
f1 (ppm)

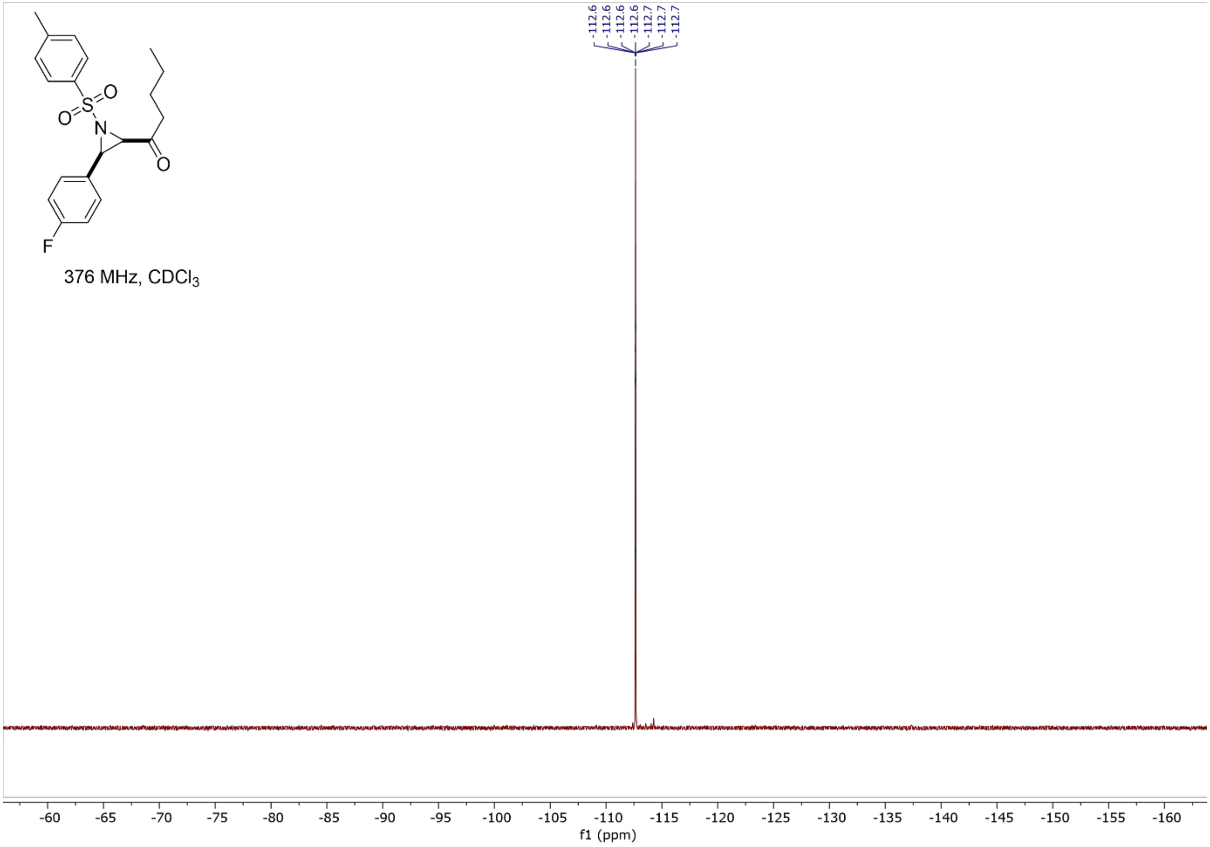
-112.6
-112.6
-112.6
-112.6
-112.7
-112.7
-112.7
376 MHz, CDCl3
f1 (ppm)

1-((2*R,3*R**)-3-(4-Bromophenyl)-1-tosylaziridin-2-yl)pentan-1-one, 12c**

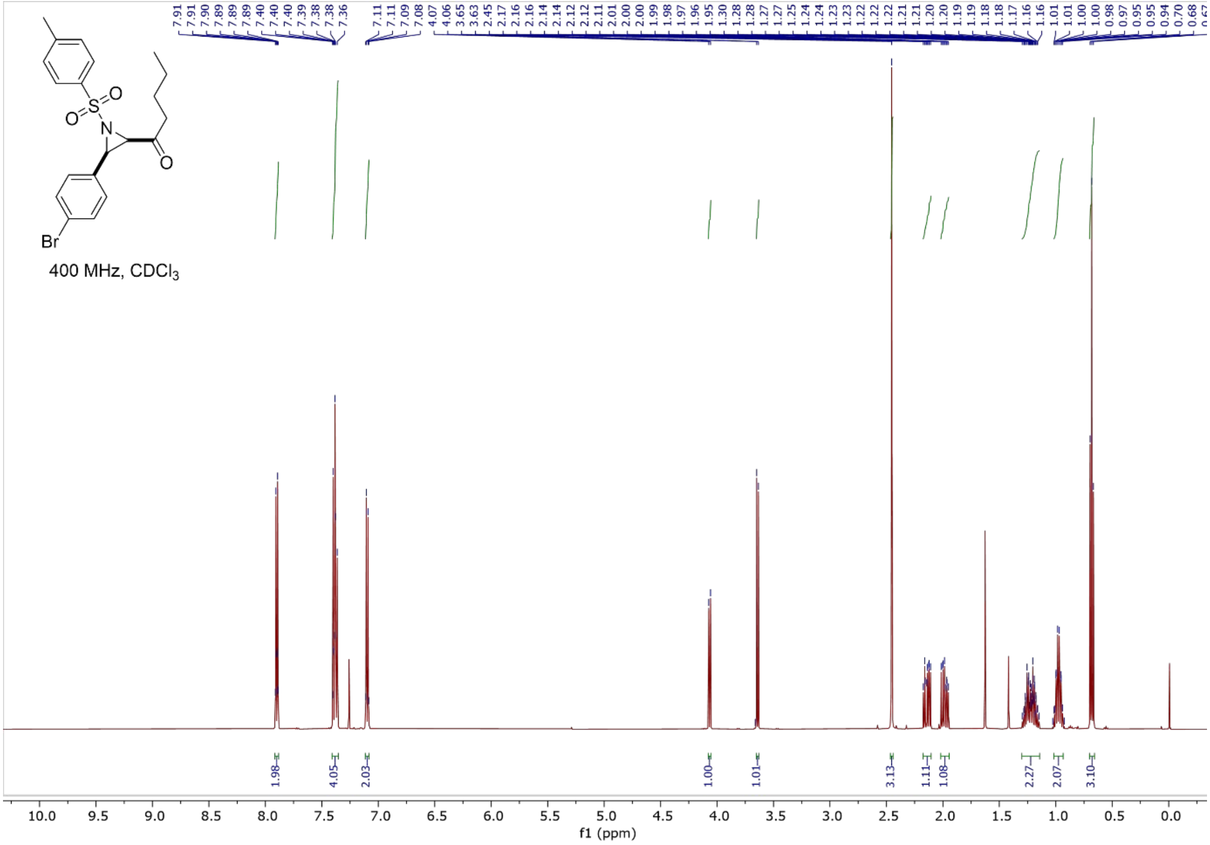

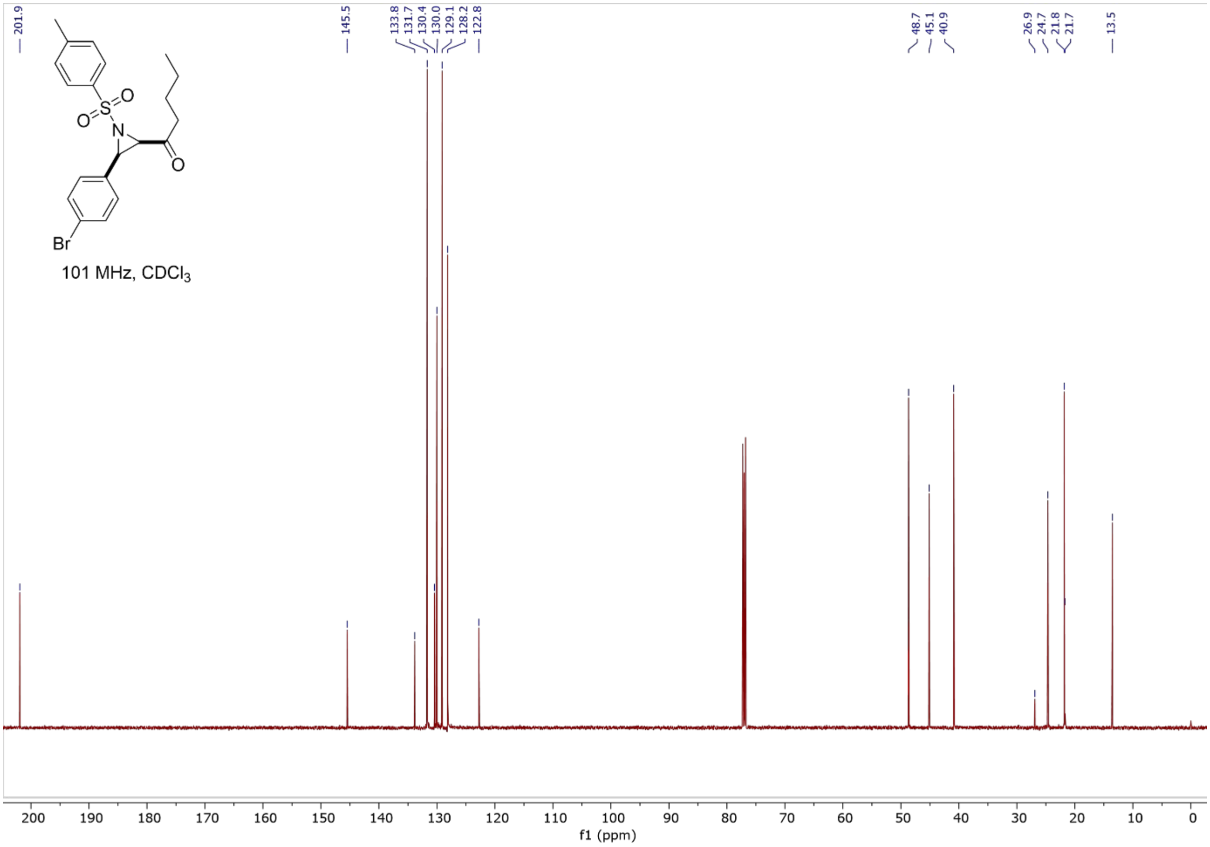

1-((2*R,3*R**)-3-(2-Chlorophenyl)-1-tosylaziridin-2-yl)pentan-1-one, 12d**

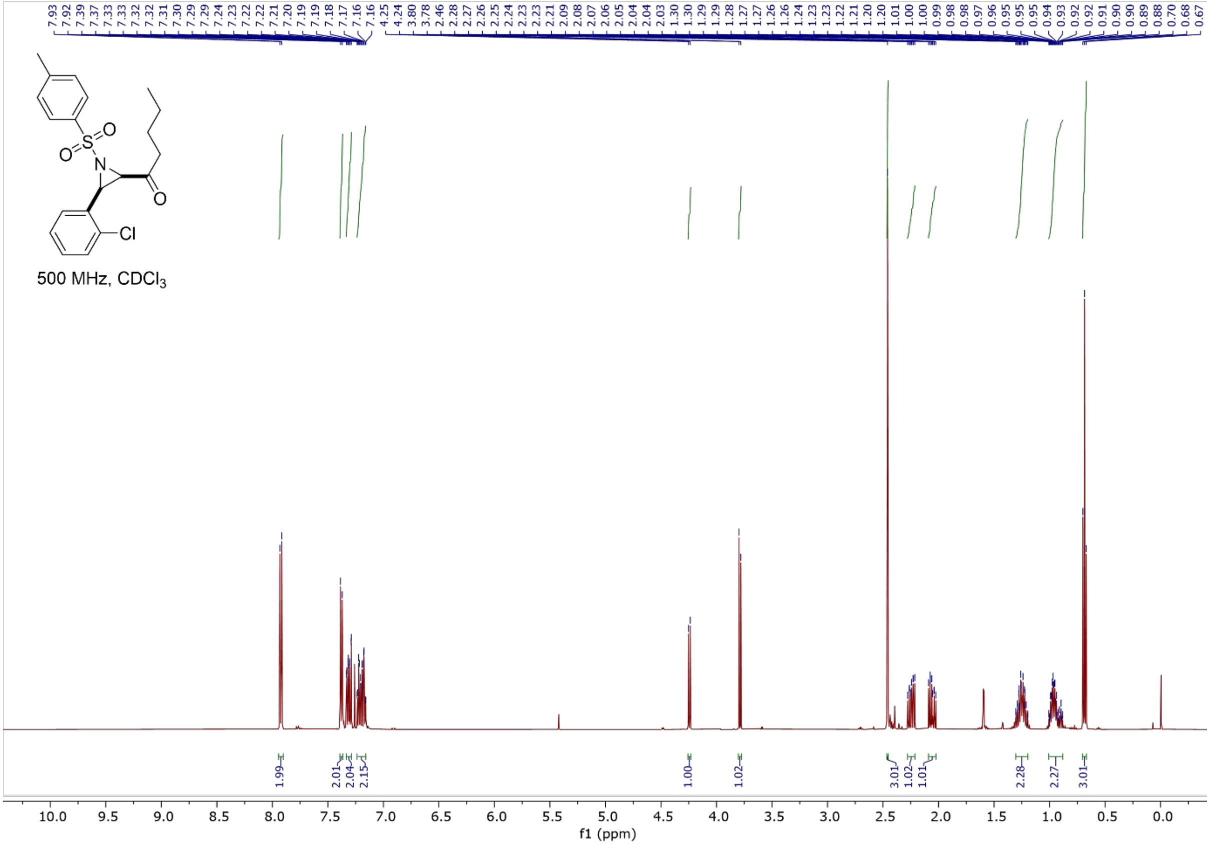

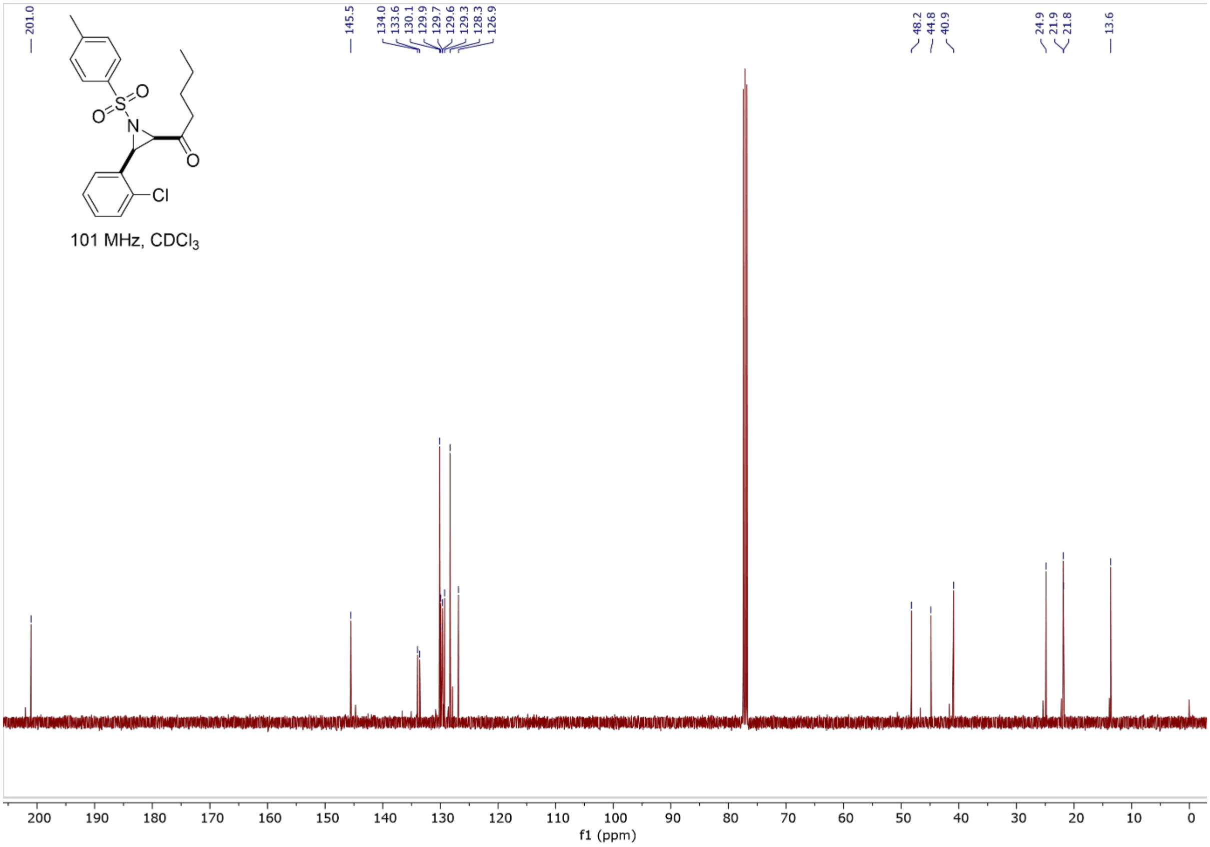

1-((2*R**,3*R**)-3-(3-Chloro-4-fluorophenyl)-1-tosylaziridin-2-yl)pentan-1-one, 12e

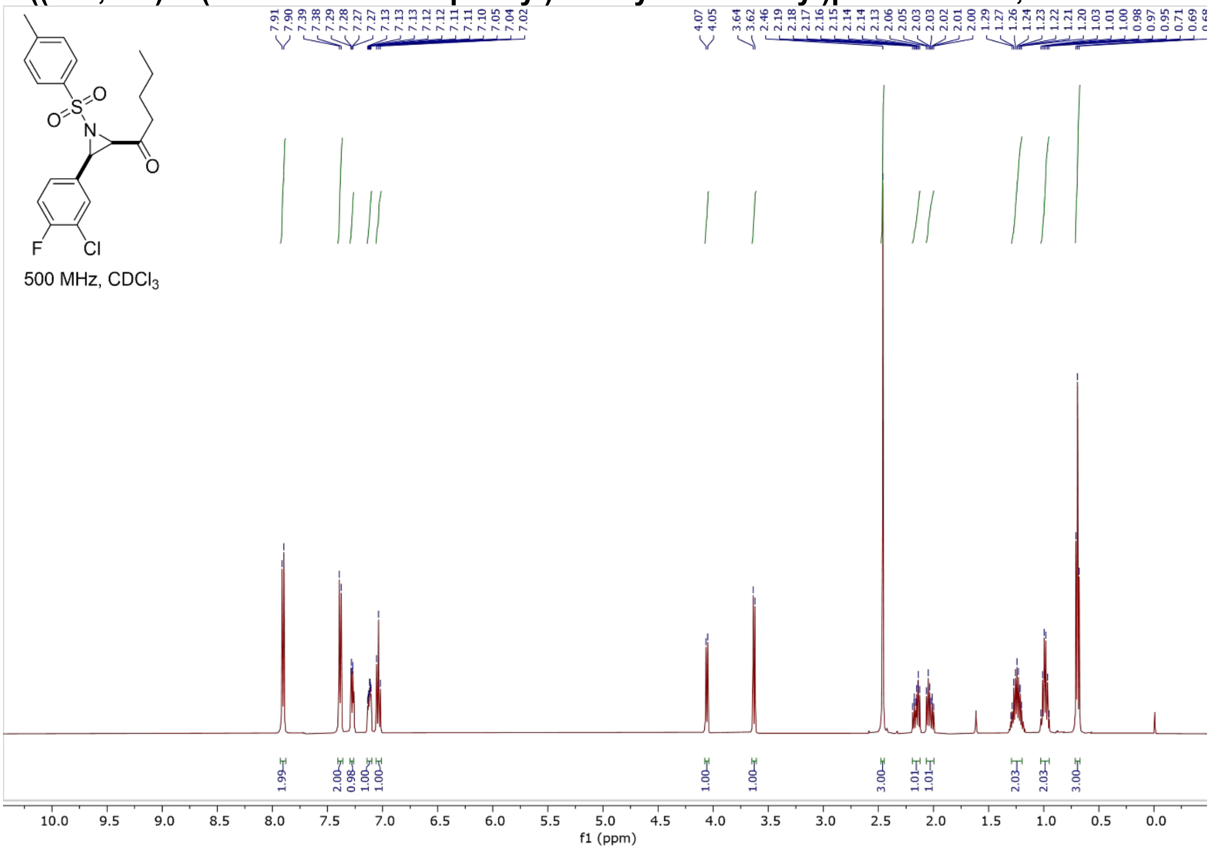

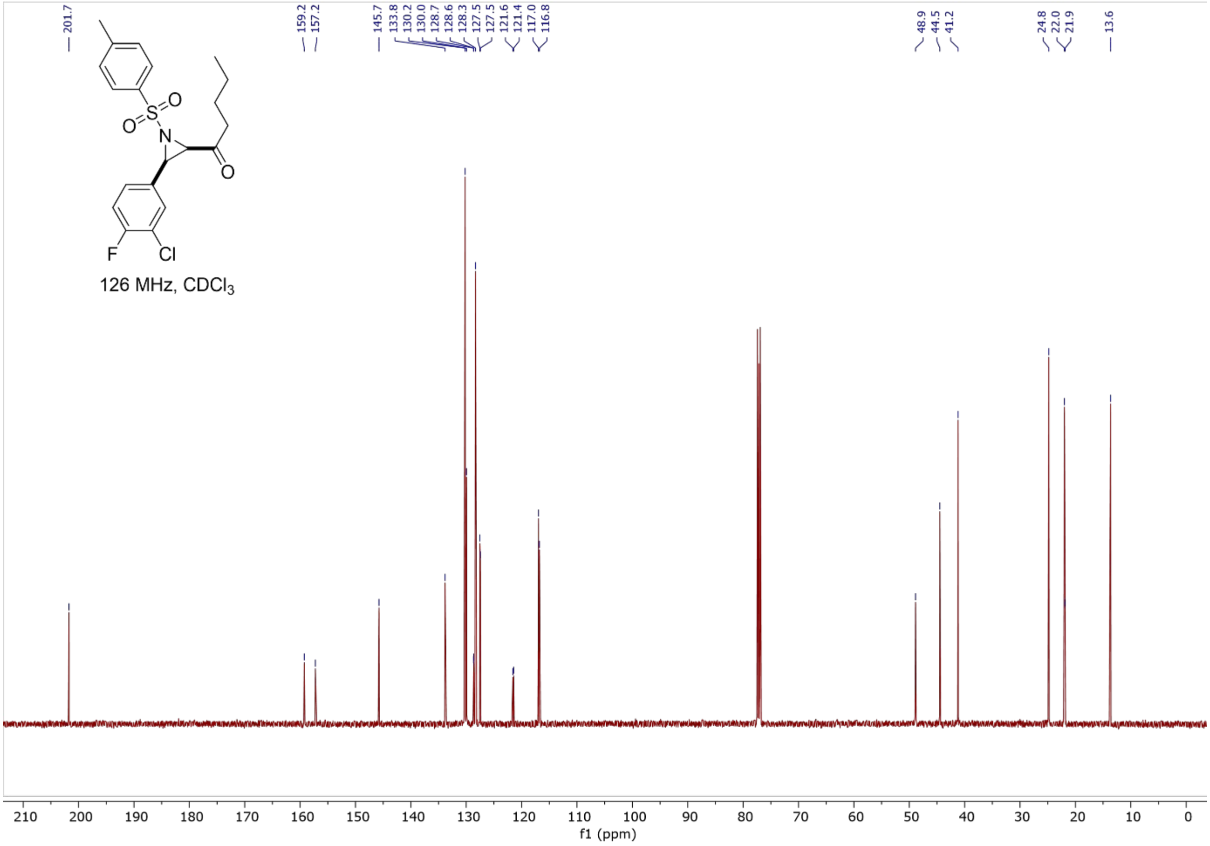

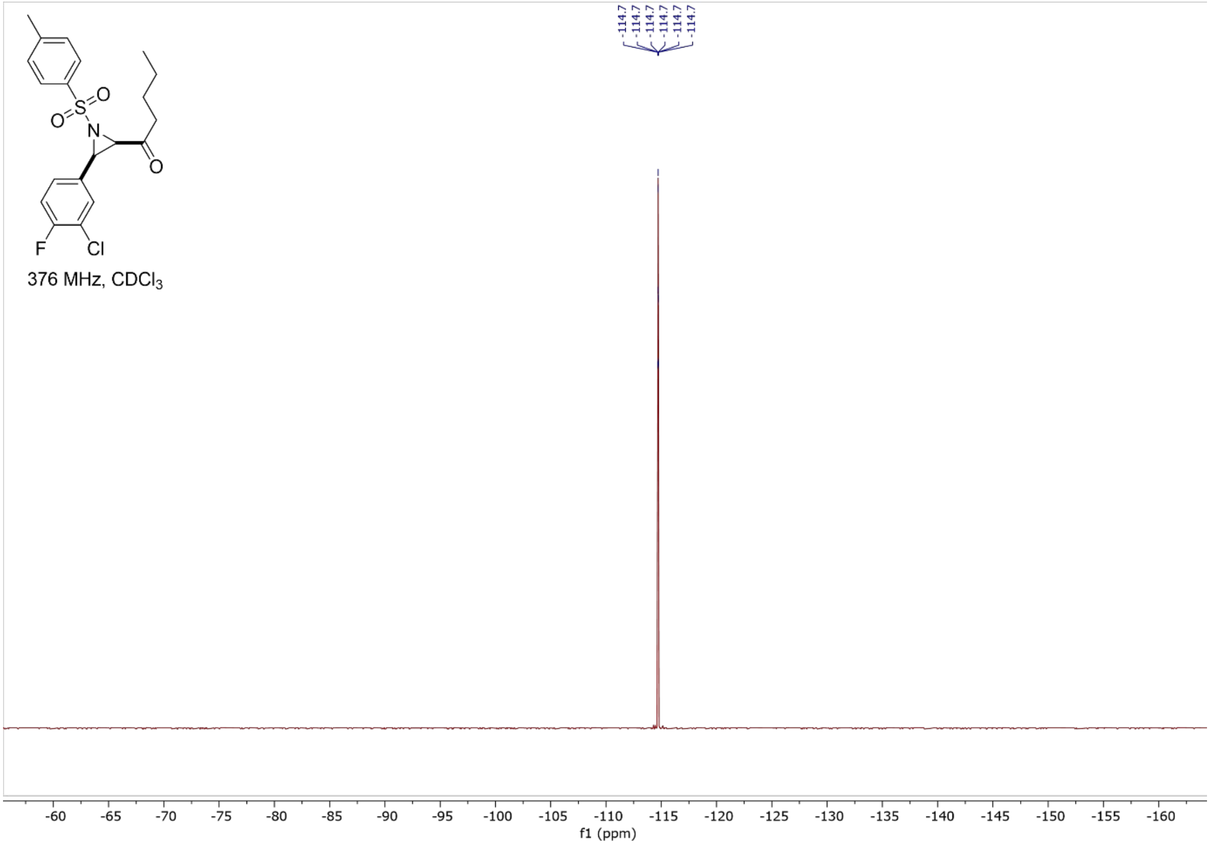

1-((2*R,3*R**)-1-Tosyl-3-(4-(trifluoromethyl)phenyl)aziridin-2-yl)pentan-1-one, 12f**

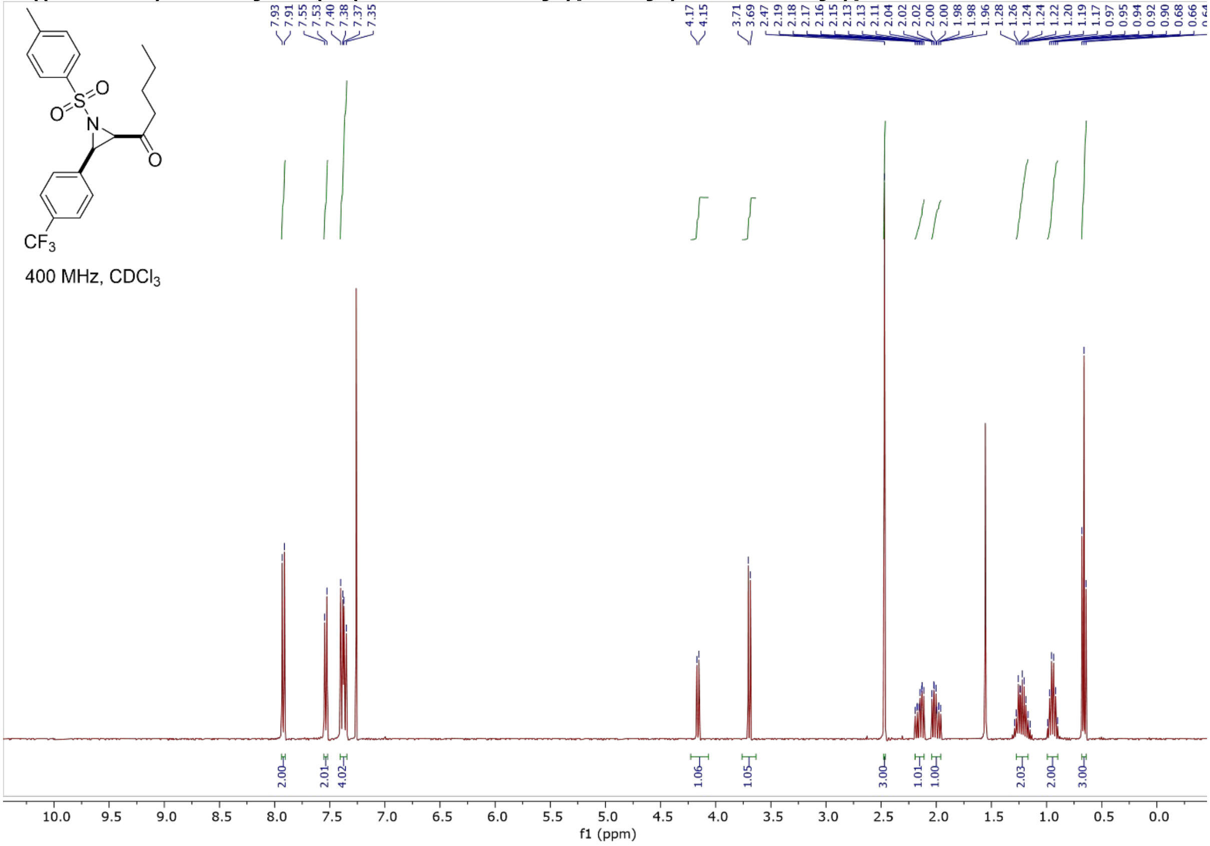

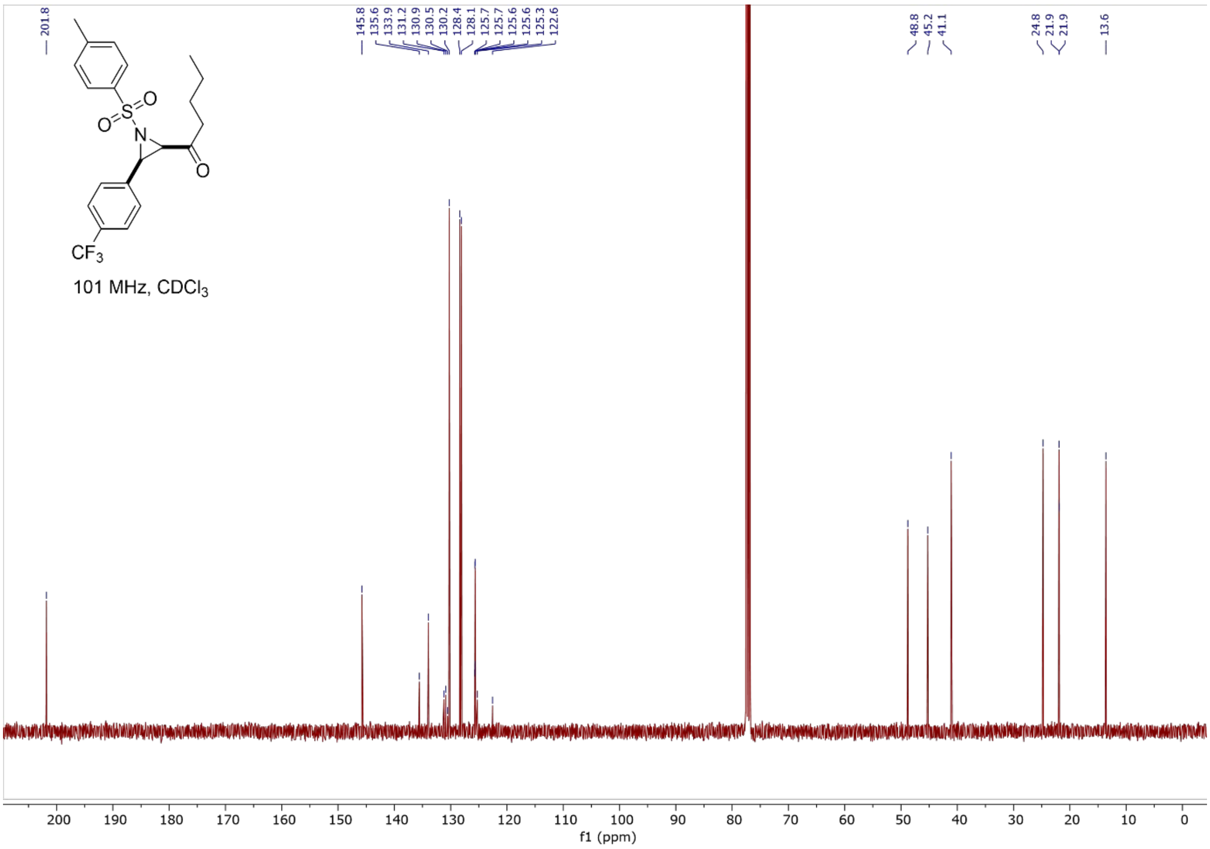
201.8
145.8
135.6
133.9
131.2
130.9
130.5
130.2
128.4
128.1
125.7
125.7
125.6
125.6
125.3
122.6
48.8
45.2
41.1
24.8
21.9
21.9
13.6
101 MHz, CDCl3
f1 (ppm)

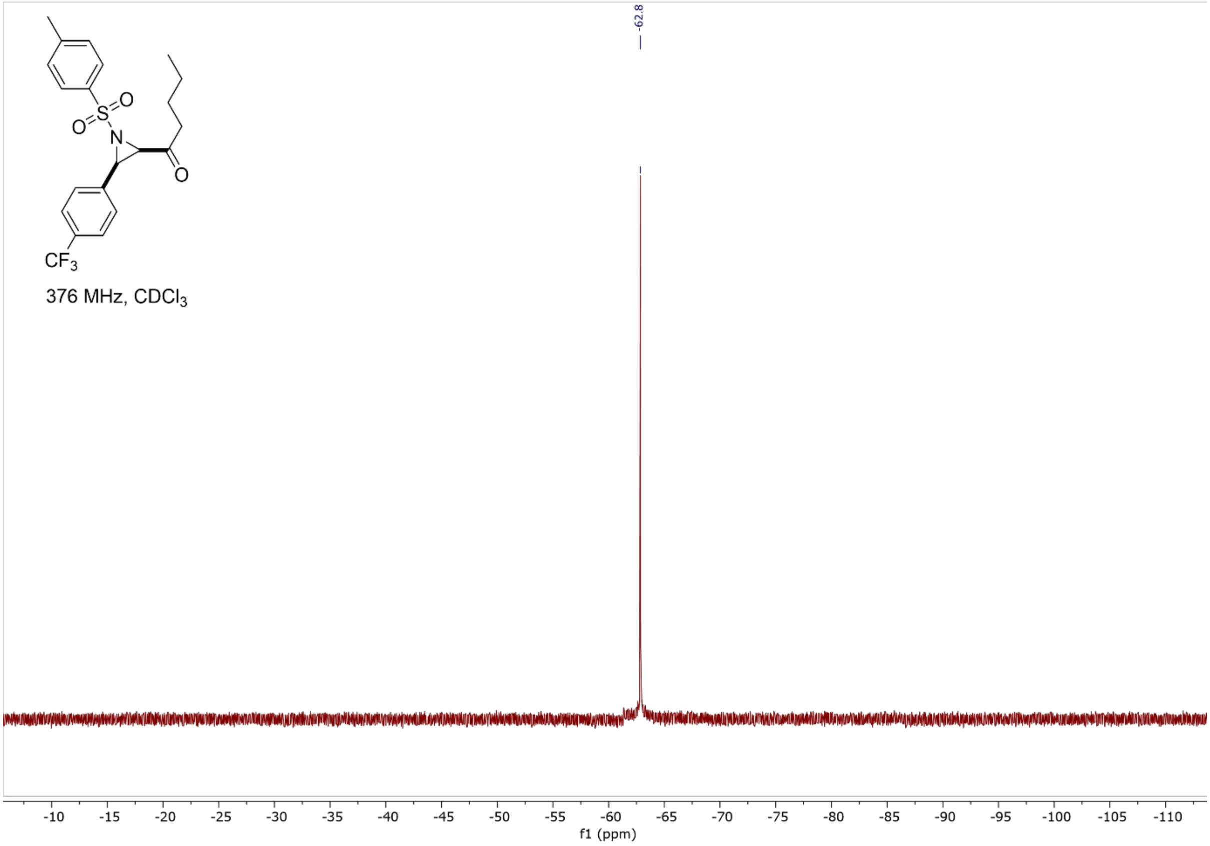
-62.8
376 MHz, CDCl3
f1 (ppm)

1-((2*R**,3*R**)-3-Phenyl-1-tosylaziridin-2-yl)pentan-1-one, 12g

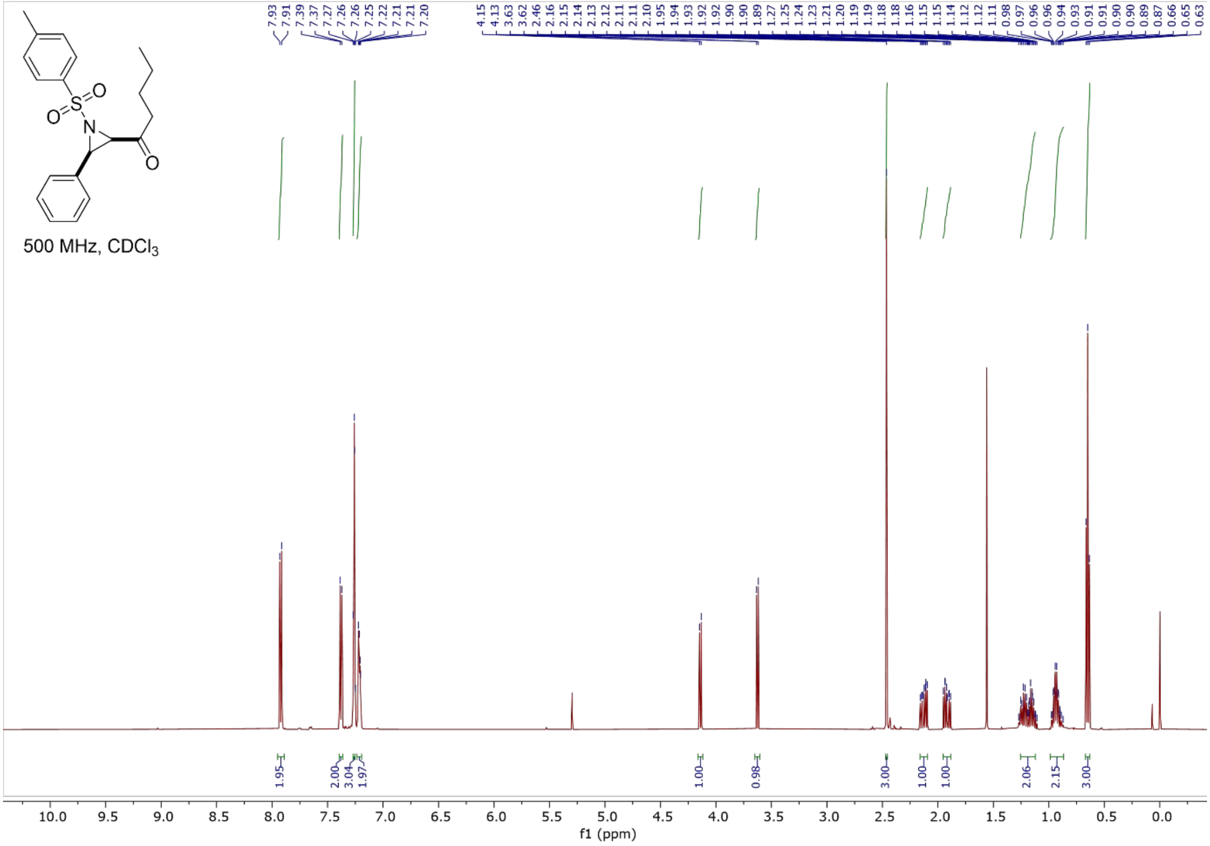

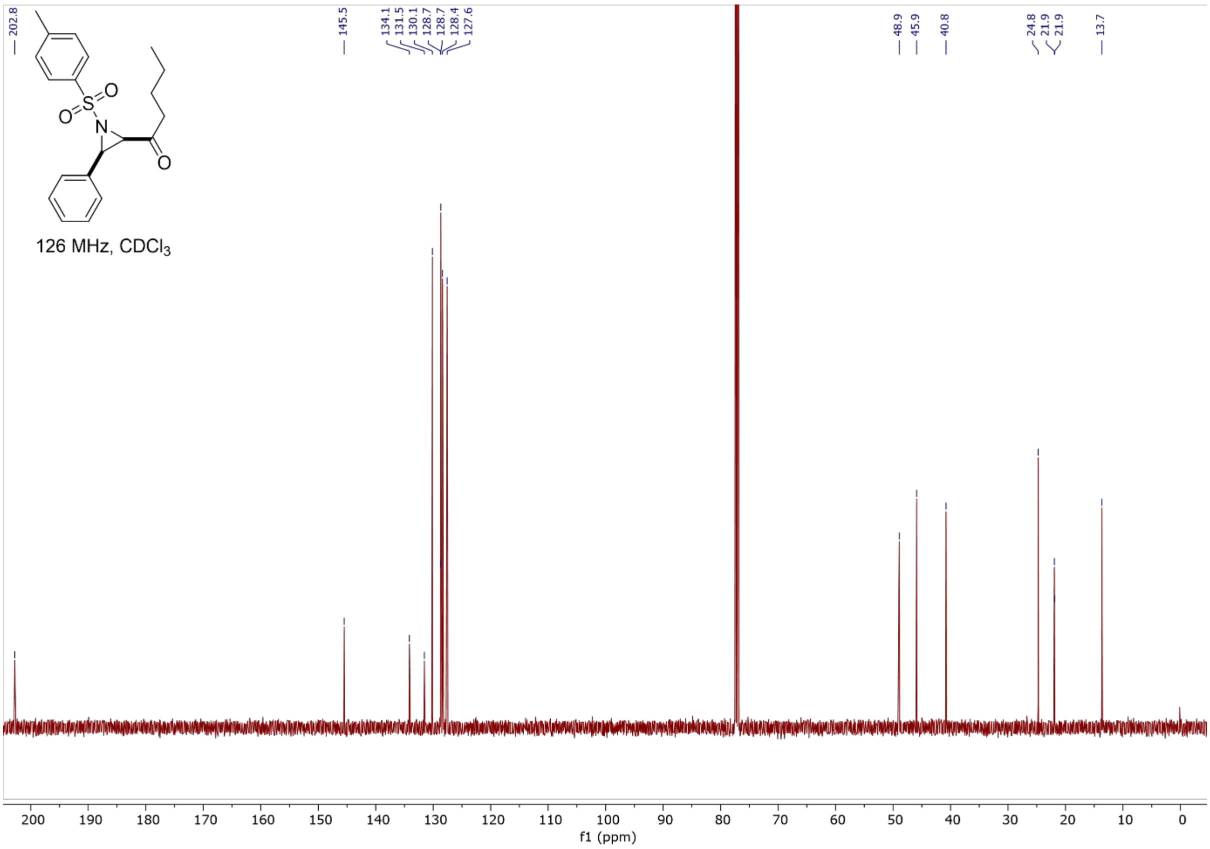

1-((2*R**,3*R**)-3-(3,4-Dimethylphenyl)-1-tosylaziridin-2-yl)pentan-1-one, 12h

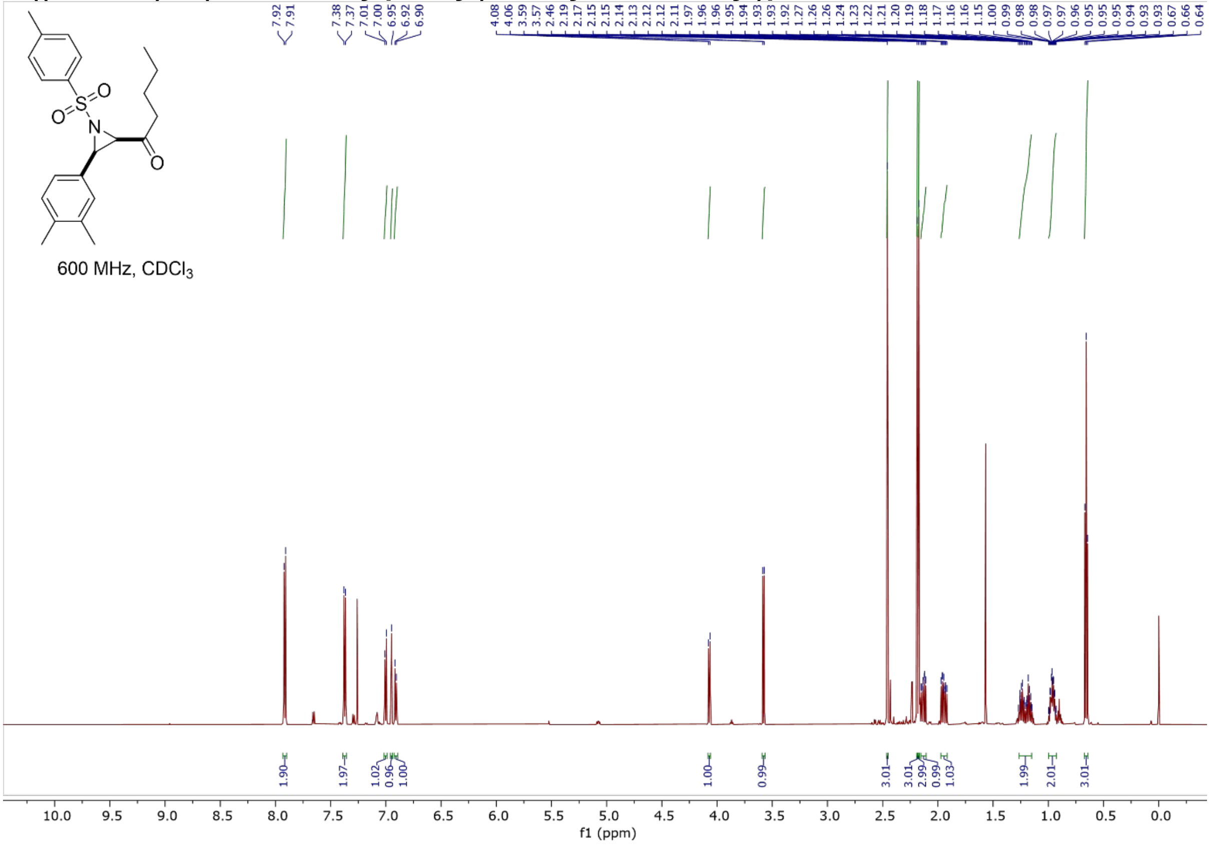

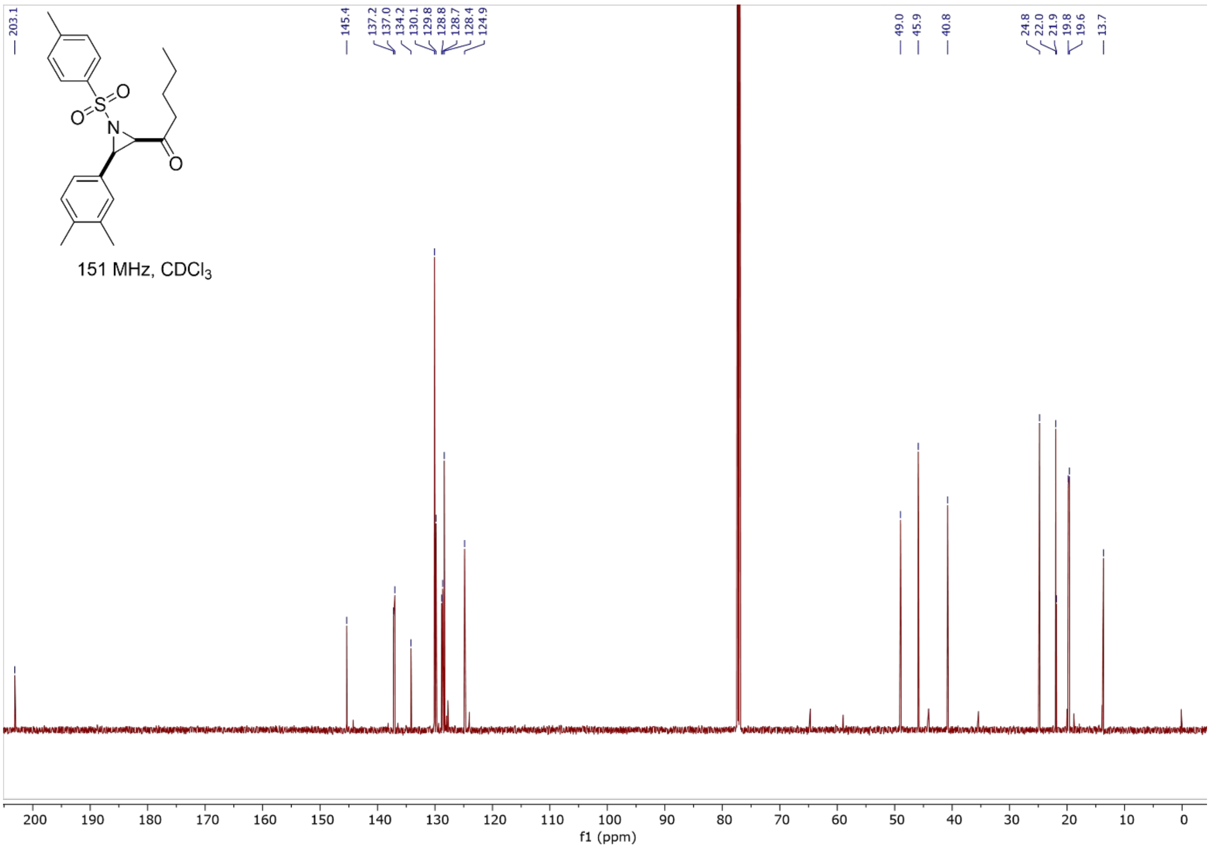

1-((2*R,3*R**)-3-(3,4-Dimethoxyphenyl)-1-tosylaziridin-2-yl)pentan-1-one, 12i**

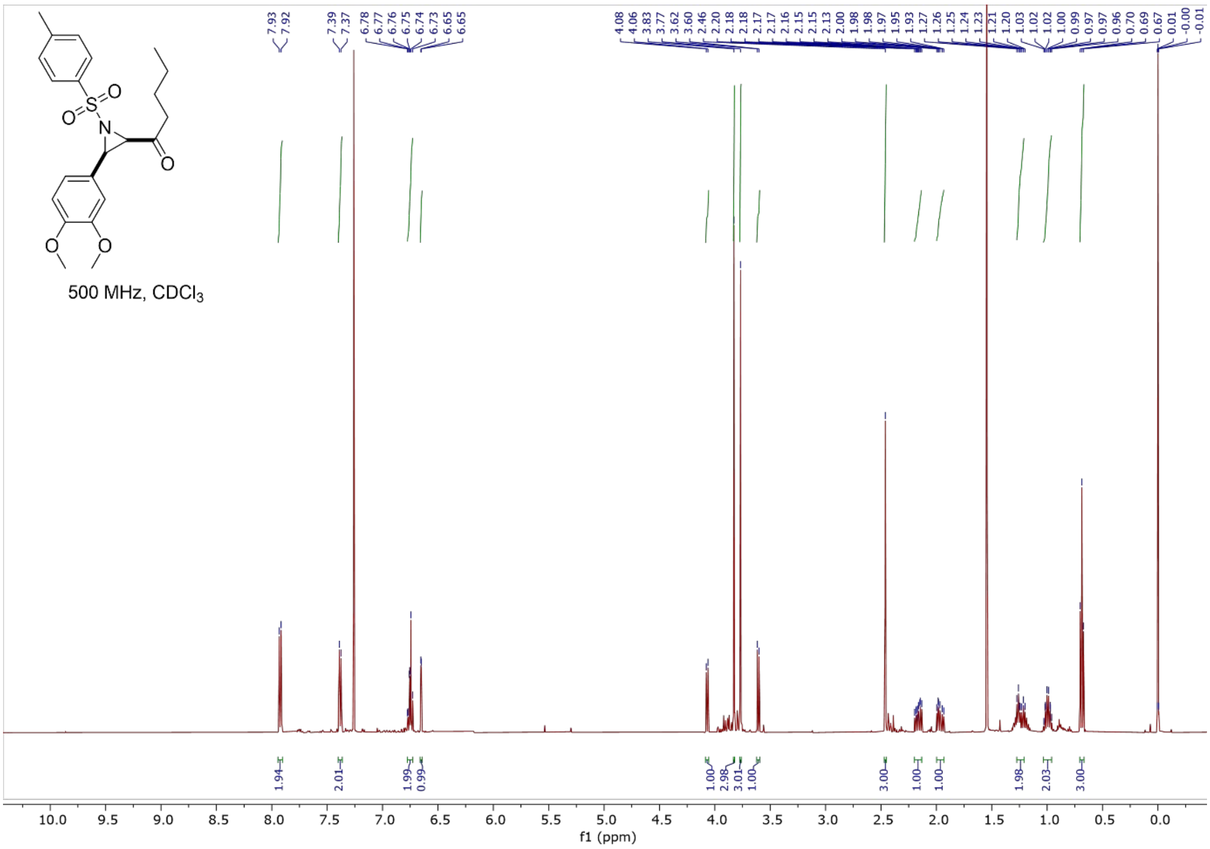

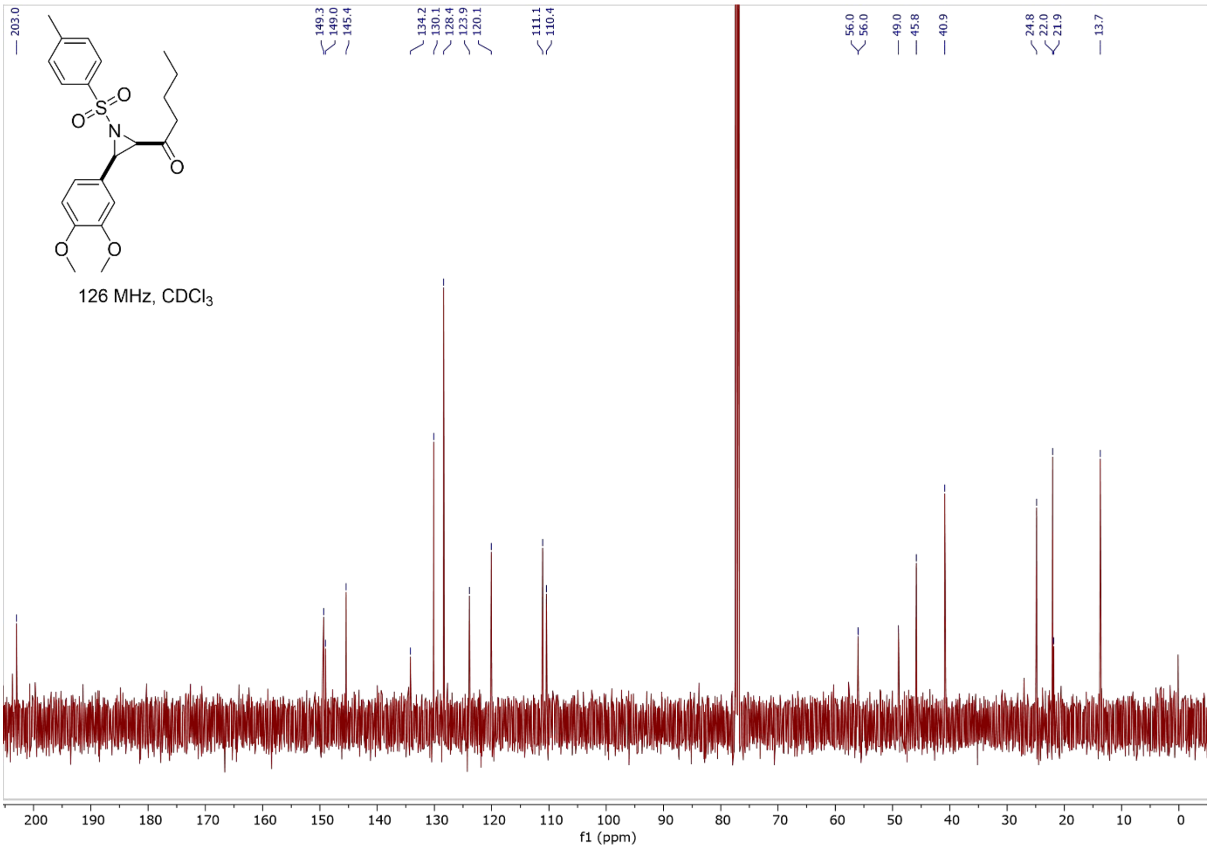

1-((2*R**,3*R**)-3-(Naphthalen-2-yl)-1-tosylaziridin-2-yl)pentan-1-one, 12k

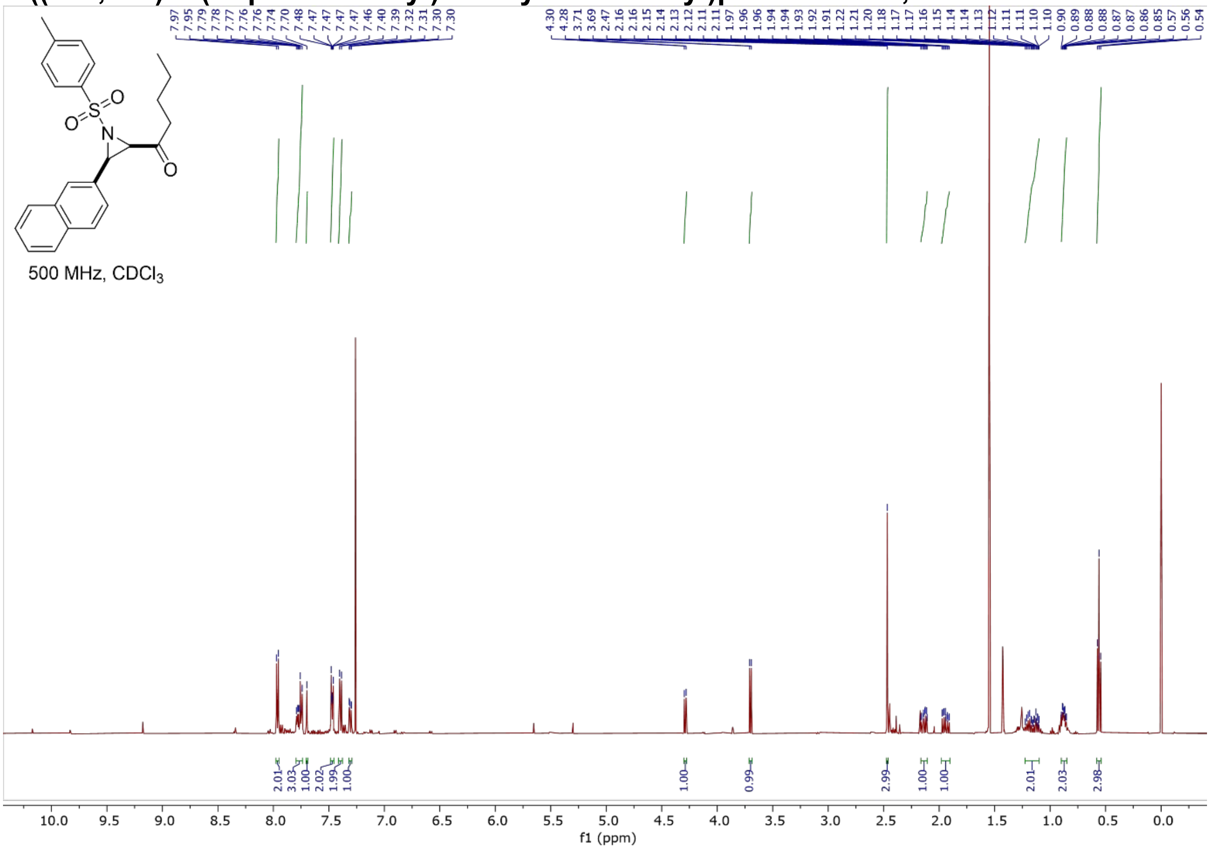

((2*R**,3*R**)-3-(2,4-Difluorophenyl)-1-tosylaziridin-2-yl)(cyclopropyl)methanone, 12l

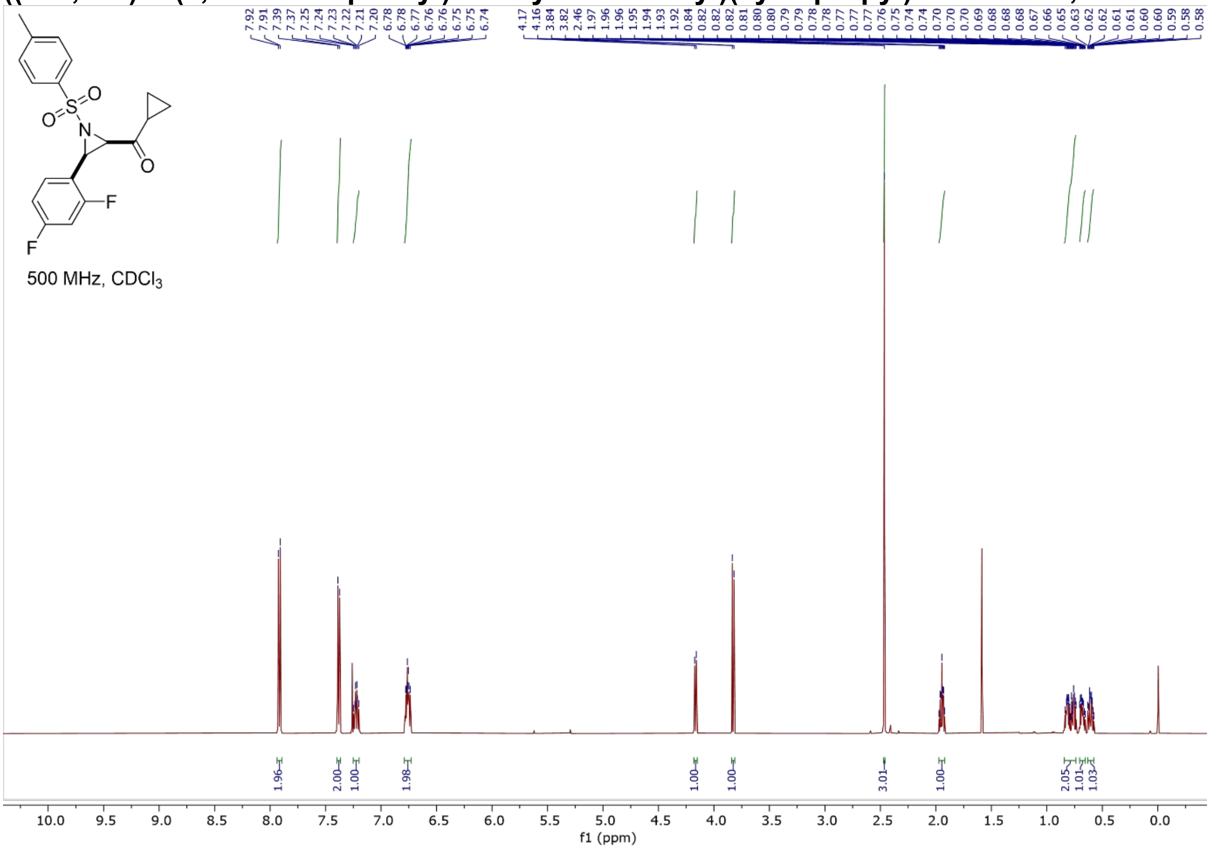

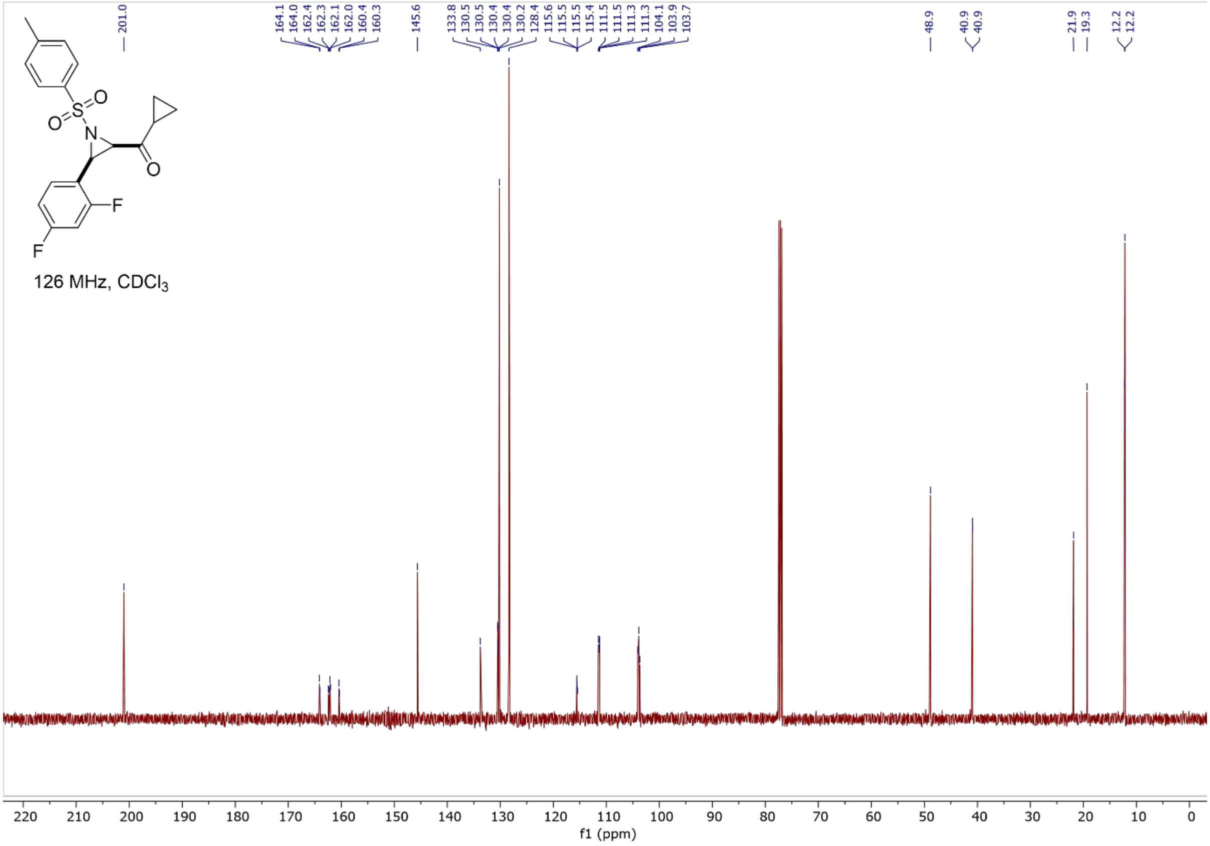
126 MHz, CDCl3
201.0
164.1
164.0
162.4
162.3
162.1
162.0
160.4
160.3
145.6
133.8
130.5
130.5
130.4
130.4
130.2
128.4
115.6
115.5
115.5
115.4
111.5
111.5
111.3
111.3
104.1
103.9
103.7
48.9
40.9
40.9
21.9
19.3
12.2
12.2
f1 (ppm)

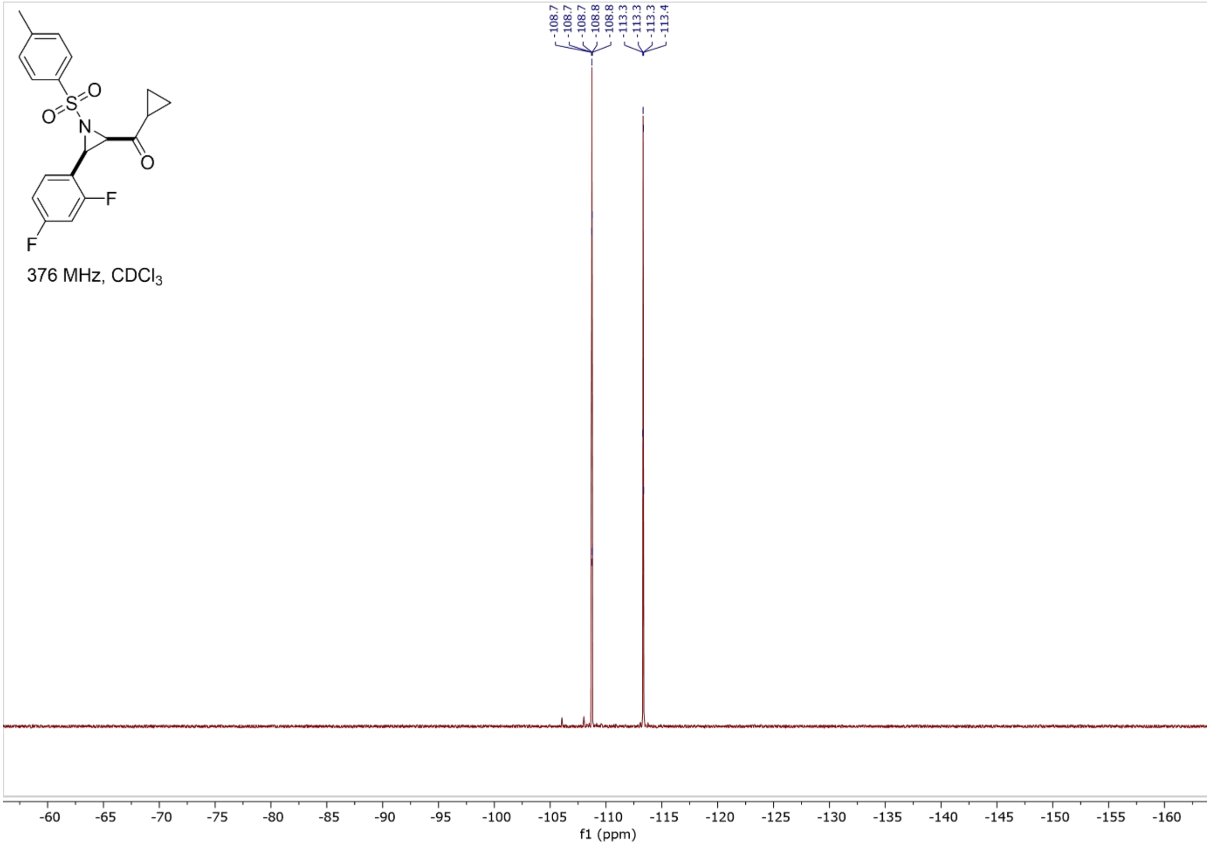
376 MHz, CDCl3
-108.7
-108.7
-108.7
-108.8
-108.8
-113.3
-113.3
-113.3
-113.4
f1 (ppm)

((2*R**,3*R**)-3-(4-Bromophenyl)-1-tosylaziridin-2-yl)(cyclopropyl)methanone, 12m

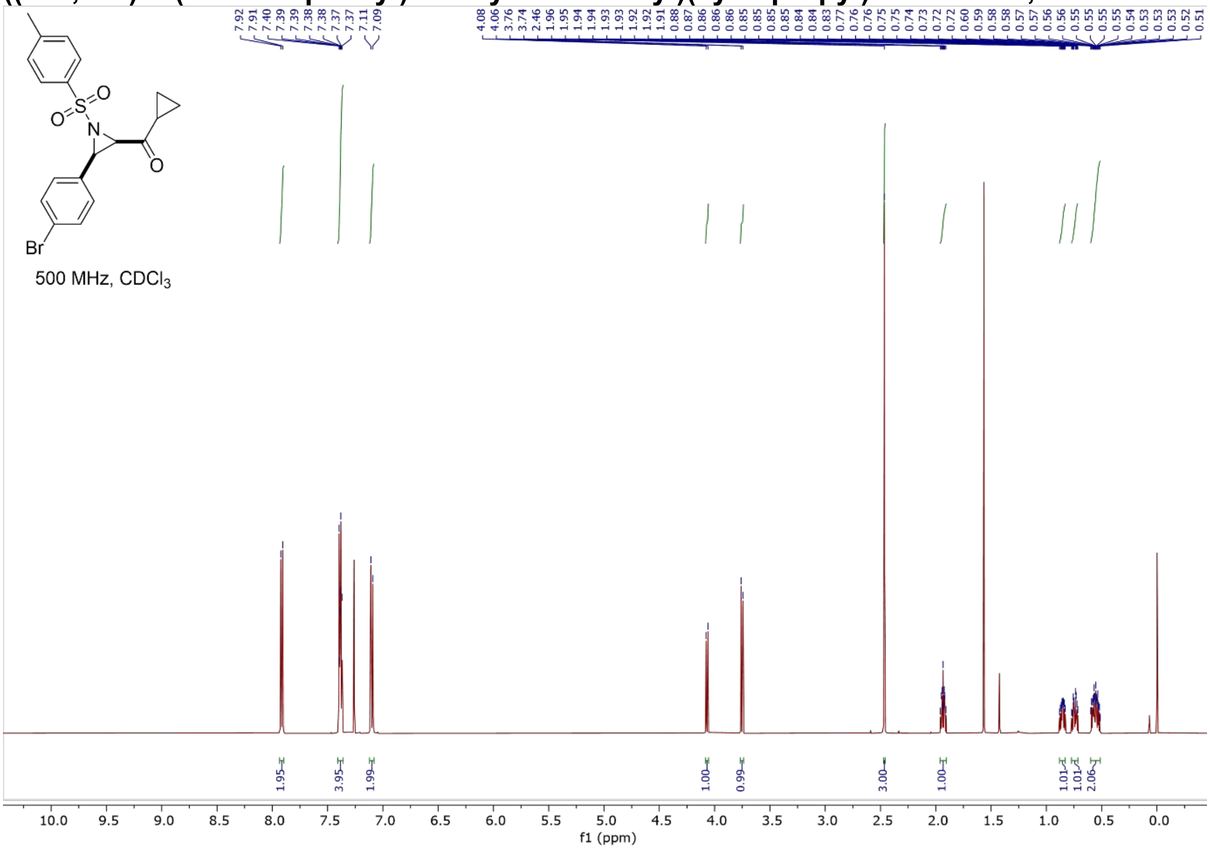

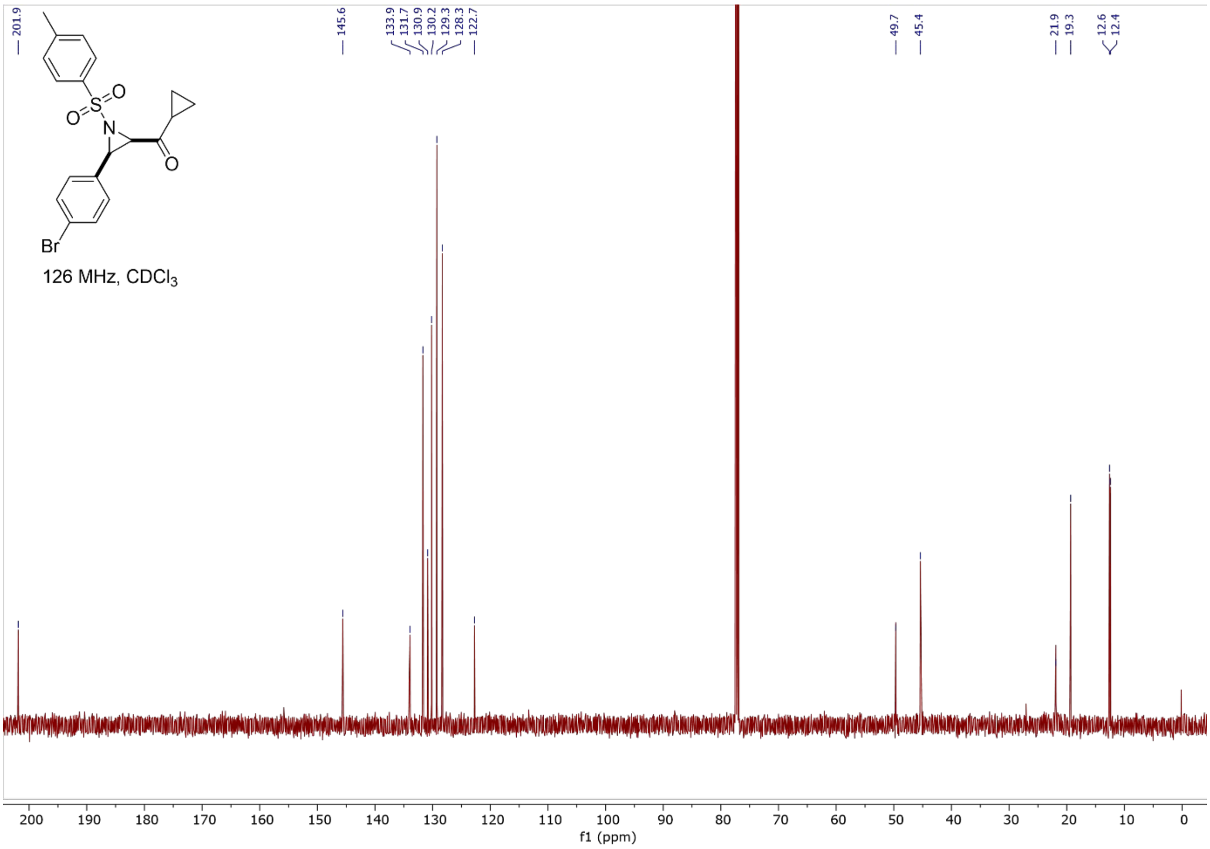

((2*R**,3*R**)-3-(Naphthalen-2-yl)-1-tosylaziridin-2-yl)(cyclopropyl)methanone, 12n

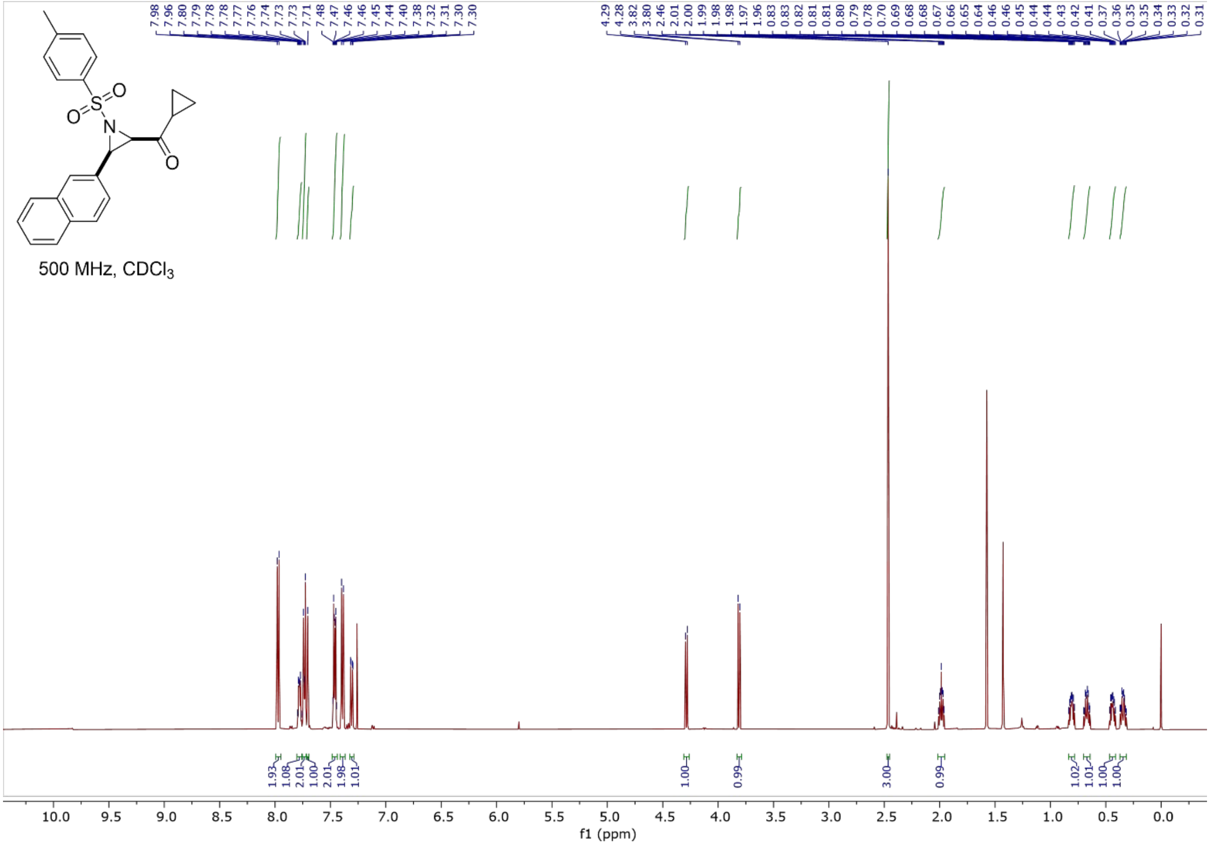

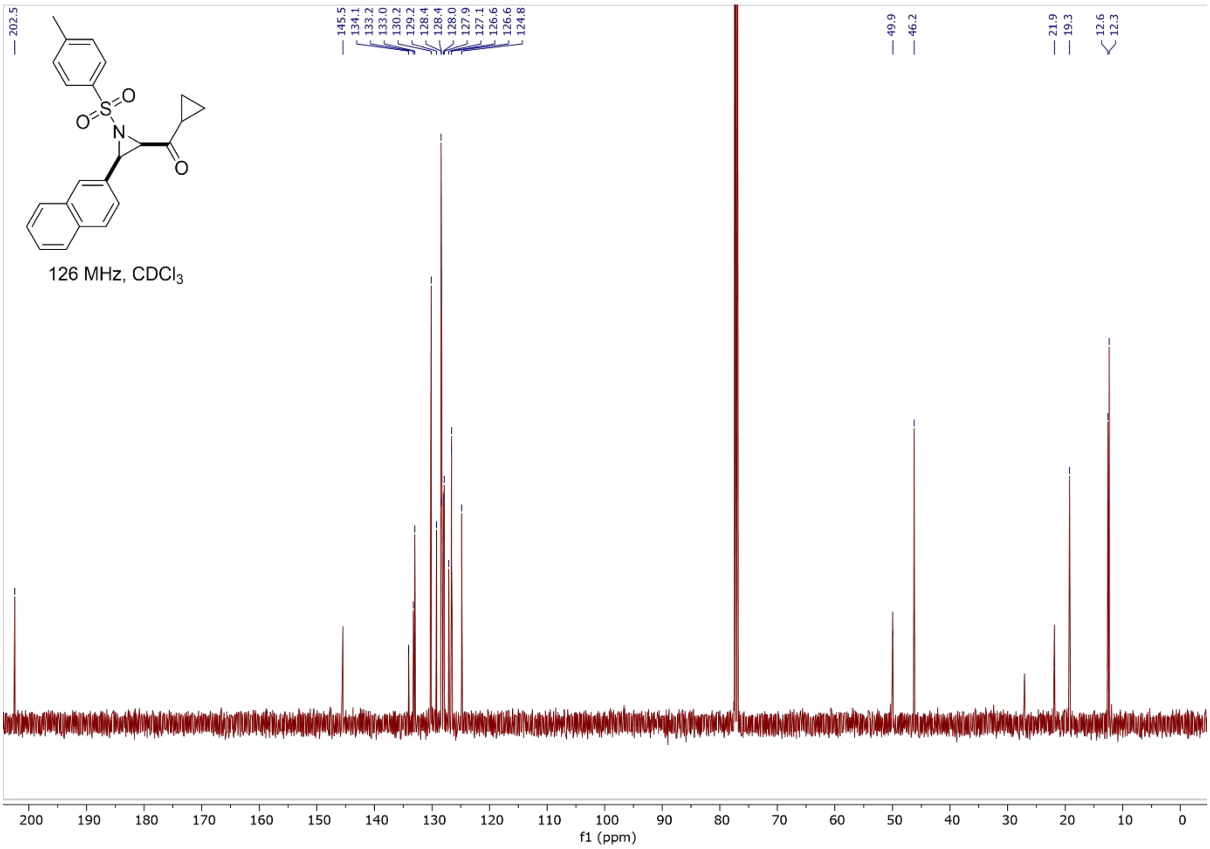

((2*R,3*R**)-3-(4-Fluorophenyl)-1-tosylaziridin-2-yl)(phenyl)methanone, 12p**

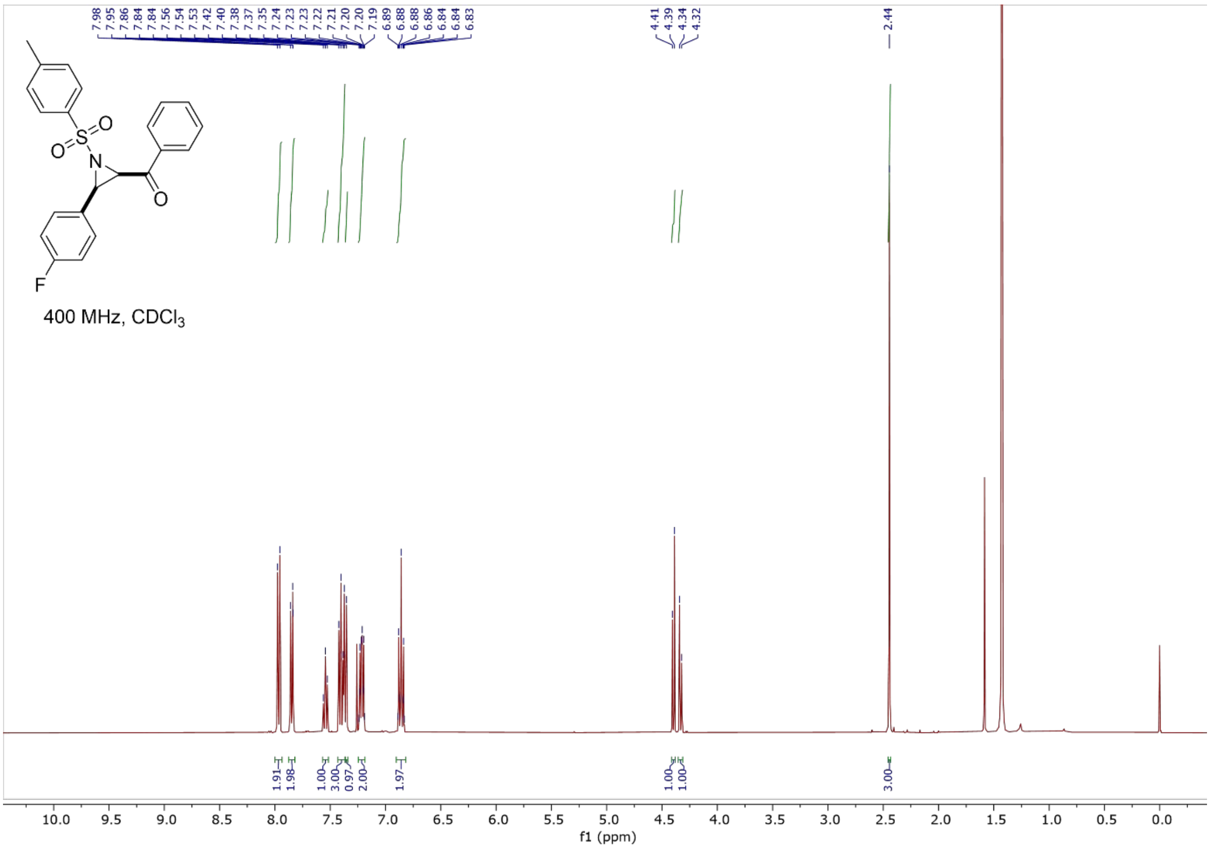

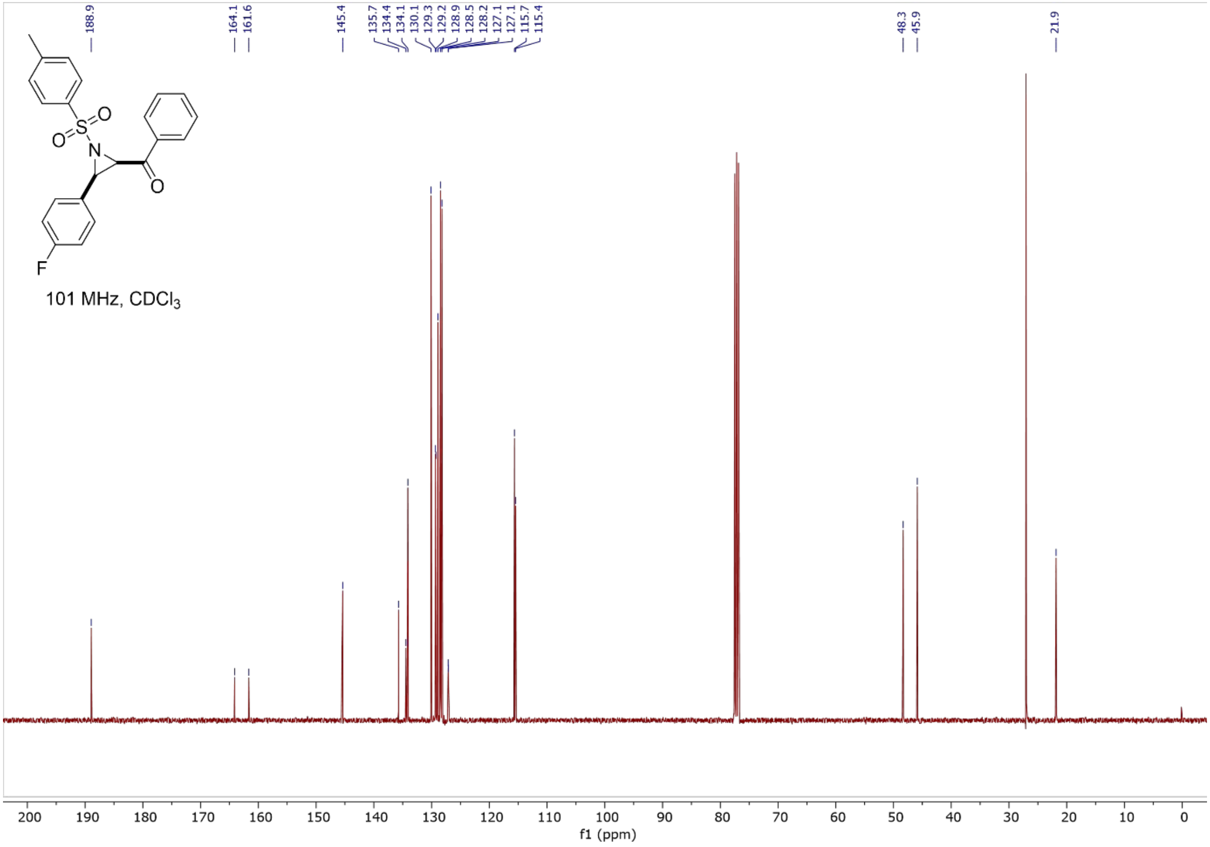

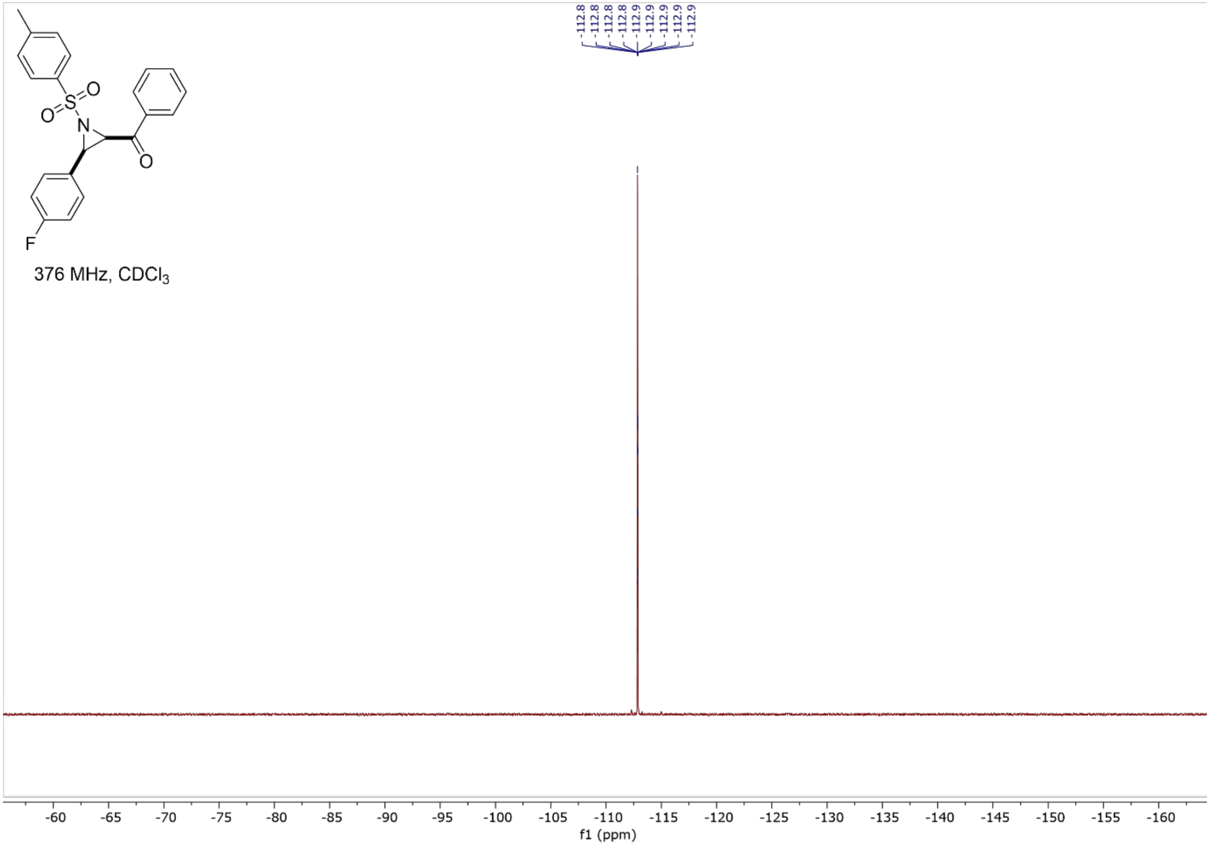

((2*R**,3*R**)-3-(4-Bromophenyl)-1-tosylaziridin-2-yl)(phenyl)methanone, 12q

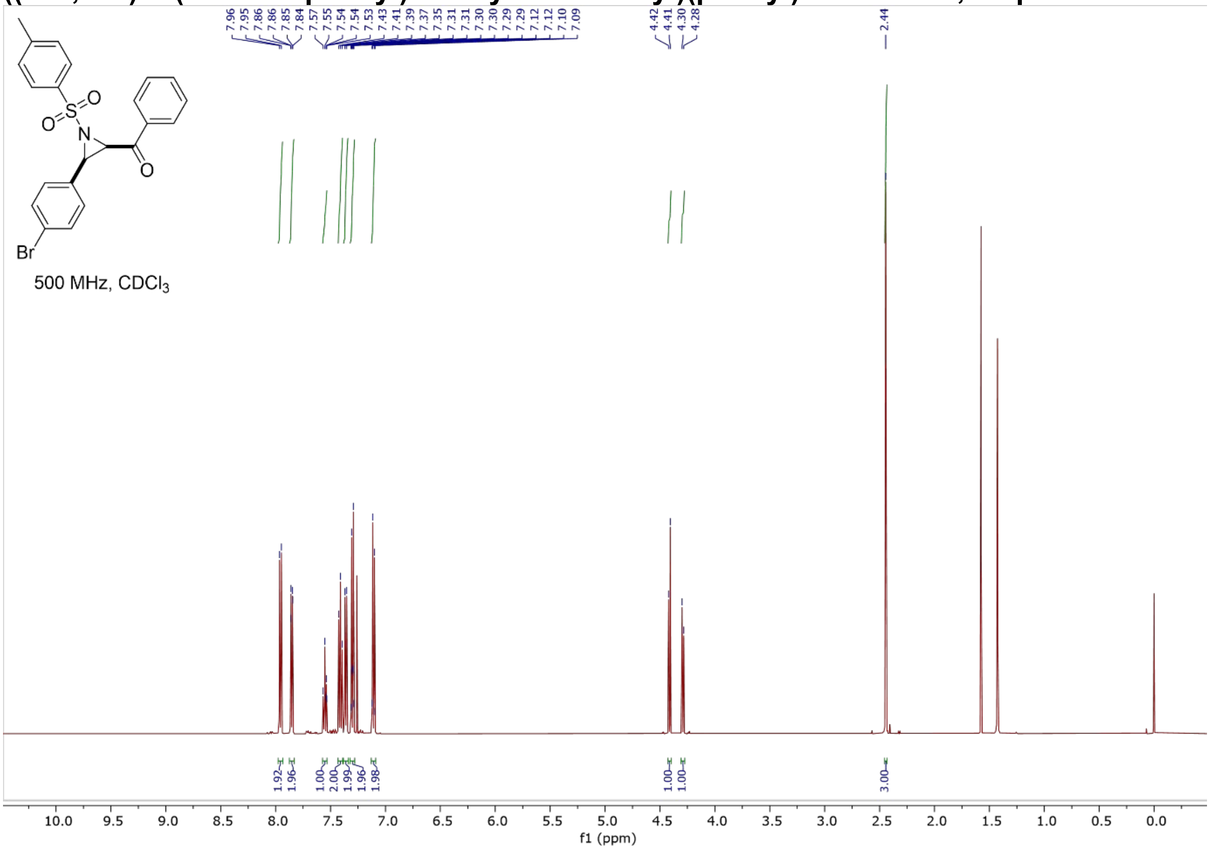

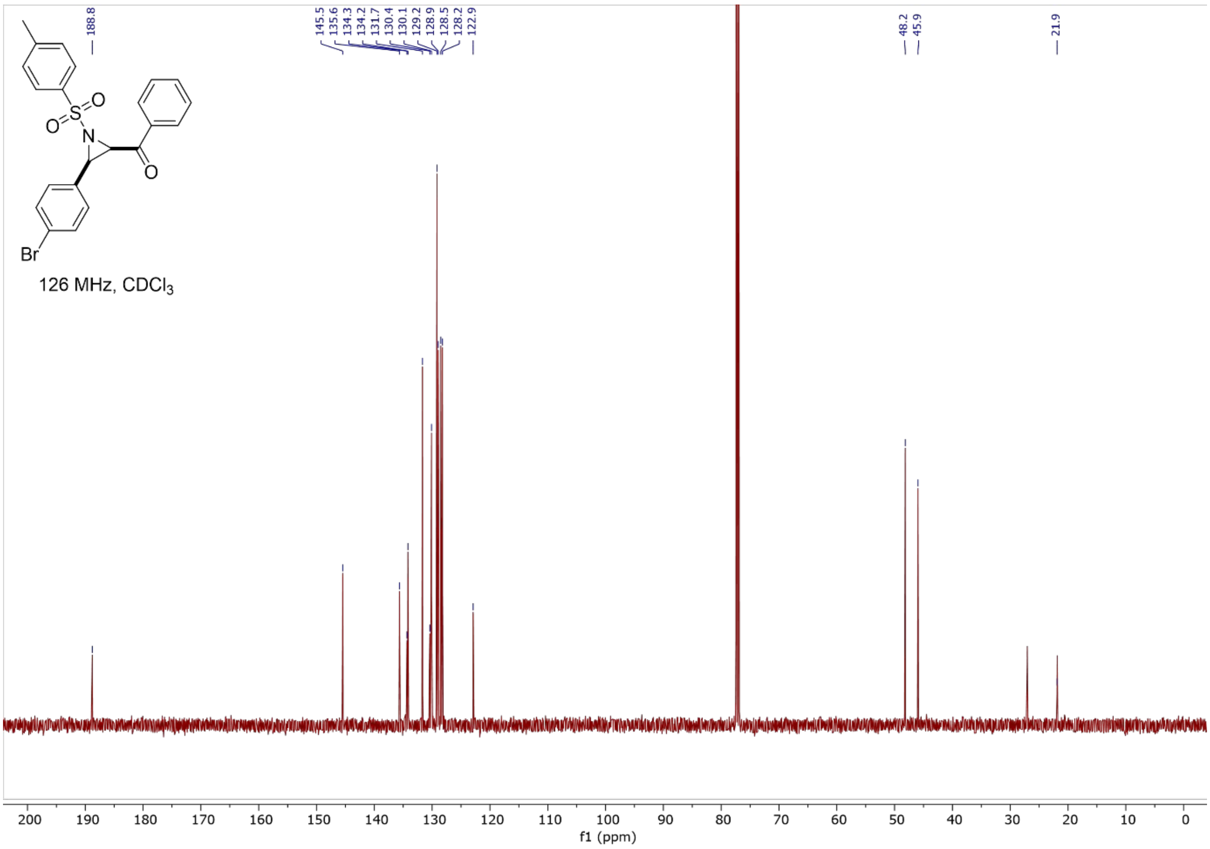

((2*R**,3*R**)-3-(Naphthalen-2-yl)-1-tosylaziridin-2-yl)(phenyl)methanone, 12r

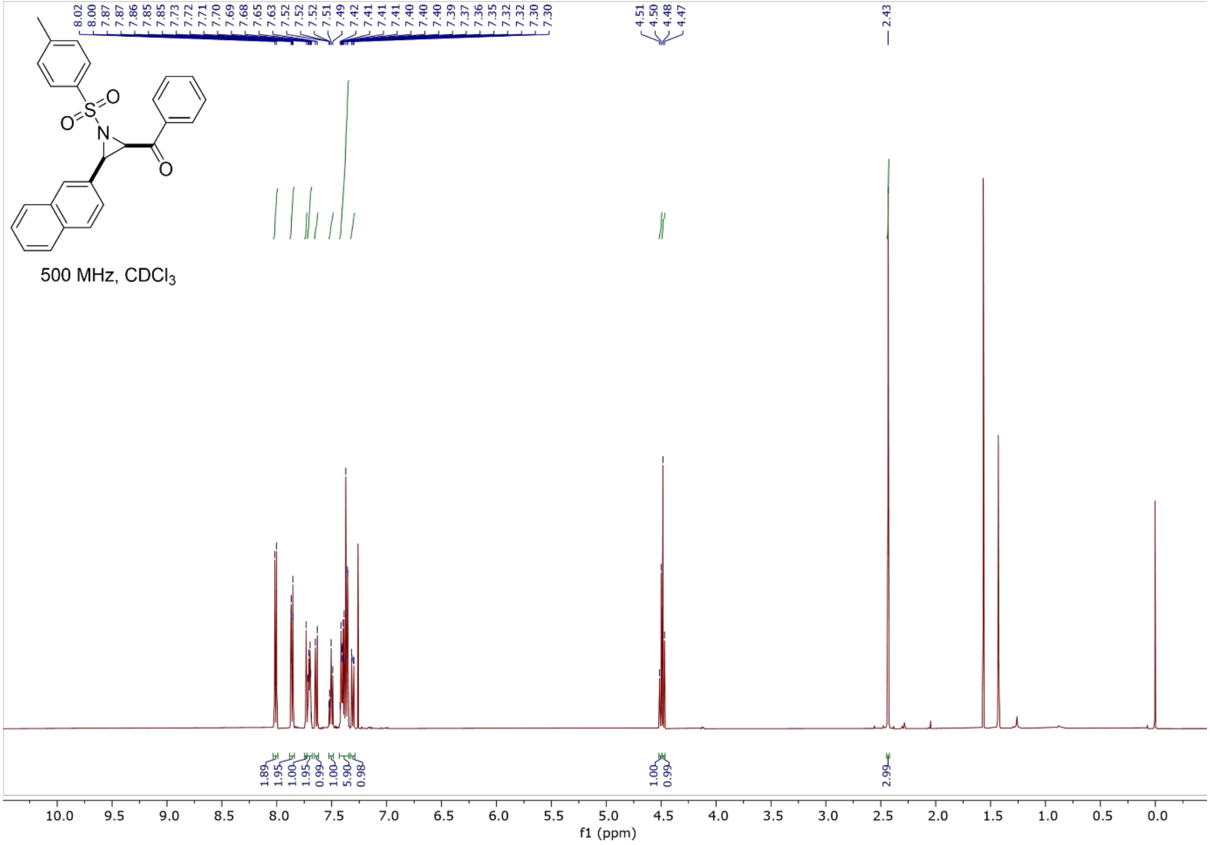

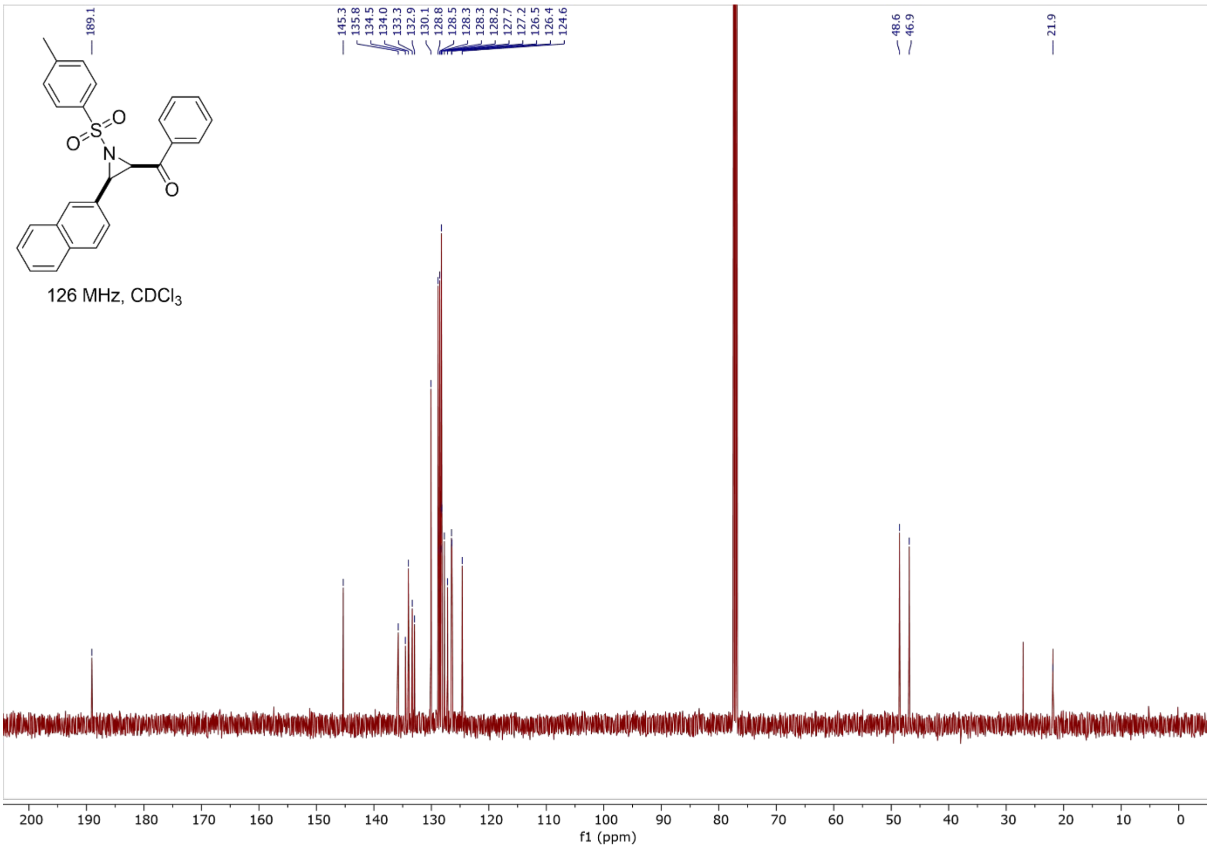

189.1
145.3
135.8
134.5
134.0
133.3
132.9
130.1
128.8
128.5
128.3
128.3
128.2
127.7
127.2
126.5
126.4
124.6
48.6
46.9
21.9
O
S
O
N
O
126 MHz, CDCl3
200 190 180 170 160 150 140 130 120 110 100 90 80 70 60 50 40 30 20 10 0
f1 (ppm)

6. References

1. Wang, A.; Venditto, N. J.; Darcy, J. W.; Emmert, M. H. Nondirected, Cu-Catalyzed sp^3 C-H Aminations with Hydroxylamine-Based Amination Reagents: Catalytic and Mechanistic Studies. *Organometallics*, **2017**, *36*, 1259-1268.
2. Han, W.; Su, J.; Mo, J. N.; Zhao, J. Photoredox Catalytic Phosphine-Mediated Deoxygenation of Hydroxylamines Enables the Construction of N-Acyliminophosphoranes. *Org. Lett.*, **2022**, *24*, 6247-6251.
3. Yan, W.; Wang, Q.; Chen, Y.; Petersen, J. L.; Shi, X. Iron-Catalyzed C−O Bond Activation for the Synthesis of Propargyl-1,2,3-triazoles and 1,1-Bis-triazoles. *Org. Lett.*, **2010**, *12*, 3308-3311.
4. Gayon, E.; Debleds, O.; Nicouleau, M.; Lamaty, F.; van der Lee, A.; Vrancken, E.; Campagne, J. M. Highly Diastereoselective Baldwin Rearrangement of Isoxazolines into cis-Acylaziridines. *J. Org. Chem.*, **2010**, *75*, 6050-6053.
5. Zhao, M.; Mohr, J. T. Vanadium(V)-mediated rearrangement/halogenation cascade: Synthesis of α-haloenones from propargyl alcohols. *Tetrahedron*, **2017**, *73*, 4115-4124.
6. Laserna, V.; Porter, M. J.; Sheppard, T. D. Gold Catalyzed Hydroamination of Propargylic Alcohols: Controlling Divergent Reaction Pathways to Access 1,3-Aminoalcohols, 3-Hydroxyketones or 3-Aminoketones. *J. Org. Chem.*, **2019**, *84*, 11391-11406.
7. Pennell, M. N.; Turner, P. G.; Sheppard, T. D. Gold- and Silver-Catalyzed Reactions of Propargylic Alcohols in the Presence of Protic Additives. *Chem. Eur. J.*, **2012**, *18*, 4748-4758.
8. Gibson, S. M.; D'Oyley, J. M.; Higham, J. I.; Sanders, K.; Laserna, V.; Aliev, A. E.; Sheppard, T. D. Dihalohydration of Alkynols: A Versatile Approach to Diverse Halogenated Molecules. *Eur. J. Org. Chem.*, **2018**, *2018*, 4018-4028.
9. Trost, B. M.; Luan, X. Contemporaneous Dual Catalysis by Coupling Highly Transient Nucleophilic and Electrophilic Intermediates Generated in Situ. *J. Am. Chem. Soc.*, **2011**, *133*, 1706-1709.
10. Tarigopula, C.; Manojveer, S.; Balamurugan, R. Synthesis of Highly Substituted Biaryls by the Construction of a Benzene Ring via In Situ Formed Acetals. *J. Org. Chem.*, **2021**, *86*, 11871-11883.
11. Jeong, Y.; Kim, B. I.; Lee, J. K.; Ryu, J. S. Direct Synthesis of 4-Fluoroisoxazoles through Gold-Catalyzed Cascade Cyclization–Fluorination of 2-Alkynone O-Methyl Oximes. *J. Org. Chem.*, **2014**, *79*, 6444-6455.

12. Downey, C. W.; Mahoney, B. D.; Lipari, V. R. Trimethylsilyl Trifluoromethanesulfonate-Accelerated Addition of Catalytically Generated Zinc Acetylides to Aldehydes. *J. Org. Chem.*, **2009**, *74*, 2904-2906.
13. Dai, M.; Sun, Z.; Chen, L. A. Palladium-Catalyzed Regiodivergent Synthesis of 1,3-Dienyl and Allyl Esters from Propargyl Esters. *Angew. Chem. Int. Ed.*, **2022**, *61*, e202203835.
14. Pennell, M. N.; Kyle, M. P.; Gibson, S. M.; Male, L.; Turner, P. G.; Grainger, R. S.; Sheppard, T. D. Intercepting the Gold-Catalysed Meyer–Schuster Rearrangement by Controlled Protodemetallation: A Regioselective Hydration of Propargylic Alcohols. *Adv. Synth. Catal.*, **2016**, *358*, 1519-1525.
15. Aizawa, K.; Nakagawa, H.; Matsuo, K.; Kawai, K.; Ieda, N.; Suzuki, T.; Miyata, N. Piloty's acid derivative with improved nitroxyl-releasing characteristics. *Bioorg. Med. Chem. Lett.*, **2013**, *23*, 2340-2343.
16. Chakraborty Ghosal, N.; Mahato, S.; Chatterjee, R.; Santra, S.; Zyryanov, G. V.; Majee, A. A Mild and Efficient Method for the Syntheses and Regioselective Ring-Opening of Aziridines. *SynOpen*, **2017**, 01, 15-23.
17. Aichhorn, S.; Gururaja, G. N.; Reisinger, M.; Waser, M. Scope and limitations of diastereoselective aziridination reactions using stabilised ammonium ylides or α-bromo carbonyl nucleophiles. *RSC Adv.*, **2013**, *3*, 4552-4557.
